# Uncovering Genes with Divergent mRNA-Protein Dynamics in *Streptomyces coelicolor*

**DOI:** 10.1371/journal.pone.0002097

**Published:** 2008-05-07

**Authors:** Karthik P. Jayapal, Robin J. Philp, Yee-Jiun Kok, Miranda G. S. Yap, David H. Sherman, Timothy J. Griffin, Wei-Shou Hu

**Affiliations:** 1 Department of Chemical Engineering and Materials Science, University of Minnesota, Minneapolis, Minnesota, United States of America; 2 Bioprocessing Technology Institute, Agency for Science Technology and Research, Singapore, Singapore; 3 Life Sciences Institute, Department of Medicinal Chemistry, University of Michigan, Ann Arbor, Michigan, United States of America; 4 Life Sciences Institute, Department of Chemistry, University of Michigan, Ann Arbor, Michigan, United States of America; 5 Life Sciences Institute, Department of Microbiology and Immunology, University of Michigan, Ann Arbor, Michigan, United States of America; 6 Department of Biochemistry, Molecular Biology and Biophysics, University of Minnesota, Minneapolis, Minnesota, United States of America; Centre de Regulació Genòmica, Spain

## Abstract

Many biological processes are intrinsically dynamic, incurring profound changes at both molecular and physiological levels. Systems analyses of such processes incorporating large-scale transcriptome or proteome profiling can be quite revealing. Although consistency between mRNA and proteins is often implicitly assumed in many studies, examples of divergent trends are frequently observed. Here, we present a comparative transcriptome and proteome analysis of growth and stationary phase adaptation in *Streptomyces coelicolor*, taking the time-dynamics of process into consideration. These processes are of immense interest in microbiology as they pertain to the physiological transformations eliciting biosynthesis of many naturally occurring therapeutic agents. A shotgun proteomics approach based on mass spectrometric analysis of isobaric stable isotope labeled peptides (iTRAQ™) enabled identification and rapid quantification of approximately 14% of the theoretical proteome of *S. coelicolor*. Independent principal component analyses of this and DNA microarray-derived transcriptome data revealed that the prominent patterns in both protein and mRNA domains are surprisingly well correlated. Despite this overall correlation, by employing a systematic concordance analysis, we estimated that over 30% of the analyzed genes likely exhibited significantly divergent patterns, of which nearly one-third displayed even opposing trends. Integrating this data with biological information, we discovered that certain groups of functionally related genes exhibit mRNA-protein discordance in a similar fashion. Our observations suggest that differences between mRNA and protein synthesis/degradation mechanisms are prominent in microbes while reaffirming the plausibility of such mechanisms acting in a concerted fashion at a protein complex or sub-pathway level.

## Introduction

Large scale transcriptome profiling using DNA microarrays has afforded us with an unprecedented ability to examine cellular dynamics in great detail. These changes, generally portrayed as sets of genes being up- or down-regulated, give clues to the regulation of many biological processes. Implicit in many such analyses is often an assumption that the dynamics of the translational product of genes parallels that of the transcripts. Although such correlations do exist in numerous cases, discordance between mRNA and proteins are not quite uncommon. Previous studies exploring disparities between transcriptome and proteome data [1]–[6] have largely employed direct comparisons of mRNA and protein expression ratios between each sample pair. However, for dynamic processes, a more rational approach would entail a comparison of temporal profiles arising from the time-dynamics of each mRNA and protein. We show here that such a comparative transcriptome and proteome analysis with a consideration for temporal dynamics can yield intriguing insights into the process of growth and stationary phase adaptation in *Streptomyces* spp.

Streptomycetes are a group of gram-positive mycelial bacteria which are capable of synthesizing an amazingly diverse repertoire of potent biomolecular agents. These multicellular differentiating prokaryotes belong to the genera actinomycetes, a class of common soil microbes that produce over two-thirds of the world's antibiotics [7]. Natural products such as antibiotics are typically synthesized in a relatively quiescent stationary phase following cessation of rapid growth, when the cells direct their metabolism toward survival and long-term propagation. This capability of streptomycetes has been extensively exploited in large-scale industrial fermentation processes for synthesizing a variety of therapeutic natural products and other biomolecules. Growth-phase dependent gene expression in *Streptomyces*, therefore, merits attention both from a fundamental, as well as a commercial perspective.

*S. coelicolor* is the most widely studied streptomycete and its genome encodes a relatively large number of genes compared to other bacteria [8]. This includes numerous regulators like sigma factors which play key roles in orchestrating global gene expression pattern shifts through transcriptional regulation. Although transcriptional control remains as one of the primary means of gene expression regulation in prokaryotes, *Streptomyces* spp. are known to employ some post-transcriptional regulatory mechanisms. The best known example thus far in *S. coelicolor* is, perhaps, the probable translational control of over 140 genes containing a rare leucine TTA codon (including antibiotic and developmental regulators) by growth dependent expression of the sole tRNA (*bldA*) that can efficiently translate them [9]. Other notable examples include AbsB, an RNase III homolog [10] and Clp, an ATP-dependent protease family [11] that play key roles in morphogenesis and/or antibiotic biosynthesis. Given the physiological and genetic complexities associated with *Streptomyces*, it is quite conceivable that numerous other such instances have simply not been elucidated yet. A major challenge in investigation of gene regulation in such organisms is to delineate the extent to which changes in proteins abundances are brought about by transcriptional or post-transcriptional regulatory means.

In the current work, we employed a shotgun proteomics approach combined with isobaric tagging for relative and absolute protein quantitation (iTRAQ™) [12] for a quantitative assessment of the proteome dynamics in *S. coelicolor* M145. Taking advantage of the multiplexing capability of the iTRAQ™ system, we constructed time-series profiles representing protein dynamics through different growth stages in liquid culture and compared the results with microarray-derived transcriptome data. We then simplified the data using principal component analysis to evaluate the overall degree of concordance between mRNA and protein levels and to identify individual instances of significant discordant behavior. Finally, this data was mapped onto a metabolic reaction network to evaluate correlations amongst functionally related genes and interpret the biological significance of such dynamics.

## Results

### Growth kinetics and experimental setup

**Figure 1:**
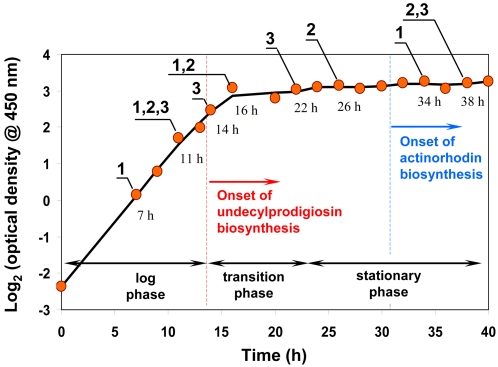
Growth-time curve of *S. coelicolor* in R5^−^ complex media. Samples analyzed by MS runs 1, 2 and 3 are indicated.

To examine the changes in proteome profiles associated with growth and adaptation in *S. coelicolor* M145 cells, we isolated total cell proteins from eight temporally spaced samples (7 h, 11 h, 14 h, 16 h, 22 h, 26 h, 34 h and 38 h) as shown in Figure 1. The samples chosen reflect changes in cellular physiology associated with growth and transition to stationary phase as well as the conspicuous onsets of two prominent antibiotics, undecylprodigiosin and actinorhodin. Since the iTRAQ™ system used in this study can analyze only four distinct samples in a single experiment, we chose to distribute the eight protein samples to three runs of mass spectrometric analysis (Figure 1). The experiments were also designed so as to enable validation of the methodology by comparison of two protein ratios (16 h/11 h and 38 h/11 h) estimated from independent replicate runs.

### Assessment of protein identification accuracy and quantification reproducibility

Protein identifications and quantifications were carried out by searching the raw spectral data (*.wiff files) against a theoretical proteome of *S. coelicolor* using ProteinPilot™ software and inbuilt Paragon™ search engine [13]. Decisions concerning the inclusion of single peptide (particularly single spectral evidence) hits and peptide confidence score cutoffs will greatly influence the final number of proteins one can report. A heuristic means to arrive at these decisions is by estimating the false positive identification rates by performing a search against a randomized decoy database. Table 1 summarizes the results of such searches at various confidence levels using data from all three experimental runs. At 99% confidence level, single peptide hits incur only 3.9% false identification rate (i.e. the fraction of all single peptide hits ( =  1100-680) that could be ‘false’ based on decoy database search ( =  18-2)). For single spectral evidence hits, a similar calculation leads to only 4.9% false identification rate. On the other hand, the 81 ( =  1181-1100) additional proteins identified by relaxing the confidence cutoff from 99% to 95% will likely include 21 ( =  39-18) false hits giving rise to ∼26% false identification rate. Consequently, only the 1100 proteins identified with ≥99% confidence were considered for further analysis. Biological interpretations from single peptide hits were, however, made only when additional evidences such as similar dynamic profiles from functionally related genes were available. This set of 1100 proteins corresponds to approximately 14% of the theoretical predicted proteome of *S. coelicolor*. 330 of these proteins were identified in all three runs thus providing an eight time-point protein profile.

**Table 1:** Summary of database search results.

	All hits^a^		≥2 MS/MS spectra^b^		≥2 peptide hits^c^	
ID confidence score^d^	≥95%	≥99%	≥95%	≥99%	≥95%	≥99%
*S. coelicolor* database search^e^	1181	1100	851	795	728	680
Decoy database search^f^	39	18	10	3	6	2
**False Identification Rate**	*3.30%*	*1.64%*	*1.18%*	*0.38%*	*0.82%*	*0.29%*

a all protein identifications.

b protein identifications with ≥2 MS/MS spectral evidences.

c protein identifications with ≥2 unique peptide hits.

d confidence score as reported by ProteinPilot™.

e database downloaded from ftp://ftp.sanger.ac.uk/pub/S_coelicolor/whole_genome/Sco.prot_fas; a list of common contaminants were included in the search but contaminant protein hits are not reported here.

f database created by randomizing amino acids sequence; this database resembles *S. coelicolor* database in terms of number of entries, sequence lengths and amino acid composition.

To assess the degree of technical variance in quantification of relative protein abundance levels, we calculated the coefficient of variation (*CV*) based on log mean average of protein ratios estimated from independent experimental runs. The 16 h to 11 h quantification ratio calculated for 420 common proteins identified in runs 1 and 2 resulted in a median *CV* of 0.081 while the 38 h to 11 h ratios estimated for 382 proteins identified in both runs 2 and 3 gave a median *CV* of 0.138. These values are comparable with those previously reported in literature for iTRAQ™ experiments [14]. A small fraction (3.1% and 6.8% respectively in the two comparisons) yielded a relatively high *CV* (>0.5) and as such, interpretation of protein profiles in these cases will require considerable circumspection. Nevertheless, consistency across technical replicates in these isobaric tagging LC-MS/MS experiments were at least comparable to, if not better than, those of 2D gel electrophoresis methods (median *CV*s recalculated in linear scale for the two duplicates were 12% and 21%; compare with 20–30% variability reported for 2D gel electrophoresis [15]).

To assess biological variability, we compared the stationary phase to log phase protein expression ratio between independent biological replicate cultures. The *CV* based on log mean ratios for this comparison was found to be 0.15 and a scatterplot showing this comparison is shown in supplemental data (Figure S1).

### A synopsis of proteins identified and their abundance

Proteins identified from the 2D LC-MS/MS experiments were categorized according to a classification scheme based on their primary annotation. Proteins from all functional categories except laterally acquired elements were identified in our analysis (Table 2). Classes of proteins that were highly represented included those that belong to nucleotide biosynthesis and ribosomal constituent families (where over three-fourths of the total annotated proteins were identified) and to a lesser extent, amino acid biosynthesis and central intermediary and energy metabolism families. Unsurprisingly, only 5% of the regulatory proteins were found, perhaps indicating the relatively low expression levels sufficient for their functional efficiency or the more likely possibility of these genes not being expressed under the culture conditions tested. Analysis of chromosomal organization of these proteins revealed that approximately 80% (872 of 1100) were located in the 4.9 Mb central core (containing only 56% of all genes) reinforcing the belief that the chromosome arms are largely comprised of auxiliary genes [8].

**Table 2:** Functional classification of proteins identified.

Function^a^	Total Present	Number Identified	Percent identified
Cell processes	800	108	14%
Macromolecule metabolism	496	130	26%
Amino acids biosynthesis	123	53	43%
Nucleotide biosynthesis	30	24	80%
Ribosomal constituents	67	51	76%
Biosynthesis of cofactors and carriers	118	38	32%
Central intermediary metabolisms	111	44	40%
Degradation of small molecules	200	37	19%
Energy metabolism	189	74	39%
Fatty acid and phosphatidic acid biosynthesis	56	15	27%
Secondary metabolism	277	63	23%
Periplasmic, exported or lipoproteins	1318	106	8%
Two-component systems	165	15	9%
RNA polymerase core enzyme binding	88	7	8%
Regulatory proteins	673	36	5%
Protein kinases	39	6	15%
Laterally acquired elements	139	0	0%
Not classified	565	76	13%
Hypothetical proteins	2371	217	9%
**Total**	**7825**	**1100**	**14.1%**

a protein classification scheme derived from EcoCyc database; downloaded from http://www.sanger.ac.uk/Projects/S_coelicolor/scheme.shtml.

**Figure 2:**
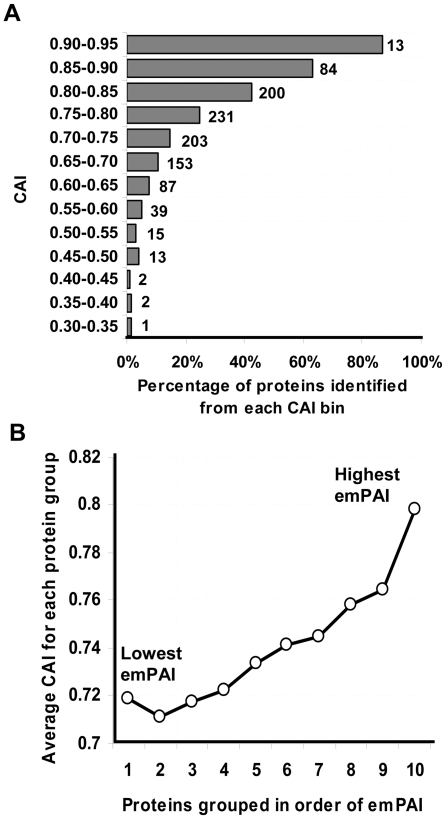
Relationship between CAI and protein identification or abundance. (A) Percentage of proteins identified in various CAI ranges. Values beside the bars represent the absolute number of proteins identified from each bin (B) Average CAI of identified proteins in different abundance groups; values in horizontal axis correspond to protein abundance, “10” indicating the top 10 percentile (most abundant) to “1” indicating the bottom 10 percentile (least abundant) proteins. Protein abundance was estimated from MS data using emPAI based on number of MS/MS spectral evidences (refer to Materials and Methods)

Several previous studies including one in *S. coelicolor* [16] have considered optimal codon usage in genes as a reliable indicator for enhanced protein expression. To examine if this is observed in our dataset, we calculated a Codon Adaptation Index (CAI, [17]) for every gene in *S. coelicolor* based on ribosomal genes as the reference set. CAI values range from 0 to 1 with higher values indicating optimal codon usage (i.e. codon usage trend similar to ribosomal genes in this case). Figure 2A shows the distribution of proteins identified from different CAI groups. Proteins in high CAI groups were significantly better represented in our identifications. In addition, even amongst those proteins identified, there is a definite positive correlation between CAI and exponentially modified protein abundance index (emPAI), a measure of protein abundance derived from the total number of spectral evidences contributing to a given protein identification [18] (Figure 2B). Also, based on emPAI calculations, some of the most abundant proteins in the cell were found to be the chaperones (GroES/EL1/EL2; SCO4296, 4761–62), elongation factor Tu-1 (SCO4662), a putative tellurium resistance protein (SCO4277) and a type I polyketide cluster reductase (SCO6282).

### Comparison of proteome and transcriptome data – correlations in average abundance

Changes in the global proteome associated with liquid culture growth of *S. coelicolor* were examined using relative protein ratios calculated from MS/MS signals of the isobaric tags. Since a common reference allows comparison of expression ratios from all samples in a single profile for each protein, data from all three experimental runs were combined to yield fold-change ratios with respect to 7 h sample. Consequently, only 894 proteins identified in run 1 (which includes the 7 h sample) could be used for this analysis.

To assess the extent to which observations from microarray analysis correlate with those from mass spectrometry, we isolated total RNA from the same eight samples shown in Figure 1 and analyzed them using spotted DNA microarrays described previously [19]. The microarray was composed of PCR amplified DNA probes printed in duplicate for 7578 different ORFs in the *S. coelicolor* genome which corresponds to approximately 97% of the predicted genes in this organism.

**Figure 3:**
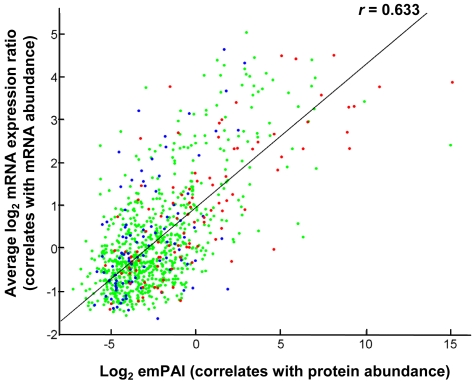
Correlation between mRNA and protein abundance. A plot of mRNA abundance calculated as [mRNA/gDNA] from microarray experiments against protein abundance calculated as emPAI based on number of MS/MS spectral evidences is shown in log_2_ scale. The top ten percentile (highest) CAI ranked genes (blue circles), bottom ten percentile CAI ranked genes (red circles) and all other genes (green circles) are shown. For the sake of clarity, five data points with exceptionally high protein abundance (log_2_[emPAI]>20) are not shown here. All data points were, however, included in the Pearson's correlation coefficient (*r*) calculation.

Since hybridizations were performed with genomic DNA as reference, the resulting mRNA/gDNA ratio (reported here in logarithmic scale and referred to as log_2_ mRNA abundance ratio) is an indicator of relative mRNA abundance in the cell. A scatter plot of average log_2_ mRNA abundance ratio versus emPAI estimated using total number of MS/MS spectra identified from all three proteomic runs revealed a moderate correlation between mRNA and protein abundance (Figure 3). The Pearson's correlation coefficient (*r*) for a straight line fit for this data was a modest 0.63. Interestingly, this correlation improved when only the top 50 high ranking genes in terms of CAI values (optimal codon containing genes) were considered (*r*  =  0.8). For low ranking genes (those with 50 lowest CAI values among proteins identified), this correlation dropped to *r*  =  0.35. One possible reason for this observation could be that low CAI ranking proteins are generally less abundant and emPAI correlates rather poorly with protein abundance in such cases (since emPAI values tend to be discrete when peptide counts are low).

### Comparison of transcriptome and proteome data – correlations in dynamics of expression

**Figure 4:**
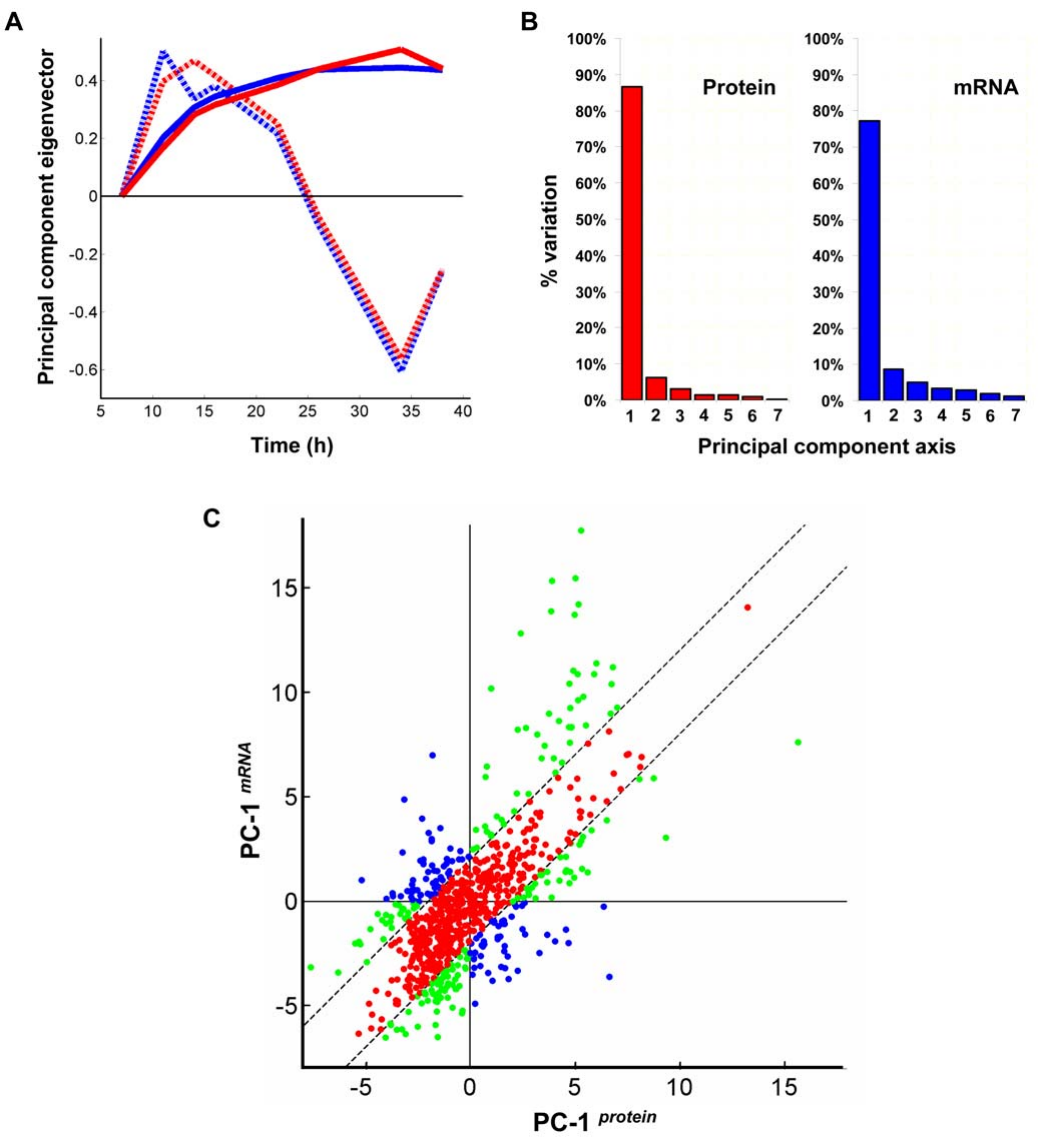
Principal component analysis of transcriptome and proteome data. (A) ‘Loadings’ (eigenvector) plot for the first two principal component axes – PC-1 (solid lines) and PC-2 (dashed lines) from proteome (red lines) and transcriptome (blue lines) data (B) Percentage of variation accounted for by each of the seven principal components in proteome and transcriptome data (C) Values of genes along PC-1*^protein^* plotted against PC-1*^mRNA^*. Green and blue dots represent genes with significantly large difference in expression trends (|PC-1*^protein^*−PC-1*^mRNA^*|≥2). Of these, blue dots indicate those which are likely to exhibit opposing trends (2*^nd^* and 4*^th^* quadrants). All other genes are shown as red dots.

We sought to identify the major trends in both mRNA and protein dynamics using Principal Component Analysis (PCA). Of the 894 genes selected earlier, we chose to analyze only 798 gene products for which quantification ratios from at least four out of eight time points could be determined from both microarray and proteomic experiments. Missing values were estimated by linear interpolation as before. Considering the eight time-point log transformed temporal gene expression profiles as data points in an eight-dimensional space, PCA enabled us to transform coordinate axes and identify the most important dimensions in this transformed space (dimensionality reduction thereby simplifying the time-course data). Independent PCA of both mRNA and protein data from the 798 gene products indicated that the first two principal components (PC-1*^mRNA^* and PC-1*^protein^*) account for over 85% of the total variance in the either dataset (Figure 4B). The loadings plots (eigenvectors) for each of these two principal components were remarkably well correlated between mRNA and protein data (Figure 4A) implying that the major trends in both proteome and transcriptome domains were strikingly similar. Furthermore, the principal component 1 represented the tendency of genes to be either up- or down-regulated during stationary phase adaptation. This component alone accounted for over 75% of the total variance in either dataset, and therefore, appears to be the most prominent pattern in our data. The other major component, PC-2, which accounted for an additional 6–9% variance, represents the tendency of certain genes to be transiently up- or down-regulated primarily during the transition between exponential and stationary phase.

### Biological insights from gene profile analysis

**Figure 5:**
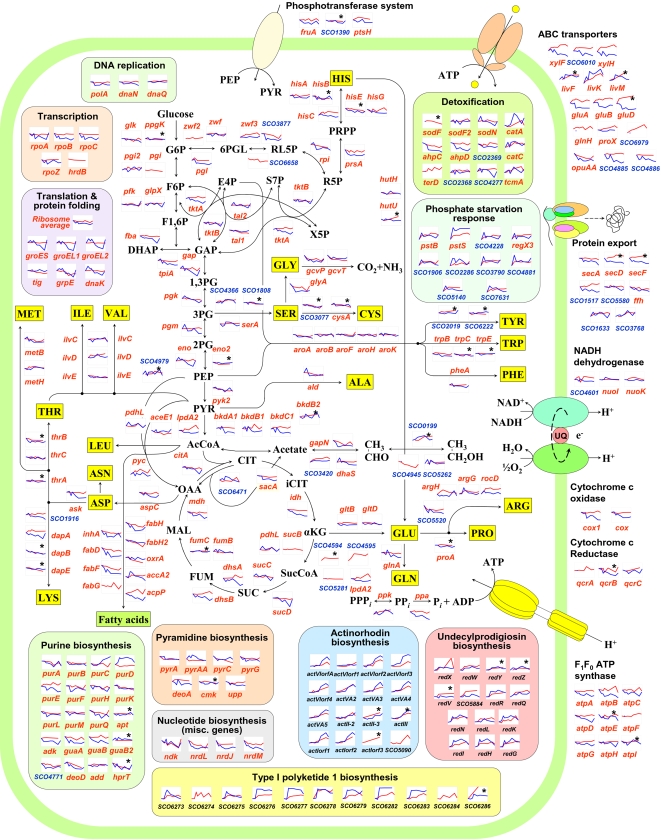
Integrated transcriptome and proteome data mapped onto a simplified metabolic network. Proteome data (red lines) and transcriptome data (blue lines) are plotted in the same graph. The horizontal axis of each plot represents time ranging from 7 h to 38 h. The fold-change ratios of genes in the vertical axis span a value of 6 in log_2_ scale, except for actinorhodin, undecylprodigiosin, type I polyketide biosynthesis and phosphate starvation response genes where the vertical axis spans a value of 8 in log_2_ scale. Each plot begins with log ratio  =  0 at 7 h. Single peptide protein identifications are indicated with an asterisk (⋆), and their MS/MS spectra are provided Figure S4. Pathways and corresponding enzymes were identified from KEGG database [34]. Only major reactions in each pathway are shown. Gene names and SCO numbers are provided in Tables S2 and S3.

Genes in major functional classes were examined for their correlations with the principal eigen-genes and eigen-proteins shown in Figure 4A. In the following discussion, presence of a gene in the top or bottom 10 percentile of PC-1, PC-2 values are considered as indications for positive or negative correlation with that particular eigen-gene or protein. To facilitate identification of patterns among functional classes in a metabolic context, the transcriptomic and proteomic data were also plotted on a simplified metabolic reaction network using gene annotations taken from KEGG pathway database (Figure 5).

#### Energy metabolism

Enzymes for every step in the core primary metabolic pathways of glycolysis, TCA cycle and pentose phosphate reactions were detected in our proteomic analysis. The expression levels of these proteins were generally stable or exhibited a weak negative correlation with PC-1 of proteins. At the mRNA level, a similar observation was observed for most proteins. However, mRNA encoding enzymes leading from glyceraldehyde 3-phosphate to pyruvate (*gap*, *pgk*, *pgm* and *eno*) were moderately correlated with PC-2 indicating a transient increase and subsequent decrease in expression. Among oxidative phosphorylation genes, *cox1* and *qcrB* and ATP synthase subunit *atpB* were strongly negatively correlated with PC-1 at the mRNA level. The corresponding proteins, however, had no clear correlations indicating a relatively flat profile. One putative cytochrome P450 gene (SCO2884) was highly correlated with PC-1 at the protein level (upto 8-fold upregulation) and to a lesser extent at the mRNA level.

#### Secondary metabolism

Biosynthetic enzymes from all four major secondary metabolite clusters (actinorhodin, 16/22 genes; undecylprodigiosin, 15/22 genes; a cryptic type I polyketide, 11/16 genes and calcium dependent antibiotic, 8/40 genes) and two other minor ones (deoxysugar synthase and coelichelin) were detected in our analysis (bottom right panels in Figure 5). Most of these genes were very strongly correlated with PC-1 at both the mRNA and protein levels. In addition, most of the actinorhodin cluster genes displayed a strong negative correlation with PC-2, primarily due to the fact that these genes are up-regulated only at very late stages of the culture. Also, mRNA of these genes appeared to be more dynamic with up to 60-fold changes while proteins levels changed by less than 20-fold.

#### Other metabolisms

Numerous amino acid metabolism proteins and transporters displayed dynamic profiles possibly hinting at extensive reorganizations in the intracellular amino acid pool to cope with the nutritional imbalances in stationary phase. Notable among them were a family of branched chain amino acid transporters (SCO2008, 2010–12, LivMKGF) which were positively correlated with PC-1 of both proteins and mRNA. Genes involved in arginine-glutamate conversion like RocD (SCO1223) and ArgH (SCO1570) displayed a prominent correlation with PC-1 only at the protein level. In contrast, a glycine cleavage system (SCO1378/5472, GcvP/T; glycine degradation), a serine hydroxymethyltransferase (SCO5470, GlyA; serine-glycine interconversion) and a related glycine betaine transporter (SCO1621, OpuAA) all of which are required for generating one-carbon moieties to be used in several cellular processes like methyl transfer reactions and purine biosynthesis were negatively correlated with PC-1 (two to five-fold down-regulation). In addition, ribonucleotide reductases like NrdJ/M involved in *de novo* DNA synthesis also displayed a strong negative correlation with PC-1 of proteins and mRNA, presumably due to reduced cellular demands.

#### Nucleic acids and protein synthesis

DNA polymerase subunits displayed a generally invariant trend while RNA polymerase subunits were negatively correlated to PC-1 prominently at the mRNA level. Among protein synthesis machinery, approximately half of the ribosomal protein sub-units were negatively correlated with PC-1 at the mRNA level while much fewer proteins displayed such a trend at the protein level.

#### Stress response genes

Catalases CatA/C (SCO0379/0560) were showed a strong positive correlation with PC-1 while alkyl hydroperoxide reductases (AhpCD, SCO5032/31) displayed a negative correlation with PC-1 at mRNA level. The corresponding proteins with the exception of SCO5032 did not display such strong correlations. Numerous known and putative phosphate starvation response genes (PstB, SCO4139; PstS, SCO4142; NeuB, SCO4881; RegX3, SCO4230; a phosphate transporter, SCO4228; alkaline phosphatase, SCO2286; SCO1906; SCO3790; SCO5140 and SCO7631) were strongly correlated with PC-1 of both proteins and mRNA. These genes also generally had a high positive value along PC-2, indicating a transient up-regulatory trend.

### Identification of genes with differential mRNA and protein dynamics

Identification of genes exhibiting differences in mRNA and protein dynamics might have important biological implications as they indicate differences in synthesis rates and stability of proteins and mRNA as well as possible post-transcriptional regulation. This was performed by comparing the values of genes along the major principal component axis (PC-1) in mRNA and protein dynamics data. Figure 4C depicts a plot of PC-1*^mRNA^* against PC-1*^protein^*. In general, the closer a gene is to the *y*  =  *x* line, the greater is the degree of concordance between mRNA and protein trends (i.e. PC-1*^mRNA^*≈PC-1*^protein^*). In contrast, genes in the top-left or bottom-right quadrants generally tend to exhibit opposing trends. One caveat with this two-dimensional analysis, however, is that the effects of PC-2 and higher principal components are neglected. Hence, this analysis should only be considered as a first approximation for comparative profile analysis that, nevertheless, will require confirmation by visual inspection.

Based on this analysis, a total of 289 gene products (36% of analyzed mRNA-protein pairs) displayed a difference of two or more between the values of PC-1 in mRNA and protein domains (|PC-1*^mRNA^*−PC-1*^protein^*|≥2). Of these, 107 (13%) were likely to exhibit varying degrees of opposing trends (Figure 4C) with 57 genes in top-left quadrant i.e. mRNA up-regulated while proteins are down-regulated, and 50 genes in bottom-right quadrant i.e. mRNA down-regulated while proteins are up-regulated.

### Functionally related genes exhibit similar discordant behavior

**Figure 6:**
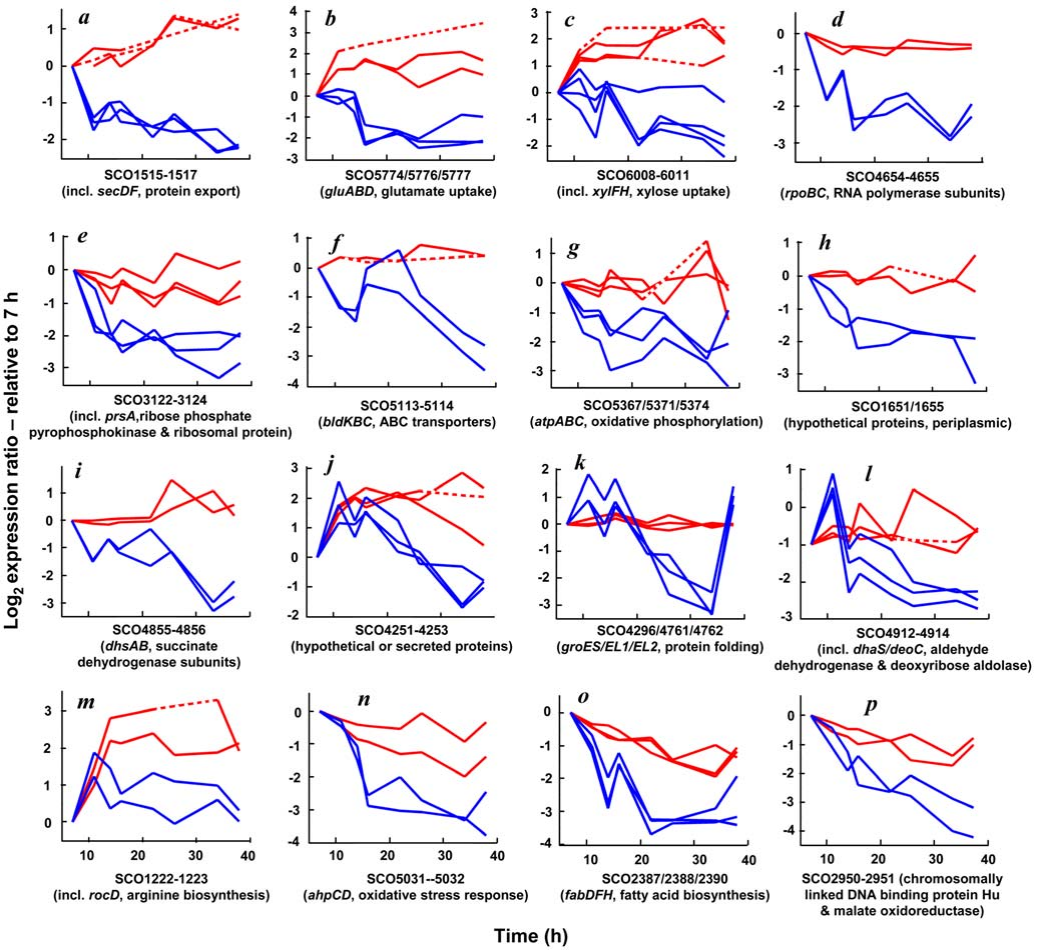
Examples of related genes displaying similar behavior. Examples of functionally or chromosomally clustered genes exhibiting discordant mRNA and protein dynamics trends as identified using PCA. Dashed lines indicate regions where data interpolation was performed for PCA calculations.

Integrating transcriptome and proteome data with available functional information yields valuable biological insights. A careful examination of Figure 5 reveals that several mRNA and proteins are regulated at a pathway or sub-pathway level. It is evident that, in a number of cases, even when dissonant trends are observed between mRNA and protein profiles (discovered using PCA), such behaviors are usually conserved across at least a few functionally related and/or chromosomally linked genes. Figure 6 shows some interesting profiles that fall in this category. Surprisingly certain groups of genes exhibited not just discordant, but contrasting mRNA-protein profiles. These include a putative protein secretion system (SCO1515–1517; Figure 6A), a glutamate uptake system (GluABCD, SCO5774–77; Figure 6B) and a xylose uptake system (SCO6008–6011; Figure 6C). In each case, the proteins exhibited a two- to eight-fold increase in abundance upon entry into stationary phase while the corresponding transcripts displayed a concomitant two- to four-fold down-regulation. Another set of genes is comprised of those for which the protein levels remained relatively constant while mRNA amounts decreased rapidly in stationary phase, hinting at lower rates of protein turnover than mRNA. These include RNA polymerase subunits (RpoBC, SCO4654/55; Figure 6D), phosphoribosyl transferases/ribosomal proteins (SCO3122–24; Figure 6E), a set of ABC transporters (BkdBC, SCO5113/14; Figure 6F), ATP synthase subunits (AtpABC, SCO5367/71/74; Figure 6G), a set of chromosomally linked hypothetical proteins (SCO1651/1655, Figure 6H) and succinate dehydrogenase subunits (DhsAB, SCO4855/56; Figure 6I). Several other distinct profiles were also observed including cases where mRNA was transiently up-regulated but subsequently down-regulated while protein levels remained constant (Figure 6K and 6L) or displayed an increase during transition to stationary phase (Figure 6J). However, the most common type of divergent behavior was differing degrees of differential patterns in largely the same direction. Four such examples are shown in Figure 6 – a set of arginine biosynthesis genes (SCO1222–23; Figure 6M; up-regulated), oxidative stress response genes (AhpCD, SCO5031–32; Figure 6N; down-regulated), fatty acid biosynthesis genes (FabDHF, SCO2387/88/90; Figure 6O; down-regulated) and two other chromosomally linked proteins (DNA-binding protein Hu and malate oxidoreductase, SCO2950/51; Figure 6P).

It is important to note that our observation of similar dynamic trends among functionally related genes lends significant biological confidence to protein quantification even in the absence of strict statistical confidence. Numerous instances of genes that could not grouped in functional clusters also exhibited divergent behavior. The complete set of genes identified from Figure 4C is presented in Table S1. A subset of these genes with substantial profile differences are plotted and shown in Supplemental Data (Figure S3). For the sake of a balanced perspective, some genes with remarkable concordance between mRNA and protein profiles are also shown alongside (Figure S2).

## Discussion

To analyze dynamic biological systems, one often needs to extract quantitative information from multiple time-point samples. A mass spectrometry based approach using iTRAQ labeled peptides is well suited for such analyses as it allows simultaneous analysis of multiple samples. Using this strategy, we identified 1100 *S. coelicolor* proteins with less than 2% false identification rate and quantification variances comparable to, if not better than, other proteomic approaches. Over half of the proteins identified in this study were previously undetected implying that our study significantly enhances the repertoire of experimentally validated ORFs in *S. coelicolor*. Consistent with previous reports on correlations between codon usage and mRNA expression [20], we observed that genes with a higher CAI (‘optimal’ codon containing genes) were more likely to be detected in our analysis.

The major goal of this study was to analyze the extent of correlation between mRNA and protein expression. A direct comparison of average mRNA and protein abundance measures shown in Figure 3 did not reveal a strong correlation. This seeming lack of correlation could be attributed to the absence of a reliable continuous metric for measuring protein abundances from mass spectrometry approaches employed here. Nevertheless, this correlation appeared to be substantially higher for genes with a stronger bias toward ‘optimal’ codons (Figure 3) raising a possibility that optimal codon containing genes are primarily regulated at the transcriptional level. This would imply that high mRNA abundance gives rise to high protein abundance and vice versa for such genes. In contrast, genes with poor codon usage may have additional post-transcriptional regulatory roadblocks and consequently exhibit a relatively poor correlation. Some recent investigations on prokaryotic translation [21], [22] also point to the relative importance of codon usage over other factors like translation initiation/termination sequences in determining mRNA-protein correlation. This evidence, taken together with the fact that *Streptomyces* display an unusually high G+C bias and generally do not require a highly conserved ribosome binding (Shine-Dalgarno) sequence for translation initiation [23], might suggest that polypeptide chain elongation during translation plays a crucial role in dictating protein expression. It is also noteworthy that, despite using a different metric for estimation of mRNA and protein abundance, a similar trend was reported earlier for a compilation of yeast transcriptome and proteome data [24].

Correlations between transcriptome and proteome data can also be evaluated at the level of the dynamics of mRNA/protein expression. Several previous studies in yeast [1]–[6] have reported widely varying, but nonetheless poor to moderate correlations between mRNA and protein abundance ratios (Spearman rank correlations ranging from 0.21 to 0.74). When similar protein and mRNA ratios from all the samples in our data pooled together were plotted together (graph not shown), we observed a relatively weak correlation of 0.55. Although this suggests, at best, a rather moderate correlation between mRNA and protein data, it fails to address one important aspect of our data, namely, its time-series nature. The introduction of time as an additional data dimension means that gene expressions can now be viewed as part of a continuing trend in the process of stationary phase adaptation instead of absolute static two-point relative fold-change ratios per se. The complexity in time-series data could then be reduced using PCA to facilitate mRNA-protein comparison. Consistent with our observations, a recent study in *S. coelicolor* using defined medium [25] had concluded that the major principal components in mRNA and protein domains are similar. However, unlike previous studies, in our approach, we extracted mRNA and proteins from the same set of samples for comparative analysis. This allowed direct gene-by-gene comparisons with much higher confidence disregarding culture to culture variations and thereby identification of discordant behaviors among individual genes.

The discordance between transcriptome and proteome data observed here can be attributed to two important factors – differences in translation efficiency that depend on codon usage and ribosome binding sites among other factors between different genes and diversity in degradation rates among different genes and also between mRNA and proteins. The latter is, probably, a crucial factor because we have seen that while mRNA levels decrease sharply for many genes in stationary phase, the protein levels displayed only a modest decrease or sometimes no change at all. The stringent response induced in *Streptomyces* and many other bacteria through increased levels of ppGpp during stationary phase is a phenomenon that causes reduced transcription of several growth related genes [26]. Redistribution of the RNA polymerase caused by decreased levels of the primary vegetative sigma factor and increased activity of alternative sigma factors is thought to be the reason for this phenomenon. Consistent with this theory, we observed that the levels of HrdB protein (the primary sigma factor in *S. coelicolor*) decreased by ∼35% between 7 h and 34 h in culture. While this caused decreased levels of several transcripts belonging to primary metabolism and DNA, RNA and protein synthesis machineries, it is conceivable from the observations that the corresponding proteins, unlike mRNA, are more stable because they show only a modest change in abundance. In addition to these effects, different proteins may have different degradation rates. The N-terminal amino acid of proteins has been proposed to play a significant role in dictating its stability [27]. *S. coelicolor* is known to carry out N-terminal processing of proteins in certain cases [26] thereby introducing N-end heterogeneity which might significantly contribute to variations in protein stability. Although we did not find correlations linking the N-terminal amino acid with mRNA-protein divergence, we found that proteins displaying concordance with mRNA (|PC-1*^mRNA^*−PC-1*^protein^*|<1) contained larger fractions of lysine, a probable destabilizing amino acid in prokaryotes compared to proteins that were less dynamic than mRNA (PC-1*^mRNA^*−PC-1*^protein^*>2) (4.6% compared to 3.9% of lysine abundance with p-value  =  0.07). This is consistent with earlier reports in yeast suggesting that the protein-wide amino acid composition may correlate with protein degradation [28]. We also explored the possibility of mRNA-protein divergence arising from known post-transcriptional regulatory mechanisms like those involving the rare leucine codon – TTA [9]. Five genes encoding TTA codon were identified and examined in our study. Three of those including an actinorhodin transporter (SCO5083) displayed consistent mRNA and protein dynamics as defined by the criteria shown in Figure 4C. However, the other two genes – a regulatory protein AdpA (SCO2792) involved in morphological differentiation [29] and a protein of unknown function (SCO6638) – displayed increasing protein trends although the corresponding mRNA levels were relatively constant. This indicates a possible role for growth dependent control of these genes by *bldA*, the only *S. coelicolor* tRNA known to translate the rare TTA codon.

To an extent experimental noises could also account for the apparent discrepancy between mRNA and protein. Accurate identification of a protein as described earlier does not necessarily guarantee an accurate quantification. Numerous factors like peptide concentration and amino acid composition can influence the quality of spectra and hence the apparent quantification ratios obtained from iTRAQ experiments. Error factor and *p*-value estimates presented in the supporting information provide a means of assessing consistency between peptides within the same protein. Given these technical limitations, discerning the actual biological effects from experimental noises is challenging. In this work, the time-series nature of the data provided an opportunity for estimating the fidelity of quantification through detection and further scrutiny of outlier points from an otherwise consistent trend (like monotonic increase, for example). Also, most importantly, consistency between functionally related genes significantly enhanced our confidence in the observations, especially in cases of mRNA-protein discordance.

Our analysis reveals that many of the molecular machineries causing divergent mRNA-protein behavior act at a protein complex or biological pathway level. Similar profiles among groups of related mRNA in bacteria frequently arise because of their organization into single transcription units (nearly 30% of the genes listed in Table S1 could be classified as belonging to operons [30]). Many of the corresponding proteins also displayed similarities in expression profiles indicating likely similarities in ribosomal occupancies, translation efficiencies and degradation rates as well. The surprising discovery presented here was that such co-expression and probable co-regulation of related mRNA and proteins was observed even in cases with divergent mRNA-protein dynamics.

In summary, we have shown that a mutually complementary analysis of transcriptome and proteome data enables one to better understand the dynamics of biological systems. While the importance of transcriptional regulation is quite evident from the unusually large number of genes encoding transcriptional regulators in *S. coelicolor* [8], we show here that post-transcriptional regulation may be an equally important facet of the intricate molecular adaptation machinery employed by this fascinating microbe.

## Materials and Methods

### Bacterial strains and culture conditions

*S. coelicolor* M145 spores were generated in Mannitol-Soy flour agar plates [31]. Liquid cultures were performed in shaker flasks (220 rpm; 30°C) in R5^−^ medium (R5 medium devoid of additional K_2_HPO_4_, CaCl_2_ and L-proline) as described earlier [32]. Briefly, ∼2.5×10^9^ spores were first pregerminated in 2×YT medium for 8 h. Germinated spores were then harvested, dispersed by brief sonication and inoculated into 250 ml of R5^−^ medium with 0.05% (v/v) antifoam 289 in siliconized conical flasks with stainless steel coiled springs. Cell growth was monitored by measuring optical density at 450 nm of dispersed (sonicated) mycelia. Samples were harvested periodically for transcriptome and proteome analysis and chilled rapidly in dry ice/ethanol bath before centrifugation. Cell pellets were stored at −80°C until further analysis.

### Cell lysis and sample preparation

Cell lysis was kept as simple as possible to avoid introduction of compounds that might potentially interfere with mass spectrometry. Accordingly, frozen cell pellets were pulverized by grinding in liquid nitrogen and cellular contents were solubilized in 50 µl of lysis buffer (8 M urea, 4% CHAPS) supplemented with 4 mM phenylmethylsulfonyl fluoride. The volumes were brought up to 400 µl each with dissolution buffer (0.5 M triethylammonium bicarbonate, pH 8) and protein assays were then carried out using Commassie Plus® Bradford assay (Pierce Research Instruments, Singapore). Aliquots of 100 µg proteins from each sample were processed for labeling with iTRAQ according to manufacturer's instructions (Applied Biosystems, Foster City, CA). The labeled samples were mixed and concentrated in a SpeedVac to reduce volatile content, before diluting 10× with cation exchange loading buffer (10 mM KH2PO4, 25% acetonitrile, pH 3).

### Strong cation exchange fractionation

Injection of the sample was performed in multiple aliquots onto a strong cation exchange column (PolyLC 2 mm×150 mm, Nestgroup, Southborough, MA). Separation of peptides was performed by developing a 2-step gradient of KCl – from 0% to 20% salt buffer (10 mM KH2PO4, 20% acetonitrile, 500 mM KCl, pH 3) over 40 min, followed by an increase to 100% salt buffer over 20 min, at a flow rate of 200 µL/min with fractions collected every 1.5 min. Fractions were desalted using C-18 spin columns (Vivapure®, Sartorius, Singapore) and eluted in 70% acetonitrile, 0.25% formic acid. Acetonitrile in the eluant was eliminated by SpeedVac and each fraction was reconstituted with 1% formic acid, 2% methanol in Milli-Q water for mass spectrometric analysis.

### Mass spectrometry

NanoLC- mass spectrometry (MS/MS) was performed using a QSTAR-XL hybrid quadrupole-time of flight tandem mass spectrometer (Applied Biosystems) coupled to an LC-Packing (Sunnyvale, CA) system comprising of a FAMOS autoinjector unit, a SWITCHOS 10 port valve unit, and an ULTIMATEPLUS nano-flow pumping unit. An injection volume of 10 µl from each sample was made onto a reversed-phase C-18 peptide trapping cartridge (300 µm×5 mm, LC-Packings) in a flow of 0.1% formic acid for 5 min at 25 µl/min. Following the wash step the flow from the pumping unit was diverted back through the trapping cartridge at 100 nl/min. Peptides were eluted from the cartridge by application of a gradient from 0 to 90% acetonitrile in 0.1% formic acid over 40 min at 100 nl/min, and separated by passing through a C-18 reversed phase column (packed in-house with 5 µm particle size packing material from Column Engineering, Ontario, CA). Peptides eluting from the column were sprayed directly into the orifice of the mass spectrometer, which was run in IDA (information dependent acquisition) mode selecting all 2+ to 4+ charged ions with signal intensity greater than eight counts per second over the specified mass range. For experimental runs 1 and 3, low protein-content fractions were run once and scanned in the m/z range 350–1100 amu, while high protein-content fractions were run thrice scanning m/z ranges of 350–600 amu, 598–800 amu, and 798–1100 amu each time. For run 2, all fractions were run first scanning an m/z range of 350–1100 amu, while certain high protein-content fractions were reinjected for analysis with an exclusion list derived from previously identified peptides. For collision-induced dissociation, nitrogen gas was used at a setting of four and the collision energy set to automatic allowing increased energy with increasing ion mass.

### Protein identification, quantification and data processing

Protein identifications were carried out by searching the raw data files (*.wiff) against a predicted *S. coelicolor* proteins database (supplemented with some common contaminant protein sequences) using ProteinPilot™ software v1.0. The predicted *S. coelicolor* protein sequences containing 7810 entries were downloaded from ftp://ftp.sanger.ac.uk/pub/S_coelicolor/whole_genome/Sco.prot_fas and appended to a list of 179 common contaminant sequences provided with TurboSequest v.27 rev 12/BioWorks 3.1 (Thermo Electron, Waltham, MA). Trypsin specificity was chosen and default options were used all other parameters including the commonly observed oxidized methionine and deamidation of asparagine being considered as possible variable modifications and N-terminal/lysine/tyrosine labeling of iTRAQ reagents as fixed modification. MMTS was chosen as cysteine alkylation modification. The instrument type was set to QSTAR ESI. Unlike many other search engines, ProteinPilot™ does not use fixed mass tolerances but rather employs feature probabilities and a concept called “Sequence Temperature Value” [13]. However, for a comparison with conventional algorithms, we note that over 95% of the peptides scored in our analysis had precursor delta masses less than 0.16 Da. Peptide and protein summary results were exported to Microsoft Excel® 2003 for further analysis.

In estimating quantification ratios, peptides where the total ion counts of iTRAQ peaks were below a threshold of 40 were excluded. The default bias-correction factors from ProteinPilot™ (based on median ratios) were used for normalization. Sample 11 h was analyzed in all three experimental runs conducted in this study. Therefore, protein abundance ratios *r^i^_j/11h,k_* were obtained from experimental run *k* for each protein *i* in sample *j* with respect to the 11 h reference sample. ProteinPilot™ also reports *p*-values and 95% confidence intervals for each protein ratio based on quantification assessments from multiple peptides when available. For those sample pairs that were analyzed in two MS runs, the relative average abundance ratio (*r^i^_j/11h_*), *p*-values and 95% confidence intervals were recalculated from individual bias-corrected peptide ratios and percentage errors of the combined peptide dataset using the algorithm described in ProteinPilot™ user's manual. This step was carried out using Matlab 7.0. Finally, all values were processed to yield protein abundance ratios with respect to the first time-point sample (i.e. 7 h) using
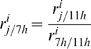
(1)


The complete dataset containing protein identifications and expression data are provided Table S2.

### RNA extraction and microarray analysis

Culture samples harvested for RNA extraction were mixed with one-fifth volume of a “stop solution” (5% phenol in ethanol, [33]) to preserve intact RNA before rapid chilling, centrifugation and storage at −80°C. For RNA extraction, cell lysis was performed using liquid nitrogen and cellular contents were resuspended in buffer RLT (RNeasy Mini kit; Qiagen Inc., Valencia, CA) while all further steps were carried out according to manufacturer's instructions. RNA integrity was assessed by gel electrophoresis and quantity/purity estimated by UV absorbance at 260 nm and 280 nm.

Microarrays produced based on chip construction protocols reported earlier [19] were used for transcriptome analysis. Briefly, ∼10 µg total RNA was reverse transcribed using SuperScript™ II RNase H- reverse transcriptase (Invitrogen, Carlsbad, CA) with random hexamer primers and amino-allyl dUTP nucleotides (Ambion, Austin, TX) to yield cDNA. This cDNA and 200 ng sheared gDNA were labeled with Alexa 647 (Invitrogen) and Cy3 (*Label*IT reagents; Mirus Bio Corp., Madison, WI) dyes respectively. Labeled samples were mixed and co-hybridized onto microarrays in the presence of 50% formamide at 50°C for 16 h. Slides were washed after hybridization and scanned with ScanArray 5000 (Perkin Elmer, Wellesley, MA). Each chip contained duplicate spots for every gene providing a means for basic statistical analysis.

Microarray images were analyzed using GenePix software (Axon Instruments, Union city, CA) to obtain raw spot intensity data. Median fluorescence intensities from each spot were used to calculate mRNA quantification ratios as log_2_[cDNA/gDNA]. Data points with standard deviations greater than 0.5 were filtered out and remaining data was normalized using a quantile normalization approach [19]. For comparison with protein profiles, this mRNA abundance data (log_2_[cDNA/gDNA]) from different samples were processed to yield log_2_[cDNA*_i_*/cDNA*_t_*_o_] relative gene levels for each sample *i* with respect to the first sample (*t*_0_  =  7 h). The dataset containing gene expression values and further analyses corresponding to those proteins identified in mass spectrometry are provided in Table S3. The complete microarray data has been deposited in a MIAME compliant manner at Gene Expression Omnibus (http://www.ncbi.nlm.nih.gov/geo/): accession GSE7172.

### Data analysis

For microarray analysis, data points from technical replicate spots falling outside of mean ±1.2 times standard deviation were discarded for every gene. Genes with large absolute standard deviations between replicate spots (≥0.5 in log_2_ scale) were also eliminated from further analysis. In addition, gene profiles generated were compared with profiles from biological replicate cultures for consistency of expression patterns.

Reproducibility of quantitative protein data from independent MS runs was assessed using coefficient of variation. Since this data is likely to resemble a log-normal distribution, the coefficient of variation was calculated as
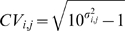
(2)where *σ_i,j_* is the standard deviation of log_10_[*r^i^_j/11h,k = 1,2,3_*] for protein *i* in sample *j* with respect to 11 h using any of the independent runs *k*, where the sample pair *j* and 11 h were analyzed together. Note that log_10_ is used specifically in this case for the sake of comparison with previous reports [14] while log_2_ is used for all other analysis described in this work. The *CV* was also calculated in linear scale as ratio of mean to standard deviation for comparing with similar results from 2D gel based experiments [15].

Protein abundance estimates were obtained from exponentially modified protein abundance index, emPAI [18] calculated as
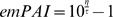
(3)where η is the total number of MS/MS spectra contributing to a given protein identification and τ is the maximum number of theoretical peptides generated from a protein sequence by trypsin digestion allowing for a maximum of one missed cleavage.

Codon Adaptation Index (CAI) was estimated as described previously [17]. The algorithm for CAI calculation was implemented in Matlab 7.0 with bioinformatics toolbox.

Data visualizations combining gene dynamics and protein annotations obtained from the Sanger Institute (Cambridge, UK) and The Institute of Genomic Research (TIGR) were also performed in Spotfire software. Information for pathway analysis was generally obtained from ScoCyc (http://scocyc.jic.bbsrc.ac.uk:1555/) and Kyoto Encyclopedia of Genes and Genomes (KEGG, [34]). All individual gene profiles discussed in this manuscript have low quantification *p*-values (≤0.05) and/or additional confidence from similar profiles from functionally grouped genes.

## Supporting Information

Figure S1 Biological replicate analysis for proteome data. Two independent biological replicate cultures were performed and samples were analyzed using iTRAQ. The resulting protein data were compared to assess quantification consistency. The figure shows a scatterplot of logarithm of stationary phase to exponential phase expression ratio from the replicates. Since the sampling time-points were not exactly same (due to variations in duration of lag phase between cultures), an overall ratio of average expression in stationary phase to exponential growth phase is shown. The ratios used here are those of 40 h : 9 h samples for replicate #2 and an average of 34 h : 7 h and 38 h : 7 h for replicate #1. Further time-points in replicate #1 were analyzed with mass spectrometry and that dataset is presented in the manuscript. Samples analyzed by MS runs 1, 2 and 3 are indicated.
(0.17 MB PDF)

Figure S2 Examples of some genes exhibiting good correlation between mRNA (blue) and protein (red) profiles. The horizontal axis corresponds to time spanning from 7 h to 38 h while the vertical axis corresponds to log_2_ expression ratio relative to 7 h sample. The numbers on the top right indicate the total number of unique peptide hits supporting each protein identification.
(0.08 MB PDF)

Figure S3 Additional examples of genes exhibiting discordant mRNA (blue) and protein (red) dynamics. The horizontal axis corresponds to time spanning from 7 h to 38 h while the vertical axis corresponds to log_2_ expression ratio relative to 7 h sample. Figure S3(a) shows additional functionally or chromosomally related genes displaying mRNA-protein discordance. Figure S3(b) shows isolated such discordance among isolated genes (genes that could not be grouped into related categories). The numbers on the top right of each panel in Figure S3(b) indicate the total number of unique peptide hits supporting each protein identification.
(0.09 MB PDF)

Figure S4 MS/MS fragmentation spectra for single peptide protein hits. This file contains a series of MS/MS fragmentation spectra for single peptide protein hits shown in Figure 5. The list also includes those single peptide hits that were sampled multiple times (i.e. multiple spectral evidence single peptide hits). In such cases, the protein number is repeated as many times as the number of spectra contributing for a given peptide.
(4.28 MB PDF)

Table S1 List of genes with probable divergent mRNA-protein behavior discovered by PCA The list shows only data for which at least two unique peptide identifications in mass spectrometry data are available. Please refer to supplementary tables S2 and S3 for complete dataset including proteins with single peptide hits and concordant mRNA-protein behavior.
(0.19 MB PDF)

Table S2 Protein identification, quantification and analysis results summary. Tab delimited text file containing mass-spectrometry protein identification and quantification summary. PCA results are also shown.
(0.29 MB TXT)

Table S3 mRNA quantification and analysis results summary for those genes identified in proteomics analysis. Tab delimited text file containing transcriptome data from microarrays. PCA results are also shown.
(0.21 MB TXT)
