# Supplementary material for: Uncovering Genes with Divergent mRNA-Protein Dynamics in Streptomyces coelicolor
Source: PLoS One. 2008 May 7;3(5):e2097. doi: 10.1371/journal.pone.0002097 (PMC2367054; doi:10.1371/journal.pone.0002097)
Supplement: Figure S1 — Biological replicate analysis for proteome data. Two independent biological replicate cultures were performed and samples were analyzed using iTRAQ. The resulting protein data were compared to assess quantification consistency. The figure shows a scatterplot of logarithm of stationary phase to exponential phase expression ratio from the replicates. Since the sampling time-points were not exactly same (due to variations in duration of lag phase between cultures), an overall ratio of average expression in stationary phase to exponential growth phase is shown. The ratios used here are those of 40 h : 9 h samples for replicate #2 and an average of 34 h : 7 h and 38 h : 7 h for replicate #1. Further time-points in replicate #1 were analyzed with mass spectrometry and that dataset is presented in the manuscript. Samples analyzed by MS runs 1, 2 and 3 are indicated. (0.17 MB PDF) [file pone.0002097.s001.pdf]

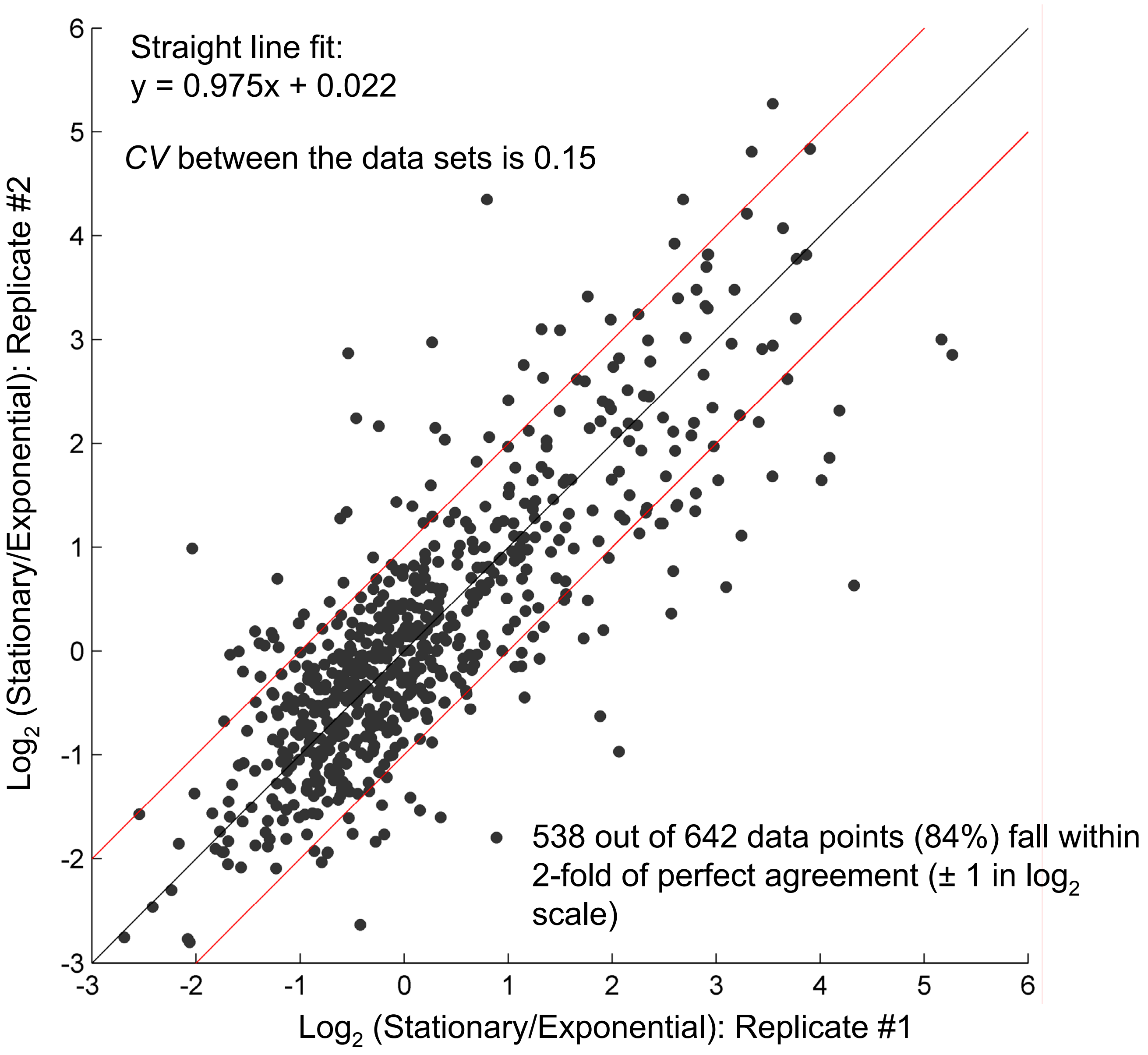

Figure S1: Biological replicate analysis for proteome data. **Two independent biological replicate cultures were performed and samples were analyzed using iTRAQ. The resulting protein data were compared to assess quantification consistency. The figure shows a scatterplot of logarithm of stationary phase to exponential phase expression ratio from the replicates. Since the sampling time-points were not exactly same (due to variations in duration of lag phase between cultures), an overall ratio of average expression in stationary phase to exponential growth phase is shown. The ratios used here are those of 40 h : 9 h samples for replicate #2 and an average of 34 h : 7 h and 38 h : 7 h for replicate #1. Further time-points in replicate #1 were analyzed with mass spectrometry and that dataset is presented in the manuscript.**
