# Supplementary material for: Uncovering Genes with Divergent mRNA-Protein Dynamics in Streptomyces coelicolor
Source: PLoS One. 2008 May 7;3(5):e2097. doi: 10.1371/journal.pone.0002097 (PMC2367054; doi:10.1371/journal.pone.0002097)
Supplement: Figure S2 — Examples of some genes exhibiting good correlation between mRNA (blue) and protein (red) profiles. The horizontal axis corresponds to time spanning from 7 h to 38 h while the vertical axis corresponds to log2 expression ratio relative to 7 h sample. The numbers on the top right indicate the total number of unique peptide hits supporting each protein identification. (0.08 MB PDF) [file pone.0002097.s002.pdf]

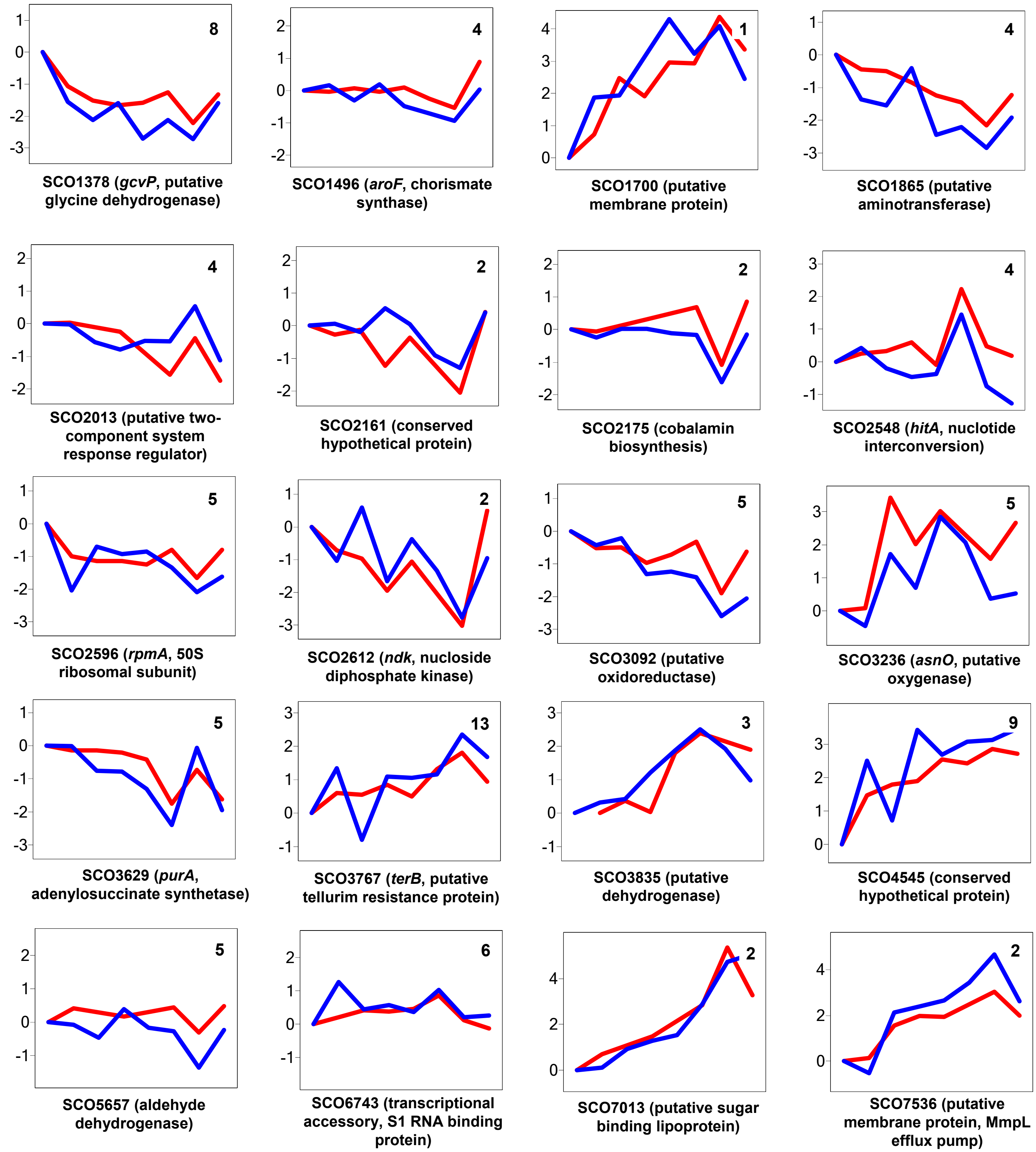

Figure S2: Examples of some genes exhibiting good correlation between mRNA (blue) and protein (red) profiles. The horizontal axis corresponds to time spanning from 7 h to 38 h while the vertical axis corresponds to log2 expression ratio relative to 7 h sample. The numbers on the top right indicate the total number of unique peptide hits supporting each protein identification.
