# Supplementary material for: Uncovering Genes with Divergent mRNA-Protein Dynamics in Streptomyces coelicolor
Source: PLoS One. 2008 May 7;3(5):e2097. doi: 10.1371/journal.pone.0002097 (PMC2367054; doi:10.1371/journal.pone.0002097)

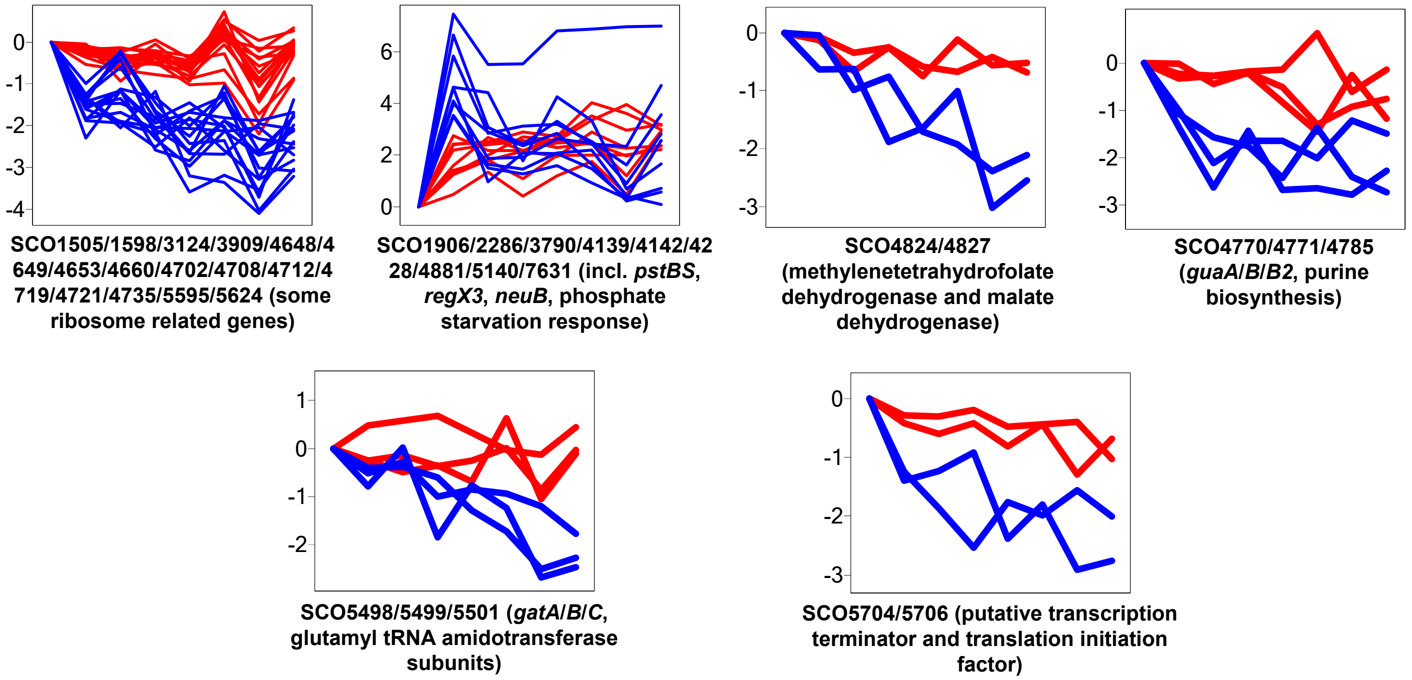


**Figure S3(a):** Additional examples of functionally or chromosomally clustered genes exhibiting discordant mRNA (blue) and protein (red) dynamics. The horizontal axis corresponds to time spanning from 7 h to 38 h while the vertical axis corresponds to $log_2$ expression ratio relative to 7 h sample.

---

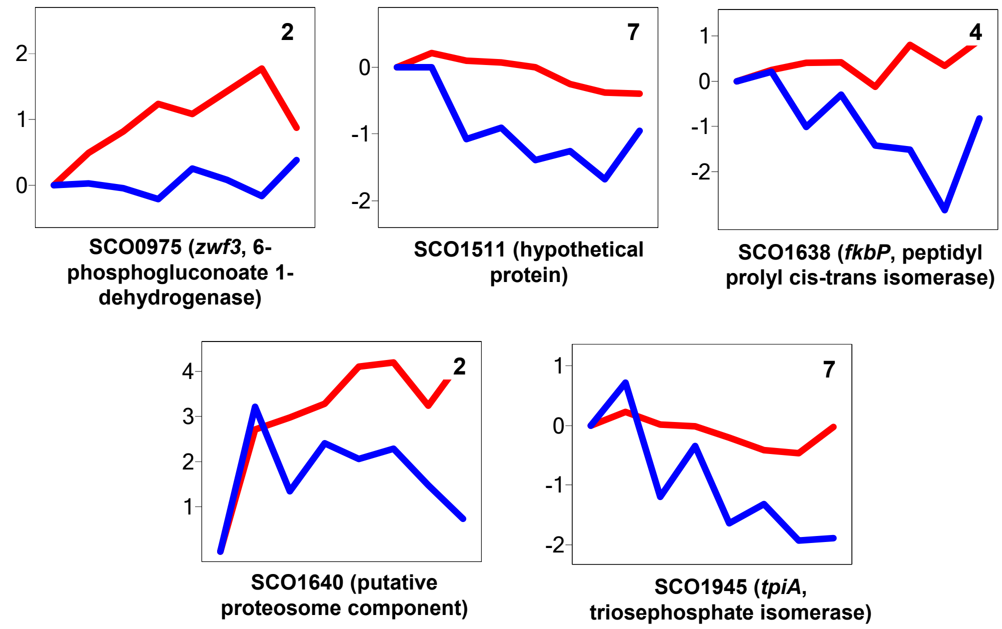


**Figure S3(b):** Examples of isolated genes that could not be clustered as functional groups exhibiting discordant mRNA (blue) and protein (red) dynamics. The horizontal axis corresponds to time spanning from 7 h to 38 h while the vertical axis corresponds to log2 expression ratio relative to 7 h sample. The numbers on the top right indicate the total number of unique peptide hits supporting each protein identification. Figure continued on next page.

## Figure S3(b) (continued)

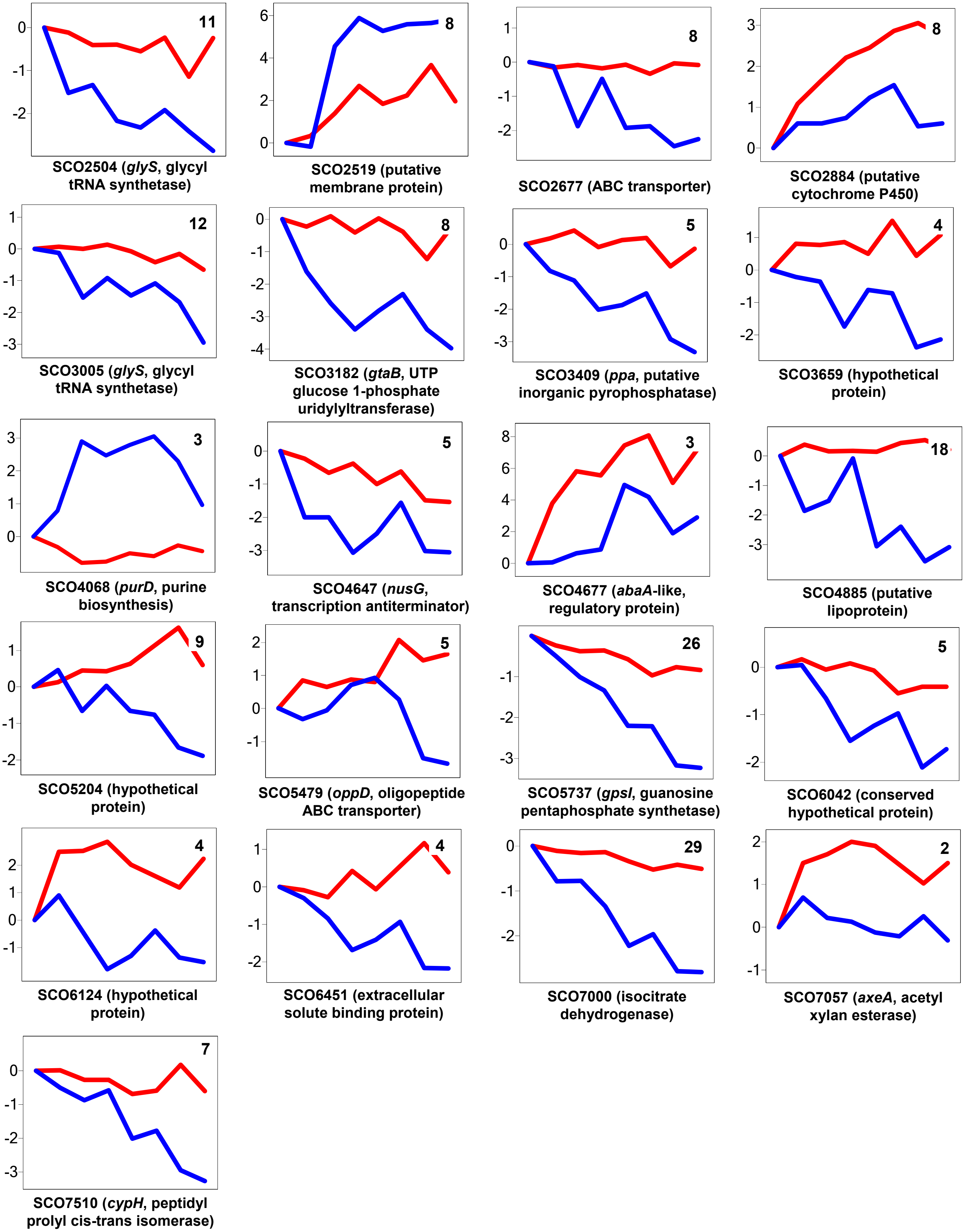

Supplement: Figure S3 — Additional examples of genes exhibiting discordant mRNA (blue) and protein (red) dynamics. The horizontal axis corresponds to time spanning from 7 h to 38 h while the vertical axis corresponds to log2 expression ratio relative to 7 h sample. Figure S3(a) shows additional functionally or chromosomally related genes displaying mRNA-protein discordance. Figure S3(b) shows isolated such discordance among isolated genes (genes that could not be grouped into related categories). The numbers on the top right of each panel in Figure S3(b) indicate the total number of unique peptide hits supporting each protein identification. (0.09 MB PDF) [file pone.0002097.s003.pdf]
