# Supplementary material for: Uncovering Genes with Divergent mRNA-Protein Dynamics in Streptomyces coelicolor
Source: PLoS One. 2008 May 7;3(5):e2097. doi: 10.1371/journal.pone.0002097 (PMC2367054; doi:10.1371/journal.pone.0002097)

This file contains a series of MS/MS fragmentation spectra for single peptide protein hits shown in Figure 5.

The list also includes those single peptide hits that were sampled multiple times (i.e. multiple spectral evidence single peptide hits). In such cases, the protein number is repeated as many times as the number of spectra contributing for a given peptide.

Protein: SCO0199 [1 of 1]

Protein Quant | Protein ID | Spectra | Summary Statistics

Spectrum List

Spectrum	Time	Prec MW	Prec m/z	Prec z	Best Sequence	Modifications	Conf	Theor MW	z	115:114	%Err 115:114	116:
32.1.1.2471.3	46.979	1674.8622	559.2947	3	IDVTDDKLQLAR	iTRAQ@N-term, iTRAQ(K)@7	99	1673.9555	3	0.8960	2.05	

Peptide ID Hypotheses

Conf	Sc	Prot N	Sequence	Modifications	Theor MW	Theor m/z	z	ΔMass
99	18	293	IDVTDDKLQLAR	iTRAQ@N-term, iTRAQ(K)@7	1673.9555	558.9925	3	0.9066
< 1	8		GGADEDAGSGLTGIRR	iTRAQ@N-term	1674.8407	559.2875	3	0.0215
< 1	8		LGGDEFVILANGLGR	iTRAQ@N-term, Deamidatio...	1674.9062	559.3094	3	-0.0441
< 1	8		MKVLSRVTQAWGR	No iTRAQ@N-term, iTRAQ(...	1674.9473	559.3231	3	-0.0852
< 1	8		SGESTTAVVAGVNLNL	iTRAQ@N-term	1674.8910	559.3043	3	-0.0288

Precursor MS Region

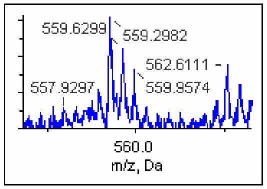

Fragmentation Evidence for Peptide

ITR | IDVTDDK[ITR]LQLAR

Residue	b	y
I	258.1934	1674.9628
D	373.2203	1417.7767
V	472.2888	1302.7498
T	573.3364	1203.6814
D	688.3634	1102.6337
D	803.3903	987.6067
K[ITR]	1075.5873	872.5798
L	1188.6714	600.3828
Q	1316.7300	487.2987
L	1429.8141	359.2401
A	1500.8512	246.1561
R	1656.9523	175.1190

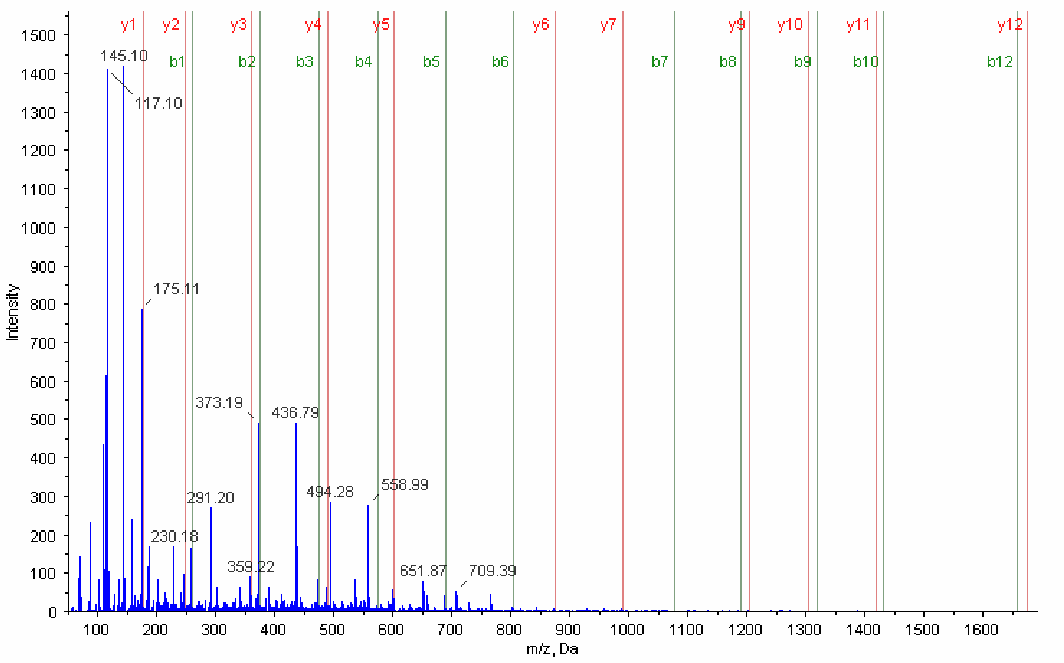

Protein: SCO1390 [1 of 2]

Protein Quant | Protein ID | Spectra | Summary Statistics

Spectrum List

Spectrum	Time	Prec MW	Prec m/z	Prec z	Best Sequence	Modifications	Conf	Theor MW	z	115:114	%Err 115:114	116:
29.1.1.1075.3	42.843	1337.7600	669.8873	2	AGESLFLWK	iTRAQ@N-term, iTRAQ(K)@9	99	1337.7587	2	0.6440	5.44	

Peptide ID Hypotheses

Conf	Sc	Prot N	Sequence	Modifications	Theor MW	Theor m/z	z	ΔMass
99	13	624	AGESLFLWK	iTRAQ@N-term, iTRAQ(K)@9	1337.7587	669.8867	2	0.0013
< 1	7		ASGDGTVVFTLK	No iTRAQ@N-term, iTRAQ(...	1337.7313	669.8729	2	0.0287
< 1	6		ALSRRHDDPK	No iTRAQ@N-term, iTRAQ(...	1337.7286	669.8716	2	0.0314
< 1	6		LTDLITAELGPPV	No iTRAQ@N-term	1337.7443	669.8794	2	0.0158
< 1	6		REYRAINK	iTRAQ@N-term, Deamidatio...	1337.7659	669.8903	2	-0.0059

Precursor MS Region

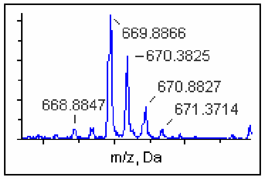

Fragmentation Evidence for Peptide

ITR AGESLFLWK[ITR]

Res	b	y
A	216.1465	1338.7660
G	273.1679	1123.6268
E	402.2105	1066.6053
S	489.2425	937.5627
L	602.3266	850.5307
F	749.3950	737.4467
L	862.4791	590.3782
W	1048.5584	477.2942
K[I	1320.7554	291.2149

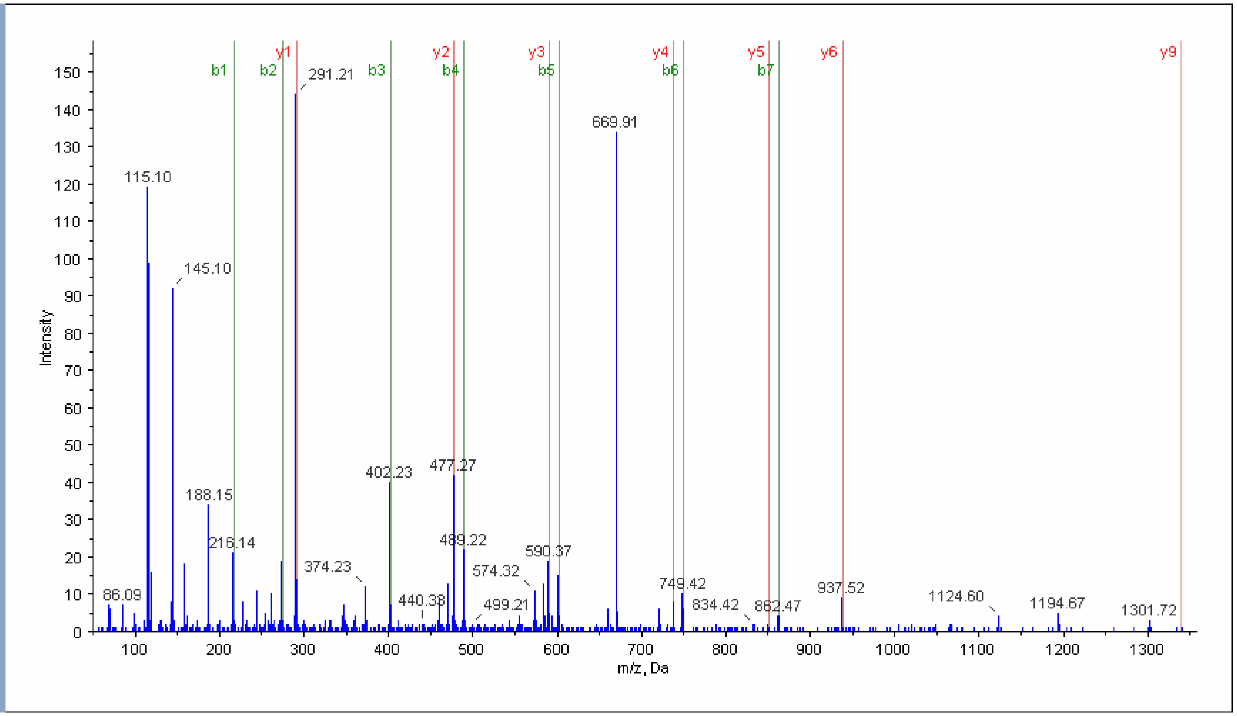

Protein: SCO1390 [2 of 2]

Protein Quant | Protein ID | Spectra | Summary Statistics

Spectrum List

Spectrum	Time	Prec MW	Prec m/z	Prec z	Best Sequence	Modifications	Conf	Theor MW	z	115:114	%Err 115:114	116:
30.1.1.1055.2	43.456	1337.7534	669.8840	2	AGESLFLWK	iTRAQ@N-term, iTRAQ(K)@9	99	1337.7587	2	0.9557	8.31	

Peptide ID Hypotheses

Conf	Sc	Prot N	Sequence	Modifications	Theor MW	Theor m/z	z	ΔMass
99	13	624	AGESLFLWK	iTRAQ@N-term, iTRAQ(K)@9	1337.7587	669.8867	2	-0.0053
< 1	7	135	QGAIAEFGEK	iTRAQ@N-term, Deamidatio...	1337.7071	669.8609	2	0.0463
< 1	7		QSTTRYVVLK	No iTRAQ@N-term, iTRAQ(...	1337.7789	669.8967	2	-0.0255
< 1	6		APGAPGAAEQGLR	iTRAQ@N-term	1337.7174	669.8660	2	0.0360
< 1	6		GAESWRPHVR	iTRAQ@N-term	1337.7075	669.8610	2	0.0459

Precursor MS Region

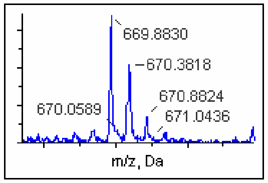

Fragmentation Evidence for Peptide

ITR | AGESLFLWK[ITR]

Res	b	y
A	216.1465	1338.7660
G	273.1679	1123.6268
E	402.2105	1066.6053
S	489.2425	937.5627
L	602.3266	850.5307
F	749.3950	737.4467
L	862.4791	590.3782
W	1048.5584	477.2942
K[I	1320.7554	291.2149

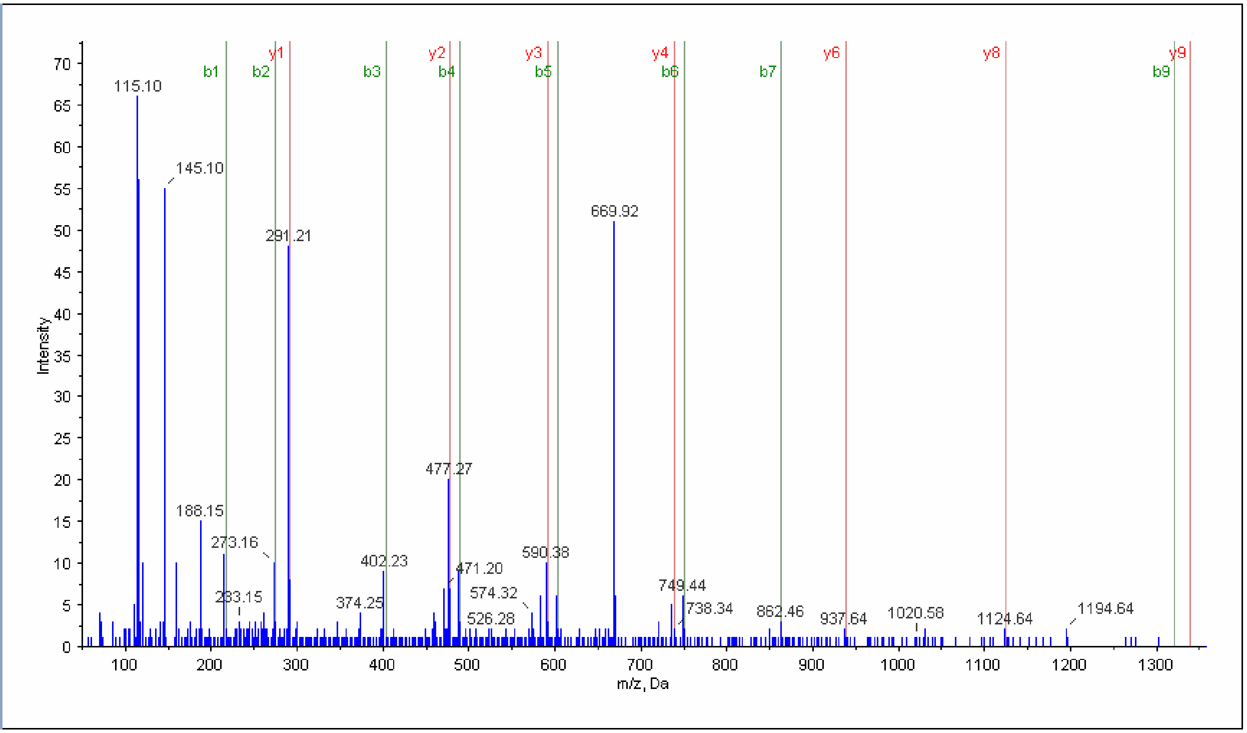

Protein: SCO1439, HisE [1 of 1]

Protein Quant | Protein ID | Spectra | Summary Statistics

Spectrum List

Spectrum	Time	Prec MW	Prec m/z	Prec z	Best Sequence	Modifications	Conf	Theor MW	z	115:114	%Err 115:114	116:
3.1.1.1043.4	44.227	1345.6940	673.8542	2	GISLDDVYAHL	iTRAQ@N-term	99	1345.7000	2	0.8188	19.16	

Peptide ID Hypotheses

Conf	Sc	Prot N	Sequence	Modifications	Theor MW	Theor m/z	z	ΔMass
99	12	633	GISLDDVYAHL	iTRAQ@N-term	1345.7000	673.8573	2	-0.0061
<1	6		AEGNRTAEILK	No iTRAQ@N-term, Deamid...	1345.7324	673.8735	2	-0.0384
<1	6		DNNASAALGPFLR	No iTRAQ@N-term, Deamid...	1345.6627	673.8386	2	0.0313
<1	5		GSVDQQGAVNAR	iTRAQ@N-term, Deamidatio...	1345.6709	673.8427	2	0.0231
<1	5		LSGEVQELTAR	iTRAQ@N-term	1345.7324	673.8735	2	-0.0384

Precursor MS Region

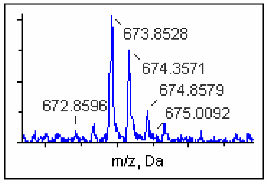

Fragmentation Evidence for Peptide

ITR GISLDDVYAHL

Res	b	y
G	202.1308	1346.7072
I	315.2149	1145.5837
S	402.2469	1032.4997
L	515.3310	945.4676
D	630.3579	832.3836
D	745.3848	717.3566
V	844.4533	602.3297
Y	1007.5166	503.2613
A	1078.5537	340.1979
H	1215.6126	269.1608
L	1328.6967	132.1019

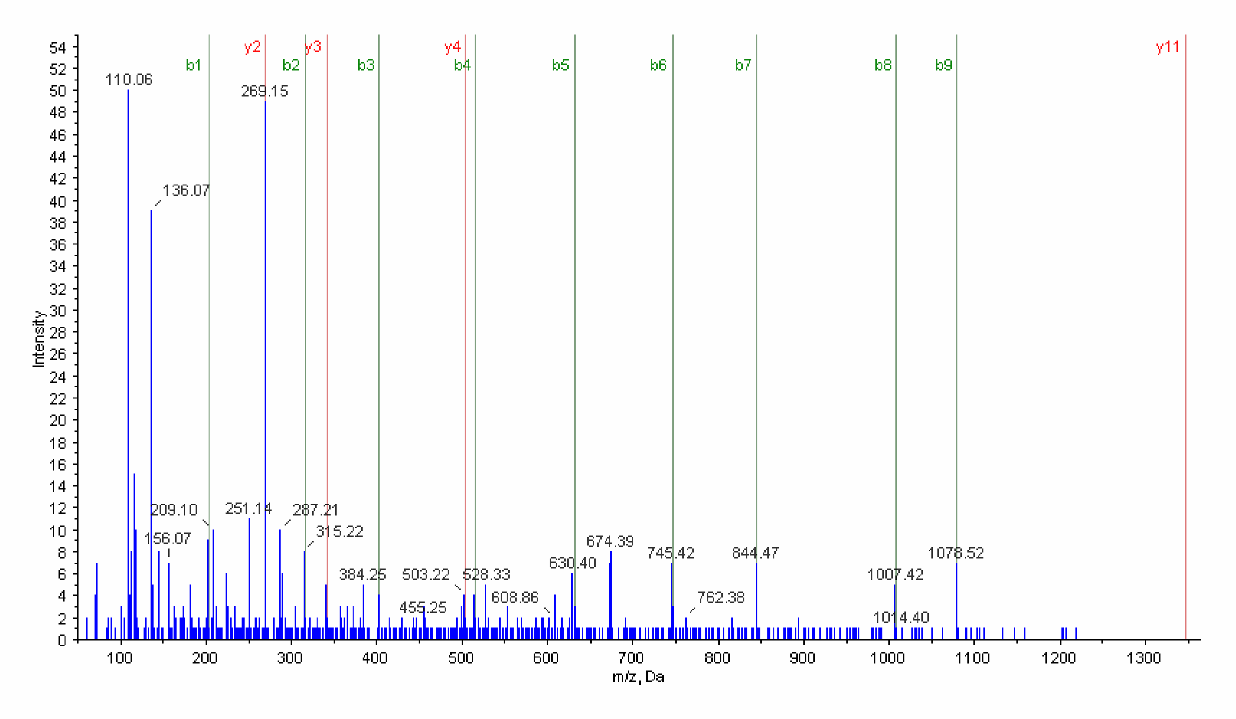

Protein: SCO1461, GuaB2 [1 of 1]

Protein Quant | Protein ID | Spectra | Summary Statistics

Spectrum List

Spectrum	Time	Prec MW	Prec m/z	Prec z	Best Sequence	Modifications	Conf	Theor MW	z	115:114	%Err 115:114	116:
12.1.1.2283.3	43.195	1352.6938	677.3542	2	ALFEEGISTSR	iTRAQ@N-term	99	1352.7058	2	0.8799	1.81	

Peptide ID Hypotheses

Conf	Sc	Prot N	Sequence	Modifications	Theor MW	Theor m/z	z	ΔMass
99	14	355	ALFEEGISTSR	iTRAQ@N-term	1352.7058	677.3602	2	-0.0120
< 1	11		ASGFLSGVTGSAR	iTRAQ@N-term	1352.7171	677.3658	2	-0.0232
< 1	9		AIQAHLNDAVR	iTRAQ@N-term, Deamidatio...	1352.7171	677.3658	2	-0.0232
< 1	9		LNTTSHELAIVR	No iTRAQ@N-term	1352.7413	677.3779	2	-0.0474
< 1	9		SRGGCTRSSGPSR	No iTRAQ@N-term, MMTS(...	1352.6038	677.3092	2	0.0900

Precursor MS Region

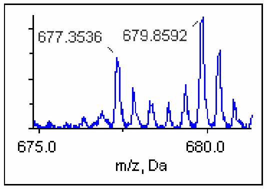

Fragmentation Evidence for Peptide

ITR ALFEEGISTSR

Residue	b	y
A	216.1465	1353.7131
L	329.2305	1138.5739
F	476.2989	1025.4898
E	605.3415	878.4214
E	734.3841	749.3788
G	791.4056	620.3362
I	904.4896	563.3148
S	991.5217	450.2307
T	1092.5694	363.1987
S	1179.6014	262.1510
R	1335.7025	175.1190

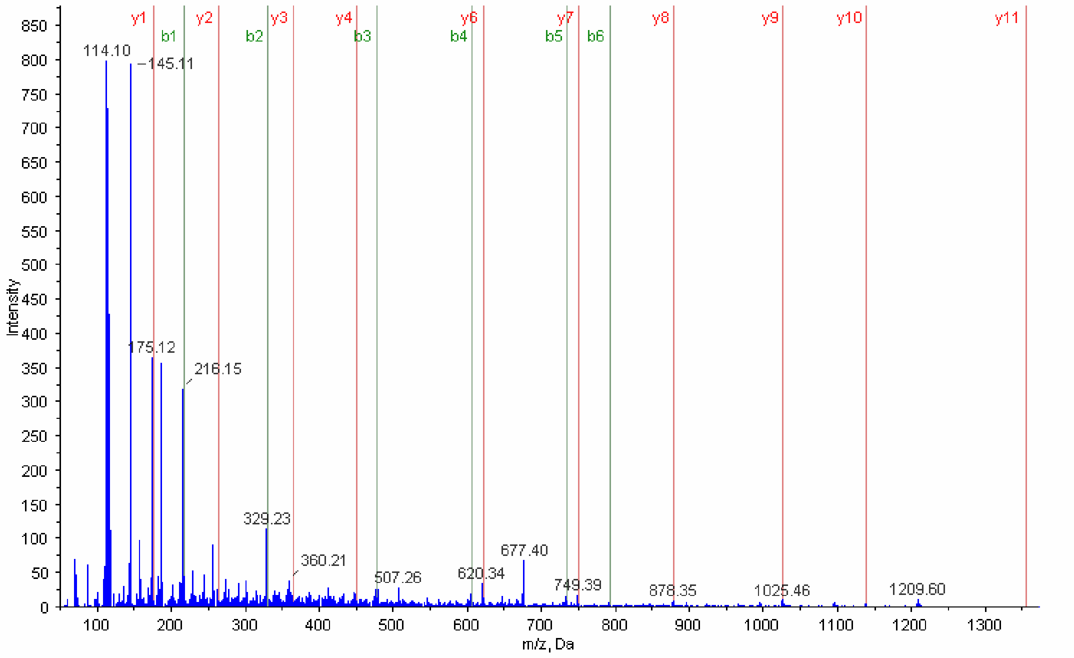

Protein: SCO1514, Apt [1 of 1]

Protein Quant | Protein ID | Spectra | Summary Statistics

Spectrum List

Spectrum	Time	Prec MW	Prec m/z	Prec z	Best Sequence	Modifications	Conf	Theor MW	z	115:114	%Err 115:114	116:
3.1.1.1027.4	40.536	1256.7623	629.3884	2	GFILGAPVALR	iTRAQ@N-term	99	1256.7727	2	1.0892	9.71	

Peptide ID Hypotheses

Conf	Sc	Prot N	Sequence	Modifications	Theor MW	Theor m/z	z	ΔMass
99	15	647	GFILGAPVALR	iTRAQ@N-term	1256.7727	629.3936	2	-0.0104
< 1	6	32	VVVLAQEEAR	iTRAQ@N-term	1256.7211	629.3678	2	0.0412
< 1	7		GFLPAPGWLR	iTRAQ@N-term	1256.7152	629.3649	2	0.0471
< 1	6		LRLTARGDLSR	No iTRAQ@N-term	1256.7314	629.3729	2	0.0310
< 1	6		RGRVTITDIAR	No iTRAQ@N-term	1256.7314	629.3729	2	0.0310

Precursor MS Region

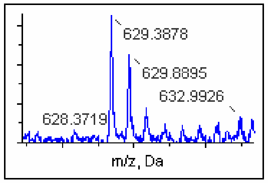

Fragmentation Evidence for Peptide

ITR GFILGAPVALR

Res	b	y
G	202.1308	1257.7800
F	349.1992	1056.6564
I	462.2833	909.5880
L	575.3673	796.5039
G	632.3888	683.4199
A	703.4259	626.3984
P	800.4787	555.3613
V	899.5471	458.3085
A	970.5842	359.2401
L	1083.6683	288.2030
R	1239.7694	175.1190

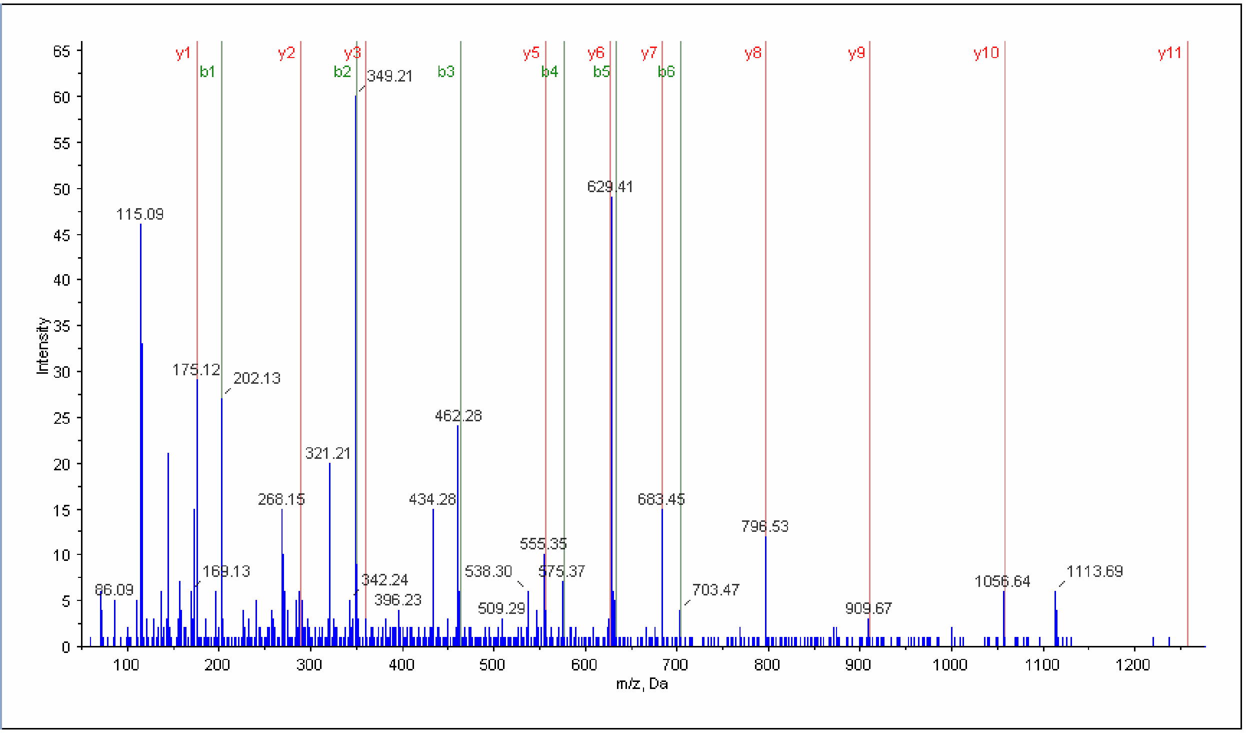

Protein: SCO1515, SecF [1 of 1]

Protein Quant | Protein ID | Spectra | Summary Statistics

Spectrum List

Spectrum	Time	Prec MW	Prec m/z	Prec z	Best Sequence	Modifications	Conf	Theor MW	z	115:114	%Err 115:114	116:
45.1.1.981.3	31.127	1687.8856	563.6358	3	LHHGEVGYDFVK	iTRAQ@N-term, iTRAQ(K)@...	99	1687.8926	3	1.3548	12.44	

Peptide ID Hypotheses

Conf	Sc	Prot N	Sequence	Modifications	Theor MW	Theor m/z	z	ΔMass
99	13	702	LHHGEVGYDFVK	iTRAQ@N-term, iTRAQ(K)@...	1687.8926	563.6381	3	-0.0070
< 1	6		LLATPRDLRFPHPR	No iTRAQ@N-term	1687.9635	563.6618	3	-0.0779
< 1	6		LTENVNELAGNLTR	iTRAQ@N-term, Deamidatio...	1687.8863	563.6361	3	-0.0007
< 1	5		TDDLETAADLPEVR	iTRAQ@N-term	1687.8387	563.6202	3	0.0469
< 1	4		ELPPAVDDVSFEAR	iTRAQ@N-term	1687.8539	563.6252	3	0.0317

Precursor MS Region

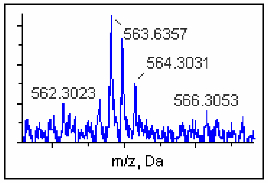

Fragmentation Evidence for Peptide

ITR LHHGEVGYDFVK[ITR]

Res	b	y
L	258.1934	1688.8999
H	395.2523	1431.7137
H	532.3112	1294.6548
G	589.3327	1157.5959
E	718.3753	1100.5744
V	817.4437	971.5318
G	874.4652	872.4634
Y	1037.5285	815.4420
D	1152.5554	652.3786
F	1299.6238	537.3517
V	1398.6923	390.2833
K[I	1670.8893	291.2149

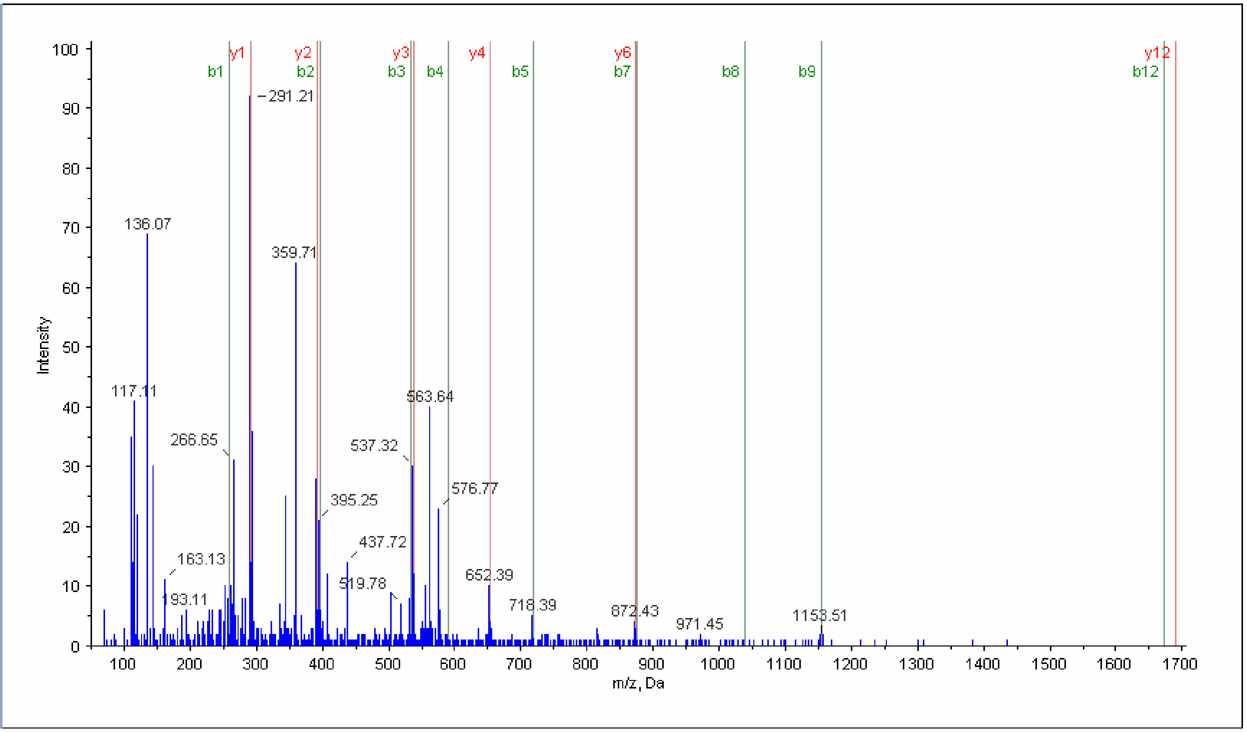

Protein: SCO1516, SecD [1 of 2]

Protein Quant | Protein ID | Spectra | Summary Statistics

Spectrum List

Spectrum	Time	Prec MW	Prec m/z	Prec z	Best Sequence	Modifications	Conf	Theor MW	z	115:114	%Err 115:114	116:
33.1.1.2318.2	40.290	0.0000	801.4051	0	AVPEAGQESAINK	iTRAQ@N-term, iTRAQ(K)@...	99	1600.8664	2	1.0581	4.55	

Peptide ID Hypotheses

Conf	Sc	Prot N	Sequence	Modifications	Theor MW	Theor m/z	z	ΔMass
99	14	317	AVPEAGQESAINK	iTRAQ@N-term, iTRAQ(K)@...	1600.8664	801.4405	2	-0.0708
< 1	8		AASSGMSPDQFAQAVVEQGQVPLLVGEVAR	iTRAQ@N-term, Oxidation(...	3201.6098	801.4097	4	-0.0185
< 1	8		AVADTLLGRIDGSGTLSLDEVR	iTRAQ@N-term	2401.2934	801.4384	3	-0.1000
< 1	8		LARAEAVAAVDRFGR	No iTRAQ@N-term	1600.8798	801.4472	2	-0.0841
< 1	8		LIAEGESLLFPASVR	No iTRAQ@N-term	1600.8825	801.4485	2	-0.0868

Precursor MS Region

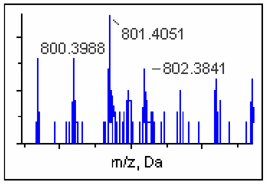

Fragmentation Evidence for Peptide

ITR | AVPEAGQESAINK[ITR]

Residue	b	y
A	216.1465	1601.8737
V	315.2149	1386.7345
P	412.2676	1287.6661
E	541.3102	1190.6133
A	612.3473	1061.5707
G	669.3688	990.5336
Q	797.4274	933.5122
E	926.4700	805.4536
S	1013.5020	676.4110
A	1084.5391	589.3790
I	1197.6232	518.3419
N	1311.6661	405.2578
K[ITR]	1583.8631	291.2149

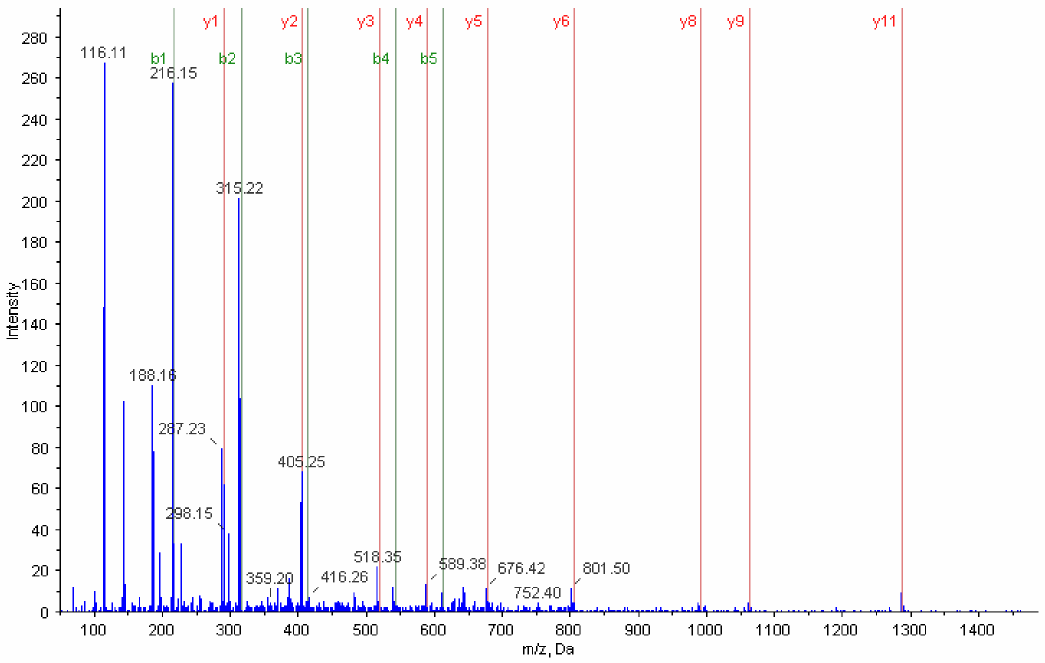

Protein: SCO1516, SecD [2 of 2]

Protein Quant | Protein ID | Spectra | Summary Statistics

Spectrum List

Spectrum	Time	Prec MW	Prec m/z	Prec z	Best Sequence	Modifications	Conf	Theor MW	z	115:114	%Err 115:114	116:
33.1.1.2319.2	40.341	1600.8800	801.4473	2	AVPEAGQESAINK	iTRAQ@N-term, iTRAQ(K)@...	99	1600.8664	2	1.0562	3.55	

Peptide ID Hypotheses

Conf	Sc	Prot N	Sequence	Modifications	Theor MW	Theor m/z	z	ΔMass
99	15	317	AVPEAGQESAINK	iTRAQ@N-term, iTRAQ(K)@...	1600.8664	801.4405	2	0.0136
<1	5	336	EAGIPVFGPSGEAAR	iTRAQ@N-term	1600.8331	801.4238	2	0.0469
<1	8		LARAEAVAAVDRFGR	No iTRAQ@N-term	1600.8798	801.4472	2	0.0002
<1	7		AQAPAQPQAAAPAPAP	iTRAQ@N-term, Deamidatio...	1600.8331	801.4238	2	0.0469
<1	7		LAVDAAEQTRVRK	No iTRAQ@N-term, Deamid...	1600.9019	801.4582	2	-0.0219

Precursor MS Region

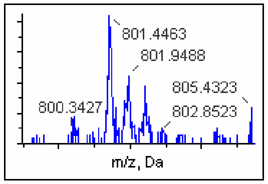

Fragmentation Evidence for Peptide

ITR AVPEAGQESAINK[ITR]

Residue	b	y
A	216.1465	1601.8737
V	315.2149	1386.7345
P	412.2676	1287.6661
E	541.3102	1190.6133
A	612.3473	1061.5707
G	669.3688	990.5336
Q	797.4274	933.5122
E	926.4700	805.4536
S	1013.5020	676.4110
A	1084.5391	589.3790
I	1197.6232	518.3419
N	1311.6661	405.2578
K[ITR]	1583.8631	291.2149

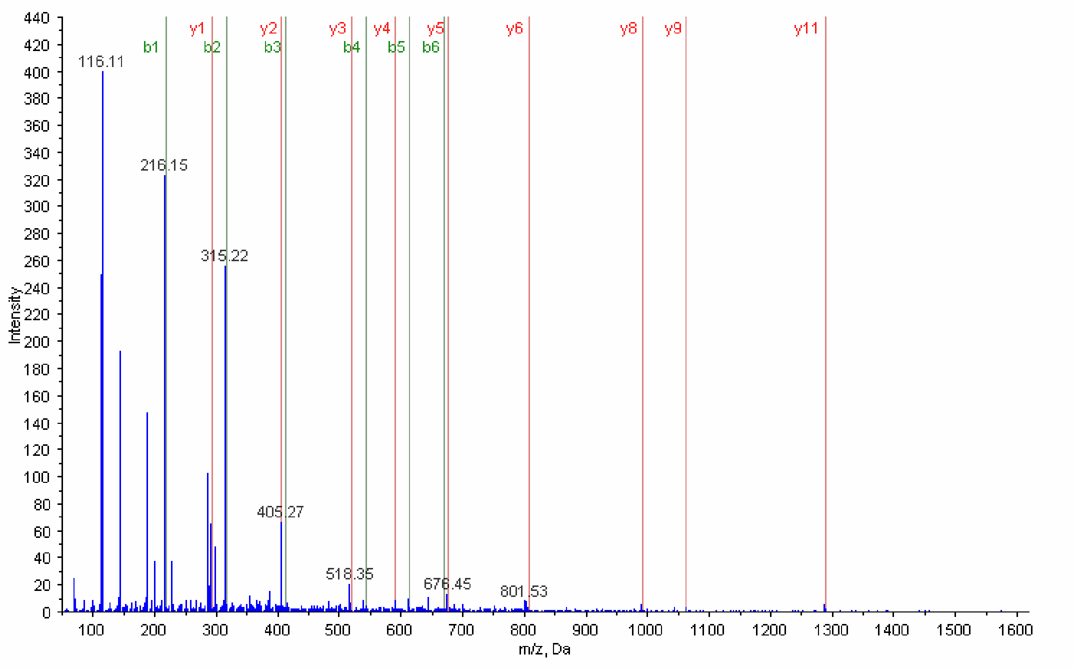

Protein: SCO1760, LivG [1 of 3]

Protein Quant | Protein ID | Spectra | Summary Statistics

Spectrum List

Spectrum	Time	Prec MW	Prec m/z	Prec z	Best Sequence	Modifications	Conf	Theor MW	z	115:114	%Err 115:114	116:
17.1.1.1198.2	42.602	1580.9564	527.9927	3	TIAAAAPLGIVVEGR	iTRAQ@N-term	99	1580.9372	3	0.8420	16.01	

Peptide ID Hypotheses

Conf	Sc	Prot N	Sequence	Modifications	Theor MW	Theor m/z	z	ΔMass
99	16	846	TIAAAAPLGIVVEGR	iTRAQ@N-term	1580.9372	527.9864	3	0.0192
< 1	7		LTAPAAALVNALRR	iTRAQ@N-term, Deamidatio...	1580.9484	527.9901	3	0.0080
< 1	6		DVLLAQAAVRIAADR	No iTRAQ@N-term	1580.8999	527.9739	3	0.0566
< 1	6		ERINKELVLDAR	PGA of E@N-term, iTRAQ(K...	1580.9120	527.9779	3	0.0444
< 1	6		ILEGGPVIDVRGGR	iTRAQ@N-term	1580.9120	527.9779	3	0.0444

Precursor MS Region

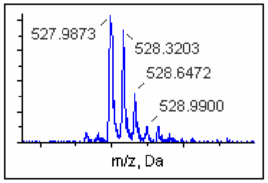

Fragmentation Evidence for Peptide

ITR TIAAAAPLGIVVEGR

Res	b	y
T	246.1570	1581.9445
I	359.2411	1336.7947
A	430.2782	1223.7106
A	501.3153	1152.6735
A	572.3524	1081.6364
A	643.3895	1010.5993
P	740.4423	939.5622
L	853.5264	842.5094
G	910.5478	729.4254
I	1023.6319	672.4039
V	1122.7003	559.3198
V	1221.7687	460.2514
E	1350.8113	361.1830
G	1407.8328	232.1404
R	1563.9339	175.1190

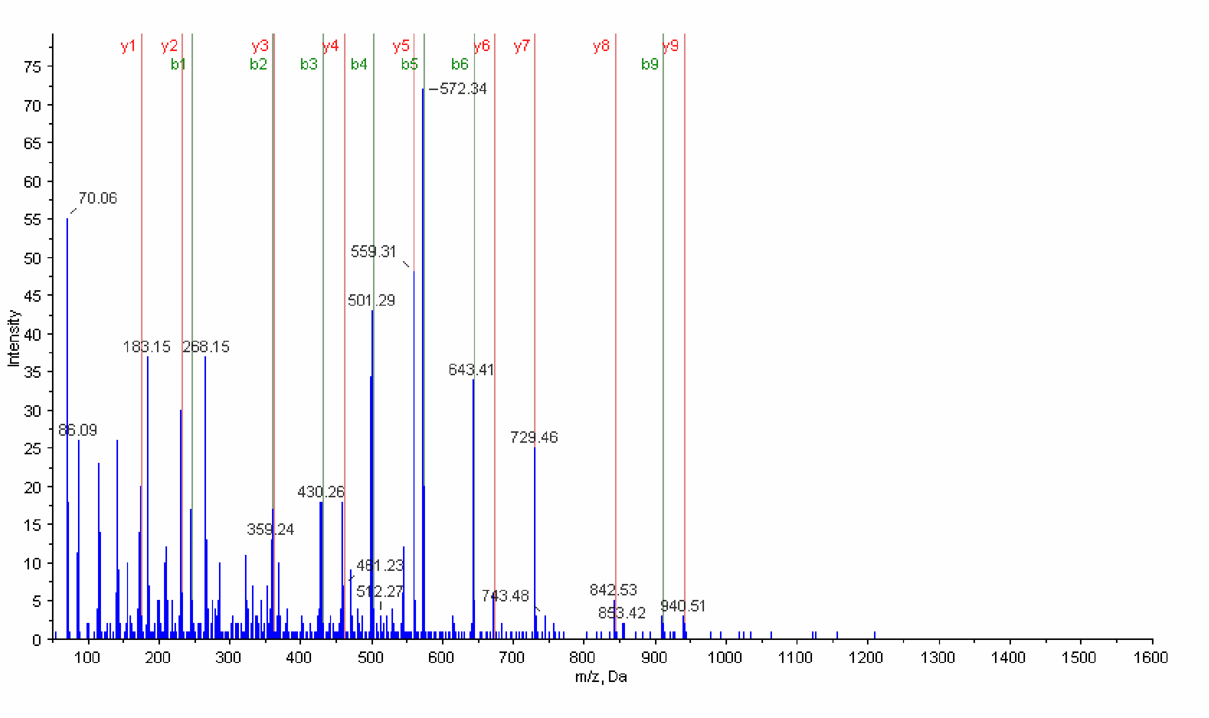

Protein: SCO1760, LivG [2 of 3]

Protein Quant | Protein ID | Spectra | Summary Statistics

Spectrum List

Spectrum	Time	Prec MW	Prec m/z	Prec z	Best Sequence	Modifications	Conf	Theor MW	z	115:114	%Err 115:114	116:
17.1.1.1198.5	42.755	1580.9410	527.9876	3	TIAAAAPLGIVVEGR	iTRAQ@N-term	99	1580.9372	3			

Peptide ID Hypotheses

Conf	Sc	Prot N	Sequence	Modifications	Theor MW	Theor m/z	z	ΔMass
99	14	846	TIAAAAPLGIVVEGR	iTRAQ@N-term	1580.9372	527.9864	3	0.0038
< 1	6		DVLLAQAAVRIAADR	No iTRAQ@N-term	1580.8999	527.9739	3	0.0412
< 1	6		EGIPLGEVLQAGVR	iTRAQ@N-term	1580.9008	527.9742	3	0.0402
< 1	6		GADPGRVAAAALRGR	iTRAQ@N-term	1580.8981	527.9733	3	0.0429
< 1	6		LTAPAAALVNALRR	iTRAQ@N-term, Deamidatio...	1580.9484	527.9901	3	-0.0074

Precursor MS Region

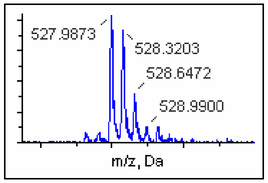

Fragmentation Evidence for Peptide

ITR | TIAAAAPLGIVVEGR

Res	b	y
T	246.1570	1581.9445
I	359.2411	1336.7947
A	430.2782	1223.7106
A	501.3153	1152.6735
A	572.3524	1081.6364
A	643.3895	1010.5993
P	740.4423	939.5622
L	853.5264	842.5094
G	910.5478	729.4254
I	1023.6319	672.4039
V	1122.7003	559.3198
V	1221.7687	460.2514
E	1350.8113	361.1830
G	1407.8328	232.1404
R	1563.9339	175.1190

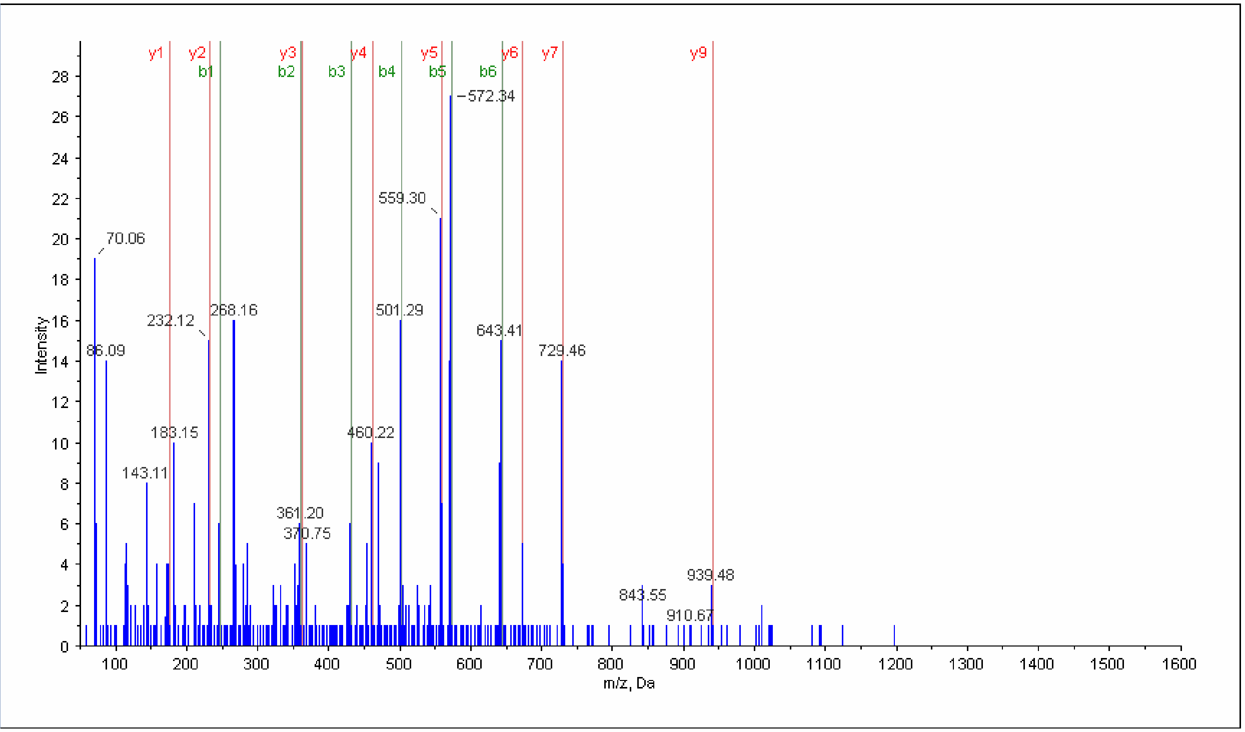

Protein: SCO1760, LivG [3 of 3]

Protein Quant | Protein ID | Spectra | Summary Statistics

Spectrum List

Spectrum	Time	Prec MW	Prec m/z	Prec z	Best Sequence	Modifications	Conf	Theor MW	z	115:114	%Err 115:114	116:
17.1.1.1197.2	42.422	1580.9330	527.9849	3	TIAAAAPLGIVVEGR	iTRAQ@N-term	99	1580.9372	3			

Peptide ID Hypotheses

Conf	Sc	Prot N	Sequence	Modifications	Theor MW	Theor m/z	z	ΔMass
99	16	846	TIAAAAPLGIVVEGR	iTRAQ@N-term	1580.9372	527.9864	3	-0.0042
<1	6		GADPGRVAAAALRGR	iTRAQ@N-term	1580.8981	527.9733	3	0.0349
<1	5		ASGPSAGRPLPKR	iTRAQ@N-term, iTRAQ(K)@...	1580.9355	527.9858	3	-0.0025
<1	5		EVPLGGPRAMRVR	iTRAQ@N-term	1580.9055	527.9758	3	0.0275
<1	5		LTAPAAALVNALRR	iTRAQ@N-term, Deamidatio...	1580.9484	527.9901	3	-0.0154

Precursor MS Region

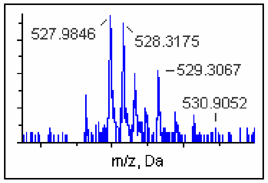

Fragmentation Evidence for Peptide

ITR TIAAAAPLGIVVEGR

Res	b	y
T	246.1570	1581.9445
I	359.2411	1336.7947
A	430.2782	1223.7106
A	501.3153	1152.6735
A	572.3524	1081.6364
A	643.3895	1010.5993
P	740.4423	939.5622
L	853.5264	842.5094
G	910.5478	729.4254
I	1023.6319	672.4039
V	1122.7003	559.3198
V	1221.7687	460.2514
E	1350.8113	361.1830
G	1407.8328	232.1404
R	1563.9339	175.1190

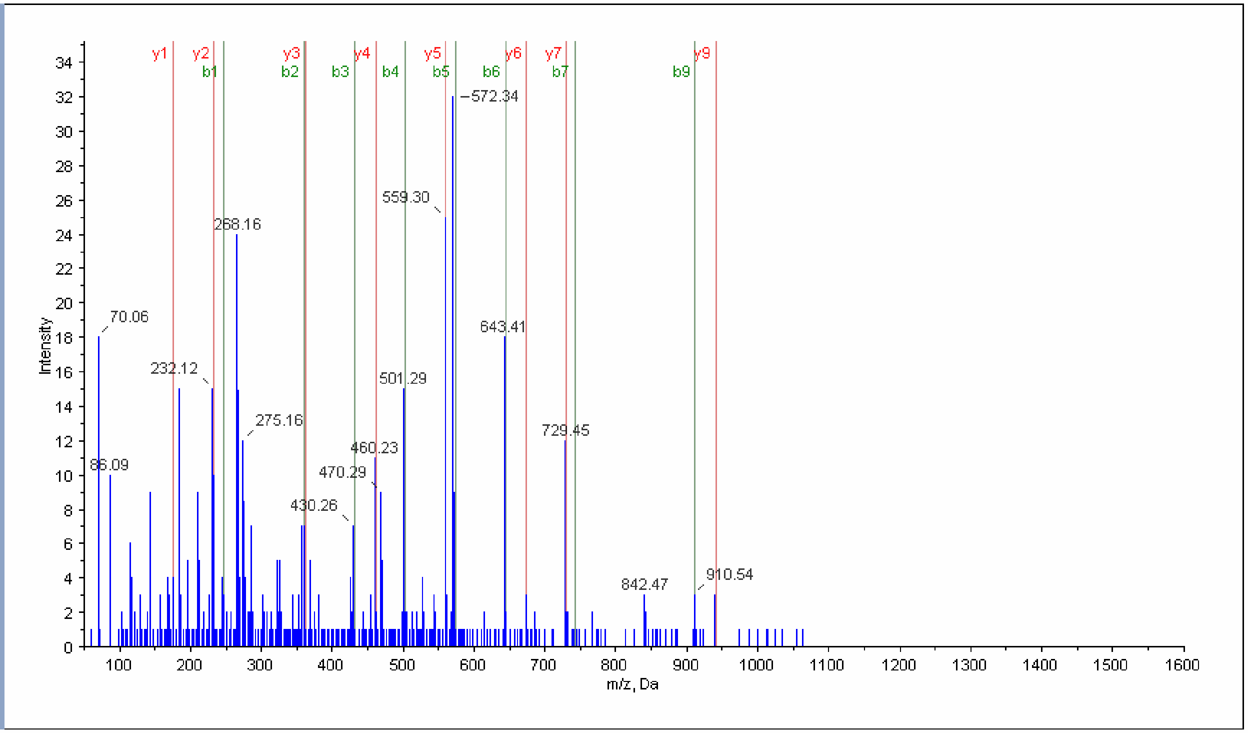

Protein: SCO1808 [1 of 2]

Protein Quant | Protein ID | Spectra | Summary Statistics

Spectrum List

Spectrum	Time	Prec MW	Prec m/z	Prec z	Best Sequence	Modifications	Conf	Theor MW	z	115:114	%Err 115:114	116:
19.1.1.2130.2	51.952	2333.4148	778.8122	3	TALATEAAALGVDIAVVAAGLHR	iTRAQ@N-term	99	2333.3190	3	0.8252	9.90	

Peptide ID Hypotheses

Conf	Sc	Prot N	Sequence	Modifications	Theor MW	Theor m/z	z	ΔMass
99	22	357	TALATEAAALGVDIAVVAAGLHR	iTRAQ@N-term	2333.3190	778.7803	3	0.0958
< 1	8		LRPDADLILSLDPDGMPWGPR	No iTRAQ@N-term	2333.1839	778.7352	3	0.2309
< 1	7		AAERLDGYTGAVRDLLGGEAR	iTRAQ@N-term	2333.2211	778.7476	3	0.1937
< 1	7		AALSPGMVRWQYLNGVRDR	iTRAQ@N-term, Deamidatio...	2333.2186	778.7468	3	0.1962
< 1	7		ALGPNLVTATACASGGTALGTAAR	iTRAQ@N-term, MMTS(C)@...	2333.1954	778.7391	3	0.2194

Precursor MS Region

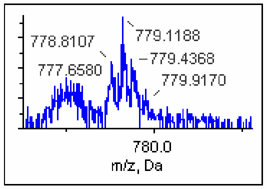

Fragmentation Evidence for Peptide

ITR TALATEAAALGVDIAVVAAGLHR

Residue	b	y
T	246.1570	2334.3262
A	317.1941	2089.1764
L	430.2782	2018.1393
A	501.3153	1905.0552
T	602.3630	1834.0181
E	731.4056	1732.9704
A	802.4427	1603.9279
A	873.4798	1532.8907
A	944.5169	1461.8536
L	1057.6010	1390.8165
G	1114.6224	1277.7324
V	1213.6909	1220.7110
D	1328.7178	1121.6426
I	1441.8019	1006.6156
A	1512.8390	893.5316
V	1611.9074	822.4944
V	1710.9758	723.4260
A	1782.0129	624.3576
A	1853.0500	553.3205
G	1910.0715	482.2834
L	2023.1556	425.2619
H	2160.2145	312.1779
R	2316.3156	175.1190

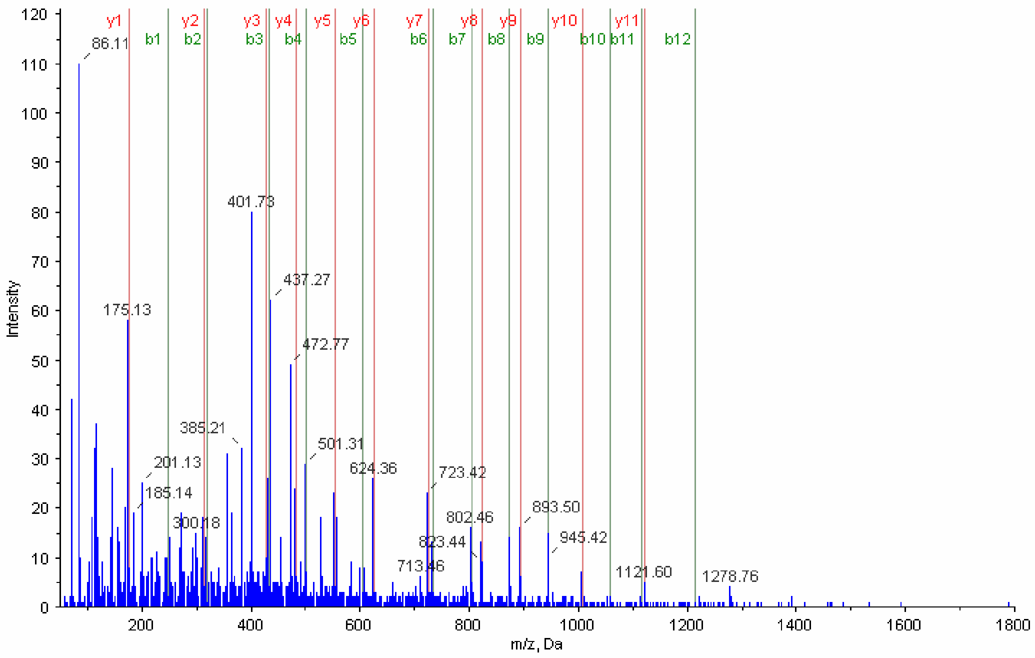

Protein: SCO1808 [2 of 2]

Protein Quant | Protein ID | Spectra | Summary Statistics

Spectrum List

Spectrum	Time	Prec MW	Prec m/z	Prec z	Best Sequence	Modifications	Conf	Theor MW	z	115:114	%Err 115:114	116:
20.1.1.2318.2	55.752	2333.3909	778.8043	3	TALATEAAALGVDIAVVAAGLHR	iTRAQ@N-term	99	2333.3188	3	0.9024	6.75	

Peptide ID Hypotheses

Conf	Sc	Prot N	Sequence	Modifications	Theor MW	Theor m/z	z	ΔMass
99	17	357	TALATEAAALGVDIAVVAAGLHR	iTRAQ@N-term	2333.3188	778.7802	3	0.0721
<1	7		LGGDLAYTTVITILTRLLEK	No iTRAQ@N-term, iTRAQ(...	2333.3691	778.7970	3	0.0218
<1	7		LVHDRTKNLVRPTTELAAR	No iTRAQ@N-term, iTRAQ(...	2333.3413	778.7877	3	0.0496
<1	5		AAERLDGYTGAVRDLLGGEAR	iTRAQ@N-term	2333.2209	778.7476	3	0.1700
<1	5		AEQQARVAELLQLVGLPGAAR	iTRAQ@N-term	2333.3300	778.7840	3	0.0608

Precursor MS Region

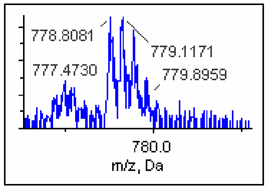

Fragmentation Evidence for Peptide

ITR TALATEAAALGVDIAVVAAGLHR

Residue	b	y
T	246.1570	2334.3262
A	317.1941	2089.1764
L	430.2782	2018.1393
A	501.3153	1905.0552
T	602.3630	1834.0181
E	731.4056	1732.9704
A	802.4427	1603.9279
A	873.4798	1532.8907
A	944.5169	1461.8536
L	1057.6010	1390.8165
G	1114.6224	1277.7324
V	1213.6909	1220.7110
D	1328.7178	1121.6426
I	1441.8019	1006.6156
A	1512.8390	893.5316
V	1611.9074	822.4944
V	1710.9758	723.4260
A	1782.0129	624.3576
A	1853.0500	553.3205
G	1910.0715	482.2834
L	2023.1556	425.2619
H	2160.2145	312.1779
R	2316.3156	175.1190

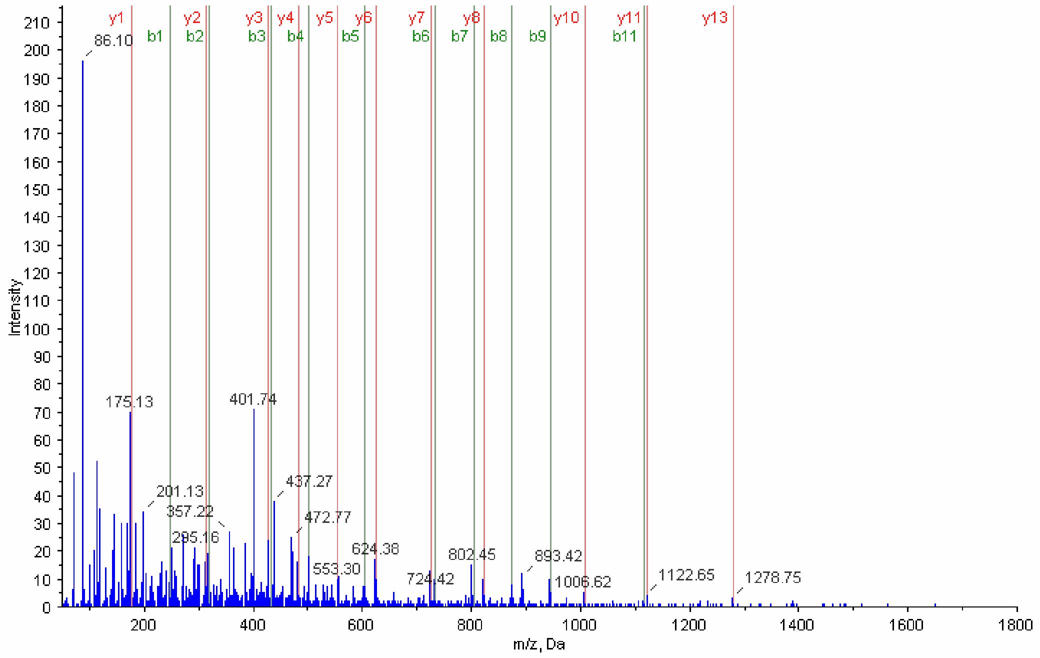

Protein: SCO2010 [1 of 1]

Protein Quant | Protein ID | Spectra | Summary Statistics

Spectrum List

Spectrum	Time	Prec MW	Prec m/z	Prec z	Best Sequence	Modifications	Conf	Theor MW	z	115:114	%Err 115:114	116:
42.1.1.967.4	33.472	2273.2246	758.7488	3	KLEFHEEDEAPAVLSK	iTRAQ@N-term, iTRAQ(K)@...	99	2273.2270	3	1.3661	28.32	

Peptide ID Hypotheses

Conf	Sc	Prot N	Sequence	Modifications	Theor MW	Theor m/z	z	ΔMass
99	12	781	KLEFHEEDEAPAVLSK	iTRAQ@N-term, iTRAQ(K)@...	2273.2270	758.7496	3	-0.0024
< 1	5		RAGHGMFMSPDEVLDIARK	No iTRAQ@N-term, iTRAQ(...	2273.1532	758.7250	3	0.0714
< 1	4		TDRLEALQGSGIVLTGVEK	iTRAQ@N-term, iTRAQ(K)@...	2273.2835	758.7684	3	-0.0589

Precursor MS Region

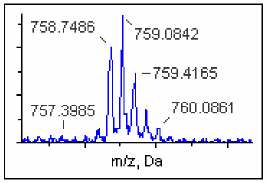

Fragmentation Evidence for Peptide

ITR K[ITR]LEFHEEDEAPAVLSK[ITR]

Res	b	y
K[I	417.3064	2274.2342
L	**530.3904**	1857.9351
E	659.4330	1744.8510
F	806.5014	1615.8084
H	**943.5603**	1468.7400
E	**1072.6029**	1331.6811
E	**1201.6455**	1202.6385
D	**1316.6725**	1073.5959
E	**1445.7151**	958.5690
A	1516.7522	829.5264
P	1613.8049	**758.4893**
A	1684.8421	**661.4365**
V	1783.9105	**590.3994**
L	1896.9945	**491.3310**
S	1984.0266	**378.2469**
K[I	2256.2236	**291.2149**

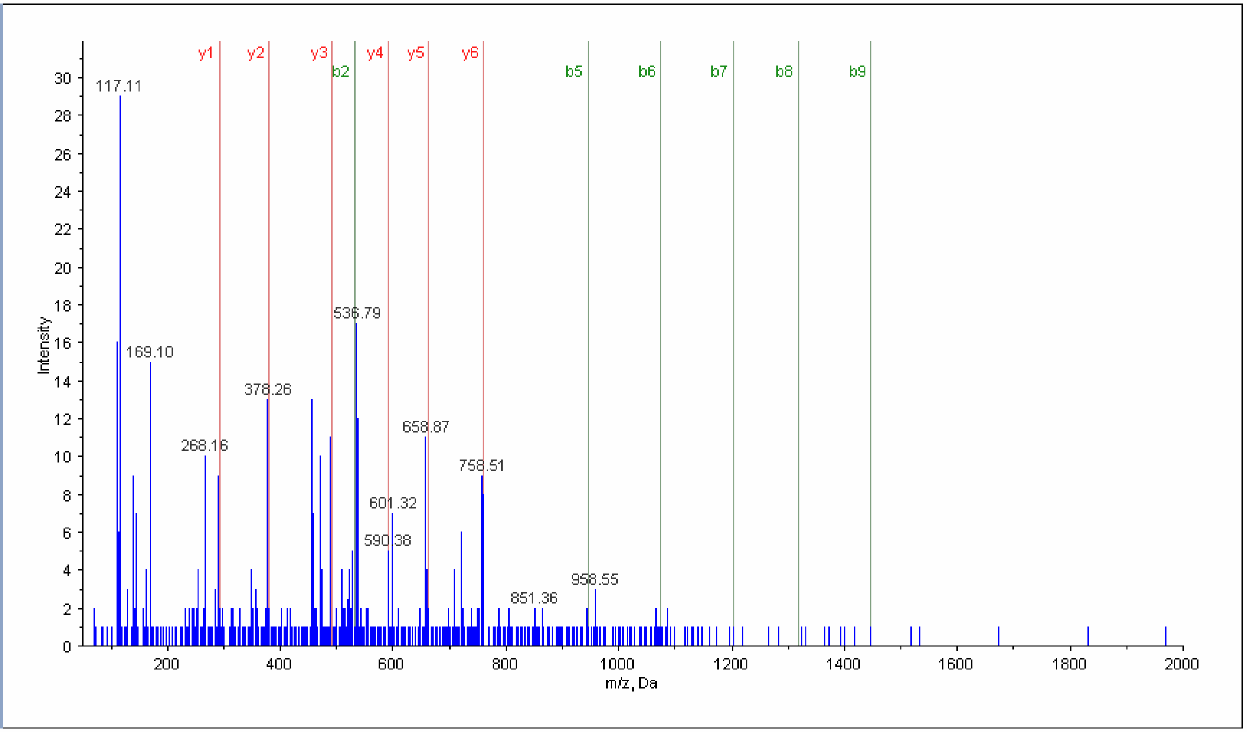

Protein: SCO2012, LivF [1 of 2]

Protein Quant | Protein ID | Spectra | Summary Statistics

Spectrum List

Spectrum	Time	Prec MW	Prec m/z	Prec z	Best Sequence	Modifications	Conf	Theor MW	z	115:114	%Err 115:114	116:
5.1.1.2108.3	45.366	1436.7739	719.3942	2	AYDLFPILGER	iTRAQ@N-term	99	1436.7786	2	1.5339	3.57	

Peptide ID Hypotheses

Conf	Sc	Prot N	Sequence	Modifications	Theor MW	Theor m/z	z	ΔMass
99	14	427	AYDLFPILGER	iTRAQ@N-term	1436.7786	719.3966	2	-0.0047
< 1	7		AADVTALTATGMR	iTRAQ@N-term, Oxidation(...	1436.7415	719.3781	2	0.0324
< 1	7		ATPDELLELHR	iTRAQ@N-term	1436.7746	719.3945	2	-0.0006
< 1	7		HPEVVEGAASAAR	iTRAQ@N-term	1436.7494	719.3820	2	0.0245
< 1	7		SESNLRLYRR	iTRAQ@N-term	1436.7970	719.4058	2	-0.0231

Precursor MS Region

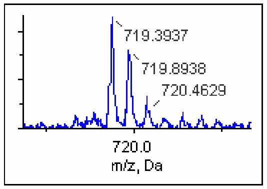

Fragmentation Evidence for Peptide

ITR AYDLFPILGER

Residue	b	y
A	216.1465	1437.7858
Y	379.2098	1222.6467
D	494.2367	1059.5833
L	607.3208	944.5564
F	754.3892	831.4723
P	851.4420	684.4039
I	964.5260	587.3511
L	1077.6101	474.2671
G	1134.6316	361.1830
E	1263.6742	304.1615
R	1419.7753	175.1190

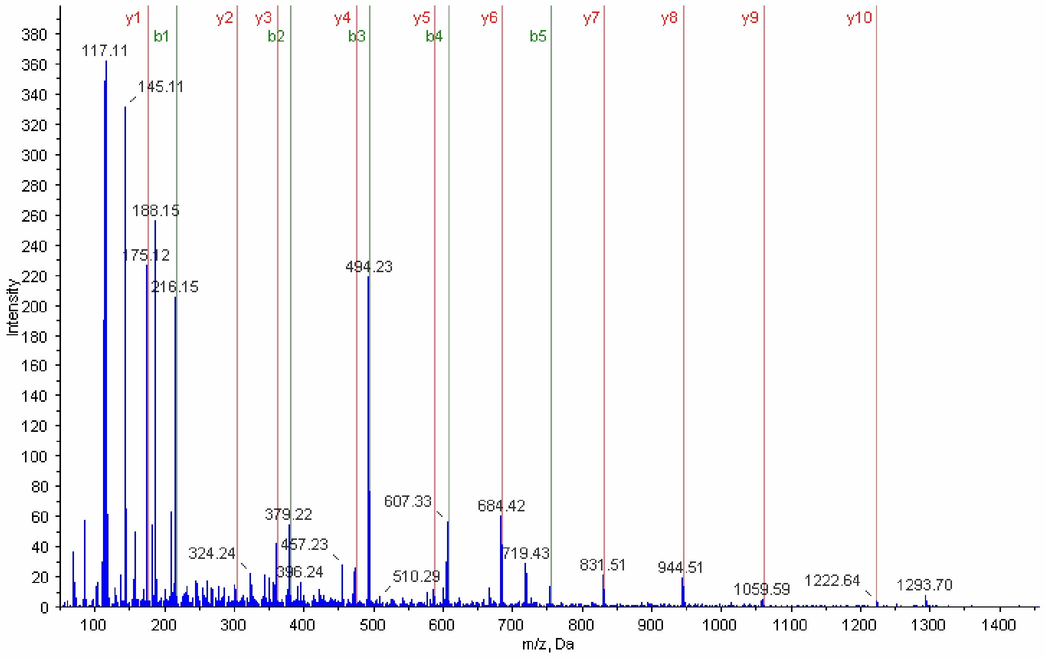

Protein: SCO2012, LivF [1 of 2]

Protein Quant | Protein ID | Spectra | Summary Statistics

Spectrum List

Spectrum	Time	Prec MW	Prec m/z	Prec z	Best Sequence	Modifications	Conf	Theor MW	z	115:114	%Err 115:114	116:
9.1.1.2080.3	45.112	1436.7689	719.3917	2	AYDLFPILGER	iTRAQ@N-term	99	1436.7786	2	1.5371	2.10	

Peptide ID Hypotheses

Conf	Sc	Prot N	Sequence	Modifications	Theor MW	Theor m/z	z	ΔMass
99	16	427	AYDLFPILGER	iTRAQ@N-term	1436.7786	719.3966	2	-0.0096
< 1	9		TIAAAAPLGIVVEGR	No iTRAQ@N-term	1436.8351	719.4248	2	-0.0662
< 1	7		AQTMDQLIAFR	iTRAQ@N-term	1436.7568	719.3857	2	0.0121
< 1	7		SESNLRLYRR	iTRAQ@N-term	1436.7970	719.4058	2	-0.0281
< 1	7		SGMDWIEVAFR	iTRAQ@N-term	1436.7170	719.3658	2	0.0519

Precursor MS Region

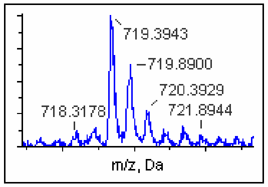

Fragmentation Evidence for Peptide

ITR AYDLFPILGER

Residue	b	y
A	216.1465	1437.7858
Y	379.2098	1222.6467
D	494.2367	1059.5833
L	607.3208	944.5564
F	754.3892	831.4723
P	851.4420	684.4039
I	964.5260	587.3511
L	1077.6101	474.2671
G	1134.6316	361.1830
E	1263.6742	304.1615
R	1419.7753	175.1190

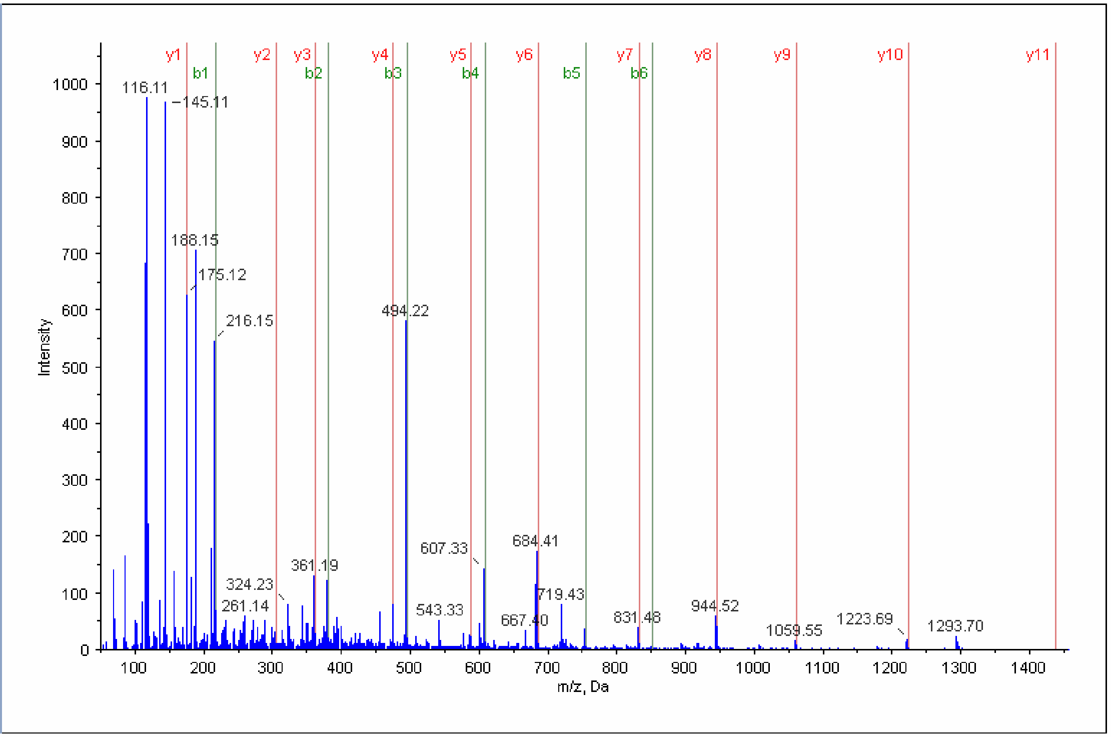

Protein: SCO2019 [1 of 1]

Protein Quant | Protein ID | Spectra | Summary Statistics

Spectrum List

Spectrum	Time	Prec MW	Prec m/z	Prec z	Best Sequence	Modifications	Conf	Theor MW	z	115:114	%Err 115:114	116:
9.1.1.934.2	25.498	1387.6829	694.8487	2	IAESNAAGGAPATD	iTRAQ@N-term	99	1387.6702	2	1.1772	16.97	

Peptide ID Hypotheses

Conf	Sc	Prot N	Sequence	Modifications	Theor MW	Theor m/z	z	ΔMass
99	16	960	IAESNAAGGAPATD	iTRAQ@N-term	1387.6702	694.8424	2	0.0127
< 1	8		GQAEEAAGRQAR	iTRAQ@N-term, Deamidatio...	1387.6927	694.8536	2	-0.0098
< 1	8		TGELDTLNDALAR	No iTRAQ@N-term	1387.6944	694.8545	2	-0.0115
< 1	7		FVRGDATDPAALR	No iTRAQ@N-term	1387.7209	694.8678	2	-0.0380
< 1	7		MTPNAASGPSRR	iTRAQ@N-term	1387.7113	694.8629	2	-0.0284

Precursor MS Region

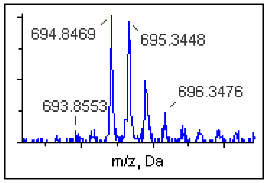

Fragmentation Evidence for Peptide

ITR IAESNAAGGAPATD

Res	b	y
I	258.1934	1388.6774
A	329.2305	1131.4913
E	458.2731	1060.4542
S	545.3051	931.4116
N	659.3481	844.3795
A	730.3852	730.3366
A	801.4223	659.2995
G	858.4438	588.2624
G	915.4652	531.2409
A	986.5023	474.2195
P	1083.5551	403.1823
A	1154.5922	306.1296
T	1255.6399	235.0925
D	1370.6668	134.0448

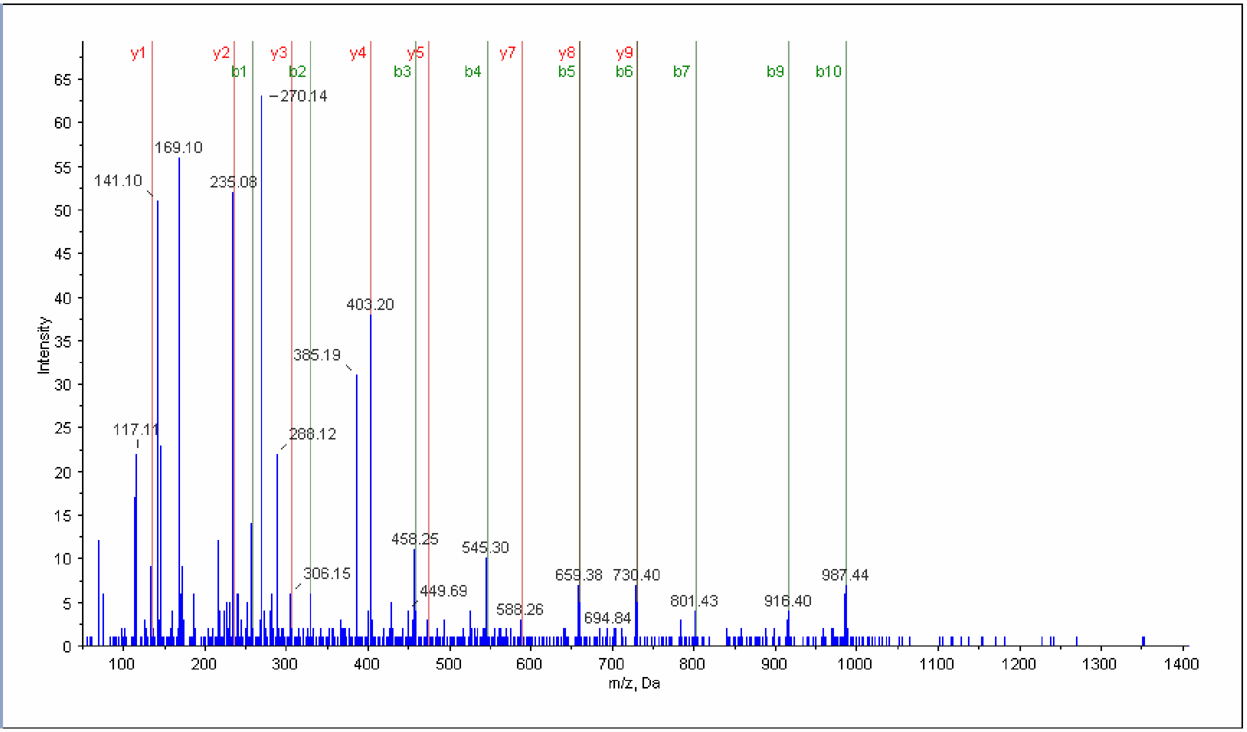

Protein: SCO2039, TrpC1 [1 of 1]

Protein Quant | Protein ID | Spectra | Summary Statistics

Spectrum List

Spectrum	Time	Prec MW	Prec m/z	Prec z	Best Sequence	Modifications	Conf	Theor MW	z	115:114	%Err 115:114	116:
50.1.1.1019.3	36.537	1749.9415	584.3211	3	TAVSDLVAAGEHPALR	iTRAQ@N-term	99	1749.9496	3	2.1929	23.40	

Peptide ID Hypotheses

Conf	Sc	Prot N	Sequence	Modifications	Theor MW	Theor m/z	z	ΔMass
99	16	850	TAVSDLVAAGEHPALR	iTRAQ@N-term	1749.9496	584.3238	3	-0.0081
< 1	7	12	TGEVLSVAVGEGYLGR	iTRAQ@N-term	1749.9384	584.3201	3	0.0031
< 1	11		EGLRNVAAGANPMALKR	PGA of E@N-term, Deamida...	1749.9309	584.3176	3	0.0106
< 1	7		LGSGGMGRVYLGRSAGGR	No iTRAQ@N-term	1749.9058	584.3092	3	0.0357
< 1	7		RSIEEAAFDNIWR	iTRAQ@N-term	1749.8921	584.3046	3	0.0494

Precursor MS Region

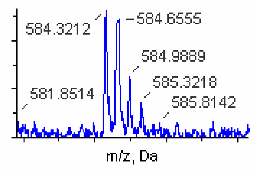

Fragmentation Evidence for Peptide

ITR TAVSDLVAAGEHPALR

Res	b	y
T	246.1570	1750.9568
A	317.1941	1505.8071
V	416.2625	1434.7700
S	503.2946	1335.7015
D	618.3215	1248.6695
L	731.4056	1133.6426
V	830.4740	1020.5585
A	901.5111	921.4901
A	972.5482	850.4530
G	1029.5697	779.4159
E	1158.6123	722.3944
H	1295.6712	593.3518
P	1392.7240	456.2929
A	1463.7611	359.2401
L	1576.8451	288.2030
R	1732.9462	175.1190

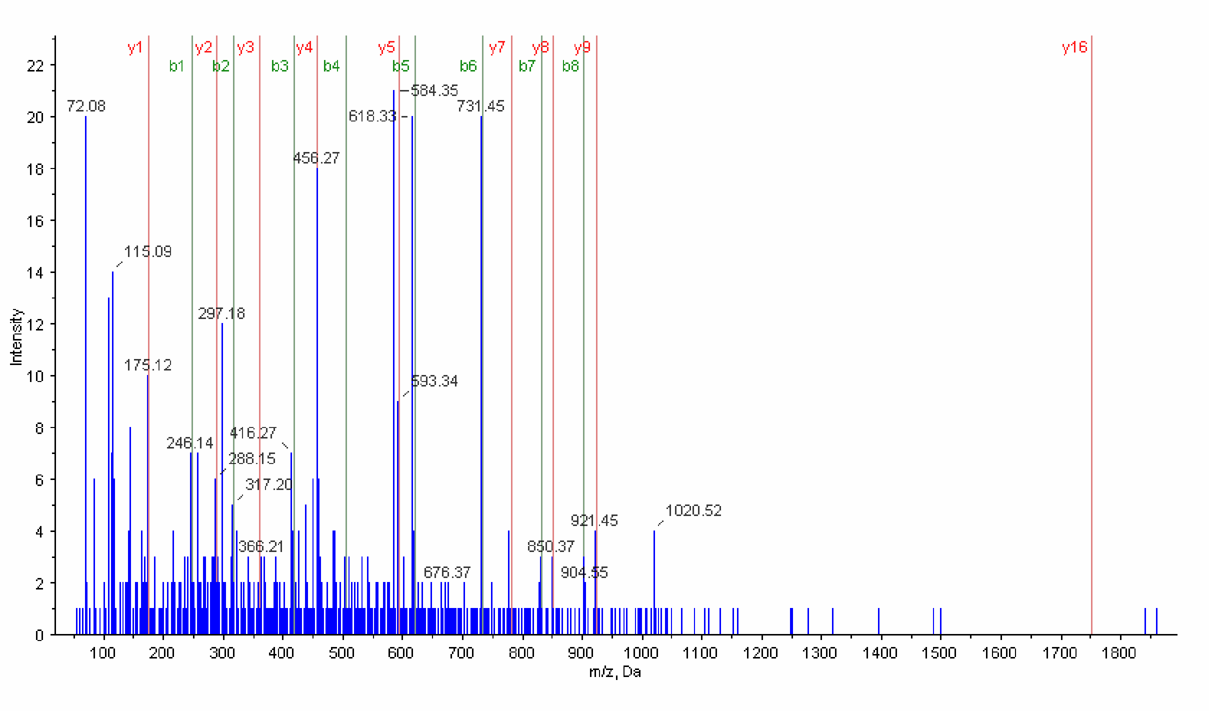

Protein: SCO2043, TrpE3 [1 of 1]

Protein Quant | Protein ID | Spectra | Summary Statistics

Spectrum List

Spectrum	Time	Prec MW	Prec m/z	Prec z	Best Sequence	Modifications	Conf	Theor MW	z	115:114	%Err 115:114	116:
33.1.1.976.4	37.778	1738.7751	580.5990	3	MDVTHDMDLDTFR	iTRAQ@N-term	99	1738.7776	3	1.1680	13.74	

Peptide ID Hypotheses

Conf	Sc	Prot N	Sequence	Modifications	Theor MW	Theor m/z	z	ΔMass
99	15	695	MDVTHDMDLDTFR	iTRAQ@N-term	1738.7776	580.5998	3	-0.0025
< 1	6		LAYDAGQAIDARNCGR	No iTRAQ@N-term, MMTS(...	1738.7879	580.6033	3	-0.0128
< 1	5		NVGTSTGPDGAHYYR	iTRAQ@N-term, Deamidatio...	1738.8033	580.6084	3	-0.0281

Precursor MS Region

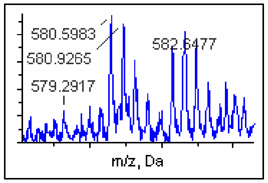

Fragmentation Evidence for Peptide

ITR MDVTHDMDLDTFR

Res	b	y
M	276.1498	1739.7849
D	391.1768	1464.6424
V	490.2452	1349.6154
T	591.2929	1250.5470
H	728.3518	1149.4993
D	843.3787	1012.4404
M	974.4192	897.4135
D	1089.4461	766.3730
L	1202.5302	651.3461
D	1317.5571	538.2620
T	1418.6048	423.2350
F	1565.6732	322.1874
R	1721.7744	175.1190

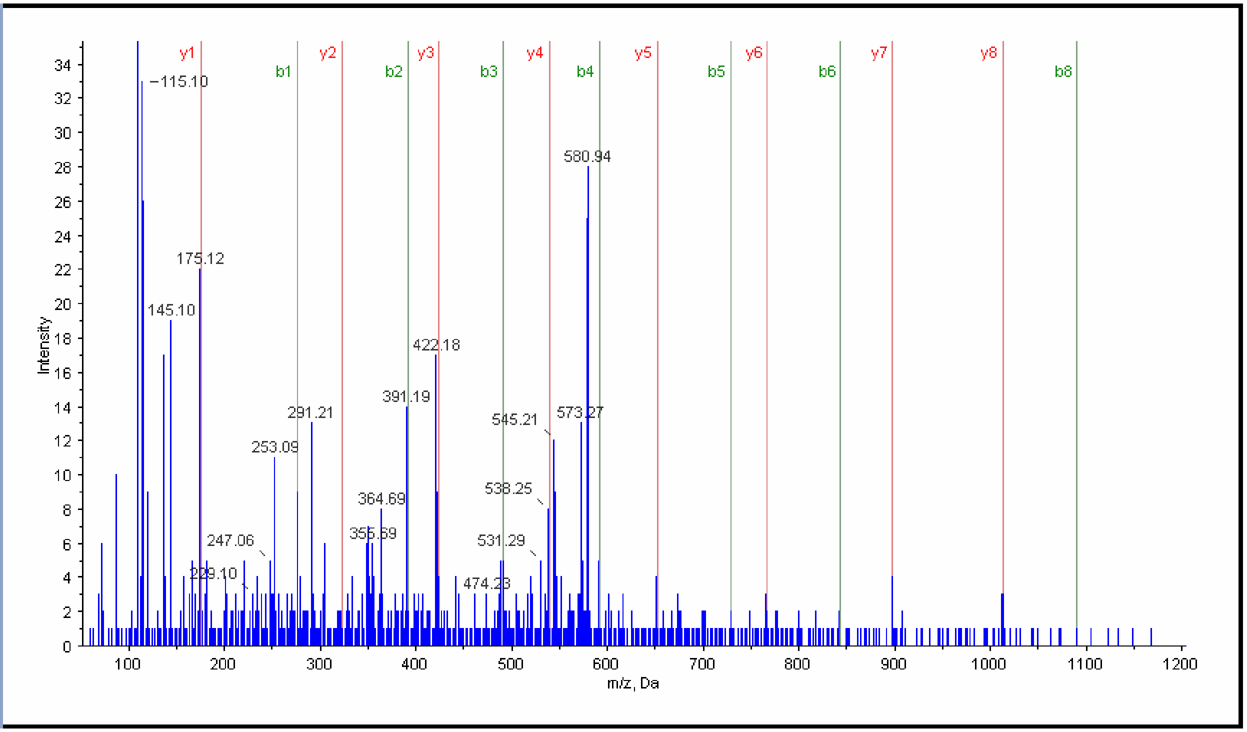

Protein: SCO2052, HisB [1 of 1]

Protein Quant | Protein ID | Spectra | Summary Statistics

Spectrum List

Spectrum	Time	Prec MW	Prec m/z	Prec z	Best Sequence	Modifications	Conf	Theor MW	z	115:114	%Err 115:114	116:
31.1.1.1076.3	49.147	2252.1089	751.7103	3	TDIATGVGFYDHMLDQLGR	iTRAQ@N-term	99	2252.1016	3	1.0629	16.70	

Peptide ID Hypotheses

Conf	Sc	Prot N	Sequence	Modifications	Theor MW	Theor m/z	z	ΔMass
99	15	926	TDIATGVGFYDHMLDQLGR	iTRAQ@N-term	2252.1016	751.7078	3	0.0073
< 1	6		LDLLDDERTVAENFAAHAPR	No iTRAQ@N-term	2252.1184	751.7134	3	-0.0096
< 1	6		TAASEQGLRADEVVVVHNSDR	No iTRAQ@N-term	2252.1144	751.7121	3	-0.0055
< 1	5		LSDGRDPMIREVDTEYGR	iTRAQ@N-term	2252.0976	751.7065	3	0.0113
< 1	5		SPSETTNVLEGRTGAACTLR	iTRAQ@N-term, MMTS(C)@...	2252.1010	751.7076	3	0.0079

Precursor MS Region

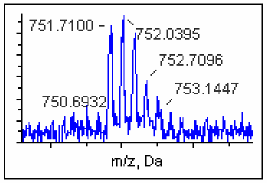

Fragmentation Evidence for Peptide

ITR TDIATGVGFYDHMLDQLGR

Res	b	y
T	**246.1570**	2253.1090
D	**361.1840**	2007.9593
I	**474.2680**	1892.9323
A	**545.3051**	1779.8483
T	**646.3528**	1708.8112
G	**703.3743**	1607.7635
V	802.4427	1550.7420
G	**859.4642**	1451.6736
F	1006.5326	1394.6521
Y	1169.5959	1247.5837
D	1284.6228	**1084.5204**
H	1421.6818	**969.4935**
M	1552.7222	832.4345
L	1665.8063	**701.3941**
D	1780.8332	**588.3100**
Q	1908.8918	**473.2831**
L	2021.9759	**345.2245**
G	2078.9974	**232.1404**
R	2235.0985	**175.1190**

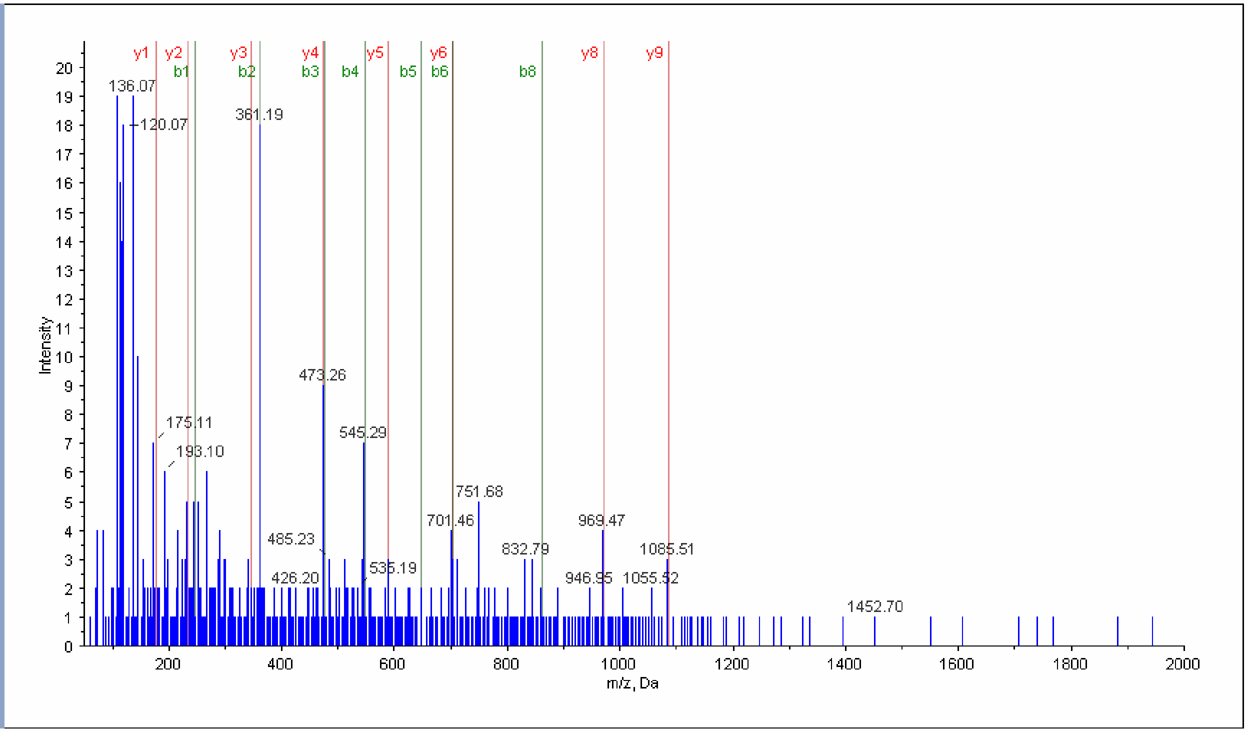

Protein: SCO2148, QcrB [1 of 1]

Protein Quant | Protein ID | Spectra | Summary Statistics

Spectrum List

Spectrum	Time	Prec MW	Prec m/z	Prec z	Best Sequence	Modifications	Conf	Theor MW	z	115:114	%Err 115:114	116:
29.1.1.1048.2	36.565	1151.7168	576.8657	2	LGIYSLAK	iTRAQ@N-term, iTRAQ(K)@8	99	1151.7158	2	0.7467	5.12	

Peptide ID Hypotheses

Conf	Sc	Prot N	Sequence	Modifications	Theor MW	Theor m/z	z	ΔMass
99	12	851	LGIYSLAK	iTRAQ@N-term, iTRAQ(K)@8	1151.7158	576.8652	2	0.0010
< 1	6	4	ITYRLAK	iTRAQ@N-term, iTRAQ(K)@7	1151.7270	576.8708	2	-0.0102
< 1	6		IGTFVEAK	iTRAQ@N-term, iTRAQ(K)@8	1151.6794	576.8470	2	0.0374
< 1	5		IGNTLHVVR	iTRAQ@N-term	1151.6897	576.8521	2	0.0271
< 1	5		LLEILHDR	iTRAQ@N-term	1151.6785	576.8465	2	0.0383

Precursor MS Region

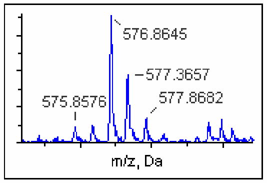

Fragmentation Evidence for Peptide

ITR LGIYSLAK[ITR]

Res	b	y
L	258.1934	1152.7231
G	315.2149	895.5369
I	428.2989	838.5155
Y	591.3623	725.4314
S	678.3943	562.3681
L	791.4784	475.3360
A	862.5155	362.2520
K[I	1134.7125	291.2149

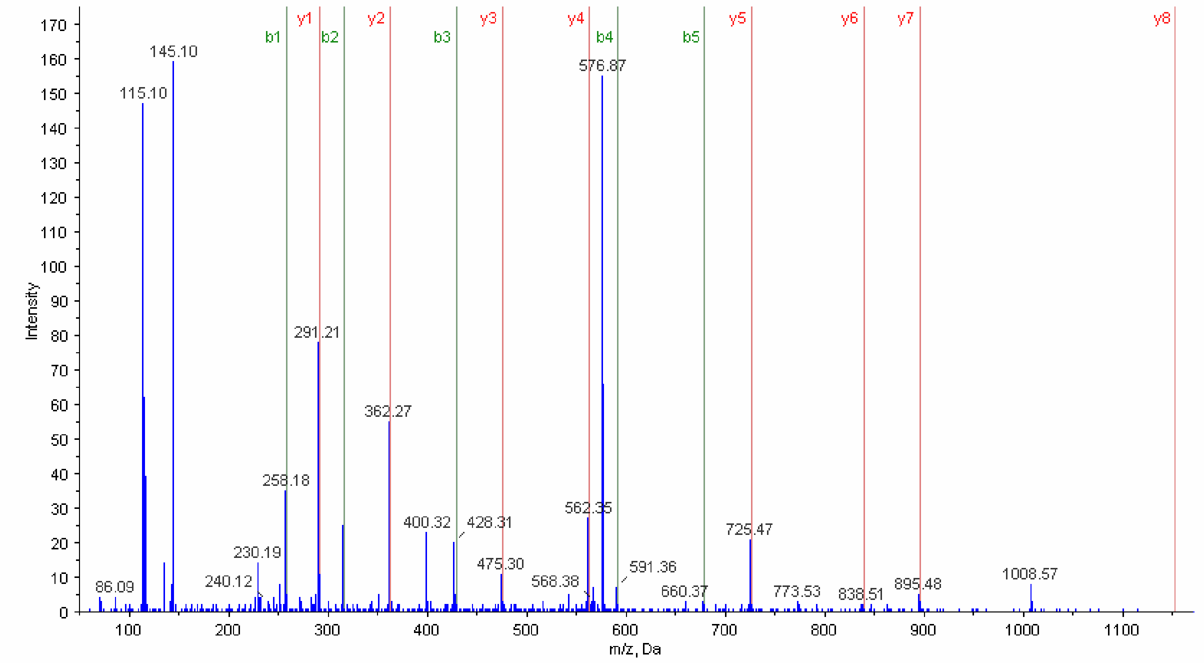

Protein: SCO2585, ProA [1 of 3]

Protein Quant | Protein ID | Spectra | Summary Statistics

Spectrum List

Spectrum	Time	Prec MW	Prec m/z	Prec z	Best Sequence	Modifications	Conf	Theor MW	z	115:114	%Err 115:114	116:
17.1.1.1232.2	45.778	1621.9125	541.6448	3	DVVALPDPVGEIVR	iTRAQ@N-term	99	1621.9160	3			

Peptide ID Hypotheses

Conf	Sc	Prot N	Sequence	Modifications	Theor MW	Theor m/z	z	ΔMass
99	13	852	DVVALPDPVGEIVR	iTRAQ@N-term	1621.9160	541.6459	3	-0.0036
< 1	6	89	LEGLAASPGRIVGRAR	No iTRAQ@N-term	1621.9376	541.6531	3	-0.0251
< 1	7		ADLSQIVKAYDVR	No iTRAQ@N-term, Deamid...	1621.8797	541.6338	3	0.0328
< 1	7		ALGRVTPDGNVRLVR	No iTRAQ@N-term	1621.9376	541.6531	3	-0.0251
< 1	7		DVADPKAYRR	iTRAQ@N-term, iTRAQ(K)@...	1621.9265	541.6494	3	-0.0140

Precursor MS Region

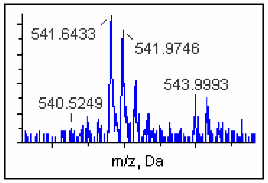

Fragmentation Evidence for Peptide

ITR DVVALPDPVGEIVR

Res	b	y
D	260.1363	1622.9234
V	359.2047	1363.7944
V	458.2731	1264.7260
A	529.3102	1165.6575
L	642.3943	1094.6204
P	739.4471	981.5364
D	854.4740	884.4836
P	951.5268	769.4567
V	1050.5952	672.4039
G	1107.6166	573.3355
E	1236.6592	516.3140
I	1349.7433	387.2714
V	1448.8117	274.1874
R	1604.9128	175.1190

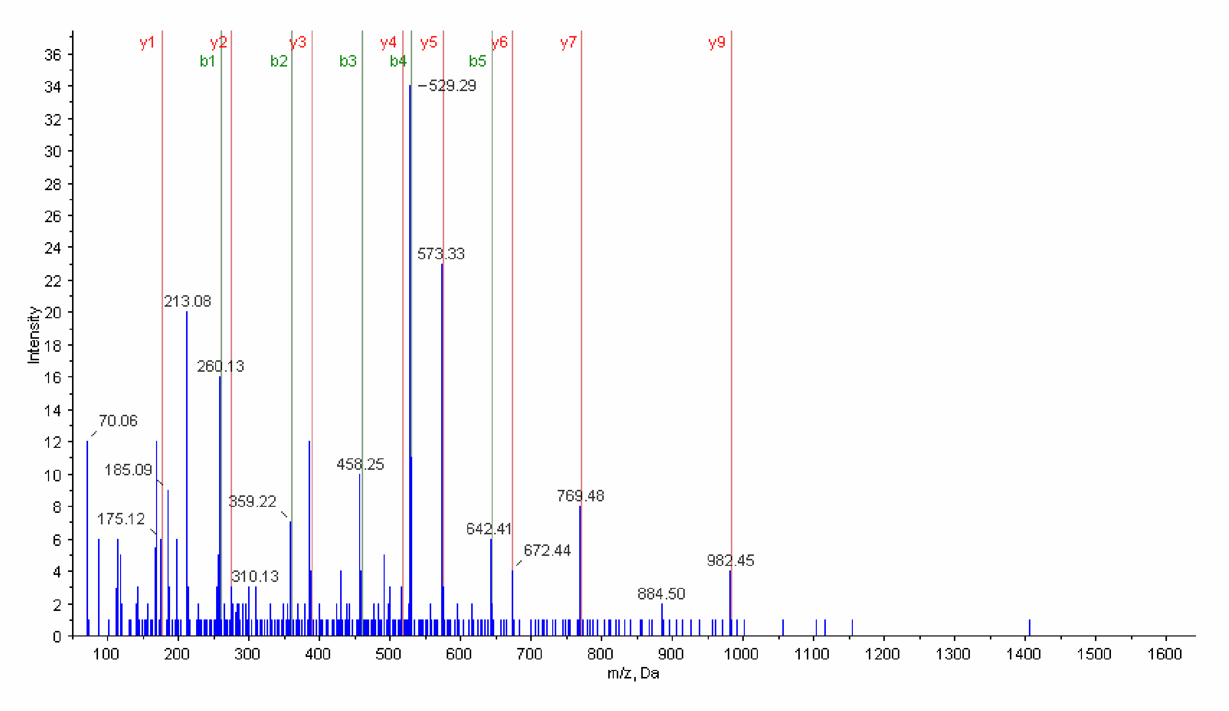

Protein: SCO2585, ProA [2 of 3]

Protein Quant | Protein ID | Spectra | Summary Statistics

Spectrum List

Spectrum	Time	Prec MW	Prec m/z	Prec z	Best Sequence	Modifications	Conf	Theor MW	z	115:114	%Err 115:114	116:
19.1.1.1350.3	45.734	1621.9303	811.9724	2	DVVALPDPVGEIVR	iTRAQ@N-term	99	1621.9162	2	0.9858	19.33	

Peptide ID Hypotheses

Conf	Sc	Prot N	Sequence	Modifications	Theor MW	Theor m/z	z	ΔMass
99	12	852	DVVALPDPVGEIVR	iTRAQ@N-term	1621.9162	811.9653	2	0.0141
< 1	5	89	LEGLAASPGRIVGRAR	No iTRAQ@N-term	1621.9377	811.9761	2	-0.0074
< 1	8		DVADPKAYRR	iTRAQ@N-term, iTRAQ(K)@...	1621.9266	811.9706	2	0.0037
< 1	8		LTAVSRHRLNRR	iTRAQ@N-term	1621.9723	811.9934	2	-0.0420
< 1	7		ALGRVTPDGNVRLVR	No iTRAQ@N-term	1621.9377	811.9761	2	-0.0074

Precursor MS Region

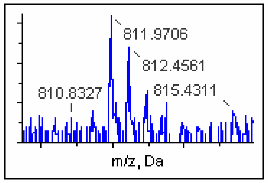

Fragmentation Evidence for Peptide

ITR DVVALPDPVGEIVR

Res	b	y
D	260.1363	1622.9234
V	359.2047	1363.7944
V	458.2731	1264.7260
A	529.3102	1165.6575
L	642.3943	1094.6204
P	739.4471	981.5364
D	854.4740	884.4836
P	951.5268	769.4567
V	1050.5952	672.4039
G	1107.6166	573.3355
E	1236.6592	516.3140
I	1349.7433	387.2714
V	1448.8117	274.1874
R	1604.9128	175.1190

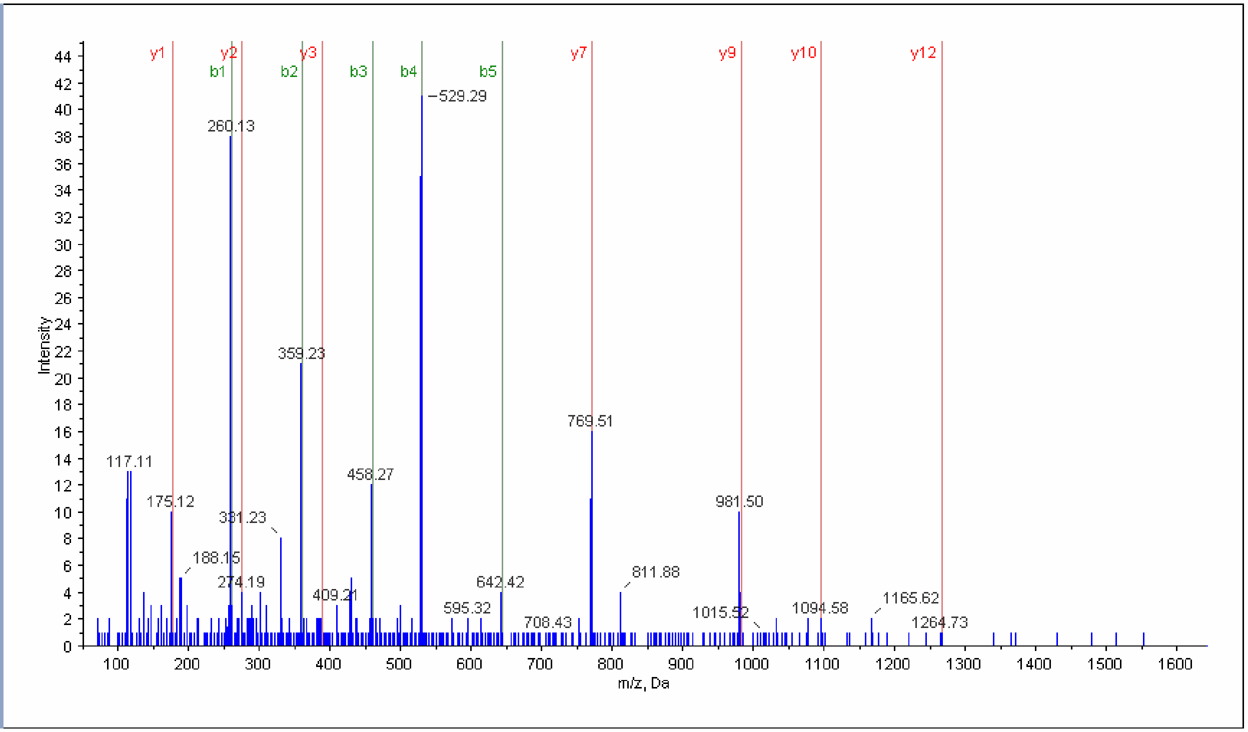

Protein: SCO2585, ProA [3 of 3]

Protein Quant | Protein ID | Spectra | Summary Statistics

Spectrum List

Spectrum	Time	Prec MW	Prec m/z	Prec z	Best Sequence	Modifications	Conf	Theor MW	z	115:114	%Err 115:114	116:
15.1.1.1115.4	45.602	1621.9208	541.6475	3	DVVALPDPVGEIVR	iTRAQ@N-term	99	1621.9162	3			

Peptide ID Hypotheses

Conf	Sc	Prot N	Sequence	Modifications	Theor MW	Theor m/z	z	ΔMass
99	13	852	DVVALPDPVGEIVR	iTRAQ@N-term	1621.9162	541.6460	3	0.0046
<1	6	89	LEGLAASPGRIVGRAR	No iTRAQ@N-term	1621.9377	541.6532	3	-0.0169
<1	8		LEQLAEHDPRVR	iTRAQ@N-term, Oxidation(H...	1621.8659	541.6292	3	0.0549
<1	7		DVADPKAYRR	iTRAQ@N-term, iTRAQ(K)@...	1621.9266	541.6495	3	-0.0059
<1	6		ALGRVTPDGNVRLVR	No iTRAQ@N-term	1621.9377	541.6532	3	-0.0169

Precursor MS Region

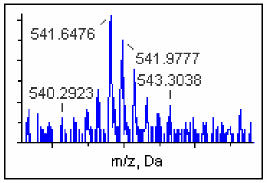

Fragmentation Evidence for Peptide

ITR | DVVALPDPVGEIVR

Res	b	y
D	**260.1363**	1622.9234
V	**359.2047**	1363.7944
V	**458.2731**	1264.7260
A	**529.3102**	1165.6575
L	**642.3943**	1094.6204
P	739.4471	**981.5364**
D	854.4740	884.4836
P	951.5268	**769.4567**
V	1050.5952	**672.4039**
G	1107.6166	**573.3355**
E	1236.6592	**516.3140**
I	1349.7433	**387.2714**
V	1448.8117	**274.1874**
R	1604.9128	**175.1190**

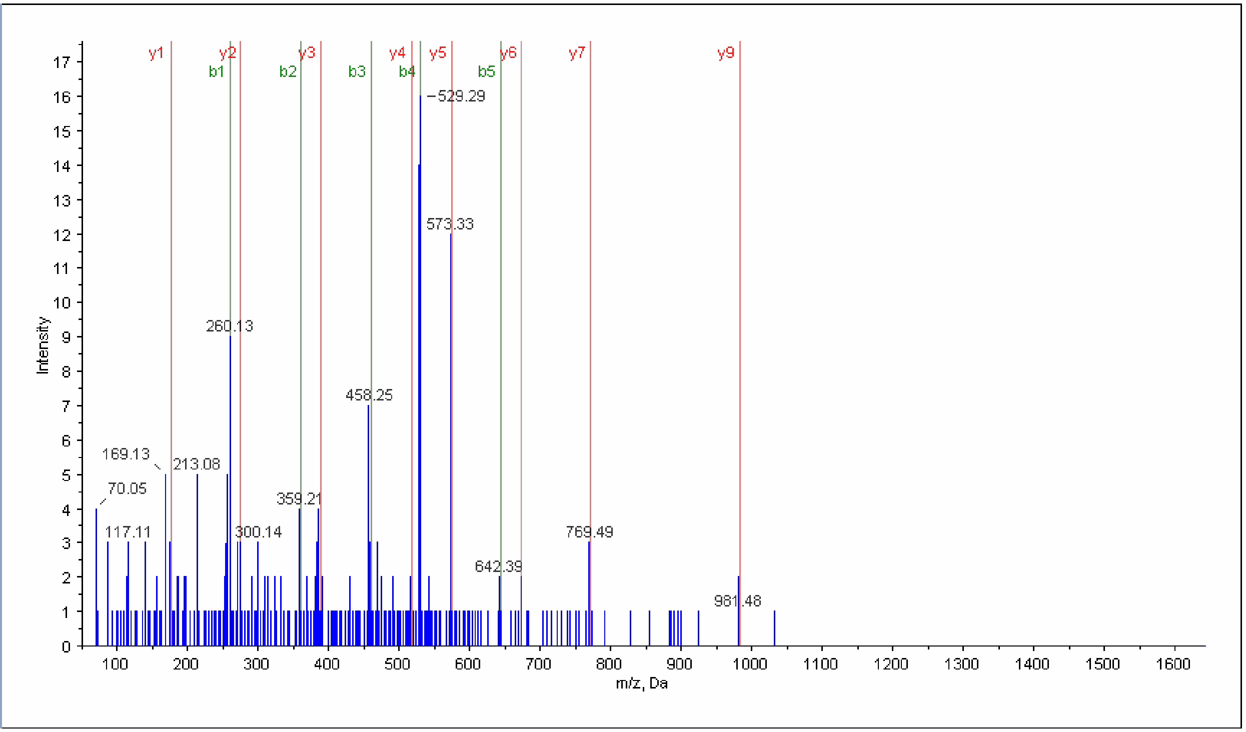

Protein: SCO2633, SodF [1 of 2]

Protein Quant | Protein ID | **Spectra** | Summary Statistics

Spectrum List

Spectrum	Time	Prec MW	Prec m/z	Prec z	Best Sequence	Modifications	Conf	Theor MW	z	115:114	%Err 115:114	116:
69.1.1.2408.3	44.607	1909.0337	955.5241	2	DKETWGSINGLEK	iTRAQ@N-term, iTRAQ(K)@...	99	1909.0159	2	1.1520	1.93	

Peptide ID Hypotheses

Conf	Sc	Prot N	Sequence	Modifications	Theor MW	Theor m/z	z	ΔMass
99	20	472	DKETWGSINGLEK	iTRAQ@N-term, iTRAQ(K)@...	1909.0159	955.5152	2	0.0178
< 1	17	226	EKESWGSINGLEK	iTRAQ@N-term, iTRAQ(K)@...	1909.0159	955.5152	2	0.0178
< 1	12		LRNTHVPEATSLSGRK	No iTRAQ@N-term, iTRAQ(...	1909.0616	955.5380	2	-0.0279
< 1	10		ADDLTDASKDGLEK	iTRAQ@N-term, iTRAQ(K)@...	1909.0006	955.5076	2	0.0331
< 1	9		ATASNVEIAYTPTNSAR	iTRAQ@N-term	1908.9663	955.4904	2	0.0674

Precursor MS Region

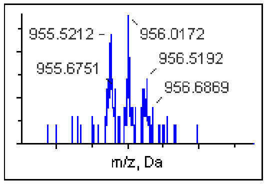

Fragmentation Evidence for Peptide

ITR DK[ITR]ETWGSIN[Dea]GLEK[ITR]

Residue	b	y
D	260.1363	1910.0231
K[ITR]	532.3333	1650.8941
E	661.3759	1378.6971
T	762.4236	1249.6545
W	948.5029	1148.6068
G	1005.5244	962.5275
S	1092.5564	905.5060
I	1205.6404	818.4740
N[Dea]	1320.6674	705.3899
G	1377.6889	590.3630
L	1490.7729	533.3415
E	1619.8155	420.2575
K[ITR]	1892.0125	291.2149

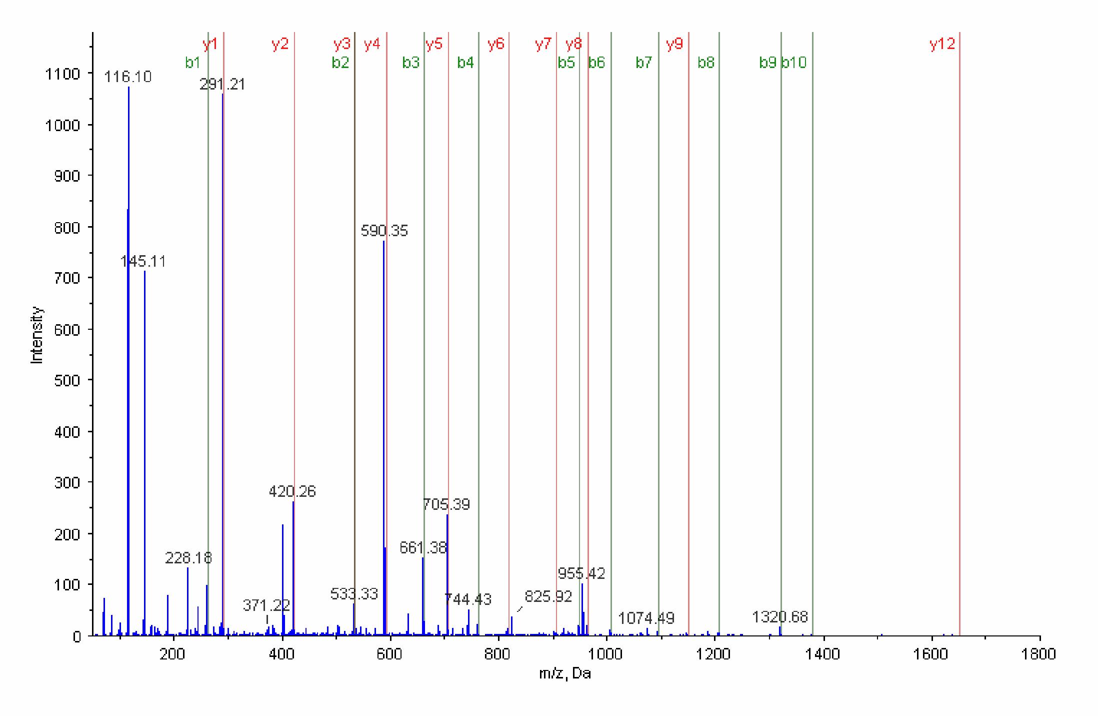

Protein: SCO2633, SodF [2 of 2]

Protein Quant | Protein ID | Spectra | Summary Statistics

Spectrum List

Spectrum	Time	Prec MW	Prec m/z	Prec z	Best Sequence	Modifications	Conf	Theor MW	z	115:114	%Err 115:114	116:
68.1.1.2309.2	44.885	1908.9968	637.3395	3	DKETWGSINGLEK	iTRAQ@N-term, iTRAQ(K)@...	98	1908.0318	3	0.9605	2.59	

Peptide ID Hypotheses

Conf	Sc	Prot N	Sequence	Modifications	Theor MW	Theor m/z	z	ΔMass
98	14	472	DKETWGSINGLEK	iTRAQ@N-term, iTRAQ(K)@...	1908.0318	637.0179	3	0.9650
< 1	11	226	EKESWGSINGLEK	iTRAQ@N-term, iTRAQ(K)@...	1909.0159	637.3459	3	-0.0190
< 1	8		ADDLTDASKDGLEK	iTRAQ@N-term, iTRAQ(K)@...	1909.0006	637.3408	3	-0.0038
< 1	8		ATGQEVSPCLVTLSASR	iTRAQ@N-term, Deamidatio...	1908.9407	637.3208	3	0.0561
< 1	8		GTDCVVALVEHHGRAR	iTRAQ@N-term, MMTS(C)@4	1908.9533	637.3250	3	0.0435

Precursor MS Region

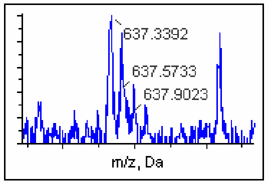

Fragmentation Evidence for Peptide

ITR DK[ITR]ETWGSINGLEK[ITR]

Residue	b	y
D	260.1363	1909.0391
K[ITR]	532.3333	1649.9101
E	661.3759	1377.7131
T	762.4236	1248.6705
W	948.5029	1147.6228
G	1005.5244	961.5435
S	1092.5564	904.5220
I	1205.6404	817.4900
N	1319.6834	704.4059
G	1376.7048	590.3630
L	1489.7889	533.3415
E	1618.8315	420.2575
K[ITR]	1891.0285	291.2149

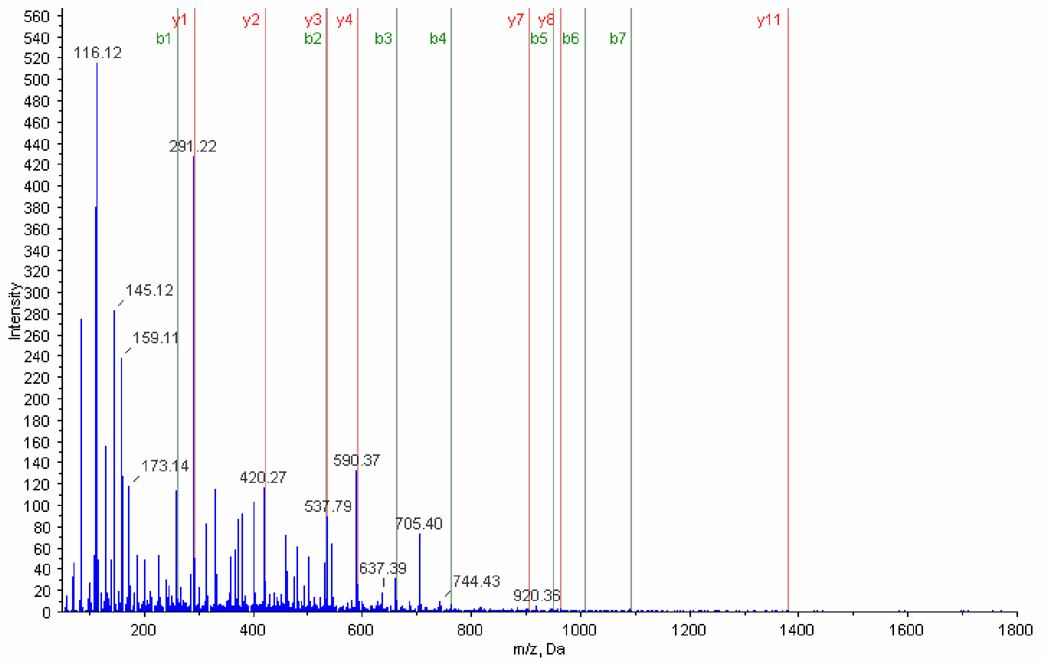

Protein: SCO3073, HutU [1 of 1]

Protein Quant | Protein ID | Spectra | Summary Statistics

Spectrum List

Spectrum	Time	Prec MW	Prec m/z	Prec z	Best Sequence	Modifications	Conf	Theor MW	z	115:114	%Err 115:114	116:
32.1.1.994.3	38.553	1753.9083	585.6434	3	ADSLDHALQLATEAR	iTRAQ@N-term	99	1753.9080	3	0.6102	15.78	

Peptide ID Hypotheses

Conf	Sc	Prot N	Sequence	Modifications	Theor MW	Theor m/z	z	ΔMass
99	17	950	ADSLDHALQLATEAR	iTRAQ@N-term	1753.9080	585.6433	3	0.0003
< 1	9		GAALAAGLSAQLAREEAR	No iTRAQ@N-term	1753.9435	585.6551	3	-0.0352
< 1	8		DWPGAVLAERLDVSAR	No iTRAQ@N-term	1753.9111	585.6443	3	-0.0028
< 1	8		LNGLGNIRALNAAVSDR	No iTRAQ@N-term, Deamid...	1753.9435	585.6551	3	-0.0352
< 1	8		LPPWSDSAAGLDRLTR	No iTRAQ@N-term	1753.9111	585.6443	3	-0.0028

Precursor MS Region

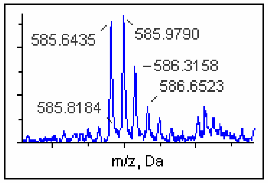

Fragmentation Evidence for Peptide

ITR ADSLDHALQLATEAR

Res	b	y
A	216.1465	1754.9153
D	331.1734	1539.7762
S	418.2054	1424.7492
L	531.2895	1337.7172
D	646.3164	1224.6331
H	783.3753	1109.6062
A	854.4125	972.5473
L	967.4965	901.5102
Q	1095.5551	788.4261
L	1208.6392	660.3675
A	1279.6763	547.2835
T	1380.7240	476.2463
E	1509.7665	375.1987
A	1580.8037	246.1561
R	1736.9048	175.1190

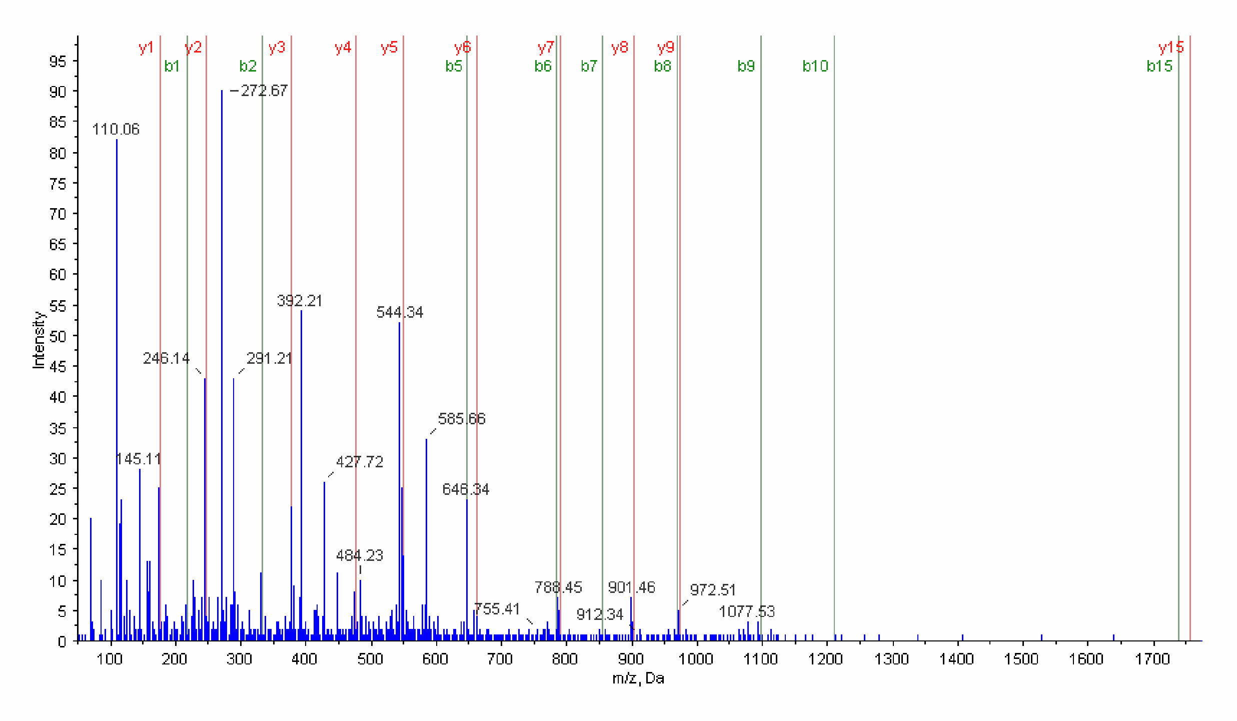

Protein: SCO3077 [1 of 2]

Protein Quant | Protein ID | Spectra | Summary Statistics

Spectrum List

Spectrum	Time	Prec MW	Prec m/z	Prec z	Best Sequence	Modifications	Conf	Theor MW	z	115:114	%Err 115:114	116:
25.1.1.1063.4	47.763	1306.7749	654.3947	2	ELLDALFAK	iTRAQ@N-term, iTRAQ(K)@9	99	1306.7740	2	1.0626	8.83	

Peptide ID Hypotheses

Conf	Sc	Prot N	Sequence	Modifications	Theor MW	Theor m/z	z	ΔMass
99	14	611	ELLDALFAK	iTRAQ@N-term, iTRAQ(K)@9	1306.7740	654.3943	2	0.0009
< 1	9		AGVSRALFAK	iTRAQ@N-term, iTRAQ(K)@...	1306.7965	654.4055	2	-0.0216
< 1	7		AGPHTAELIVR	iTRAQ@N-term	1306.7479	654.3812	2	0.0270
< 1	6		LSVIVPFYNVR	No iTRAQ@N-term, Deamid...	1306.7285	654.3715	2	0.0464
< 1	6		QLPHLRGWR	iTRAQ@N-term, Deamidatio...	1306.7380	654.3763	2	0.0369

Precursor MS Region

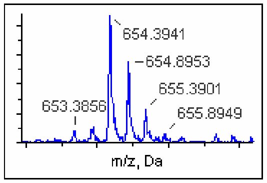

Fragmentation Evidence for Peptide

ITR ELLDALFAK[ITR]

Res	b	y
E	274.1519	1307.7813
L	387.2360	1034.6366
L	500.3201	921.5526
D	615.3470	808.4685
A	686.3841	693.4416
L	799.4682	622.4045
F	946.5366	509.3204
A	1017.5737	362.2520
K[I	1289.7707	291.2149

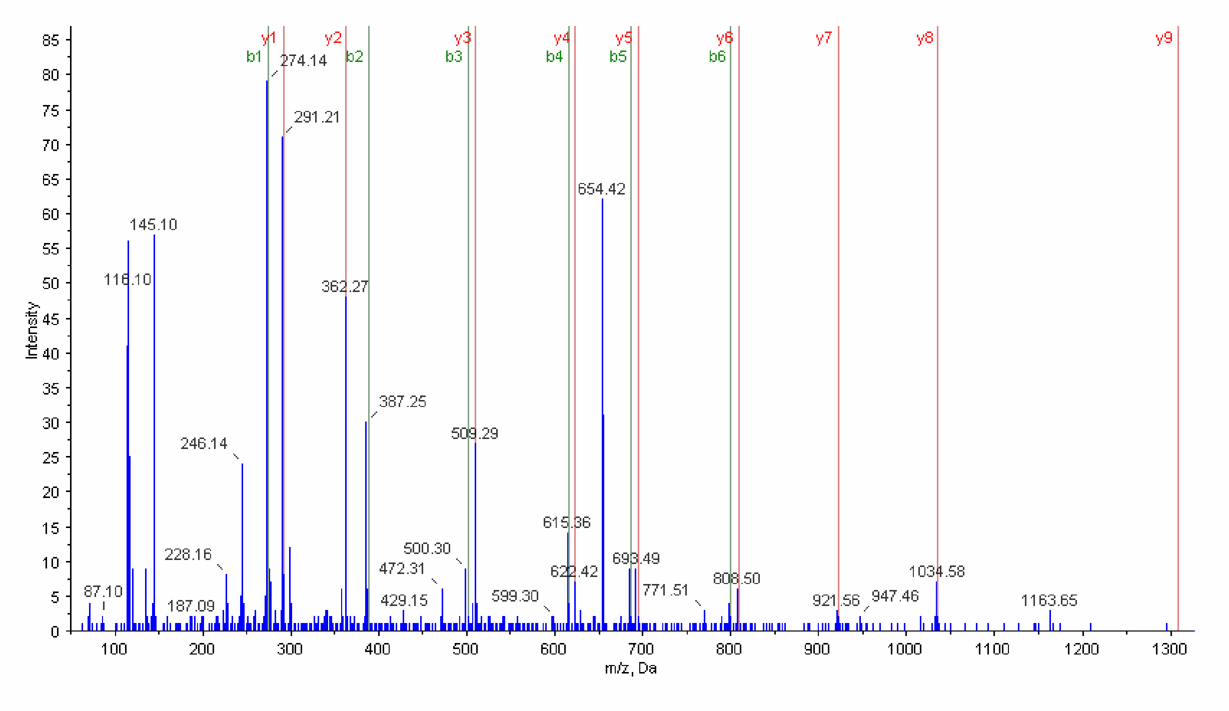

Protein: SCO3077 [2 of 2]

Protein Quant | Protein ID | Spectra | Summary Statistics

Spectrum List

Spectrum	Time	Prec MW	Prec m/z	Prec z	Best Sequence	Modifications	Conf	Theor MW	z	115:114	%Err 115:114	116:
26.1.1.1054.4	48.154	1306.7770	654.3957	2	ELLDALFAK	iTRAQ@N-term, iTRAQ(K)@9	99	1306.7741	2	1.2922	7.59	

Peptide ID Hypotheses

Conf	Sc	Prot N	Sequence	Modifications	Theor MW	Theor m/z	z	ΔMass
99	14	611	ELLDALFAK	iTRAQ@N-term, iTRAQ(K)@9	1306.7741	654.3943	2	0.0029
< 1	10		AGVSRALFAK	iTRAQ@N-term, iTRAQ(K)@ ...	1306.7966	654.4055	2	-0.0196
< 1	9		AGPHTAELIVR	iTRAQ@N-term	1306.7480	654.3812	2	0.0290
< 1	6		QLPHLRGWR	iTRAQ@N-term, Deamidatio ...	1306.7381	654.3763	2	0.0389
< 1	6		QRLEIARALVR	PGA of Q@N-term	1306.7834	654.3990	2	-0.0064

Precursor MS Region

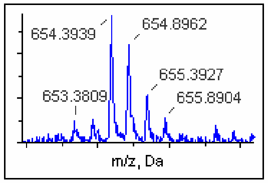

Fragmentation Evidence for Peptide

ITR ELLDALFAK[ITR]

Res	b	y
E	274.1519	1307.7813
L	387.2360	1034.6366
L	500.3201	921.5526
D	615.3470	808.4685
A	686.3841	693.4416
L	799.4682	622.4045
F	946.5366	509.3204
A	1017.5737	362.2520
K[I	1289.7707	291.2149

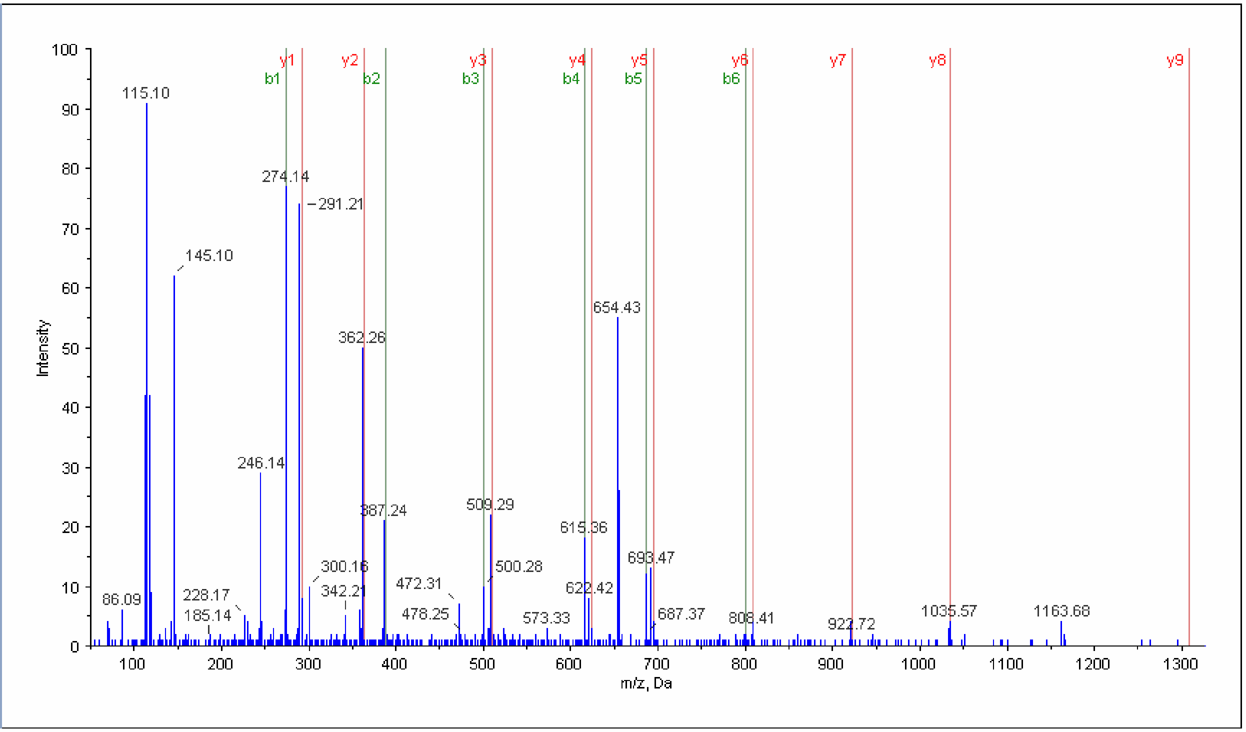

Protein: SCO3405, HprT [1 of 1]

Protein Quant | Protein ID | Spectra | Summary Statistics

Spectrum List

Spectrum	Time	Prec MW	Prec m/z	Prec z	Best Sequence	Modifications	Conf	Theor MW	z	115:114	%Err 115:114	116:
19.1.1.1353.2	46.374	1684.9115	843.4630	2	NLPFVGTLAPHVYGG	iTRAQ@N-term	99	1684.9060	2	0.7095	6.42	

Peptide ID Hypotheses

Conf	Sc	Prot N	Sequence	Modifications	Theor MW	Theor m/z	z	ΔMass
99	15	783	NLPFVGTLAPHVYGG	iTRAQ@N-term	1684.9060	843.4603	2	0.0055
< 1	7	25	AIAVGEVSNALARNR	iTRAQ@N-term, Deamidatio...	1684.9343	843.4744	2	-0.0228
< 1	7		EDLLEDAGVDPAGLK	No iTRAQ@N-term, iTRAQ(...	1684.8642	843.4394	2	0.0473
< 1	7		MTLDLPELPFPLR	iTRAQ@N-term	1684.9345	843.4745	2	-0.0230
< 1	6		TPRVEEAMRTVPR	iTRAQ@N-term	1684.9165	843.4655	2	-0.0050

Precursor MS Region

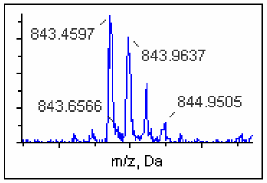

Fragmentation Evidence for Peptide

ITR NLPFVGTLAPHVYGG

Res	b	y
N	259.1523	1685.9132
L	372.2363	1427.7682
P	469.2891	1314.6841
F	616.3575	1217.6313
V	715.4259	1070.5629
G	772.4474	971.4945
T	873.4951	914.4730
L	986.5791	813.4254
A	1057.6162	700.3413
P	1154.6690	629.3042
H	1291.7279	532.2514
V	1390.7963	395.1925
Y	1553.8597	296.1241
G	1610.8811	133.0608
G	1667.9026	76.0393

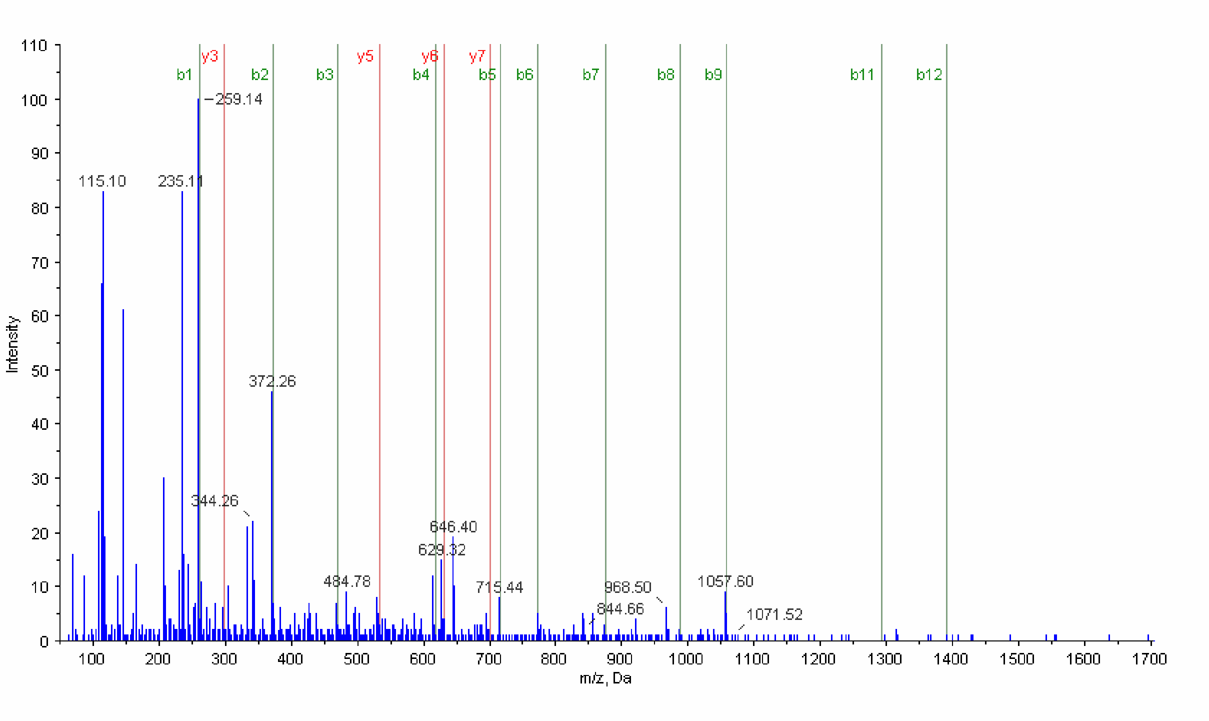

Protein: SCO3830, BkdB2 [1 of 2]

Protein Quant | Protein ID | Spectra | Summary Statistics

Spectrum List

Spectrum	Time	Prec MW	Prec m/z	Prec z	Best Sequence	Modifications	Conf	Theor MW	z	115:114	%Err 115:114	116:
36.1.1.2534.2	53.420	0.0000	843.4221	0	DAMAADPGVHVLGEDVGTLGGVFR	iTRAQ@N-term	99	2526.2659	3	1.9243	7.02	

Peptide ID Hypotheses

Conf	Sc	Prot N	Sequence	Modifications	Theor MW	Theor m/z	z	ΔMass
99	15	526	DAMAADPGVHVLGEDVGTLGGVFR	iTRAQ@N-term	2526.2659	843.0959	3	0.9787
< 1	8		TNIGHLEGAAGMAGLI	iTRAQ@N-term, Deamidatio...	1684.8576	843.4361	2	-0.0279
< 1	7		AGLPADWQPKTWDEVLDAAR	iTRAQ@N-term, Deamidatio...	2527.2952	843.4390	3	-0.0505
< 1	7		ATSPAVAAR	No iTRAQ@N-term	842.4610	843.4683	1	-0.0462
< 1	7		SLRDTVGMVTQDGHLFHDTVR	iTRAQ@N-term	2527.2724	843.4314	3	-0.0278

Precursor MS Region

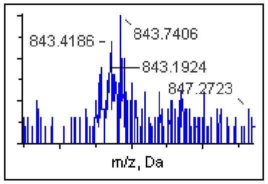

Fragmentation Evidence for Peptide

ITR DAMAADPGVHVLGEDVGTLGGVFR

Residue	b	y
D	260.1363	2527.2731
A	331.1734	2268.1441
M	462.2139	2197.1070
A	533.2510	2066.0665
A	604.2881	1995.0294
D	719.3151	1923.9923
P	816.3678	1808.9654
G	873.3893	1711.9126
V	972.4577	1654.8911
H	1109.5166	1555.8227
V	1208.5850	1418.7638
L	1321.6691	1319.6954
G	1378.6905	1206.6113
E	1507.7331	1149.5899
D	1622.7601	1020.5473
V	1721.8285	905.5203
G	1778.8500	806.4519
T	1879.8976	749.4304
L	1992.9817	648.3828
G	2050.0032	535.2987
G	2107.0246	478.2772
V	2206.0930	421.2558
F	2353.1615	322.1874
R	2509.2626	175.1190

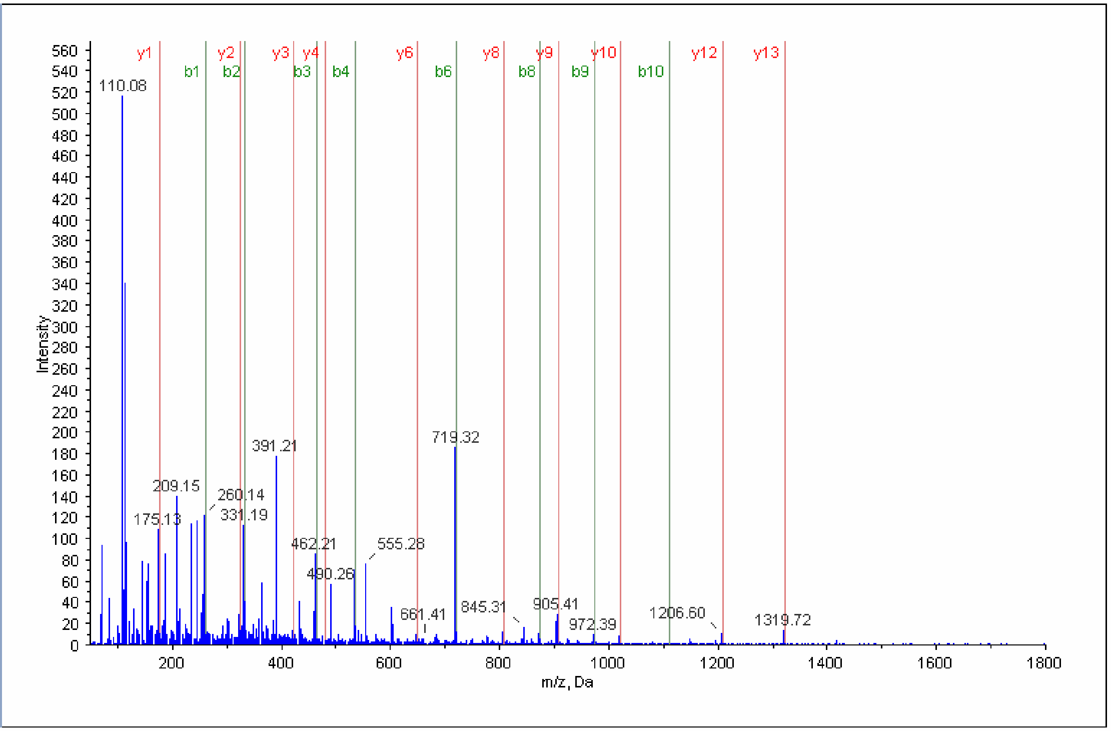

Protein: SCO3830, BkdB2 [2 of 2]

Protein Quant | Protein ID | Spectra | Summary Statistics

Spectrum List

Spectrum	Time	Prec MW	Prec m/z	Prec z	Best Sequence	Modifications	Conf	Theor MW	z	115:114	%Err 115:114	116:
36.1.1.2525.3	52.364	2542.2388	848.4202	3	DAMAADPGVHVLGEDVGTLGGVFR	iTRAQ@N-term, Oxidation(...	99	2542.2606	3	1.5356	8.33	

Peptide ID Hypotheses

Conf	Sc	Prot N	Sequence	Modifications	Theor MW	Theor m/z	z	ΔMass
99	18	526	DAMAADPGVHVLGEDVGTLGGVFR	iTRAQ@N-term, Oxidation(...	2542.2606	848.4275	3	-0.0219
< 1	8		AAWEAARGSAPGRHDDFGADGEGSR	No iTRAQ@N-term	2542.1332	848.3850	3	0.1055
< 1	8		ATVAGHAATFTGAGRWAESREGLAR	No iTRAQ@N-term	2542.2788	848.4335	3	-0.0400
< 1	7		AAVVVVGAAGADLGPADAYDVVVR	iTRAQ@N-term, iTRAQ(Y)@...	2542.3997	848.4738	3	-0.1610
< 1	7		AFVASLASAERPDRLGVVLMDGRDSVSSGGAR	iTRAQ@N-term	3389.7595	848.4472	4	-0.1077

Precursor MS Region

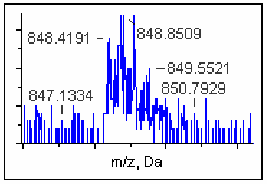

Fragmentation Evidence for Peptide

ITR DAM[Oxi]AADPGVHVLGEDVGTLGGVFR

Residue	b	y
D	**260.1363**	2543.2680
A	**331.1734**	2284.1390
M[Oxi]	**478.2088**	2213.1019
A	**549.2459**	2066.0665
A	**620.2830**	1995.0294
D	**735.3100**	1923.9923
P	832.3627	1808.9654
G	**889.3842**	1711.9126
V	**988.4526**	1654.8911
H	**1125.5115**	1555.8227
V	**1224.5799**	1418.7638
L	1337.6640	**1319.6954**
G	1394.6855	**1206.6113**
E	1523.7281	1149.5899
D	1638.7550	**1020.5473**
V	1737.8234	**905.5203**
G	1794.8449	**806.4519**
T	1895.8926	**749.4304**
L	2008.9766	**648.3828**
G	2065.9981	**535.2987**
G	2123.0195	**478.2772**
V	2222.0880	**421.2558**
F	2369.1564	**322.1874**
R	2525.2575	**175.1190**

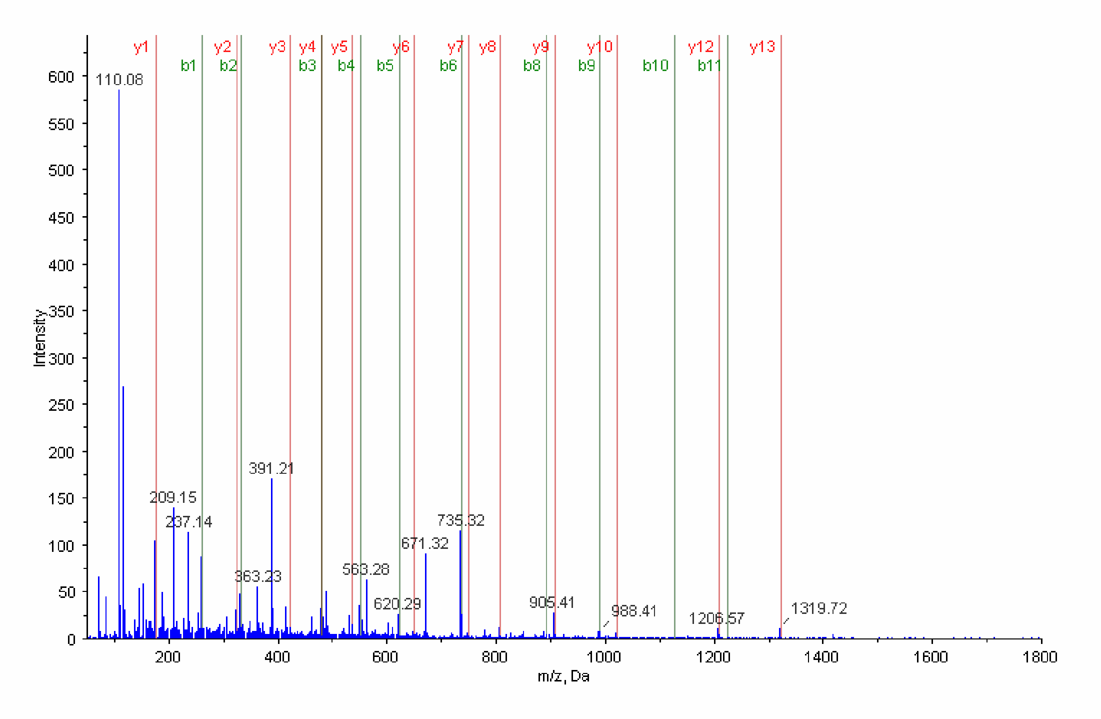

Protein: SCO3920, CysA [1 of 2]

Protein Quant | Protein ID | Spectra | Summary Statistics

Spectrum List

Spectrum	Time	Prec MW	Prec m/z	Prec z	Best Sequence	Modifications	Conf	Theor MW	z	115:114	%Err 115:114	116:
19.1.1.1440.2	54.185	1941.0204	971.5174	2	LSVGAEDPDDLVADLLR	iTRAQ@N-term	99	1941.0177	2	1.1602	17.19	

Peptide ID Hypotheses

Conf	Sc	Prot N	Sequence	Modifications	Theor MW	Theor m/z	z	ΔMass
99	13	492	LSVGAEDPDDLVADLLR	iTRAQ@N-term	1941.0177	971.5161	2	0.0027
< 1	7		VSNIETGIRTISPERLR	No iTRAQ@N-term, Deamid...	1941.0644	971.5395	2	-0.0440
< 1	6		LDAGLFFLAYQRDIR	iTRAQ@N-term	1941.0595	971.5370	2	-0.0391
< 1	6		LSLRGQGTTRAPGAEAGR	iTRAQ@N-term	1941.0627	971.5386	2	-0.0423
< 1	6		NDLGRVTEPGTVRVER	iTRAQ@N-term	1941.0514	971.5330	2	-0.0310

Precursor MS Region

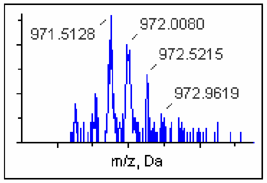

Fragmentation Evidence for Peptide

ITR LSVGAEDPDDLVADLLR

Res	b	y
L	258.1934	1942.0249
S	345.2254	1684.8388
V	444.2938	1597.8068
G	501.3153	1498.7384
A	572.3524	1441.7169
E	701.3950	1370.6798
D	816.4220	1241.6372
P	913.4747	1126.6103
D	1028.5017	1029.5575
D	1143.5286	914.5306
L	1256.6127	799.5036
V	1355.6811	686.4196
A	1426.7182	587.3511
D	1541.7451	516.3140
L	1654.8292	401.2871
L	1767.9133	288.2030
R	1924.0144	175.1190

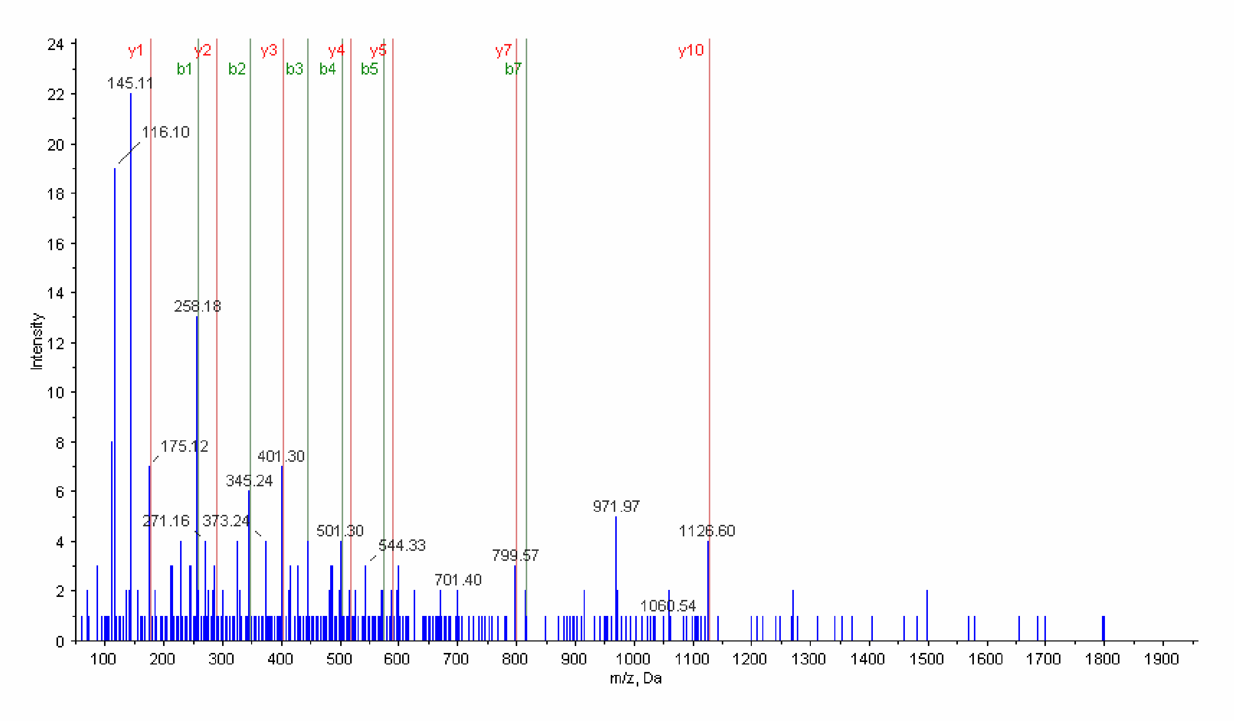

Protein: SCO3920, CysA [2 of 2]

Protein Quant | Protein ID | Spectra | Summary Statistics

Spectrum List

Spectrum	Time	Prec MW	Prec m/z	Prec z	Best Sequence	Modifications	Conf	Theor MW	z	115:114	%Err 115:114	116:
5.1.1.2134.3	48.517	1941.0127	971.5136	2	LSVGAEDPDDLVADLLR	iTRAQ@N-term	99	1941.0177	2	0.8533	5.95	

Peptide ID Hypotheses

Conf	Sc	Prot N	Sequence	Modifications	Theor MW	Theor m/z	z	ΔMass
99	16	390	LSVGAEDPDDLVADLLR	iTRAQ@N-term	1941.0177	971.5161	2	-0.0050
< 1	8		AVLAGGCFWGMEELIR	iTRAQ@N-term, MMTS(C)@7	1940.9433	971.4789	2	0.0694
< 1	8		RLASIDFPVPVEALDR	iTRAQ@N-term	1941.0805	971.5476	2	-0.0679
< 1	8		VAIANADAEDALERLAR	iTRAQ@N-term	1941.0401	971.5274	2	-0.0274
< 1	8		VTAVADAAEARARLLDR	iTRAQ@N-term	1941.0878	971.5512	2	-0.0751

Precursor MS Region

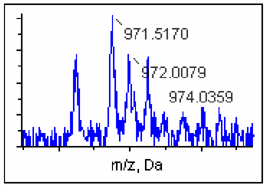

Fragmentation Evidence for Peptide

ITR LSVGAEDPDDLVADLLR

Residue	b	y
L	258.1934	1942.0249
S	345.2254	1684.8388
V	444.2938	1597.8068
G	501.3153	1498.7384
A	572.3524	1441.7169
E	701.3950	1370.6798
D	816.4220	1241.6372
P	913.4747	1126.6103
D	1028.5017	1029.5575
D	1143.5286	914.5306
L	1256.6127	799.5036
V	1355.6811	686.4196
A	1426.7182	587.3511
D	1541.7451	516.3140
L	1654.8292	401.2871
L	1767.9133	288.2030
R	1924.0144	175.1190

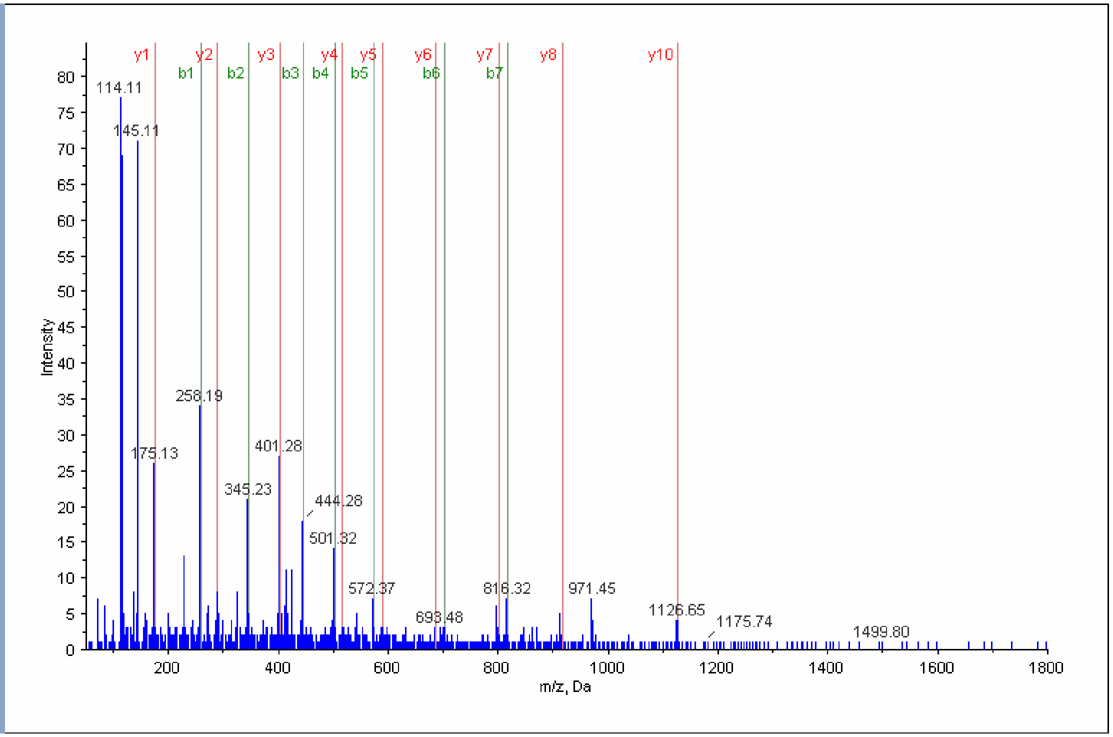

Protein: SCO4594 [1 of 1]

Protein Quant | Protein ID | Spectra | Summary Statistics

Spectrum List

Spectrum	Time	Prec MW	Prec m/z	Prec z	Best Sequence	Modifications	Conf	Theor MW	z	115:114	%Err 115:114	116:
38.1.1.2452.2	46.241	1898.0402	633.6873	3	LADPDTLHHTPIGVLR	iTRAQ@N-term	99	1898.0496	3	2.4765	3.17	

Peptide ID Hypotheses

Conf	Sc	Prot N	Sequence	Modifications	Theor MW	Theor m/z	z	ΔMass
99	15	423	LADPDTLHHTPIGVLR	iTRAQ@N-term	1898.0496	633.6905	3	-0.0095
< 1	9		NVTPEDVDKIAGELVR	No iTRAQ@N-term, iTRAQ(...	1898.0231	633.6816	3	0.0170
< 1	8		DTARVLGRMFDGIEYR	No iTRAQ@N-term	1897.9469	633.6562	3	0.0933
< 1	8		EARSVYWSAGGADMQDR	No iTRAQ@N-term	1897.8377	633.6199	3	0.2024
< 1	7		ALPLDESLSAAEARRR	iTRAQ@N-term	1898.0456	633.6892	3	-0.0054

Precursor MS Region

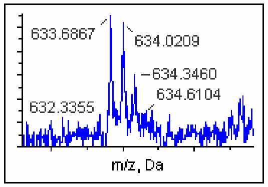

Fragmentation Evidence for Peptide

ITR LADPDTLHHTPIGVLR

Residue	b	y
L	258.1934	1899.0569
A	329.2305	1641.8707
D	444.2575	1570.8336
P	541.3102	1455.8067
D	656.3372	1358.7539
T	757.3848	1243.7270
L	870.4689	1142.6793
H	1007.5278	1029.5952
H	1144.5867	892.5363
T	1245.6344	755.4774
P	1342.6872	654.4297
I	1455.7712	557.3770
G	1512.7927	444.2929
V	1611.8611	387.2714
L	1724.9452	288.2030
R	1881.0463	175.1190

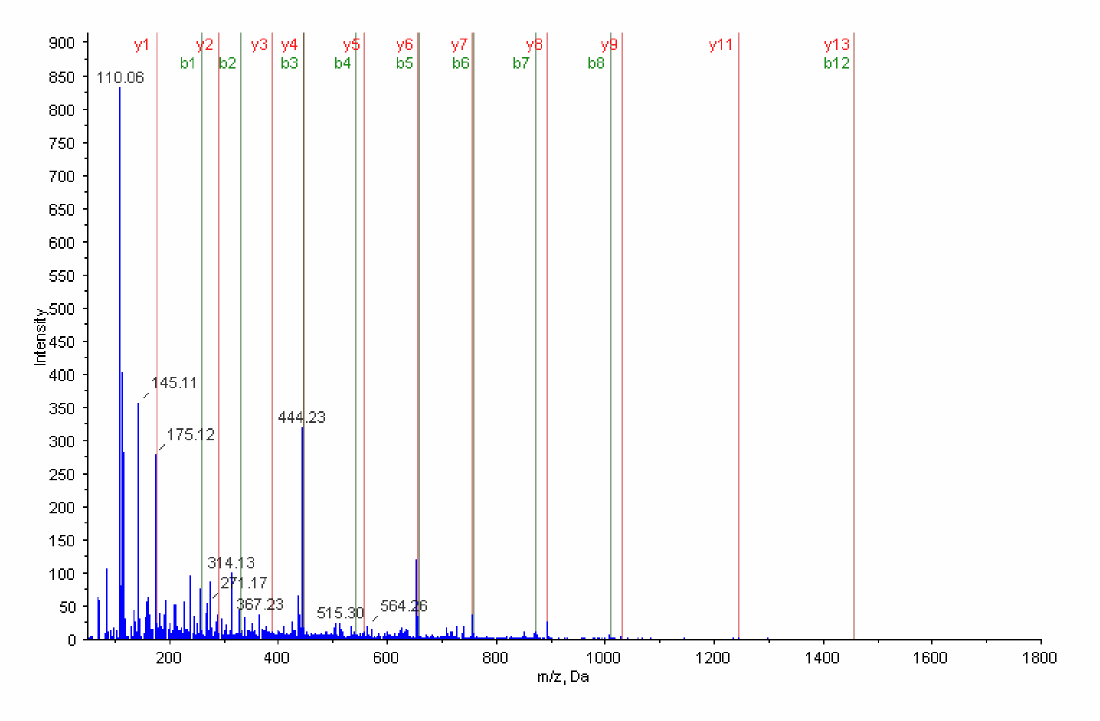

Protein: SCO4601 [1 of 1]

Protein Quant | Protein ID | Spectra | Summary Statistics

Spectrum List

Spectrum	Time	Prec MW	Prec m/z	Prec z	Best Sequence	Modifications	Conf	Theor MW	z	115:114	%Err 115:114	116:
16.1.1.1029.2	26.910	0.0000	968.4505	0	AAGPADGPTTADTAAGQGDGTST...	iTRAQ@N-term, Deamidatio...	99	2902.3298	3			

Peptide ID Hypotheses

Conf	Sc	Prot N	Sequence	Modifications	Theor MW	Theor m/z	z	ΔMass
99	16	613	AAGPADGPTTADTAAGQGDGTSTGTGAPATGR	iTRAQ@N-term, Deamidatio...	2902.3298	968.4506	3	-0.0002
< 1	6		ELDWREILKAWCASMPEDWGYTPDNMTGPIR	No iTRAQ@N-term, iTRAQ(...	3869.7796	968.4522	4	-0.0067
< 1	6		WLVSMDGSPARTADGGR	iTRAQ@N-term, Oxidation(...	1934.9391	968.4768	2	-0.0526
< 1	5		AAGFSPETVLEVEDDGR	iTRAQ@N-term	1934.9344	968.4744	2	-0.0479
< 1	5		AAPADTPTGAP	No iTRAQ@N-term	967.4611	968.4683	1	-0.0178

Precursor MS Region

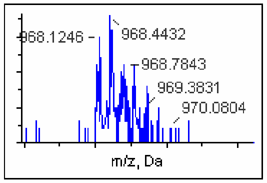

Fragmentation Evidence for Peptide

ITR | AAGPADGPTTADTAAGQ[Dea]GDGTSTGTGAPATGR

Res	b	y
A	216.1465	2903.33
A	287.1836	2688.19
G	344.2050	2617.16
P	441.2578	2560.13
A	512.2949	2463.08
D	627.3219	2392.04
G	684.3433	2277.02
P	781.3961	2220.00
T	882.4438	2122.94
T	983.4914	2021.90
A	1054.5285	1920.85
D	1169.5555	1849.81
T	1270.6032	1734.78
A	1341.6403	1633.74
A	1412.6774	1562.70
G	1469.6989	1491.66
Q[	1598.7415	1434.64
G	1655.7629	1305.60
D	1770.7899	1248.58
G	1827.8113	1133.55
T	1928.8590	1076.53
S	2015.8910	975.48
T	2116.9387	888.45
G	2173.9602	787.40
T	2275.0079	730.38

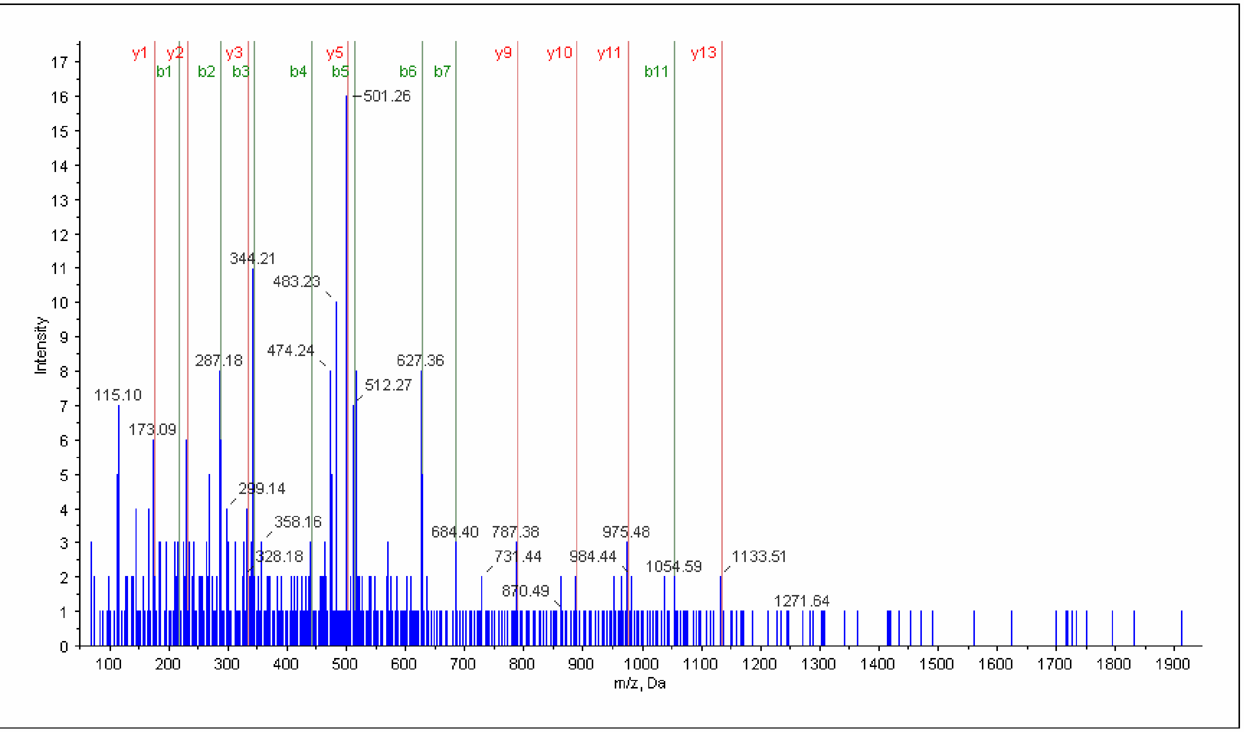

Protein: SCO4603, NuoI2 [1 of 1]

Protein Quant | Protein ID | Spectra | Summary Statistics

Spectrum List

Spectrum	Time	Prec MW	Prec m/z	Prec z	Best Sequence	Modifications	Conf	Theor MW	z	115:114	%Err 115:114	116:
38.1.1.2365.3	42.696	2158.0144	720.3454	3	LAAQQQPDQPGPDHPGQPDESGQ...	iTRAQ@N-term	99	2882.3303	4	1.1283	4.83	

Peptide ID Hypotheses

Conf	Sc	Prot N	Sequence	Modifications	Theor MW	Theor m/z	z	ΔMass
99	15	519	LAAQQQPDQPGPDHPGQPDESGQEGR	iTRAQ@N-term	2882.3303	721.5899	4	-4.9777
< 1	9	438	EAAQSLAGGRPEAEEDDGGR	iTRAQ@N-term	2158.0010	720.3409	3	0.0134
< 1	8		EAAEQEATLEAVAQRLER	iTRAQ@N-term, Deamidatio...	2158.0989	720.3736	3	-0.0845
< 1	8		MAGMATHVITGAGSGIGAAVAR	iTRAQ@N-term, Oxidation(...	2158.1110	720.3776	3	-0.0966
< 1	7		GEPRRVQAYDGELGAAVAR	iTRAQ@N-term	2158.1366	720.3862	3	-0.1222

Precursor MS Region

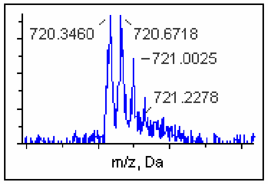

Fragmentation Evidence for Peptide

ITR LAAQQQPDQPGPDHPGQPDESGQEGR

Residue	b	y
L	**258.1934**	2883.3374
A	**329.2305**	2626.1513
A	**400.2676**	2555.1142
Q	**528.3262**	2484.0770
Q	**656.3848**	2356.0185
Q	**784.4434**	2227.9599
P	881.4961	2099.9013
D	996.5231	2002.8486
Q	1124.5816	1887.8216
P	1221.6344	1759.7630
G	1278.6559	1662.7103
P	1375.7086	1605.6888
D	1490.7356	1508.6360
H	1627.7945	**1393.6091**
P	1724.8473	**1256.5502**
G	1781.8687	1159.4974
Q	1909.9273	1102.4760
P	2006.9801	**974.4174**
D	2122.0070	877.3646
E	2251.0496	**762.3377**
S	2338.0816	**633.2951**
G	2395.1031	**546.2631**
Q	2523.1617	489.2416
E	2652.2043	**361.1830**
G	2709.2257	**232.1404**
[illegible]	[illegible]	[illegible]

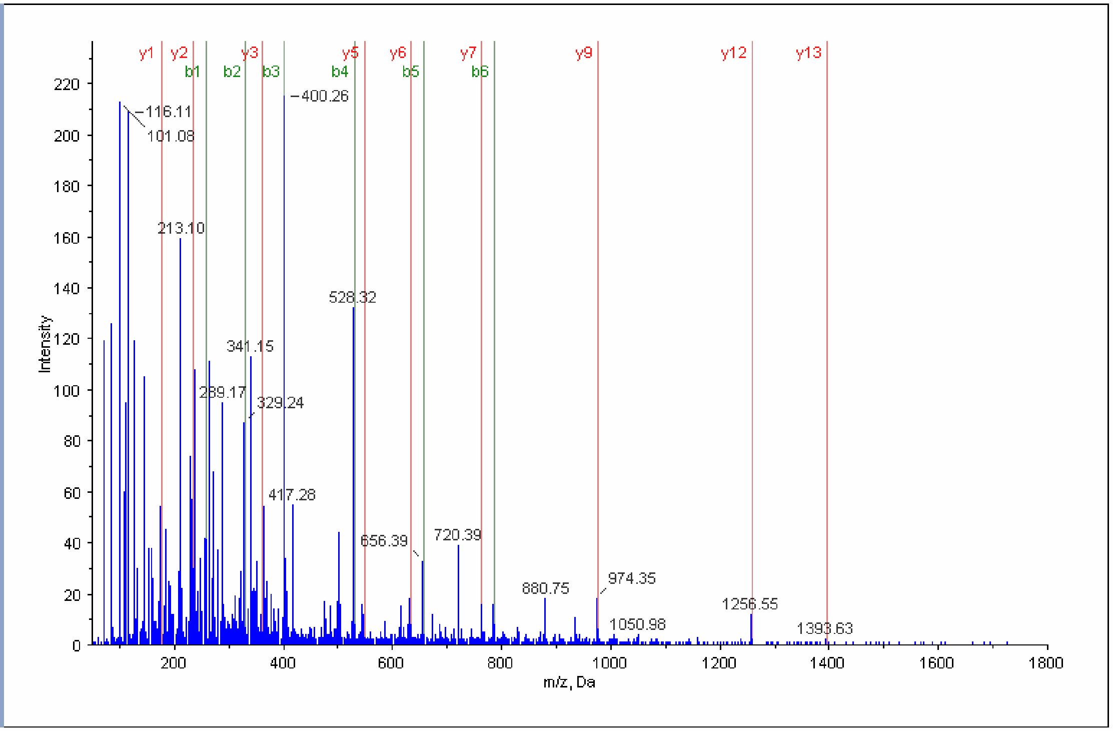

Protein: SCO4605, NuoK2 [1 of 1]

Protein Quant | Protein ID | Spectra | Summary Statistics

Spectrum List

Spectrum	Time	Prec MW	Prec m/z	Prec z	Best Sequence	Modifications	Conf	Theor MW	z	115:114	%Err 115:114	116:
48.1.1.2523.2	44.355	2670.2371	891.0863	3	DTAEGHEPDGPDTDTPATGTAAEK	iTRAQ@N-term, iTRAQ(K)@...	98	2670.2137	3	1.3288	9.37	

Peptide ID Hypotheses

Conf	Sc	Prot N	Sequence	Modifications	Theor MW	Theor m/z	z	ΔMass
98	14	587	DTAEGHEPDGPDTDTPATGTAAEK	iTRAQ@N-term, iTRAQ(K)@...	2670.2137	891.0785	3	0.0234
< 1	6		GTGPDGTVRPRGRTHHAYGTSGYDR	No iTRAQ@N-term	2670.2760	891.0992	3	-0.0389

Precursor MS Region

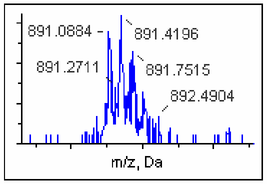

Fragmentation Evidence for Peptide

ITR | DTAEGHEPDGPDTDTPATGTAAEK[ITR]

Residue	b	y
D	**260.1363**	2671.2209
T	**361.1840**	2412.0919
A	**432.2211**	2311.0442
E	**561.2637**	2240.0071
G	**618.2851**	2110.9645
H	**755.3440**	2053.9431
E	**884.3866**	1916.8842
P	981.4394	1787.8416
D	1096.4663	1690.7888
G	1153.4878	1575.7619
P	1250.5406	1518.7404
D	1365.5675	1421.6876
T	1466.6152	1306.6607
D	1581.6421	1205.6130
T	1682.6898	1090.5861
P	1779.7426	**989.5384**
A	1850.7797	892.4856
T	1951.8274	**821.4485**
G	2008.8488	**720.4008**
T	2109.8965	**663.3794**
A	2180.9336	**562.3317**
A	2251.9707	**491.2946**
E	2381.0133	**420.2575**
K[ITR]	2653.2104	**291.2149**

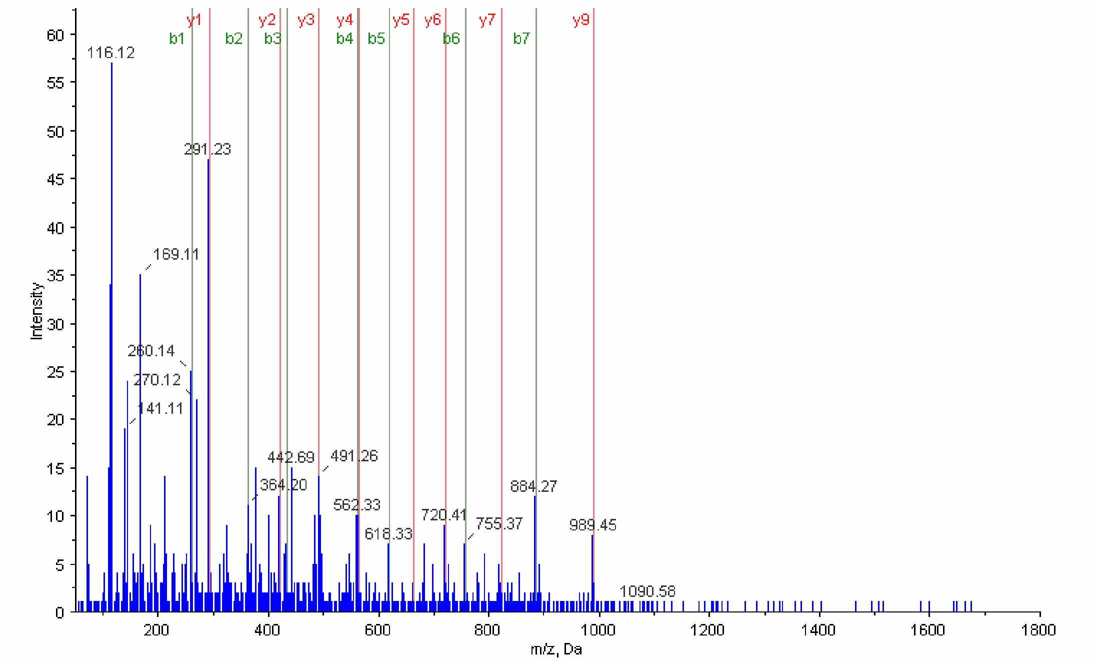

Protein: SCO4979 [1 of 2]

Protein Quant | Protein ID | Spectra | Summary Statistics

Spectrum List

Spectrum	Time	Prec MW	Prec m/z	Prec z	Best Sequence	Modifications	Conf	Theor MW	z	115:114	%Err 115:114	116:
56.1.1.2404.2	52.361	1827.9717	610.3312	3	DEGWLAEHMLVLK	iTRAQ@N-term, iTRAQ(K)@...	99	1827.9797	3	0.8682	4.56	

Peptide ID Hypotheses

Conf	Sc	Prot N	Sequence	Modifications	Theor MW	Theor m/z	z	ΔMass
99	14	506	DEGWLAEHMLVLK	iTRAQ@N-term, iTRAQ(K)@...	1827.9797	610.3339	3	-0.0080
< 1	8		GPGGLLHNVGVEALIQVR	No iTRAQ@N-term	1828.0319	610.3513	3	-0.0603
< 1	7		DIAATIQPEQDDLVR	iTRAQ@N-term, Deamidatio...	1827.9336	610.3185	3	0.0381
< 1	7		IDRAGARTDGLLGDVR	iTRAQ@N-term	1828.0037	610.3418	3	-0.0320
< 1	7		RRMPVGAVGITGAVER	iTRAQ@N-term, Oxidation(...	1828.0223	610.3481	3	-0.0507

Precursor MS Region

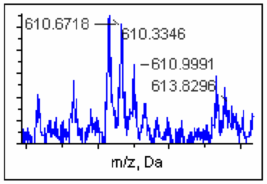

Fragmentation Evidence for Peptide

ITR DEGWLAEHMLVLK[ITR]

Residue	b	y
D	260.1363	1828.9870
E	389.1789	1569.8579
G	446.2003	1440.8154
W	632.2797	1383.7939
L	745.3637	1197.7146
A	816.4008	1084.6305
E	945.4434	1013.5934
H	1082.5023	884.5508
M	1213.5428	747.4919
L	1326.6269	616.4514
V	1425.6953	503.3673
L	1538.7794	404.2989
K[ITR]	1810.9764	291.2149

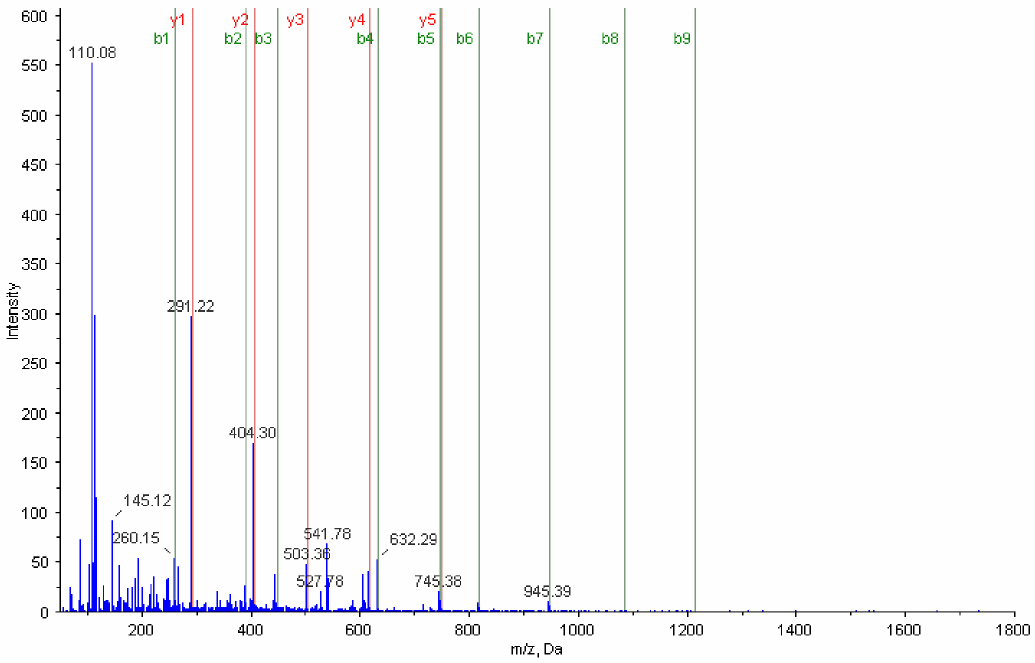

Protein: SCO4979 [1 of 2]

Protein Quant | Protein ID | Spectra | Summary Statistics

Spectrum List

Spectrum	Time	Prec MW	Prec m/z	Prec z	Best Sequence	Modifications	Conf	Theor MW	z	115:114	%Err 115:114	116:
56.1.1.2405.2	52.482	1827.9720	610.3313	3	DEGWLAEHMLVLK	iTRAQ@N-term, iTRAQ(K)@...	99	1827.9797	3	1.0970	4.36	

Peptide ID Hypotheses

Conf	Sc	Prot N	Sequence	Modifications	Theor MW	Theor m/z	z	ΔMass
99	15	506	DEGWLAEHMLVLK	iTRAQ@N-term, iTRAQ(K)@...	1827.9797	610.3339	3	-0.0076
< 1	6		ALYVGVSNYSPEQTR	iTRAQ@N-term, Deamidatio...	1827.9125	610.3114	3	0.0596
< 1	6		GVPMSTPVTGAVQRER	iTRAQ@N-term	1827.9747	610.3322	3	-0.0027
< 1	6		SRRGAELISVIAQER	iTRAQ@N-term	1828.0401	610.3540	3	-0.0680
< 1	5		EDNSFVLPLELRPR	iTRAQ@N-term	1827.9965	610.3394	3	-0.0245

Precursor MS Region

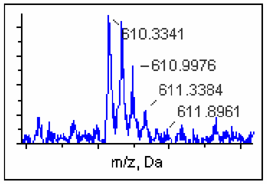

Fragmentation Evidence for Peptide

ITR DEGWLAEHMLVLK[ITR]

Residue	b	y
D	260.1363	1828.9870
E	389.1789	1569.8579
G	446.2003	1440.8154
W	632.2797	1383.7939
L	745.3637	1197.7146
A	816.4008	1084.6305
E	945.4434	1013.5934
H	1082.5023	884.5508
M	1213.5428	747.4919
L	1326.6269	616.4514
V	1425.6953	503.3673
L	1538.7794	404.2989
K[ITR]	1810.9764	291.2149

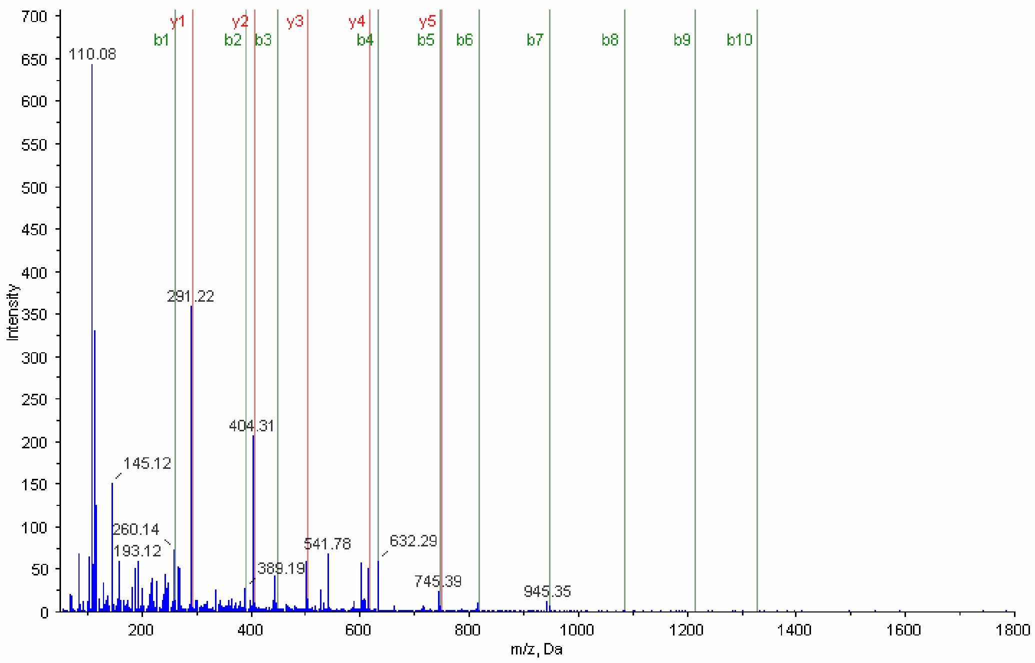

Protein: SCO5042, FumC [1 of 2]

Protein Quant | Protein ID | Spectra | Summary Statistics

Spectrum List

Spectrum	Time	Prec MW	Prec m/z	Prec z	Best Sequence	Modifications	Conf	Theor MW	z	115:114	%Err 115:114	116:
31.1.1.1082.5	50.632	1747.9816	583.6678	3	DLIPALDHLAGALER	iTRAQ@N-term	99	1746.9750	3			

Peptide ID Hypotheses

Conf	Sc	Prot N	Sequence	Modifications	Theor MW	Theor m/z	z	ΔMass
99	13	750	DLIPALDHLAGALER	iTRAQ@N-term	1746.9750	583.3323	3	1.0065
< 1	6		EVAPGVTVEGPVPQLTR	No iTRAQ@N-term	1747.9469	583.6562	3	0.0347
< 1	6		IGDLHGDRRLGALAER	No iTRAQ@N-term	1747.9442	583.6553	3	0.0374
< 1	5		AIGMVERPEYKRR	No iTRAQ@N-term, iTRAQ(...	1747.9638	583.6619	3	0.0178
< 1	5		LGTNAYRLSVAWPR	iTRAQ@N-term, Deamidatio...	1747.9492	583.6570	3	0.0324

Precursor MS Region

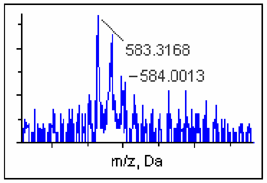

Fragmentation Evidence for Peptide

ITR DLIPALDHLAGALER

Res	b	y
D	260.1363	1747.9823
L	373.2203	1488.8533
I	486.3044	1375.7692
P	583.3572	1262.6852
A	654.3943	1165.6324
L	767.4784	1094.5953
D	882.5053	981.5112
H	1019.5642	866.4843
L	1132.6483	729.4254
A	1203.6854	616.3413
G	1260.7068	545.3042
A	1331.7440	488.2827
L	1444.8280	417.2456
E	1573.8706	304.1615
R	1729.9717	175.1190

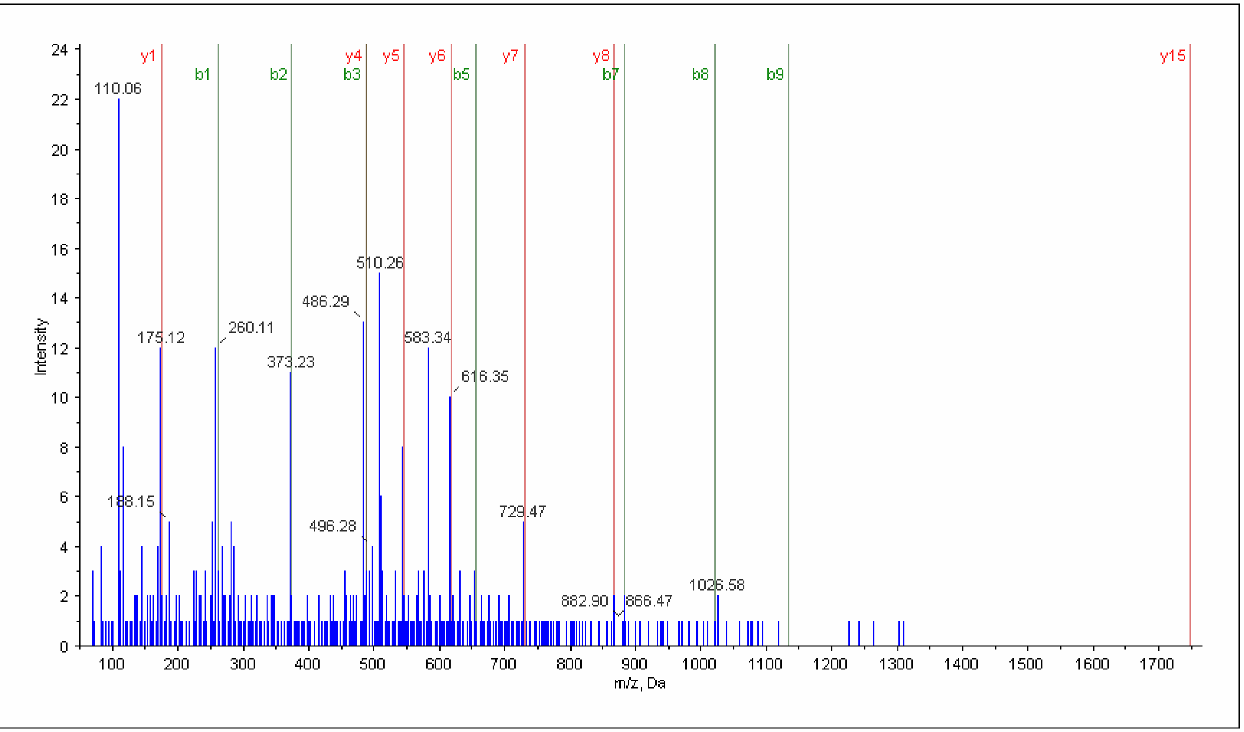

Protein: SCO5042, FumC [2 of 2]

Protein Quant | Protein ID | Spectra | Summary Statistics

Spectrum List

Spectrum	Time	Prec MW	Prec m/z	Prec z	Best Sequence	Modifications	Conf	Theor MW	z	115:114	%Err 115:114	116:
50.1.1.1084.4	51.575	1746.9734	583.3318	3	DLIPALDHLAGALER	iTRAQ@N-term	99	1746.9750	3			

Peptide ID Hypotheses

Conf	Sc	Prot N	Sequence	Modifications	Theor MW	Theor m/z	z	ΔMass
99	12	750	DLIPALDHLAGALER	iTRAQ@N-term	1746.9750	583.3323	3	-0.0016
< 1	5	621	AAQQTLPPHLATTPR	iTRAQ@N-term, Deamidatio...	1746.9386	583.3201	3	0.0348
< 1	6		LVAEALGDDRVLHLAR	No iTRAQ@N-term	1746.9740	583.3319	3	-0.0006
< 1	6		VINKYLDQVDPR	iTRAQ@N-term, iTRAQ(K)@4	1746.9872	583.3363	3	-0.0138
< 1	5		DLLLAHASHWLDGR	iTRAQ@N-term	1746.9287	583.3168	3	0.0447

Precursor MS Region

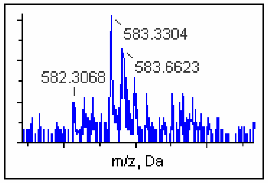

Fragmentation Evidence for Peptide

ITR DLIPALDHLAGALER

Res	b	y
D	**260.1363**	1747.9823
L	**373.2203**	1488.8533
I	**486.3044**	1375.7692
P	**583.3572**	**1262.6852**
A	**654.3943**	1165.6324
L	767.4784	1094.5953
D	882.5053	981.5112
H	**1019.5642**	866.4843
L	1132.6483	**729.4254**
A	1203.6854	**616.3413**
G	1260.7068	**545.3042**
A	1331.7440	**488.2827**
L	1444.8280	417.2456
E	1573.8706	304.1615
R	1729.9717	**175.1190**

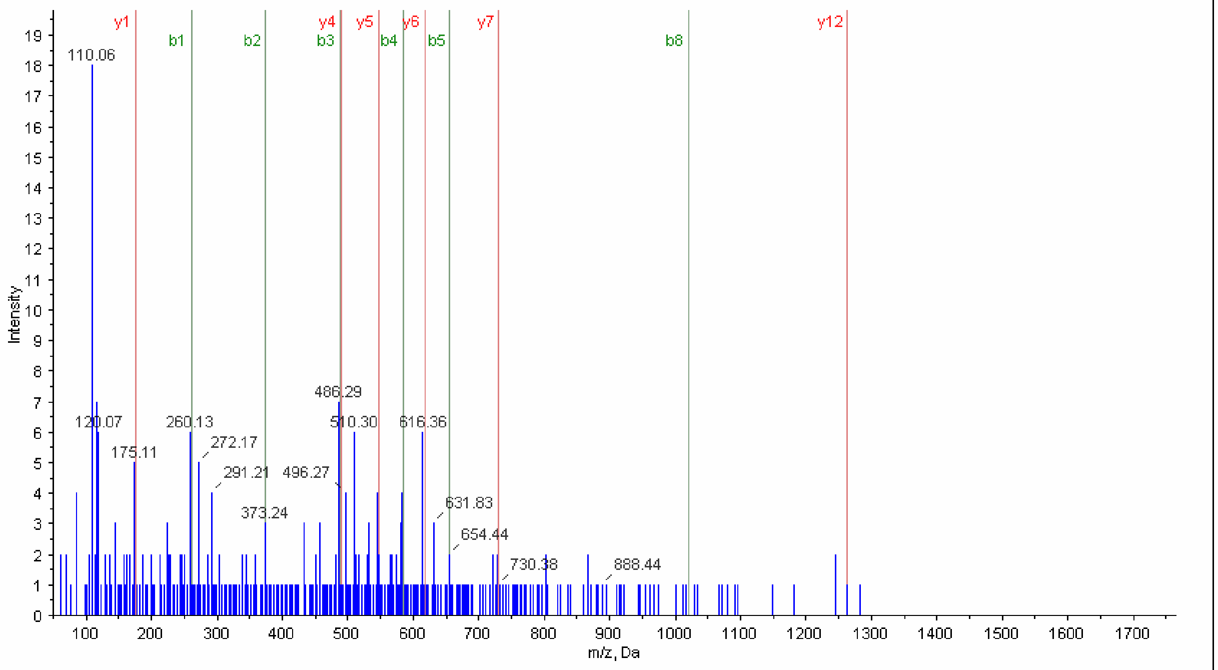

Protein: SCO5059, PpgK [1 of 2]

Protein Quant | Protein ID | Spectra | Summary Statistics

Spectrum List

Spectrum	Time	Prec MW	Prec m/z	Prec z	Best Sequence	Modifications	Conf	Theor MW	z	115:114	%Err 115:114	116:
1.1.1.1007.4	32.126	1961.0890	654.7036	3	VLTPHPATPDGVADGVK	iTRAQ@N-term, iTRAQ(K)@...	99	1961.0824	3	0.9284	13.80	

Peptide ID Hypotheses

Conf	Sc	Prot N	Sequence	Modifications	Theor MW	Theor m/z	z	ΔMass
99	15	679	VLTPHPATPDGVADGVK	iTRAQ@N-term, iTRAQ(K)@...	1961.0824	654.7014	3	0.0065
< 1	5	75	ILSGGVDSTALYPPKR	iTRAQ@N-term, iTRAQ(K)@...	1961.1188	654.7135	3	-0.0298
< 1	7		EVVVPVATPADLPSARSK	PGA of E@N-term, iTRAQ(K...	1961.1066	654.7095	3	-0.0177
< 1	7		LSGAAGRNVLNAAYLVAR	iTRAQ@N-term, Deamidatio...	1961.0815	654.7011	3	0.0075
< 1	7		PEGVFLGGSNLAVAAGSK	iTRAQ@N-term, iTRAQ(K)@...	1961.0824	654.7014	3	0.0065

Precursor MS Region

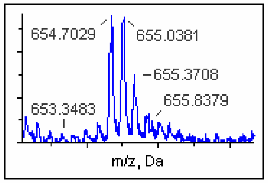

Fragmentation Evidence for Peptide

ITR | VLTPHPATPDGVADGVK[ITR]

Res	b	y
V	244.1778	1962.0898
L	357.2618	1718.9194
T	458.3095	1605.8353
P	555.3623	1504.7876
H	692.4212	1407.7349
P	789.4739	1270.6759
A	860.5110	1173.6232
T	961.5587	1102.5861
P	1058.6115	1001.5384
D	1173.6384	904.4856
G	1230.6599	789.4587
V	1329.7283	732.4372
A	1400.7654	633.3688
D	1515.7924	562.3317
G	1572.8138	447.3047
V	1671.8822	390.2833
K[I	1944.0793	291.2149

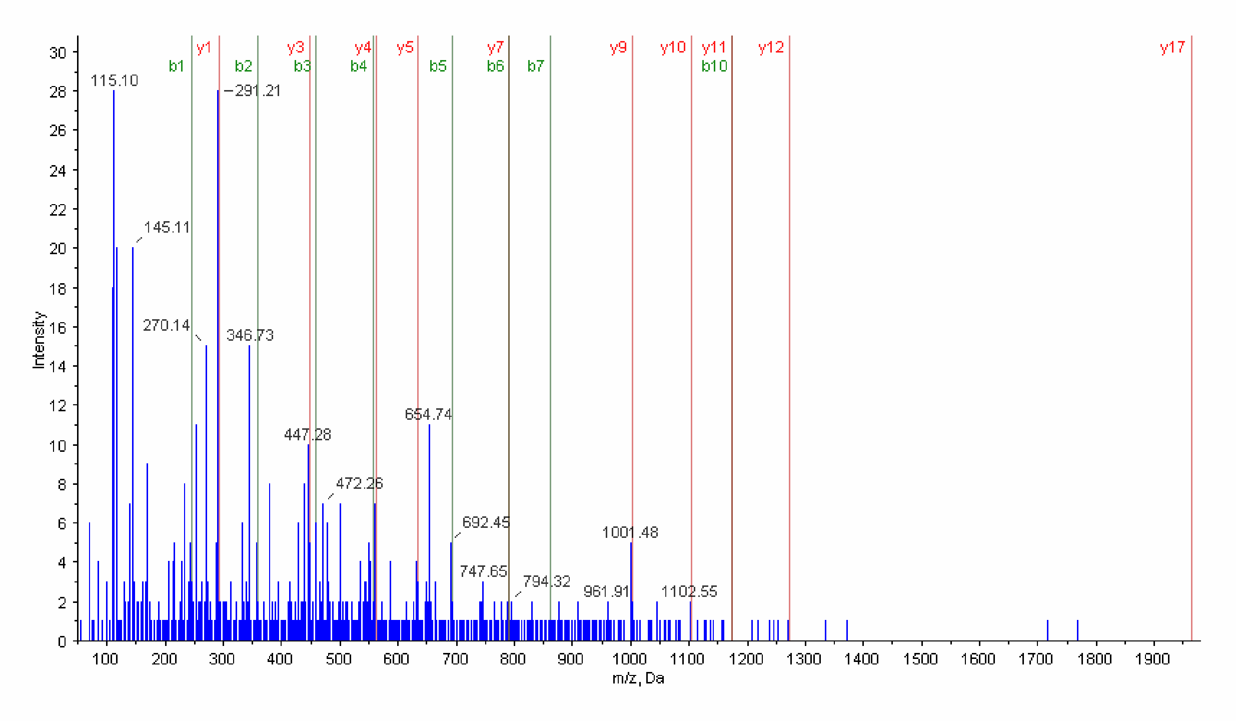

Protein: SCO5059, PpgK [2 of 2]

Protein Quant | Protein ID | Spectra | Summary Statistics

Spectrum List

Spectrum	Time	Prec MW	Prec m/z	Prec z	Best Sequence	Modifications	Conf	Theor MW	z	115:114	%Err 115:114	116:
32.1.1.961.2	30.891	1961.0903	654.7040	3	VLTPHPATPDGVADGVK	iTRAQ@N-term, iTRAQ(K)@...	99	1961.0826	3	0.9754	6.38	

Peptide ID Hypotheses

Conf	Sc	Prot N	Sequence	Modifications	Theor MW	Theor m/z	z	ΔMass
99	18	679	VLTPHPATPDGVADGVK	iTRAQ@N-term, iTRAQ(K)@...	1961.0826	654.7015	3	0.0077
< 1	8		ALSAAKGSKDPATGQK	No iTRAQ@N-term, iTRAQ(...	1961.1271	654.7163	3	-0.0368
< 1	7		AAELMERVGLDASLAK	iTRAQ@N-term, iTRAQ(K)@...	1961.0860	654.7026	3	0.0044
< 1	6		AAPAVLDSLAVVCNRK	iTRAQ@N-term, MMTS(C)@...	1961.0682	654.6967	3	0.0221
< 1	6		AQIDGITVGGKTGTAQR	iTRAQ@N-term, Deamidatio...	1961.0786	654.7001	3	0.0118

Precursor MS Region

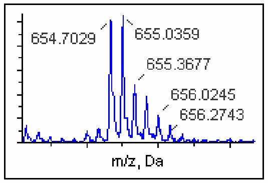

Fragmentation Evidence for Peptide

ITR VLTPHPATPDGVADGVK[ITR]

Res	b	y
V	244.1778	1962.0898
L	357.2618	1718.9194
T	458.3095	1605.8353
P	555.3623	1504.7876
H	692.4212	1407.7349
P	789.4739	1270.6759
A	860.5110	1173.6232
T	961.5587	1102.5861
P	1058.6115	1001.5384
D	1173.6384	904.4856
G	1230.6599	789.4587
V	1329.7283	732.4372
A	1400.7654	633.3688
D	1515.7924	562.3317
G	1572.8138	447.3047
V	1671.8822	390.2833
K[I	1944.0793	291.2149

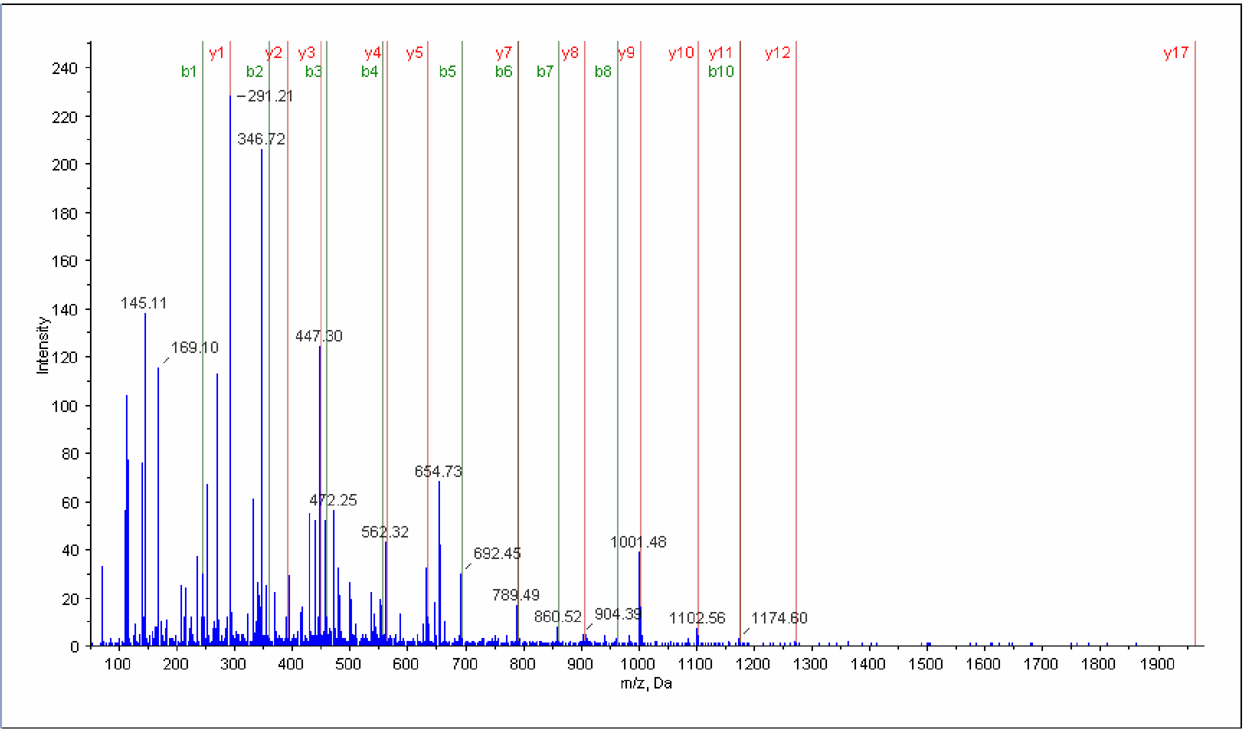

Protein: SCO5084, ActII-3 [1 of 1]

Protein Quant | Protein ID | Spectra | Summary Statistics

Spectrum List

Spectrum	Time	Prec MW	Prec m/z	Prec z	Best Sequence	Modifications	Conf	Theor MW	z	115:114	%Err 115:114	116:
50.1.1.1011.5	34.795	2259.1226	754.0481	3	ADATHFADGTGVVGEPYGPVR	iTRAQ@N-term	99	2259.1043	3			

Peptide ID Hypotheses

Conf	Sc	Prot N	Sequence	Modifications	Theor MW	Theor m/z	z	ΔMass
99	22	836	ADATHFADGTGVVGEPYGPVR	iTRAQ@N-term	2259.1043	754.0420	3	0.0183
<1	8		EKPGMCHTGAQRNGVSAAVR	No iTRAQ@N-term, iTRAQ(...	2259.0793	754.0337	3	0.0432
<1	7		AQGELFDAMGFLPTFTDVR	iTRAQ@N-term, Deamidatio...	2259.1004	754.0407	3	0.0221
<1	7		DGTDDALTSEVAGRILADERR	No iTRAQ@N-term	2259.1092	754.0437	3	0.0134
<1	7		LFIERGFEHTTIAEIADAAR	No iTRAQ@N-term	2259.1649	754.0622	3	-0.0423

Precursor MS Region

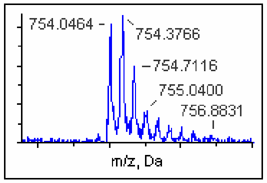

Fragmentation Evidence for Peptide

ITR ADATHFADGTGVVGEPYGPVR

Res	b	y
A	216.1465	2260.1115
D	331.1734	2044.9723
A	402.2105	1929.9454
T	503.2582	1858.9082
H	640.3171	1757.8606
F	787.3855	1620.8016
A	858.4226	1473.7332
D	973.4496	1402.6961
G	1030.4710	1287.6692
T	1131.5187	1230.6477
G	1188.5402	1129.6000
V	1287.6086	1072.5786
V	1386.6770	973.5102
G	1443.6985	874.4417
E	1572.7411	817.4203
P	1669.7938	688.3777
Y	1832.8572	591.3249
G	1889.8786	428.2616
P	1986.9314	371.2401
V	2085.9998	274.1874
R	2242.1009	175.1190

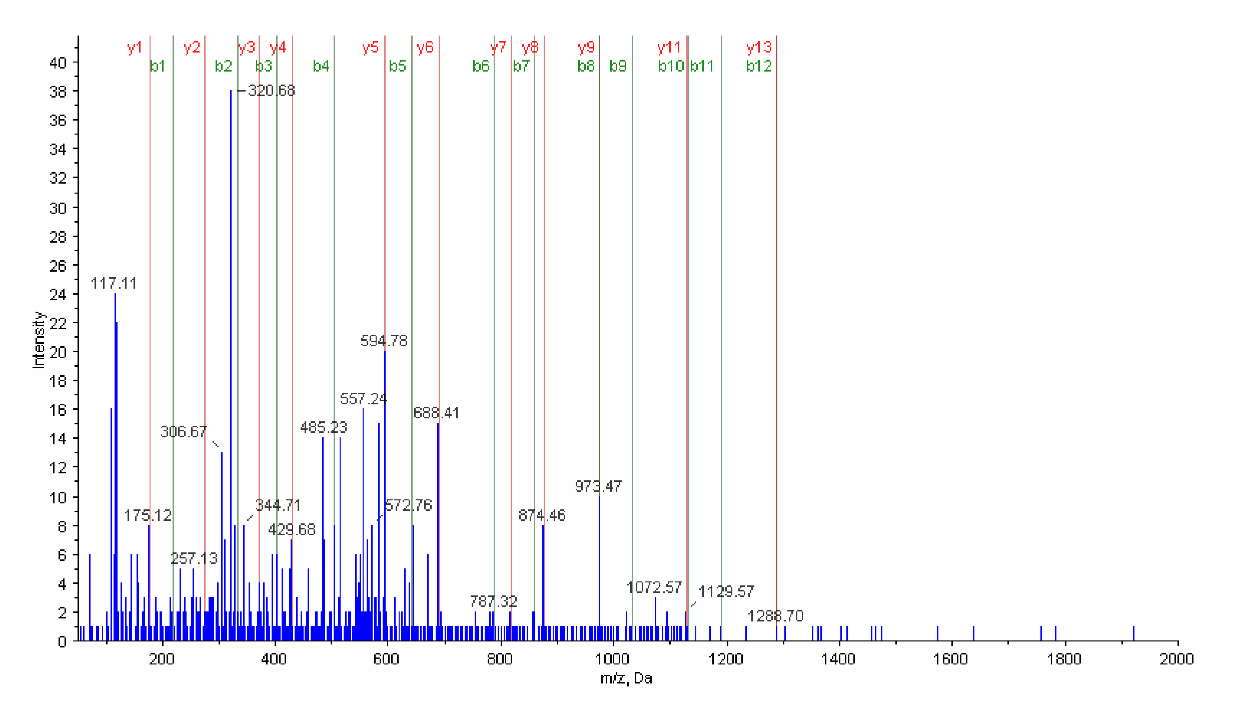

Protein: SCO5086, ActII [1 of 18]

Protein Quant | Protein ID | Spectra | Summary Statistics

Spectrum List

Spectrum	Time	Prec MW	Prec m/z	Prec z	Best Sequence	Modifications	Conf	Theor MW	z	115:114	%Err 115:114	116:
20.1.1.1087.2	58.588	1724.9767	575.9995	3	SVPEIEALVAAVVER	iTRAQ@N-term	99	1724.9794	3			

Peptide ID Hypotheses

Conf	Sc	Prot N	Sequence	Modifications	Theor MW	Theor m/z	z	ΔMass
99	17	744	SVPEIEALVAAVVER	iTRAQ@N-term	1724.9794	576.0004	3	-0.0027
< 1	9		AISLGMPDVPTRLAER	No iTRAQ@N-term	1724.9243	575.9820	3	0.0524
< 1	8		GVLAEMVRVLKPGGR	No iTRAQ@N-term, iTRAQ(...	1725.0205	576.0141	3	-0.0438
< 1	7		PAAYVPLPIGNGEQR	iTRAQ@N-term	1724.9331	575.9850	3	0.0436
< 1	7		TALTLVAADALEVAPDR	No iTRAQ@N-term	1724.9308	575.9842	3	0.0459

Precursor MS Region

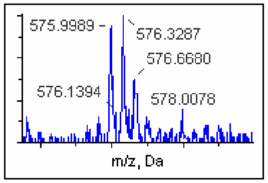

Fragmentation Evidence for Peptide

ITR SVPEIEALVAAVVER

Res	b	y
S	232.1414	1725.9867
V	331.2098	1494.8526
P	428.2625	1395.7842
E	557.3051	1298.7314
I	670.3892	1169.6888
E	799.4318	1056.6048
A	870.4689	927.5622
L	983.5530	856.5251
V	1082.6214	743.4410
A	1153.6585	644.3726
A	1224.6956	573.3355
V	1323.7640	502.2984
V	1422.8324	403.2300
E	1551.8750	304.1615
R	1707.9761	175.1190

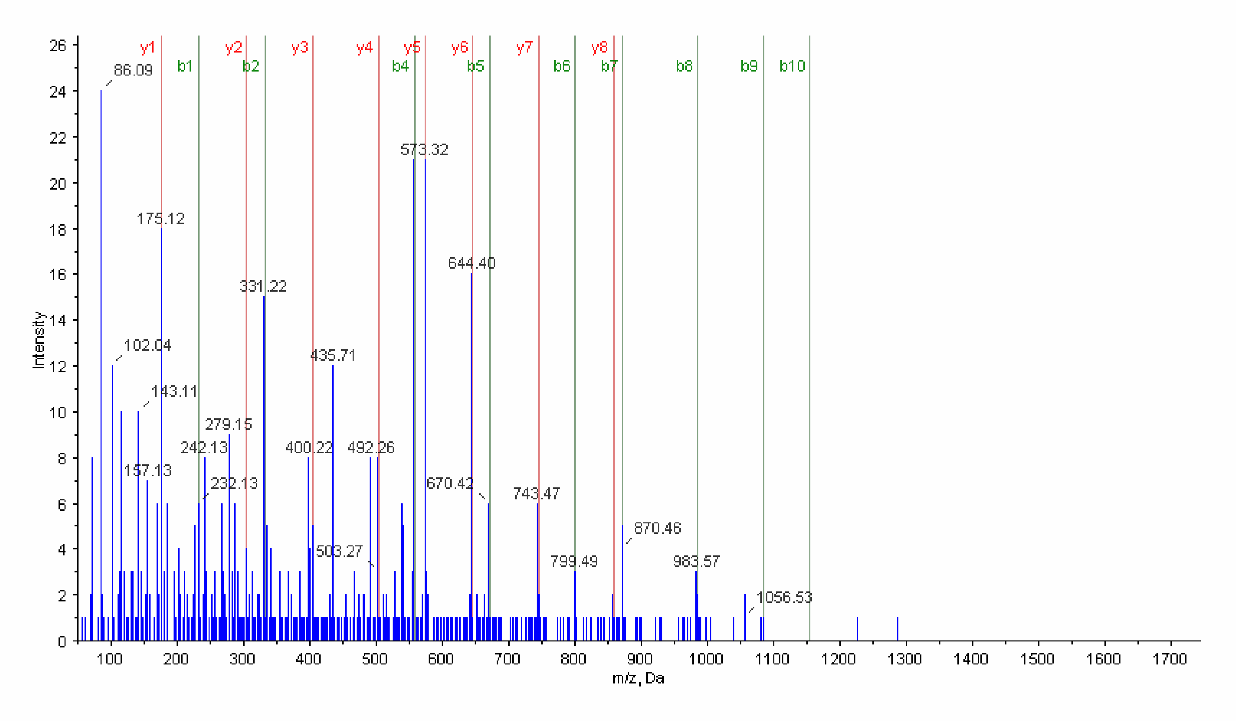

Protein: SCO5086, ActII [2 of 18]

Protein Quant | Protein ID | Spectra | Summary Statistics

Spectrum List

Spectrum	Time	Prec MW	Prec m/z	Prec z	Best Sequence	Modifications	Conf	Theor MW	z	115:114	%Err 115:114	116:
20.1.1.1088.3	58.870	1724.9734	863.4940	2	SVPEIEALVAAVVER	iTRAQ@N-term	99	1724.9794	2			

Peptide ID Hypotheses

Conf	Sc	Prot N	Sequence	Modifications	Theor MW	Theor m/z	z	ΔMass
99	16	744	SVPEIEALVAAVVER	iTRAQ@N-term	1724.9794	863.4970	2	-0.0060
< 1	7		APSVFTQVGGLGAVIPGR	No iTRAQ@N-term	1724.9573	863.4859	2	0.0161
< 1	7		NVAKLVEPPRTDSR	No iTRAQ@N-term, iTRAQ(...	1724.9655	863.4900	2	0.0079
< 1	7		PAAYVPLPIGNGEQR	iTRAQ@N-term	1724.9331	863.4738	2	0.0403
< 1	7		TALTLVAADALEVAPDR	No iTRAQ@N-term	1724.9308	863.4727	2	0.0426

Precursor MS Region

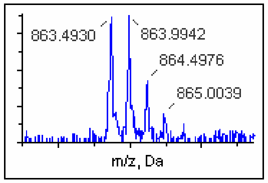

Fragmentation Evidence for Peptide

ITR SVPEIEALVAAVVER

Res	b	y
S	232.1414	1725.9867
V	331.2098	1494.8526
P	428.2625	1395.7842
E	557.3051	1298.7314
I	670.3892	1169.6888
E	799.4318	1056.6048
A	870.4689	927.5622
L	983.5530	856.5251
V	1082.6214	743.4410
A	1153.6585	644.3726
A	1224.6956	573.3355
V	1323.7640	502.2984
V	1422.8324	403.2300
E	1551.8750	304.1615
R	1707.9761	175.1190

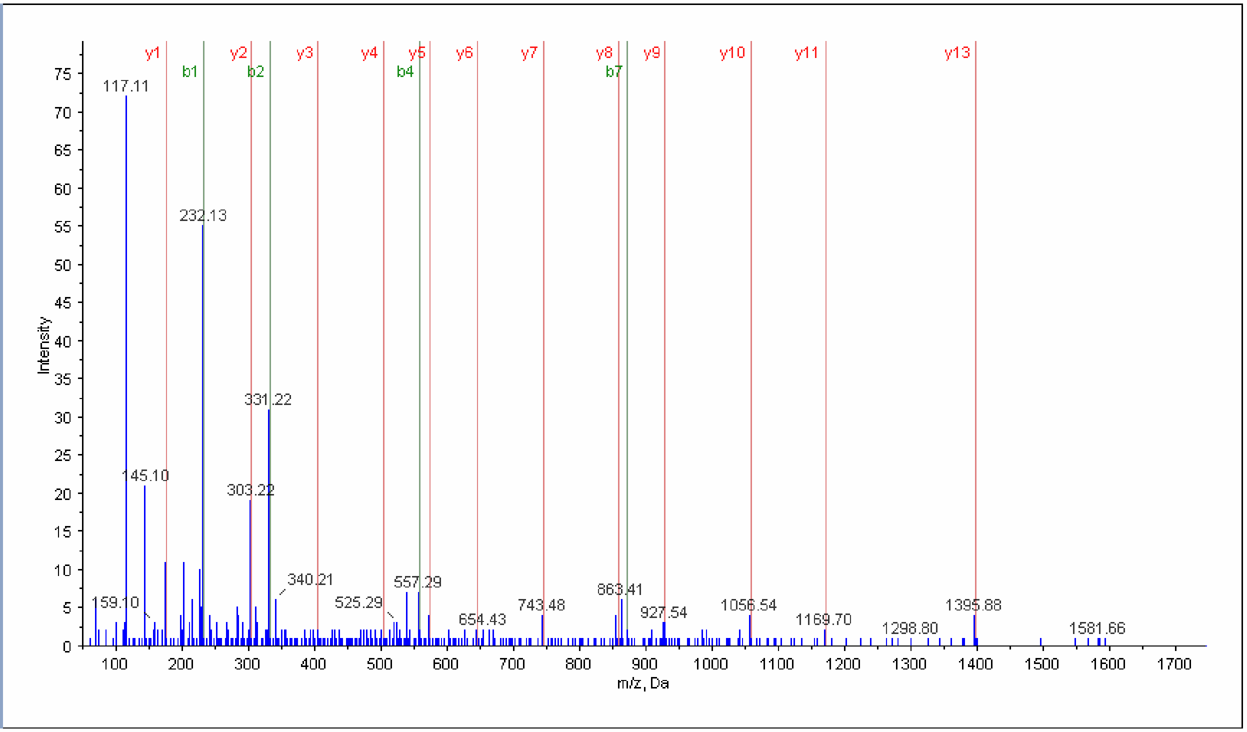

Protein: SCO5086, ActII [3 of 18]

Protein Quant | Protein ID | Spectra | Summary Statistics

Spectrum List

Spectrum	Time	Prec MW	Prec m/z	Prec z	Best Sequence	Modifications	Conf	Theor MW	z	115:114	%Err 115:114	116:
20.1.1.1092.2	59.741	1724.9617	575.9945	3	SVPEIEALVAAVVER	iTRAQ@N-term	99	1724.9794	3			

Peptide ID Hypotheses

Conf	Sc	Prot N	Sequence	Modifications	Theor MW	Theor m/z	z	ΔMass
99	15	744	SVPEIEALVAAVVER	iTRAQ@N-term	1724.9794	576.0004	3	-0.0177
< 1	5	211	ISGGGVSGQAGALRLGVAR	No iTRAQ@N-term	1724.9645	575.9954	3	-0.0028
< 1	8		PAAYVPLPIGNGEQR	iTRAQ@N-term	1724.9331	575.9850	3	0.0286
< 1	8		TALTLVAADALEVAPDR	No iTRAQ@N-term	1724.9308	575.9842	3	0.0309
< 1	7		AISLGMPDVPTRLAER	No iTRAQ@N-term	1724.9243	575.9820	3	0.0374

Precursor MS Region

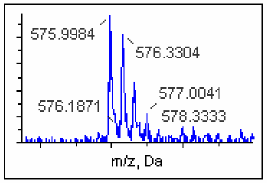

Fragmentation Evidence for Peptide

ITR SVPEIEALVAAVVER

Res	b	y
S	232.1414	1725.9867
V	331.2098	1494.8526
P	428.2625	1395.7842
E	557.3051	1298.7314
I	670.3892	1169.6888
E	799.4318	1056.6048
A	870.4689	927.5622
L	983.5530	856.5251
V	1082.6214	743.4410
A	1153.6585	644.3726
A	1224.6956	573.3355
V	1323.7640	502.2984
V	1422.8324	403.2300
E	1551.8750	304.1615
R	1707.9761	175.1190

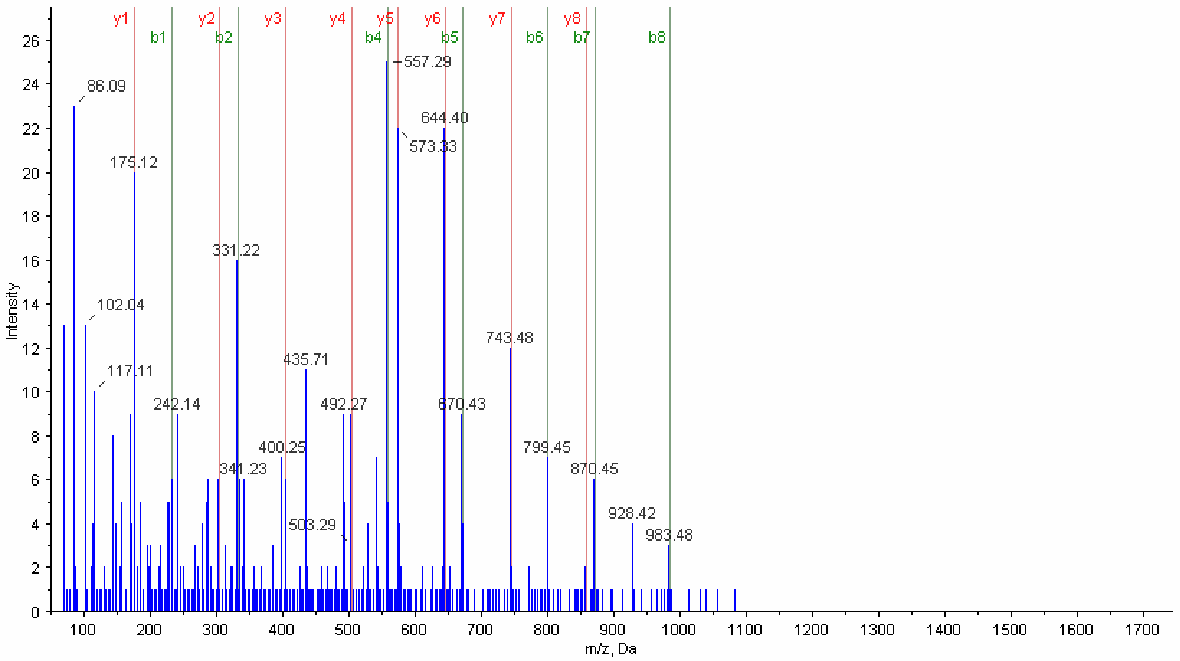

Protein: SCO5086, ActII [4 of 18]

Protein Quant | Protein ID | Spectra | Summary Statistics

Spectrum List

Spectrum	Time	Prec MW	Prec m/z	Prec z	Best Sequence	Modifications	Conf	Theor MW	z	115:114	%Err 115:114	116:
20.1.1.1093.3	60.023	1724.9741	863.4944	2	SVPEIEALVAAVVER	iTRAQ@N-term	99	1724.9794	2			

Peptide ID Hypotheses

Conf	Sc	Prot N	Sequence	Modifications	Theor MW	Theor m/z	z	ΔMass
99	13	744	SVPEIEALVAAVVER	iTRAQ@N-term	1724.9794	863.4970	2	-0.0053
< 1	6		LVARSNADLQRVIDR	No iTRAQ@N-term	1724.9645	863.4895	2	0.0096
< 1	6		NVAKLVEPPRTDSR	No iTRAQ@N-term, iTRAQ(...	1724.9655	863.4900	2	0.0086
< 1	6		RLDGEVPQEILRR	iTRAQ@N-term, Deamidatio...	1724.9655	863.4900	2	0.0086
< 1	6		VPEELSARVPAEQR	iTRAQ@N-term, Deamidatio...	1724.9179	863.4662	2	0.0563

Precursor MS Region

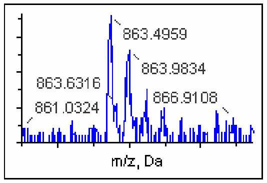

Fragmentation Evidence for Peptide

ITR SVPEIEALVAAVVER

Res	b	y
S	232.1414	1725.9867
V	331.2098	1494.8526
P	428.2625	1395.7842
E	557.3051	1298.7314
I	670.3892	1169.6888
E	799.4318	1056.6048
A	870.4689	927.5622
L	983.5530	856.5251
V	1082.6214	743.4410
A	1153.6585	644.3726
A	1224.6956	573.3355
V	1323.7640	502.2984
V	1422.8324	403.2300
E	1551.8750	304.1615
R	1707.9761	175.1190

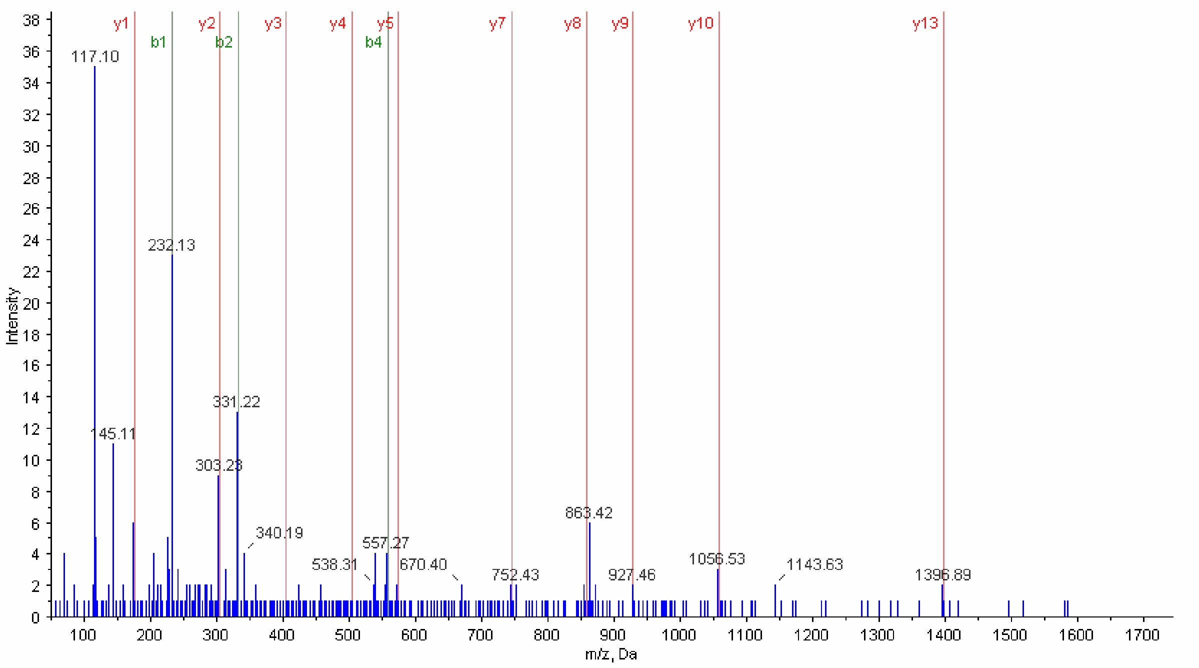

Protein: SCO5086, ActII [5 of 18]

Protein Quant | Protein ID | Spectra | Summary Statistics

Spectrum List

Spectrum	Time	Prec MW	Prec m/z	Prec z	Best Sequence	Modifications	Conf	Theor MW	z	115:114	%Err 115:114	116:
21.1.1.1071.4	58.789	1724.9670	575.9963	3	SVPEIEALVAAVVER	iTRAQ@N-term	99	1724.9795	3			

Peptide ID Hypotheses

Conf	Sc	Prot N	Sequence	Modifications	Theor MW	Theor m/z	z	ΔMass
99	15	744	SVPEIEALVAAVVER	iTRAQ@N-term	1724.9795	576.0004	3	-0.0125
< 1	9		AISLGMPDVPTRLAER	No iTRAQ@N-term	1724.9244	575.9821	3	0.0426
< 1	8		TAAGKPVANRELVVR	No iTRAQ@N-term, iTRAQ(...	1725.0020	576.0079	3	-0.0350
< 1	7		TALTLVAADALEVAPDR	No iTRAQ@N-term	1724.9310	575.9842	3	0.0361
< 1	6		ASTPGSYTFILPATR	iTRAQ@N-term	1724.9220	575.9813	3	0.0450

Precursor MS Region

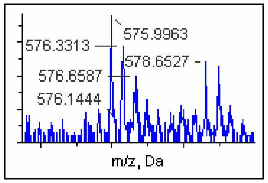

Fragmentation Evidence for Peptide

ITR SVPEIEALVAAVVER

Res	b	y
S	232.1414	1725.9867
V	331.2098	1494.8526
P	428.2625	1395.7842
E	557.3051	1298.7314
I	670.3892	1169.6888
E	799.4318	1056.6048
A	870.4689	927.5622
L	983.5530	856.5251
V	1082.6214	743.4410
A	1153.6585	644.3726
A	1224.6956	573.3355
V	1323.7640	502.2984
V	1422.8324	403.2300
E	1551.8750	304.1615
R	1707.9761	175.1190

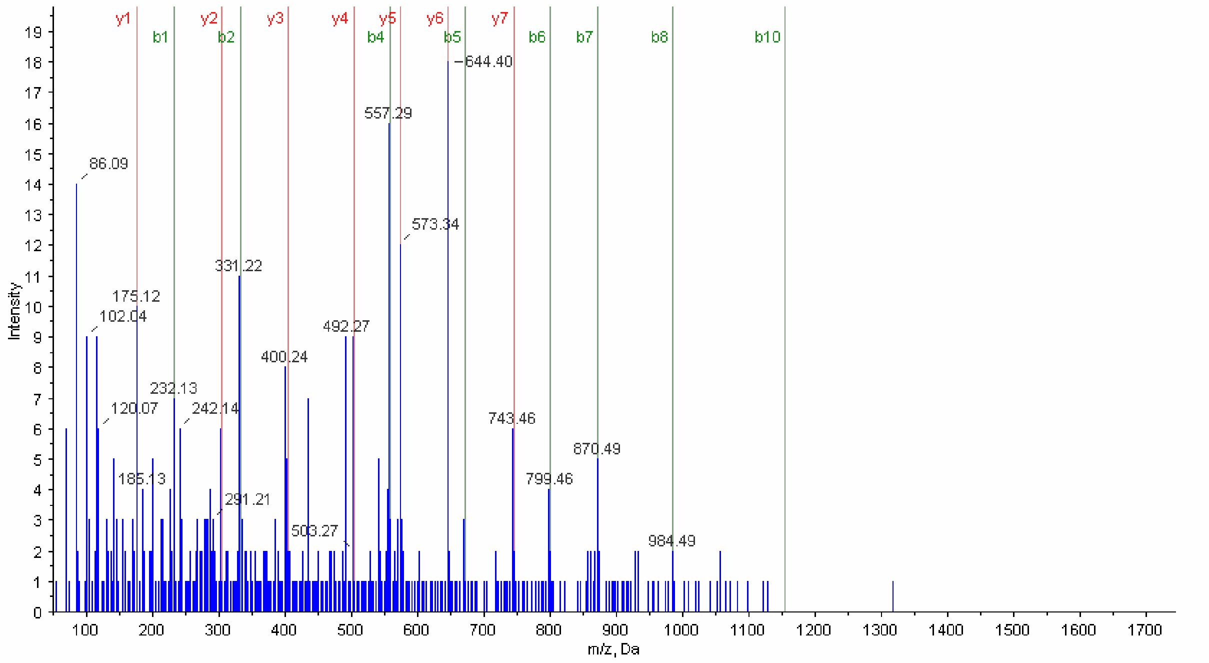

Protein: SCO5086, ActII [6 of 18]

Protein Quant | Protein ID | Spectra | Summary Statistics

Spectrum List

Spectrum	Time	Prec MW	Prec m/z	Prec z	Best Sequence	Modifications	Conf	Theor MW	z	115:114	%Err 115:114	116:
22.1.1.1100.5	59.732	1724.9868	576.0029	3	SVPEIEALVAAVVER	iTRAQ@N-term	99	1724.9794	3			

Peptide ID Hypotheses

Conf	Sc	Prot N	Sequence	Modifications	Theor MW	Theor m/z	z	ΔMass
99	14	744	SVPEIEALVAAVVER	iTRAQ@N-term	1724.9794	576.0004	3	0.0074
< 1	7		QLADGVRVVAAGEALL	iTRAQ@N-term	1724.9907	576.0041	3	-0.0038
< 1	7		RTGLMWVNPLLLRR	No iTRAQ@N-term, Deamid...	1724.9872	576.0030	3	-0.0004
< 1	6		TALTLVAADALEVAPDR	No iTRAQ@N-term	1724.9309	575.9842	3	0.0560
< 1	6		VPEELSARVPAEQR	iTRAQ@N-term, Deamidatio...	1724.9179	575.9799	3	0.0689

Precursor MS Region

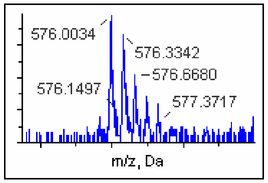

Fragmentation Evidence for Peptide

ITR SVPEIEALVAAVVER

Res	b	y
S	**232.1414**	1725.9867
V	**331.2098**	1494.8526
P	428.2625	1395.7842
E	**557.3051**	1298.7314
I	**670.3892**	1169.6888
E	**799.4318**	1056.6048
A	**870.4689**	927.5622
L	**983.5530**	856.5251
V	1082.6214	**743.4410**
A	1153.6585	**644.3726**
A	1224.6956	**573.3355**
V	1323.7640	**502.2984**
V	1422.8324	**403.2300**
E	1551.8750	**304.1615**
R	1707.9761	**175.1190**

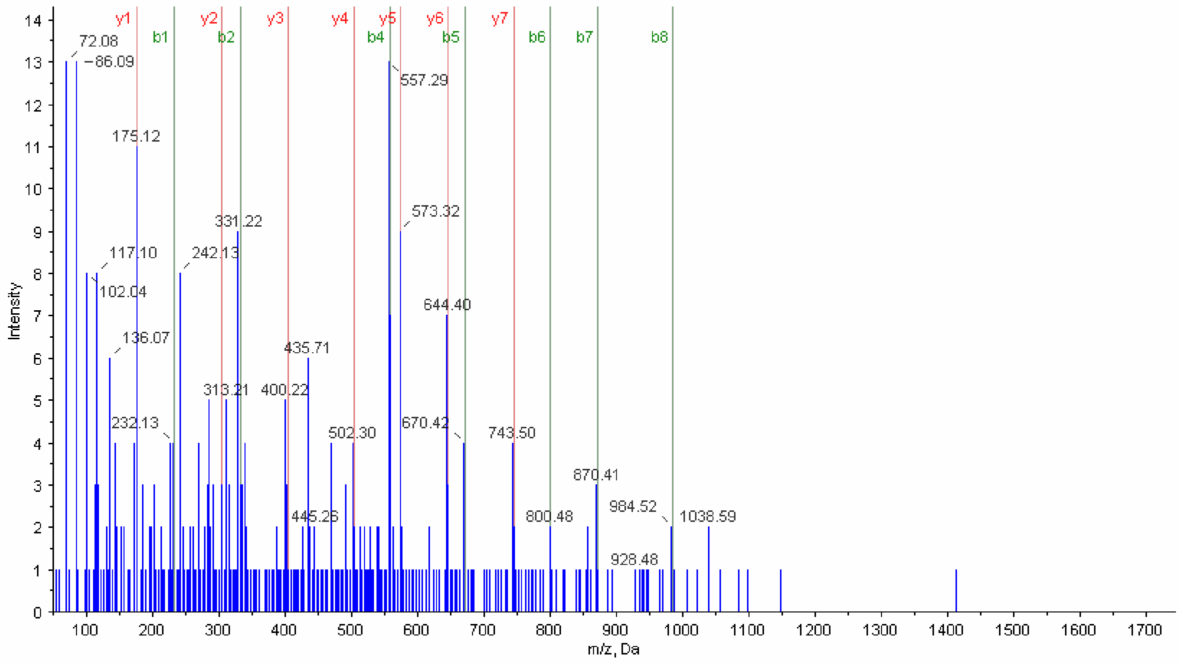

Protein: SCO5086, ActII [7 of 18]

Protein Quant | Protein ID | Spectra | Summary Statistics

Spectrum List

Spectrum	Time	Prec MW	Prec m/z	Prec z	Best Sequence	Modifications	Conf	Theor MW	z	115:114	%Err 115:114	116:
23.1.1.1108.2	59.630	1724.9532	575.9917	3	SVPEIEALVAAVVER	iTRAQ@N-term	99	1724.9795	3	0.7584	31.11	

Peptide ID Hypotheses

Conf	Sc	Prot N	Sequence	Modifications	Theor MW	Theor m/z	z	ΔMass
99	14	744	SVPEIEALVAAVVER	iTRAQ@N-term	1724.9795	576.0004	3	-0.0262
< 1	8	211	ISGGGVSGQAGALRLGVAR	No iTRAQ@N-term	1724.9646	575.9955	3	-0.0113
< 1	6		EQQLIADQARLRER	No iTRAQ@N-term	1724.9282	575.9834	3	0.0250
< 1	6		RAAGAVVAVGQVDGRR	iTRAQ@N-term	1724.9880	576.0033	3	-0.0348
< 1	6		VPEELSARVPAEQR	iTRAQ@N-term, Deamidatio...	1724.9179	575.9799	3	0.0353

Precursor MS Region

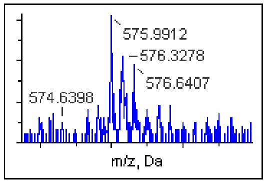

Fragmentation Evidence for Peptide

ITR SVPEIEALVAAVVER

Res	b	y
S	232.1414	1725.9867
V	331.2098	1494.8526
P	428.2625	1395.7842
E	557.3051	1298.7314
I	670.3892	1169.6888
E	799.4318	1056.6048
A	870.4689	927.5622
L	983.5530	856.5251
V	1082.6214	743.4410
A	1153.6585	644.3726
A	1224.6956	573.3355
V	1323.7640	502.2984
V	1422.8324	403.2300
E	1551.8750	304.1615
R	1707.9761	175.1190

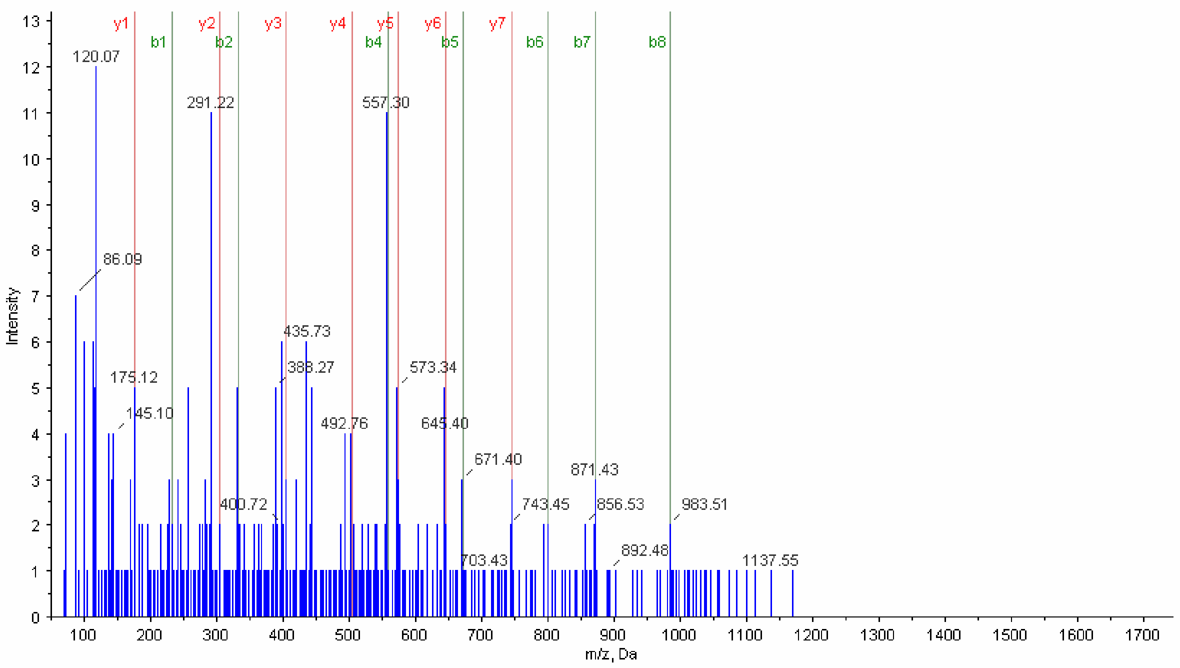

Protein: SCO5086, ActII [8 of 18]

Protein Quant | Protein ID | Spectra | Summary Statistics

Spectrum List

Spectrum	Time	Prec MW	Prec m/z	Prec z	Best Sequence	Modifications	Conf	Theor MW	z	115:114	%Err 115:114	116:
3.1.1.1112.2	59.778	1724.9719	575.9979	3	SVPEIEALVAAVVER	iTRAQ@N-term	99	1724.9793	3			

Peptide ID Hypotheses

Conf	Sc	Prot N	Sequence	Modifications	Theor MW	Theor m/z	z	ΔMass
99	16	744	SVPEIEALVAAVVER	iTRAQ@N-term	1724.9793	576.0004	3	-0.0074
< 1	7	211	ISGGGVSGQAGALRLGVAR	No iTRAQ@N-term	1724.9645	575.9954	3	0.0074
< 1	9		TALTLVAADALEVAPDR	No iTRAQ@N-term	1724.9308	575.9842	3	0.0411
< 1	8		PAAYVPLPIGNGEQR	iTRAQ@N-term	1724.9331	575.9850	3	0.0389
< 1	7		GVLAEMVRVLKPGGR	No iTRAQ@N-term, iTRAQ(...	1725.0204	576.0141	3	-0.0485

Precursor MS Region

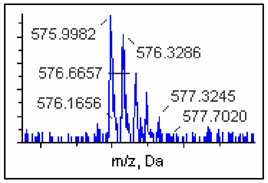

Fragmentation Evidence for Peptide

ITR SVPEIEALVAAVVER

Res	b	y
S	232.1414	1725.9867
V	331.2098	1494.8526
P	428.2625	1395.7842
E	557.3051	1298.7314
I	670.3892	1169.6888
E	799.4318	1056.6048
A	870.4689	927.5622
L	983.5530	856.5251
V	1082.6214	743.4410
A	1153.6585	644.3726
A	1224.6956	573.3355
V	1323.7640	502.2984
V	1422.8324	403.2300
E	1551.8750	304.1615
R	1707.9761	175.1190

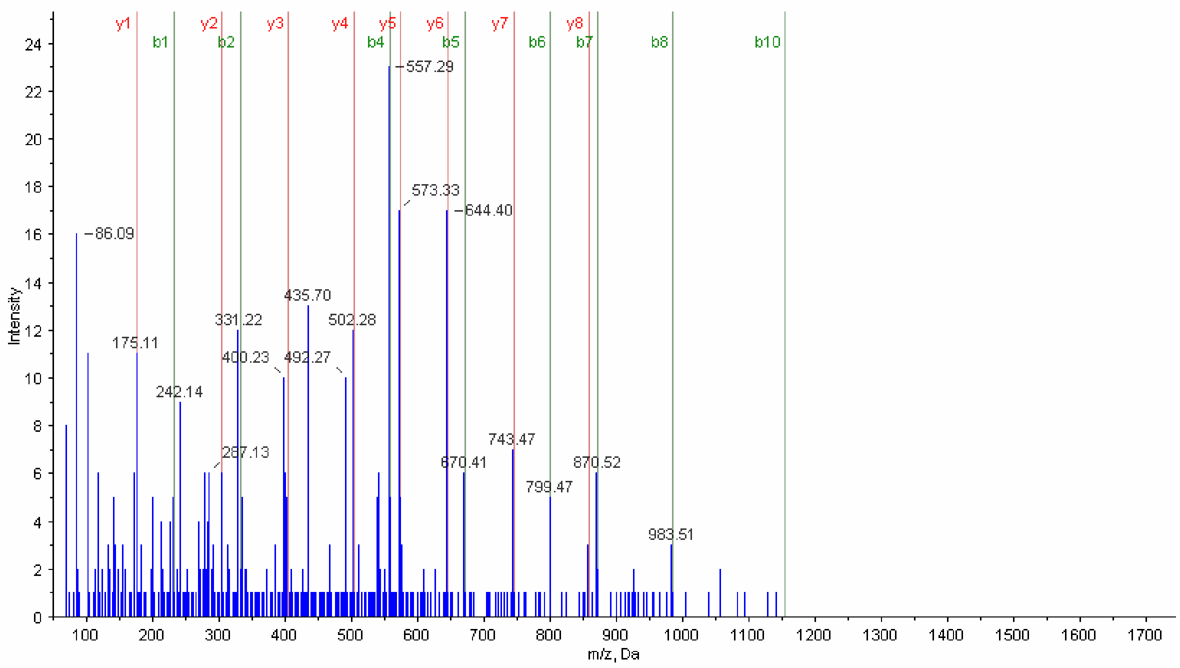

Protein: SCO5086, ActII [9 of 18]

Protein Quant | Protein ID | Spectra | Summary Statistics

Spectrum List

Spectrum	Time	Prec MW	Prec m/z	Prec z	Best Sequence	Modifications	Conf	Theor MW	z	115:114	%Err 115:114	116:
17.1.1.1639.2	59.725	1724.9780	575.9999	3	SVPEIEALVAAVVER	iTRAQ@N-term	99	1724.9795	3			

Peptide ID Hypotheses

Conf	Sc	Prot N	Sequence	Modifications	Theor MW	Theor m/z	z	ΔMass
99	14	744	SVPEIEALVAAVVER	iTRAQ@N-term	1724.9795	576.0004	3	-0.0015
< 1	7		AISLGMPDVPTRLAER	No iTRAQ@N-term	1724.9244	575.9821	3	0.0536
< 1	7		PAAYVPLPIGNGEQR	iTRAQ@N-term	1724.9333	575.9850	3	0.0448
< 1	7		TALTLVAADALEVAPDR	No iTRAQ@N-term	1724.9310	575.9842	3	0.0471
< 1	6		RKLTDGPAPAEPSSR	No iTRAQ@N-term, iTRAQ(...	1724.9292	575.9837	3	0.0488

Precursor MS Region

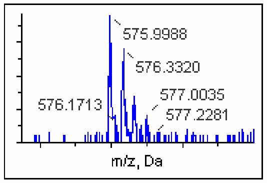

Fragmentation Evidence for Peptide

ITR SVPEIEALVAAVVER

Res	b	y
S	232.1414	1725.9867
V	331.2098	1494.8526
P	428.2625	1395.7842
E	557.3051	1298.7314
I	670.3892	1169.6888
E	799.4318	1056.6048
A	870.4689	927.5622
L	983.5530	856.5251
V	1082.6214	743.4410
A	1153.6585	644.3726
A	1224.6956	573.3355
V	1323.7640	502.2984
V	1422.8324	403.2300
E	1551.8750	304.1615
R	1707.9761	175.1190

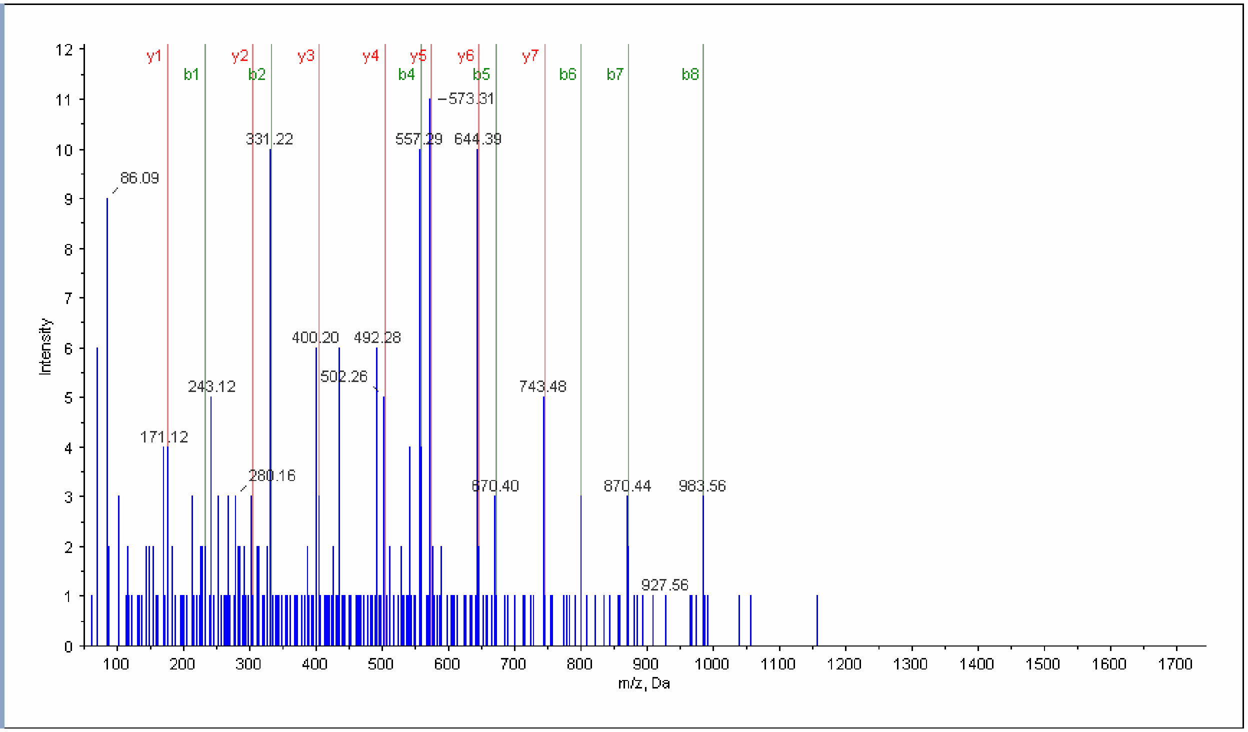

Protein: SCO5086, ActII [10 of 18]

Protein Quant | Protein ID | Spectra | Summary Statistics

Spectrum List

Spectrum	Time	Prec MW	Prec m/z	Prec z	Best Sequence	Modifications	Conf	Theor MW	z	115:114	%Err 115:114	116:
20.1.1.1097.2	60.894	1724.9879	576.0032	3	SVPEIEALVAAVVER	iTRAQ@N-term	99	1724.9794	3			

Peptide ID Hypotheses

Conf	Sc	Prot N	Sequence	Modifications	Theor MW	Theor m/z	z	ΔMass
99	14	744	SVPEIEALVAAVVER	iTRAQ@N-term	1724.9794	576.0004	3	0.0085
< 1	8		AISLGMPDVPTRLAER	No iTRAQ@N-term	1724.9243	575.9820	3	0.0636
< 1	7		PAAYVPLPIGNGEQR	iTRAQ@N-term	1724.9332	575.9850	3	0.0548
< 1	6		GVLAEMVRVLKPGGR	No iTRAQ@N-term, iTRAQ(...	1725.0205	576.0141	3	-0.0326
< 1	6		TALTLVAADALEVAPDR	No iTRAQ@N-term	1724.9309	575.9842	3	0.0571

Precursor MS Region

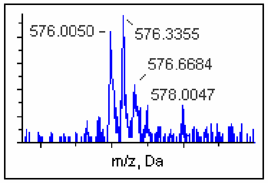

Fragmentation Evidence for Peptide

ITR SVPEIEALVAAVVER

Res	b	y
S	232.1414	1725.9867
V	331.2098	1494.8526
P	428.2625	1395.7842
E	557.3051	1298.7314
I	670.3892	1169.6888
E	799.4318	1056.6048
A	870.4689	927.5622
L	983.5530	856.5251
V	1082.6214	743.4410
A	1153.6585	644.3726
A	1224.6956	573.3355
V	1323.7640	502.2984
V	1422.8324	403.2300
E	1551.8750	304.1615
R	1707.9761	175.1190

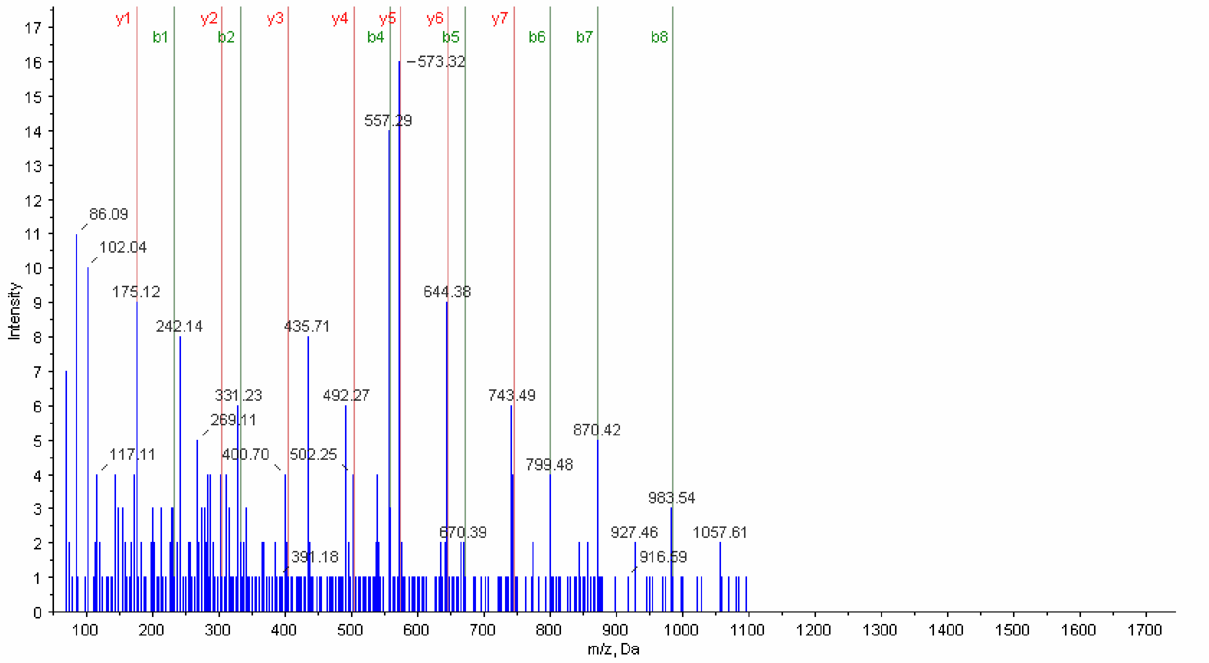

56/81

Protein: SCO5086, ActII [11 of 18]

Protein Quant | Protein ID | Spectra | Summary Statistics

Spectrum List

Spectrum	Time	Prec MW	Prec m/z	Prec z	Best Sequence	Modifications	Conf	Theor MW	z	115:114	%Err 115:114	116:
20.1.1.1102.2	62.047	1724.9843	576.0020	3	SVPEIEALVAAVVER	iTRAQ@N-term	99	1724.9794	3			

Peptide ID Hypotheses

Conf	Sc	Prot N	Sequence	Modifications	Theor MW	Theor m/z	z	ΔMass
99	13	744	SVPEIEALVAAVVER	iTRAQ@N-term	1724.9794	576.0004	3	0.0048
< 1	6	211	ISGGGVSGQAGALRLGVAR	No iTRAQ@N-term	1724.9646	575.9955	3	0.0197
< 1	7		TALTLVAADALEVAPDR	No iTRAQ@N-term	1724.9309	575.9842	3	0.0534
< 1	6		ASTPGSYTFILPATR	iTRAQ@N-term	1724.9219	575.9812	3	0.0623
< 1	6		GVLAEMVRVLKPGGR	No iTRAQ@N-term, iTRAQ(...	1725.0205	576.0141	3	-0.0363

Precursor MS Region

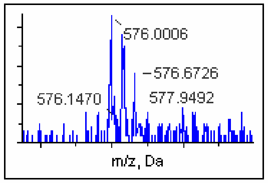

Fragmentation Evidence for Peptide

ITR SVPEIEALVAAVVER

Res	b	y
S	**232.1414**	1725.9867
V	**331.2098**	1494.8526
P	428.2625	1395.7842
E	**557.3051**	1298.7314
I	**670.3892**	1169.6888
E	**799.4318**	1056.6048
A	**870.4689**	927.5622
L	**983.5530**	856.5251
V	1082.6214	**743.4410**
A	1153.6585	**644.3726**
A	1224.6956	**573.3355**
V	1323.7640	**502.2984**
V	1422.8324	**403.2300**
E	1551.8750	304.1615
R	1707.9761	**175.1190**

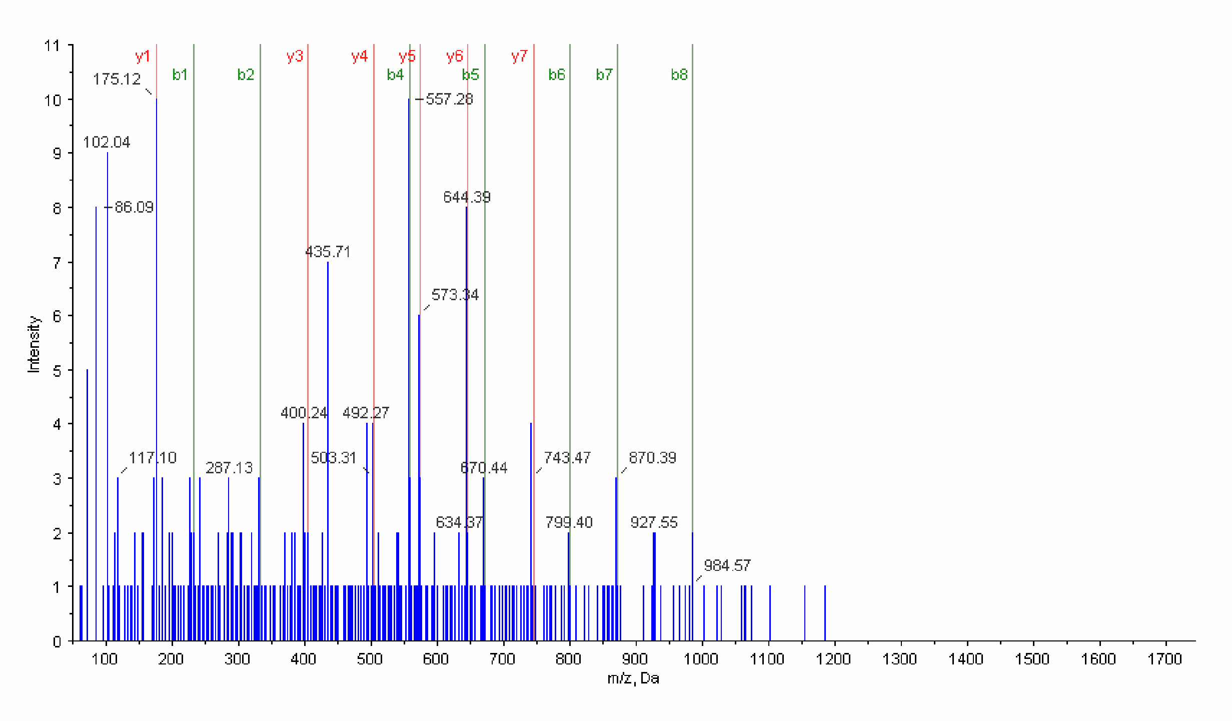

Protein: SCO5086, ActII [12 of 18]

Protein Quant | Protein ID | Spectra | Summary Statistics

Spectrum List

Spectrum	Time	Prec MW	Prec m/z	Prec z	Best Sequence	Modifications	Conf	Theor MW	z	115:114	%Err 115:114	116:
20.1.1.1119.3	65.915	1724.9785	576.0001	3	SVPEIEALVAAVVER	iTRAQ@N-term	99	1724.9794	3			

Peptide ID Hypotheses

Conf	Sc	Prot N	Sequence	Modifications	Theor MW	Theor m/z	z	ΔMass
99	13	744	SVPEIEALVAAVVER	iTRAQ@N-term	1724.9794	576.0004	3	-0.0009
< 1	4	211	ISGGGVSGQAGALRLGVAR	No iTRAQ@N-term	1724.9645	575.9954	3	0.0140
< 1	6		GVLAEMVRVLKPGGR	No iTRAQ@N-term, iTRAQ(...	1725.0205	576.0141	3	-0.0420
< 1	6		TALTLVAADALEVAPDR	No iTRAQ@N-term	1724.9308	575.9842	3	0.0477
< 1	6		VPEELSARVPAEQR	iTRAQ@N-term, Deamidatio...	1724.9178	575.9799	3	0.0607

Precursor MS Region

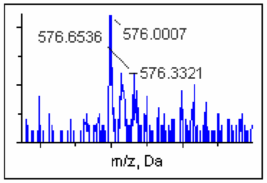

Fragmentation Evidence for Peptide

ITR SVPEIEALVAAVVER

Res	b	y
S	232.1414	1725.9867
V	331.2098	1494.8526
P	428.2625	1395.7842
E	557.3051	1298.7314
I	670.3892	1169.6888
E	799.4318	1056.6048
A	870.4689	927.5622
L	983.5530	856.5251
V	1082.6214	743.4410
A	1153.6585	644.3726
A	1224.6956	573.3355
V	1323.7640	502.2984
V	1422.8324	403.2300
E	1551.8750	304.1615
R	1707.9761	175.1190

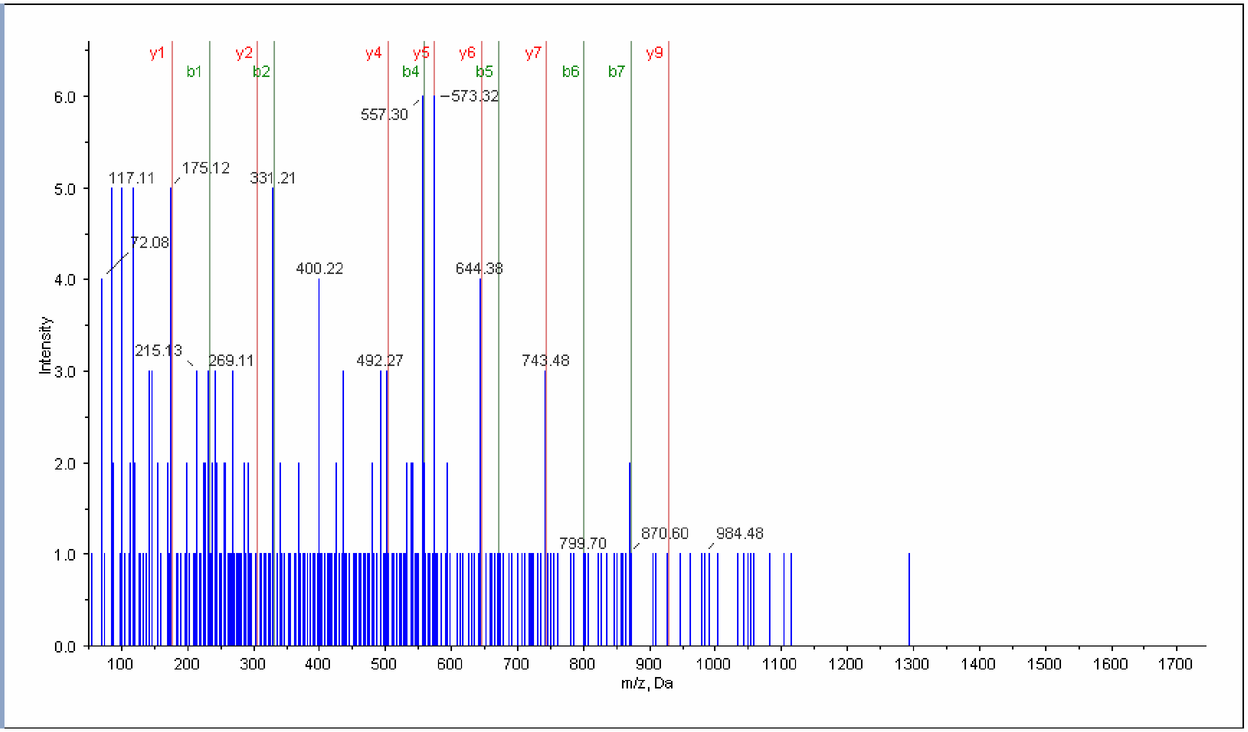

Protein: SCO5086, ActII [13 of 18]

Protein Quant | Protein ID | Spectra | Summary Statistics

Spectrum List

Spectrum	Time	Prec MW	Prec m/z	Prec z	Best Sequence	Modifications	Conf	Theor MW	z	115:114	%Err 115:114	116:
21.1.1.1076.2	59.839	1724.9785	576.0001	3	SVPEIEALVAAVVER	iTRAQ@N-term	99	1724.9795	3			

Peptide ID Hypotheses

Conf	Sc	Prot N	Sequence	Modifications	Theor MW	Theor m/z	z	ΔMass
99	15	744	SVPEIEALVAAVVER	iTRAQ@N-term	1724.9795	576.0004	3	-0.0010
< 1	6		ARRSVVVAVAALDRV	iTRAQ@N-term	1725.0496	576.0238	3	-0.0710
< 1	6		PTGVEVVVADLSEGIP	iTRAQ@N-term	1724.9319	575.9846	3	0.0467
< 1	6		TALTLVAADALEVAPDR	No iTRAQ@N-term	1724.9309	575.9842	3	0.0476
< 1	5		RATLNRLLFPGPAR	iTRAQ@N-term	1725.0284	576.0168	3	-0.0499

Precursor MS Region

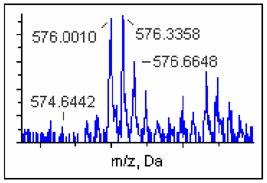

Fragmentation Evidence for Peptide

ITR SVPEIEALVAAVVER

Res	b	y
S	232.1414	1725.9867
V	331.2098	1494.8526
P	428.2625	1395.7842
E	557.3051	1298.7314
I	670.3892	1169.6888
E	799.4318	1056.6048
A	870.4689	927.5622
L	983.5530	856.5251
V	1082.6214	743.4410
A	1153.6585	644.3726
A	1224.6956	573.3355
V	1323.7640	502.2984
V	1422.8324	403.2300
E	1551.8750	304.1615
R	1707.9761	175.1190

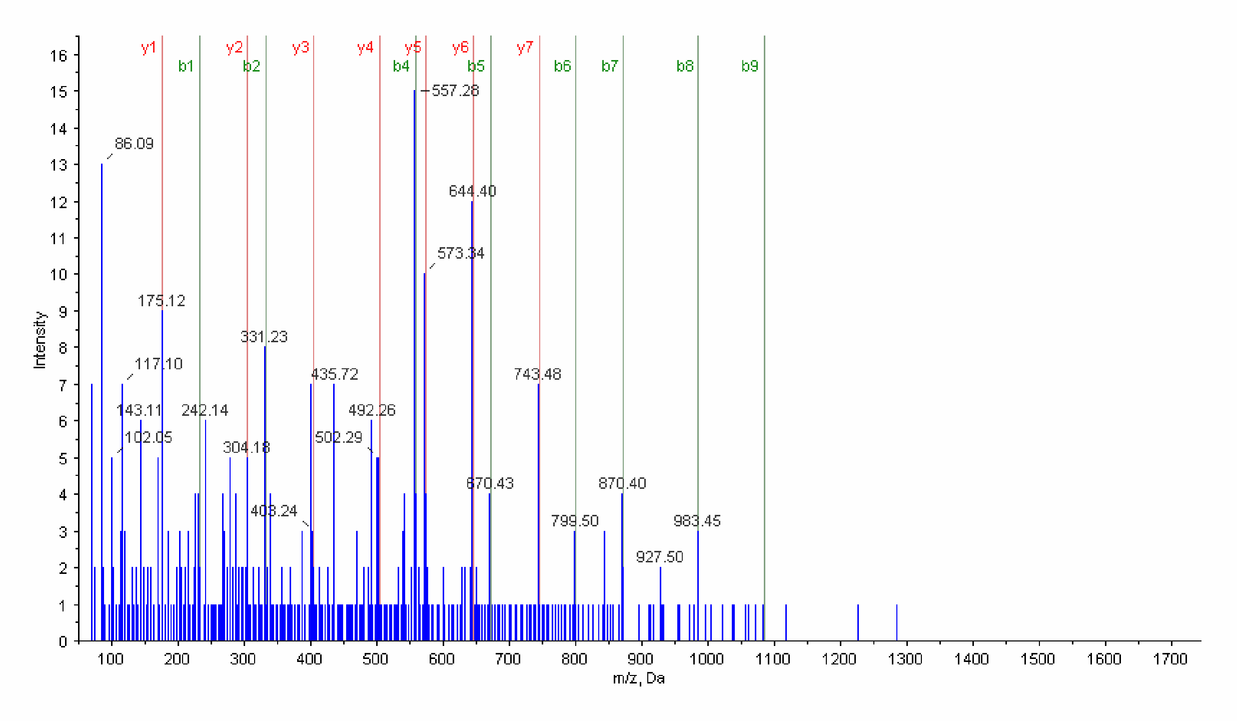

Protein: SCO5086, ActII [14 of 18]

Protein Quant | Protein ID | Spectra | Summary Statistics

Spectrum List

Spectrum	Time	Prec MW	Prec m/z	Prec z	Best Sequence	Modifications	Conf	Theor MW	z	115:114	%Err 115:114	116:
22.1.1.1101.4	59.911	1724.9763	575.9994	3	SVPEIEALVAAVVER	iTRAQ@N-term	99	1724.9794	3			

Peptide ID Hypotheses

Conf	Sc	Prot N	Sequence	Modifications	Theor MW	Theor m/z	z	ΔMass
99	15	744	SVPEIEALVAAVVER	iTRAQ@N-term	1724.9794	576.0004	3	-0.0030
< 1	6	211	ISGGGVSGQAGALRLGVAR	No iTRAQ@N-term	1724.9645	575.9954	3	0.0118
< 1	8		AISLGMPDVPTRLAER	No iTRAQ@N-term	1724.9243	575.9820	3	0.0521
< 1	7		QLADGVRVVAAGEALL	iTRAQ@N-term	1724.9906	576.0041	3	-0.0143
< 1	6		APSVFTQVGGLGAVIPGR	No iTRAQ@N-term	1724.9573	575.9930	3	0.0190

Precursor MS Region

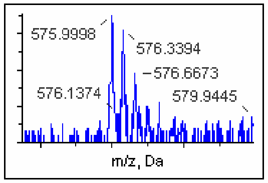

Fragmentation Evidence for Peptide

ITR SVPEIEALVAAVVER

Res	b	y
S	232.1414	1725.9867
V	331.2098	1494.8526
P	428.2625	1395.7842
E	557.3051	1298.7314
I	670.3892	1169.6888
E	799.4318	1056.6048
A	870.4689	927.5622
L	983.5530	856.5251
V	1082.6214	743.4410
A	1153.6585	644.3726
A	1224.6956	573.3355
V	1323.7640	502.2984
V	1422.8324	403.2300
E	1551.8750	304.1615
R	1707.9761	175.1190

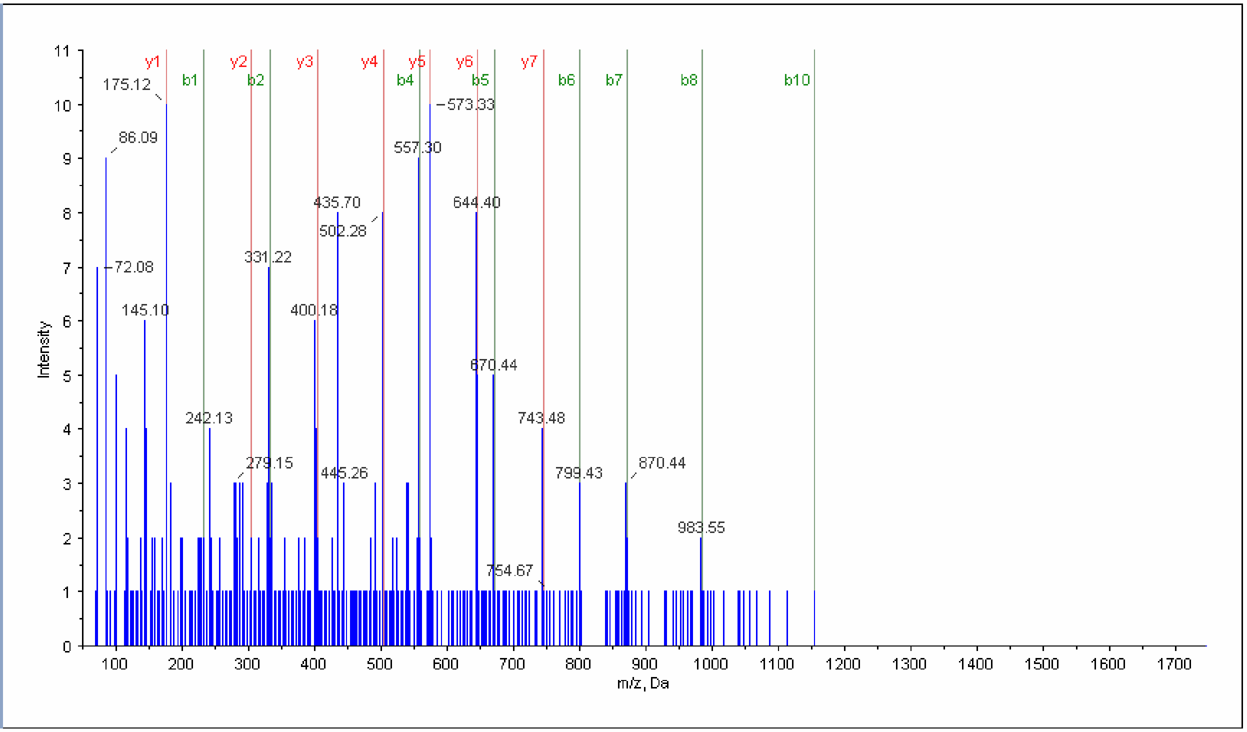

60/81

Protein: SCO5086, ActII [15 of 18]

Protein Quant | Protein ID | Spectra | Summary Statistics

Spectrum List

Spectrum	Time	Prec MW	Prec m/z	Prec z	Best Sequence	Modifications	Conf	Theor MW	z	115:114	%Err 115:114	116:
5.1.1.2158.3	51.444	1724.9735	863.4940	2	SVPEIEALVAAVVER	iTRAQ@N-term	99	1724.9795	2	0.5154	15.32	

Peptide ID Hypotheses

Conf	Sc	Prot N	Sequence	Modifications	Theor MW	Theor m/z	z	ΔMass
99	15	411	SVPEIEALVAAVVER	iTRAQ@N-term	1724.9795	863.4970	2	-0.0060
< 1	8	74	ISGGGVSGQAGALRLGVAR	No iTRAQ@N-term	1724.9646	863.4896	2	0.0089
< 1	9		TAAGKPVANRELVVR	No iTRAQ@N-term, iTRAQ(...	1725.0020	863.5083	2	-0.0285
< 1	9		TALTLVAADALEVAPDR	No iTRAQ@N-term	1724.9309	863.4727	2	0.0426
< 1	8		ASALAGERPGELVRR	iTRAQ@N-term	1724.9768	863.4957	2	-0.0033

Precursor MS Region

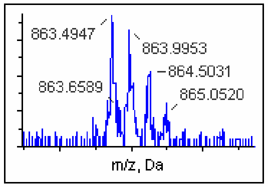

Fragmentation Evidence for Peptide

ITR SVPEIEALVAAVVER

Residue	b	y
S	232.1414	1725.9867
V	331.2098	1494.8526
P	428.2625	1395.7842
E	557.3051	1298.7314
I	670.3892	1169.6888
E	799.4318	1056.6048
A	870.4689	927.5622
L	983.5530	856.5251
V	1082.6214	743.4410
A	1153.6585	644.3726
A	1224.6956	573.3355
V	1323.7640	502.2984
V	1422.8324	403.2300
E	1551.8750	304.1615
R	1707.9761	175.1190

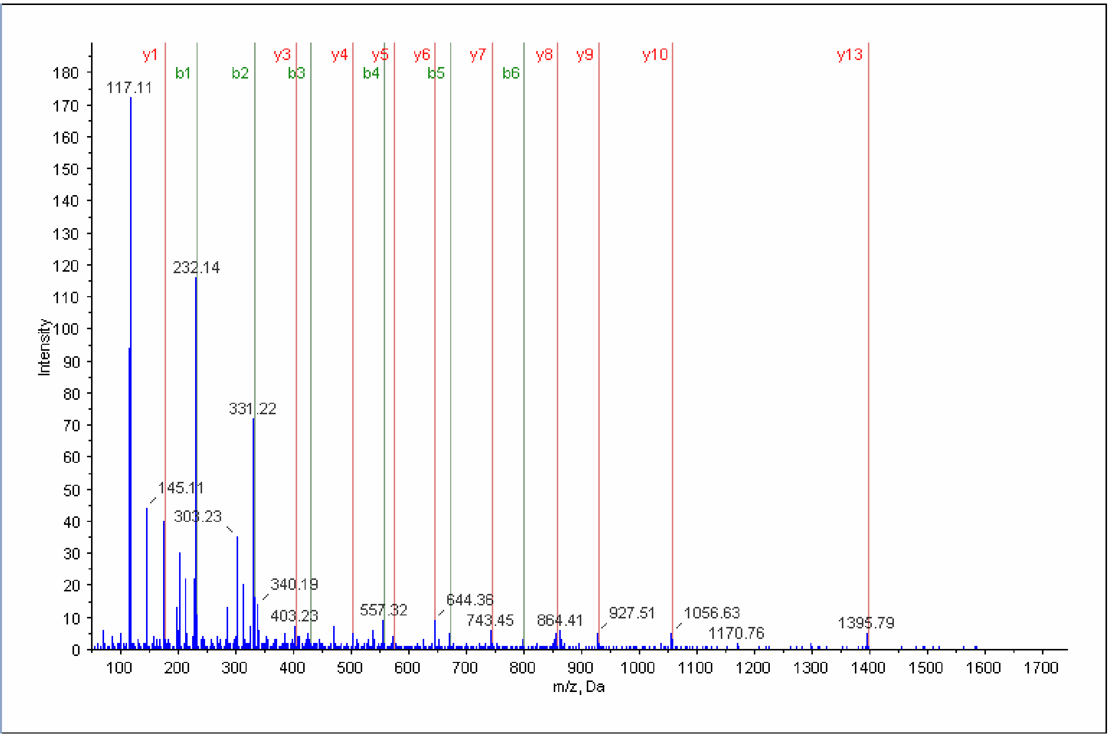

Protein: SCO5086, ActII [16 of 18]

Protein Quant | Protein ID | Spectra | Summary Statistics

Spectrum List

Spectrum	Time	Prec MW	Prec m/z	Prec z	Best Sequence	Modifications	Conf	Theor MW	z	115:114	%Err 115:114	116:
7.1.1.2173.2	51.301	0.0000	863.5010	0	SVPEIEALVAAVVER	iTRAQ@N-term	99	1724.9795	2	1.0282	10.23	

Peptide ID Hypotheses

Conf	Sc	Prot N	Sequence	Modifications	Theor MW	Theor m/z	z	ΔMass
99	16	411	SVPEIEALVAAVVER	iTRAQ@N-term	1724.9795	863.4970	2	0.0081
< 1	9		ASALAGERPGELVRR	iTRAQ@N-term	1724.9768	863.4957	2	0.0108
< 1	8		ASTPGSYTFILPATR	iTRAQ@N-term	1724.9219	863.4683	2	0.0656
< 1	8		NVAKLVEPPRTDSR	No iTRAQ@N-term, iTRAQ(...	1724.9655	863.4901	2	0.0220
< 1	8		TALTLVAADALEVAPDR	No iTRAQ@N-term	1724.9309	863.4727	2	0.0567

Precursor MS Region

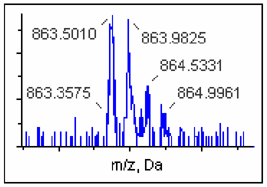

Fragmentation Evidence for Peptide

ITR SVPEIEALVAAVVER

Residue	b	y
S	**232.1414**	1725.9867
V	**331.2098**	1494.8526
P	**428.2625**	**1395.7842**
E	**557.3051**	1298.7314
I	**670.3892**	**1169.6888**
E	799.4318	**1056.6048**
A	870.4689	**927.5622**
L	983.5530	**856.5251**
V	**1082.6214**	**743.4410**
A	1153.6585	**644.3726**
A	1224.6956	**573.3355**
V	1323.7640	502.2984
V	1422.8324	**403.2300**
E	1551.8750	304.1615
R	1707.9761	**175.1190**

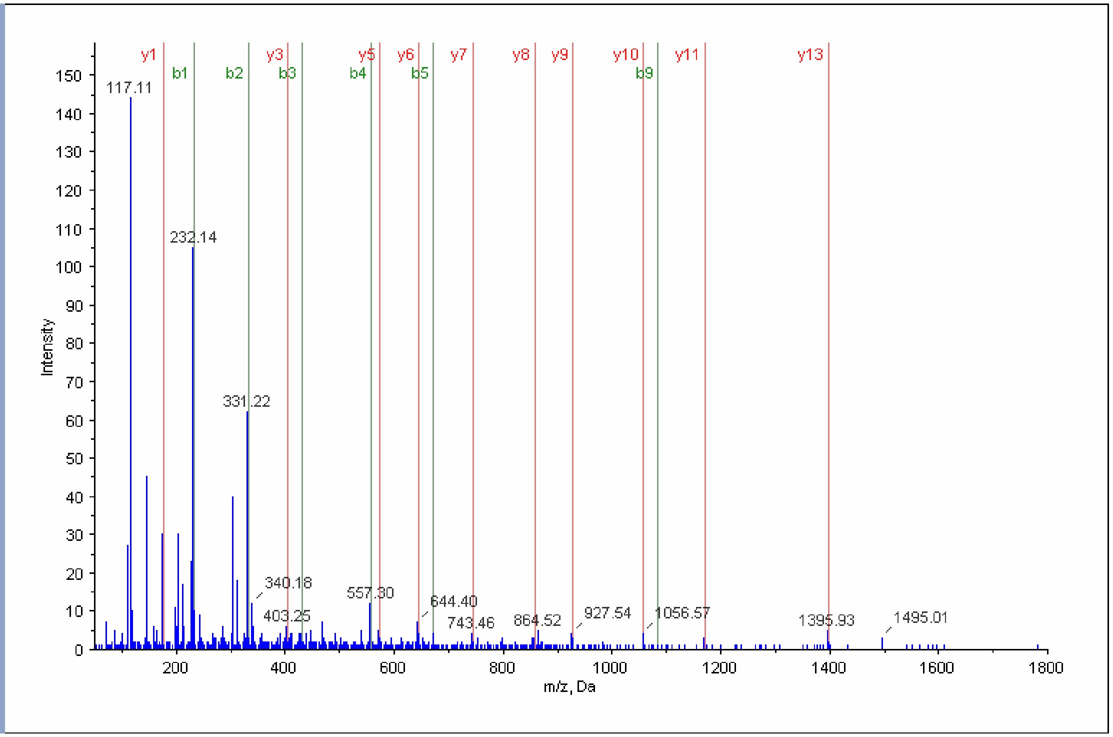

Protein: SCO5086, ActII [17 of 18]

Protein Quant | Protein ID | Spectra | Summary Statistics

Spectrum List

Spectrum	Time	Prec MW	Prec m/z	Prec z	Best Sequence	Modifications	Conf	Theor MW	z	115:114	%Err 115:114	116:
9.1.1.2132.2	51.334	0.0000	863.5089	0	SVPEIEALVAAVVER	iTRAQ@N-term	99	1724.9794	2	0.9699	8.08	

Peptide ID Hypotheses

Conf	Sc	Prot N	Sequence	Modifications	Theor MW	Theor m/z	z	ΔMass
99	15	411	SVPEIEALVAAVVER	iTRAQ@N-term	1724.9794	863.4970	2	0.0238
< 1	9		AAAASGADLAGLAHALRAVALDDPQR	No iTRAQ@N-term	2587.3254	863.4491	3	0.1793
< 1	8		EVVGADDPRVAAARR	iTRAQ@N-term	1724.9403	863.4774	2	0.0628
< 1	8		TAAGKPVANRELVVR	No iTRAQ@N-term, iTRAQ(...	1725.0019	863.5082	2	0.0013
< 1	8		TALTLVAADALEVAPDR	No iTRAQ@N-term	1724.9308	863.4727	2	0.0723

Precursor MS Region

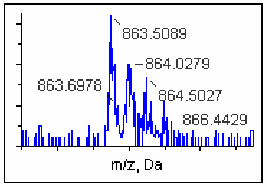

Fragmentation Evidence for Peptide

ITR SVPEIEALVAAVVER

Residue	b	y
S	232.1414	1725.9867
V	331.2098	1494.8526
P	428.2625	1395.7842
E	557.3051	1298.7314
I	670.3892	1169.6888
E	799.4318	1056.6048
A	870.4689	927.5622
L	983.5530	856.5251
V	1082.6214	743.4410
A	1153.6585	644.3726
A	1224.6956	573.3355
V	1323.7640	502.2984
V	1422.8324	403.2300
E	1551.8750	304.1615
R	1707.9761	175.1190

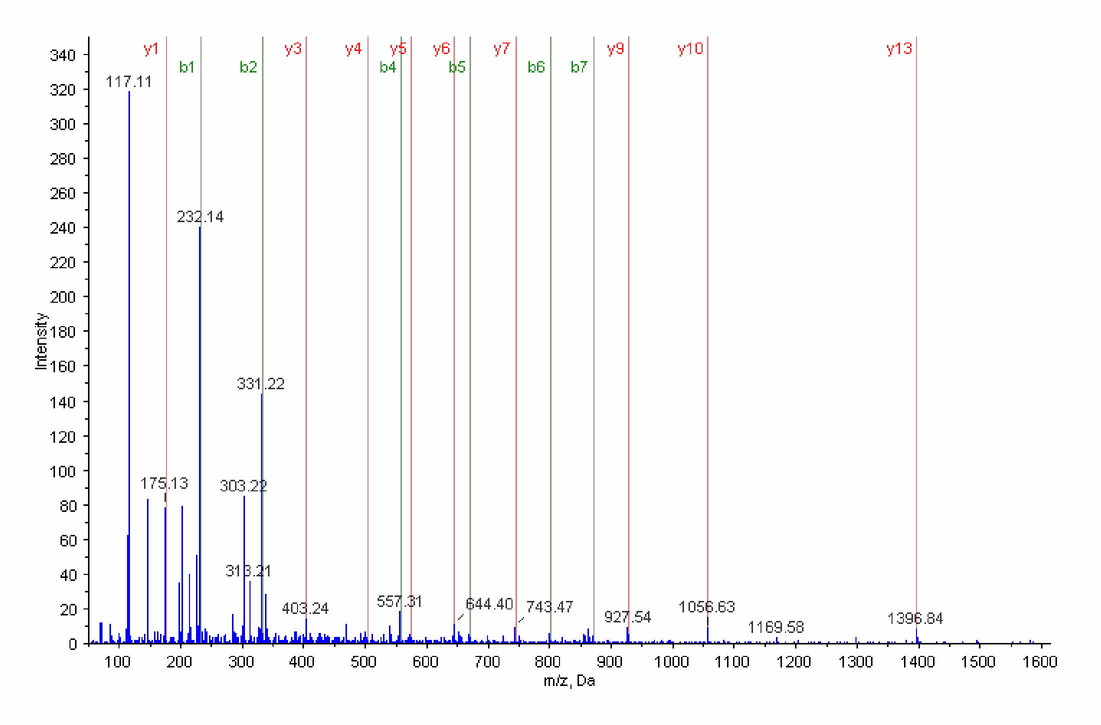

Protein: SCO5086, ActII [18 of 18]

Protein Quant | Protein ID | Spectra | Summary Statistics

Spectrum List

Spectrum	Time	Prec MW	Prec m/z	Prec z	Best Sequence	Modifications	Conf	Theor MW	z	115:114	%Err 115:114	116:
9.1.1.2132.3	51.385	0.0000	863.6394	0	SVPEIEALVAAVVER	iTRAQ@N-term	99	1724.9794	2	0.9188	8.08	

Peptide ID Hypotheses

Conf	Sc	Prot N	Sequence	Modifications	Theor MW	Theor m/z	z	ΔMass
99	16	411	SVPEIEALVAAVVER	iTRAQ@N-term	1724.9794	863.4970	2	0.2848
< 1	8		VEVREVAEGVYAYEQAPGGWCVSNAGIVVGGDGALVVD...	iTRAQ@N-term, MMTS(C)@...	5175.6355	863.6132	6	0.1570
< 1	7		VAPTDLDDWGRMAQYSDPSGGQFAWQPGR	iTRAQ@N-term	3450.6173	863.6616	4	-0.0889
< 1	6		VGVGCMVDSCRECENCKAGR	iTRAQ@N-term, MMTS(C)@...	2588.0178	863.6799	3	-0.1215

Precursor MS Region

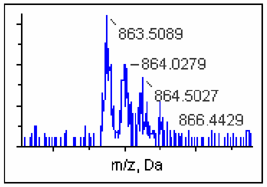

Fragmentation Evidence for Peptide

ITR SVPEIEALVAAVVER

Residue	b	y
S	232.1414	1725.9867
V	331.2098	1494.8526
P	428.2625	1395.7842
E	557.3051	1298.7314
I	670.3892	1169.6888
E	799.4318	1056.6048
A	870.4689	927.5622
L	983.5530	856.5251
V	1082.6214	743.4410
A	1153.6585	644.3726
A	1224.6956	573.3355
V	1323.7640	502.2984
V	1422.8324	403.2300
E	1551.8750	304.1615
R	1707.9761	175.1190

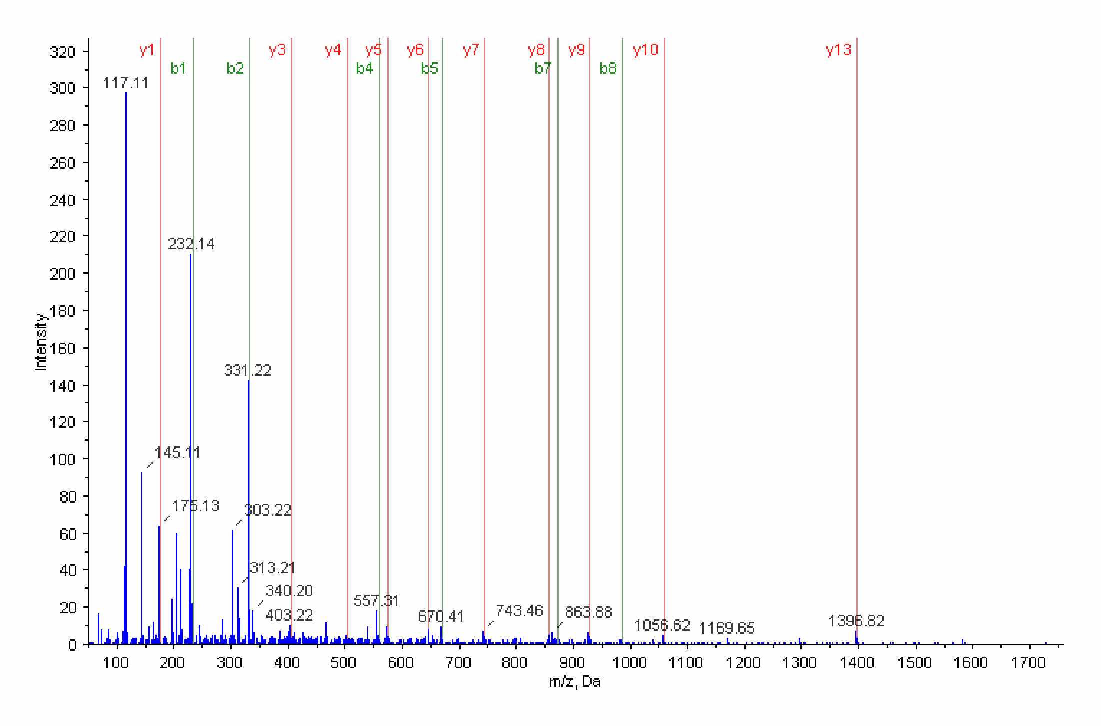

Protein: SCO5089, ActI ORF3 [1 of 1]

Protein Quant | Protein ID | Spectra | Summary Statistics

Spectrum List

Spectrum	Time	Prec MW	Prec m/z	Prec z	Best Sequence	Modifications	Conf	Theor MW	z	115:114	%Err 115:114	116:
21.1.1.960.2	33.084	1391.7156	696.8650	2	YGVSIPDDVAGR	iTRAQ@N-term	99	1391.7167	2	1.4267	22.67	

Peptide ID Hypotheses

Conf	Sc	Prot N	Sequence	Modifications	Theor MW	Theor m/z	z	ΔMass
99	17	642	YGVSIPDDVAGR	iTRAQ@N-term	1391.7167	696.8656	2	-0.0012
< 1	9		VLNGRSDVSSTTR	No iTRAQ@N-term, Deamid...	1391.7005	696.8576	2	0.0151
< 1	8		YGPAATTEADPR	iTRAQ@N-term	1391.6803	696.8474	2	0.0352
< 1	8		YRAEISEGRAP	iTRAQ@N-term	1391.7280	696.8712	2	-0.0124
< 1	7		SLGWNQVDGK	iTRAQ@N-term, Deamidatio...	1391.7289	696.8717	2	-0.0133

Precursor MS Region

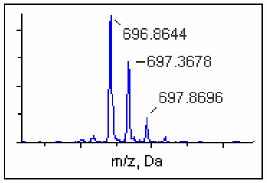

Fragmentation Evidence for Peptide

ITR YGVSIPDDVAGR

Res	b	y
Y	308.1727	1392.7240
G	365.1941	1085.5586
V	464.2625	1028.5371
S	551.2946	929.4687
I	664.3786	842.4367
P	761.4314	729.3526
D	876.4583	632.2998
D	991.4853	517.2729
V	1090.5537	402.2459
A	1161.5908	303.1775
G	1218.6123	232.1404
R	1374.7134	175.1190

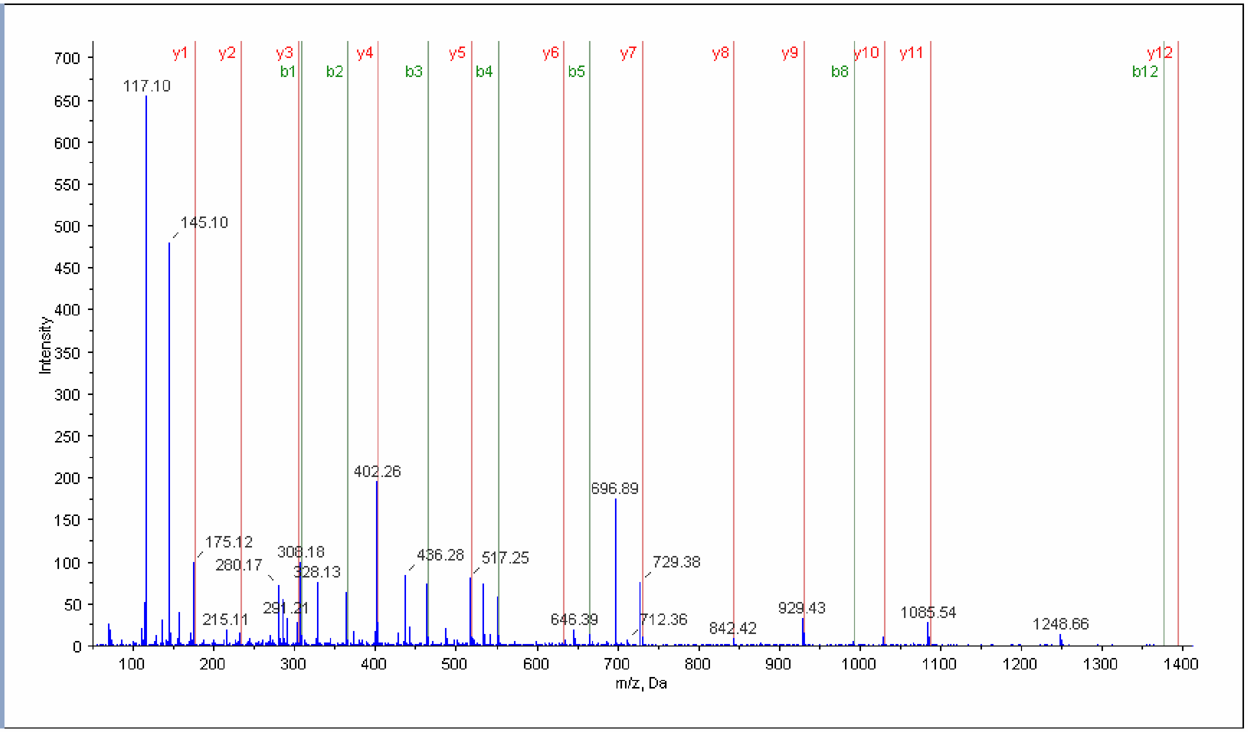

Protein: SCO5139, DapE [1 of 1]

Protein Quant | Protein ID | Spectra | Summary Statistics

Spectrum List

Spectrum	Time	Prec MW	Prec m/z	Prec z	Best Sequence	Modifications	Conf	Theor MW	z	115:114	%Err 115:114	116:
42.1.1.952.5	30.064	1853.9569	464.4965	4	YAPDRSPEEALAHVR	iTRAQ@N-term	99	1853.9506	4	1.0617	11.55	

Peptide ID Hypotheses

Conf	Sc	Prot N	Sequence	Modifications	Theor MW	Theor m/z	z	ΔMass
99	14	803	YAPDRSPEEALAHVR	iTRAQ@N-term	1853.9506	464.4949	4	0.0063
< 1	6	751	LAHALEQTVGADVDVR	iTRAQ@N-term, Oxidation(H...	1853.9605	464.4974	4	-0.0036
< 1	9		ASVAVYADPDRDALHVR	No iTRAQ@N-term	1853.9384	464.4919	4	0.0185
< 1	7		AHLDIGNLPTTTNTDK	No iTRAQ@N-term, iTRAQ(...	1853.9605	464.4974	4	-0.0036
< 1	6		ELATTGLFIAIGHDPR	iTRAQ@N-term	1854.0122	464.5103	4	-0.0553

Precursor MS Region

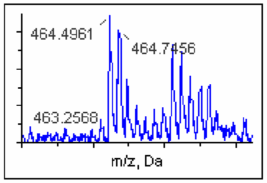

Fragmentation Evidence for Peptide

ITR YAPDRSPEEALAHVR

Res	b	y
Y	308.1727	1854.9579
A	379.2098	1547.7925
P	476.2625	1476.7554
D	591.2895	1379.7026
R	747.3906	1264.6757
S	834.4226	1108.5745
P	931.4754	1021.5425
E	1060.5180	924.4898
E	1189.5606	795.4472
A	1260.5977	666.4046
L	1373.6818	595.3675
A	1444.7189	482.2834
H	1581.7778	411.2463
V	1680.8462	274.1874
R	1836.9473	175.1190

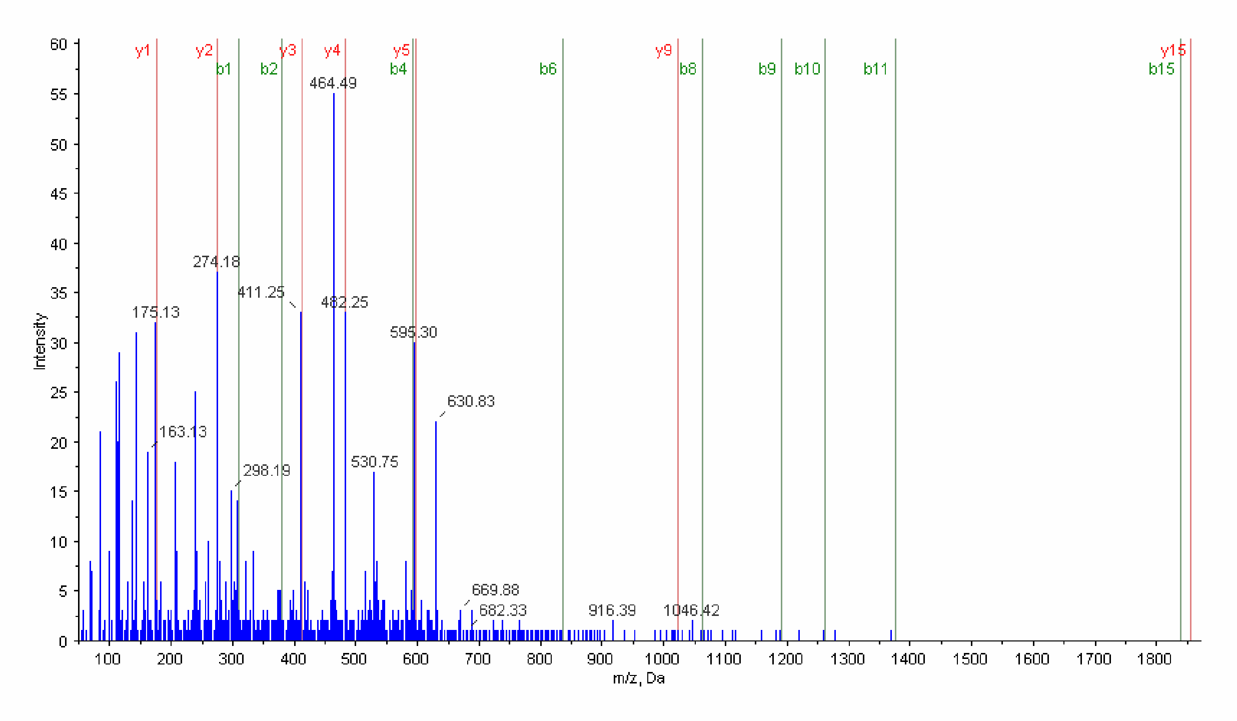

Protein: SCO5354, ThrA [1 of 1]

Protein Quant | Protein ID | Spectra | Summary Statistics

Spectrum List

Spectrum	Time	Prec MW	Prec m/z	Prec z	Best Sequence	Modifications	Conf	Theor MW	z	115:114	%Err 115:114	116:
41.1.1.994.2	34.999	1895.0726	474.7754	4	VHPAMIPLSHPLANVR	iTRAQ@N-term	99	1895.0685	4	1.1405	9.41	

Peptide ID Hypotheses

Conf	Sc	Prot N	Sequence	Modifications	Theor MW	Theor m/z	z	ΔMass
99	17	892	VHPAMIPLSHPLANVR	iTRAQ@N-term	1895.0685	474.7744	4	0.0041
< 1	7		EAGGNGLPSYPHPRR	iTRAQ@N-term, iTRAQ(Y)@...	1895.0006	474.7574	4	0.0721
< 1	6		HEVTVRVRGTAEEIR	iTRAQ@N-term	1895.0459	474.7688	4	0.0267
< 1	6		HLAQVLGLSLFRELR	iTRAQ@N-term	1895.1227	474.7879	4	-0.0501

Precursor MS Region

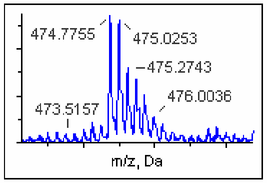

Fragmentation Evidence for Peptide

ITR VHPAMIPLSHPLANVR

Res	b	y
V	244.1778	1896.0758
H	381.2367	1652.9053
P	478.2894	1515.8464
A	549.3265	1418.7937
M	680.3670	1347.7566
I	793.4511	1216.7161
P	890.5039	1103.6320
L	1003.5879	1006.5792
S	1090.6199	893.4952
H	1227.6789	806.4631
P	1324.7316	669.4042
L	1437.8157	572.3515
A	1508.8528	459.2674
N	1622.8957	388.2303
V	1721.9641	274.1874
R	1878.0653	175.1190

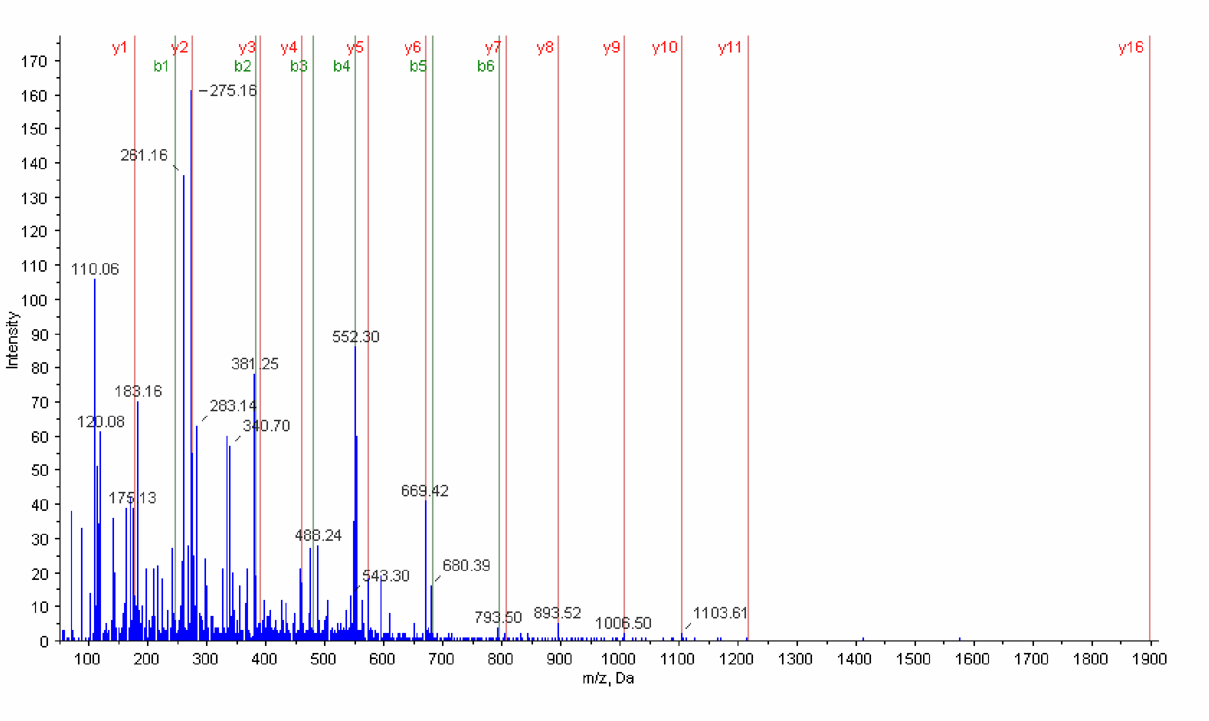

Protein: SCO5356, ThrB [1 of 1]

Protein Quant | Protein ID | Spectra | Summary Statistics

Spectrum List

Spectrum	Time	Prec MW	Prec m/z	Prec z	Best Sequence	Modifications	Conf	Theor MW	z	115:114	%Err 115:114	116:
33.1.1.921.5	25.121	1408.7286	470.5835	3	SVPHVDAAANAGR	iTRAQ@N-term, Deamidatio...	99	1408.7180	3	0.9327	14.23	

Peptide ID Hypotheses

Conf	Sc	Prot N	Sequence	Modifications	Theor MW	Theor m/z	z	ΔMass
99	15	723	SVPHVDAAANAGR	iTRAQ@N-term, Deamidatio...	1408.7180	470.5799	3	0.0106
< 1	7		SSAAFGRDEVSAGR	No iTRAQ@N-term	1408.6695	470.5638	3	0.0592
< 1	7		TGAAGETLSFRR	iTRAQ@N-term	1408.7544	470.5921	3	-0.0258
< 1	6		AQGDGGLDHSALLR	No iTRAQ@N-term	1408.7059	470.5759	3	0.0228
< 1	6		TGHVQIADNPGR	iTRAQ@N-term, Deamidatio...	1408.7180	470.5799	3	0.0106

Precursor MS Region

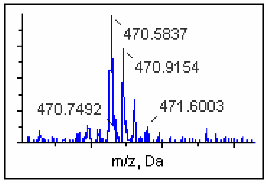

Fragmentation Evidence for Peptide

ITR SVPHVDAAAN[Dea]AGR

Res	b	y
S	232.1414	1409.7254
V	331.2098	1178.5913
P	428.2625	1079.5228
H	565.3215	982.4701
V	664.3899	845.4112
D	779.4168	746.3428
A	850.4539	631.3158
A	921.4910	560.2787
A	992.5282	489.2416
N[	1107.5551	418.2045
A	1178.5922	303.1775
G	1235.6137	232.1404
R	1391.7148	175.1190

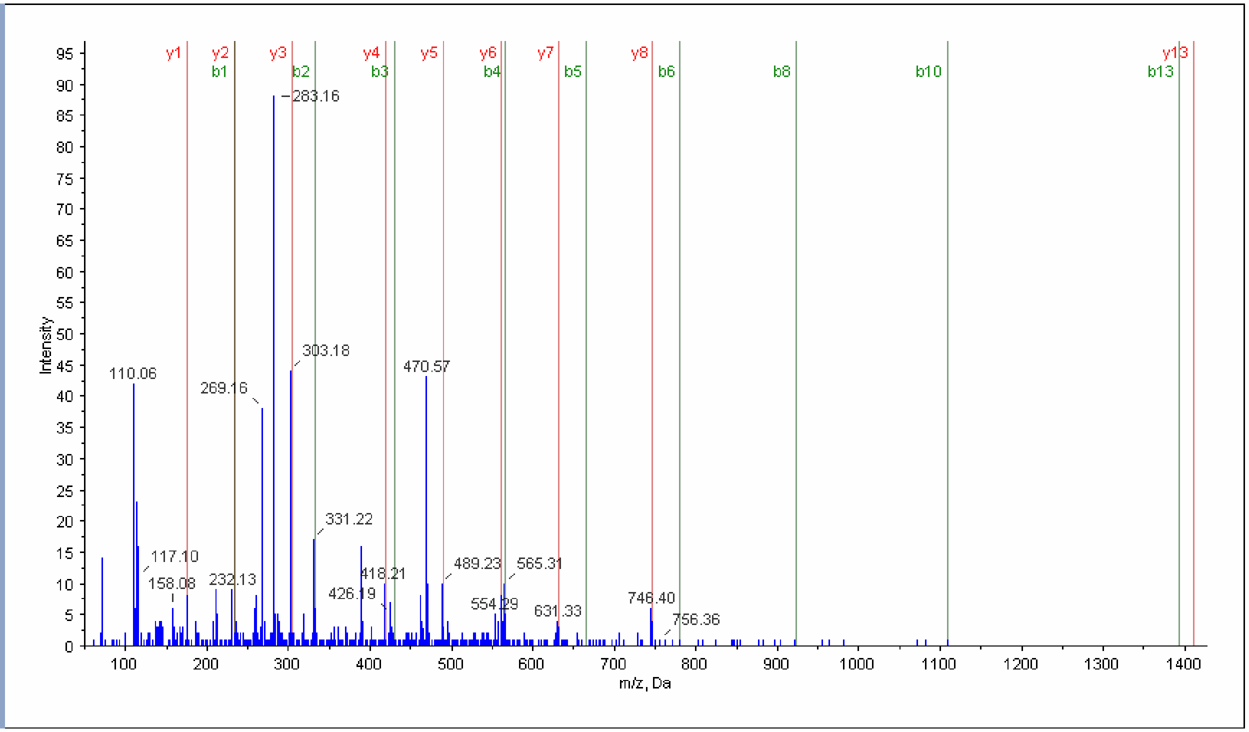

Protein: SCO5366, AtpI [1 of 1]

Protein Quant | Protein ID | Spectra | Summary Statistics

Spectrum List

Spectrum	Time	Prec MW	Prec m/z	Prec z	Best Sequence	Modifications	Conf	Theor MW	z	115:114	%Err 115:114	116:
36.1.1.935.3	28.233	2146.1736	716.3985	3	VLYVEPEPTGEKPEK	iTRAQ@N-term, iTRAQ(K)@...	99	2146.1887	3	0.9372	7.92	

Peptide ID Hypotheses

Conf	Sc	Prot N	Sequence	Modifications	Theor MW	Theor m/z	z	ΔMass
99	14	987	VLYVEPEPTGEKPEK	iTRAQ@N-term, iTRAQ(K)@...	2146.1887	716.4035	3	-0.0151
< 1	7		DLGAGYQRILDGTRAATAAAR	No iTRAQ@N-term	2146.1243	716.3820	3	0.0492
< 1	7		TDRSIELINAAAQAAADK	iTRAQ@N-term, Deamidatio...	2146.1474	716.3898	3	0.0262
< 1	6		EHQTPALLARSAAAAPAGDR	iTRAQ@N-term	2146.1365	716.3861	3	0.0371
< 1	6		VASVGLPVTAYNNPIDTK	iTRAQ@N-term, iTRAQ(K)@...	2146.1878	716.4032	3	-0.0142

Precursor MS Region

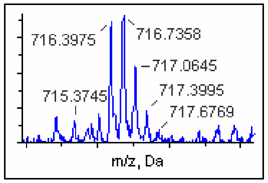

Fragmentation Evidence for Peptide

ITR | VLYVEPEPTGEK[ITR]PEK[ITR]

Res	b	y
V	244.1778	2147.1960
L	357.2618	1904.0255
Y	520.3251	1790.9414
V	619.3936	1627.8781
E	748.4362	1528.8097
P	845.4889	1399.7671
E	974.5315	1302.7143
P	1071.5843	1173.6717
T	1172.6320	1076.6190
G	1229.6534	975.5713
E	1358.6960	918.5498
K[I	1630.8930	789.5072
P	1727.9458	517.3102
E	1856.9884	420.2575
K[I	2129.1854	291.2149

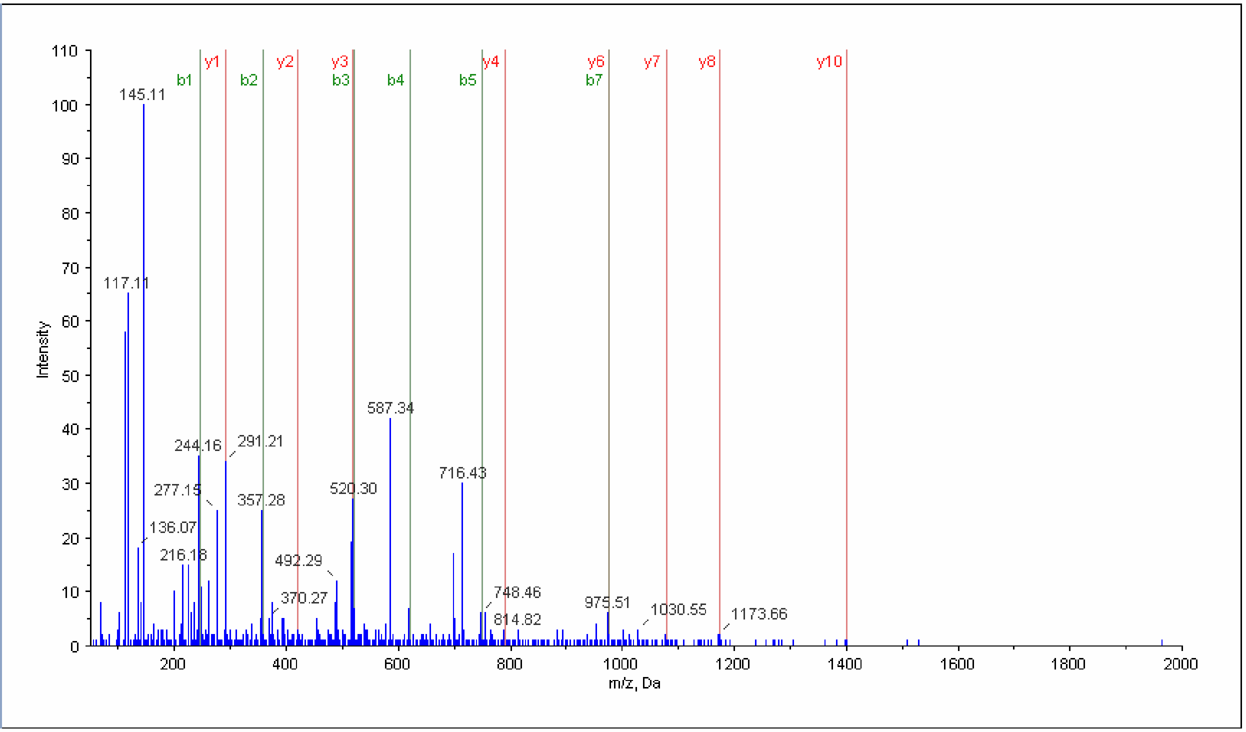

Protein: SCO5368, AtpE [1 of 2]

Protein Quant | Protein ID | Spectra | Summary Statistics

Spectrum List

Spectrum	Time	Prec MW	Prec m/z	Prec z	Best Sequence	Modifications	Conf	Theor MW	z	115:114	%Err 115:114	116:
4.1.1.1305.2	33.706	936.5016	469.2581	2	QPEAAGLIR	PGA of Q@N-term	99	936.5029	2			

Peptide ID Hypotheses

Conf	Sc	Prot N	Sequence	Modifications	Theor MW	Theor m/z	z	ΔMass
99	12	692	QPEAAGLIR	PGA of Q@N-term	936.5029	469.2587	2	-0.0013
< 1	8		DQLTAAYR	No iTRAQ@N-term	936.4665	469.2405	2	0.0351
< 1	7		ELAAAYR	iTRAQ@N-term	936.5151	469.2648	2	-0.0134
< 1	6		DRLAYR	iTRAQ@N-term	936.5263	469.2704	2	-0.0247
< 1	6		EEPAEQPR	PGA of E@N-term	936.4301	469.2223	2	0.0715

Precursor MS Region

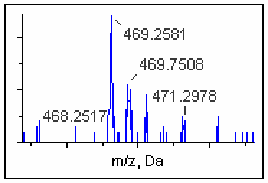

Fragmentation Evidence for Peptide

PGQ QPEAAGLIR

Res	b	y
Q	112.0393	937.5102
P	**209.0921**	826.4781
E	**338.1347**	**729.4254**
A	**409.1718**	**600.3828**
A	**480.2089**	**529.3457**
G	**537.2304**	**458.3085**
L	650.3144	**401.2871**
I	763.3985	**288.2030**
R	919.4996	**175.1190**

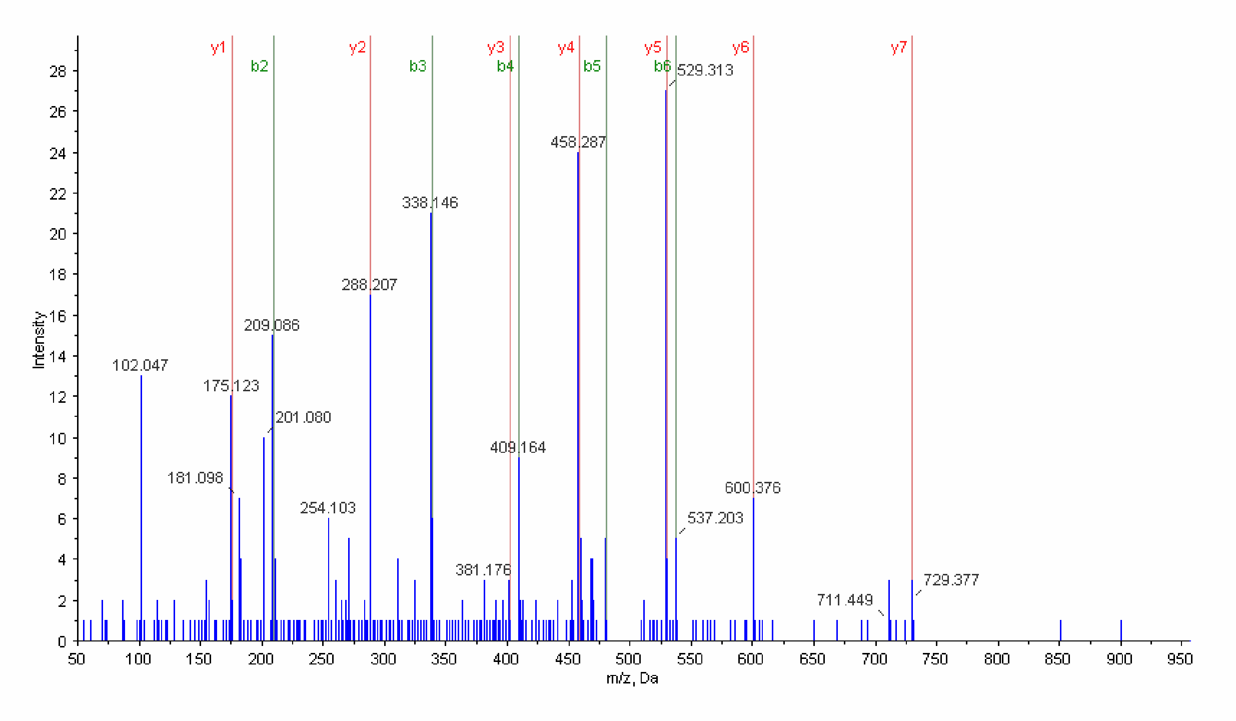

Protein: SCO5368, AtpE [2 of 2]

Protein Quant | Protein ID | Spectra | Summary Statistics

Spectrum List

Spectrum	Time	Prec MW	Prec m/z	Prec z	Best Sequence	Modifications	Conf	Theor MW	z	115:114	%Err 115:114	116:
5.1.1.2131.2	40.063	1097.6117	549.8131	2	QPEAAGLIR	iTRAQ@N-term	99	1097.6315	2	0.9454	1.87	

Peptide ID Hypotheses

Conf	Sc	Prot N	Sequence	Modifications	Theor MW	Theor m/z	z	ΔMass
99	14	594	QPEAAGLIR	iTRAQ@N-term	1097.6315	549.8230	2	-0.0198
< 1	8		EDAQIKIR	PGA of E@N-term, iTRAQ(K...	1097.6315	549.8230	2	-0.0198
< 1	8		RAGLARDAIR	No iTRAQ@N-term	1097.6418	549.8281	2	-0.0301
< 1	8		VQVVAEGPR	iTRAQ@N-term	1097.6315	549.8230	2	-0.0198
< 1	7		DELVPLLR	iTRAQ@N-term	1097.6566	549.8356	2	-0.0449

Precursor MS Region

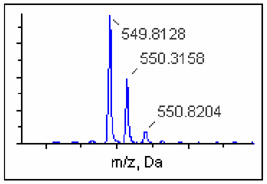

Fragmentation Evidence for Peptide

ITR QPEAAGLIR

Residue	b	y
Q	273.1679	1098.6388
P	370.2207	826.4781
E	499.2633	729.4254
A	570.3004	600.3828
A	641.3375	529.3457
G	698.3590	458.3085
L	811.4430	401.2871
I	924.5271	288.2030
R	1080.6282	175.1190

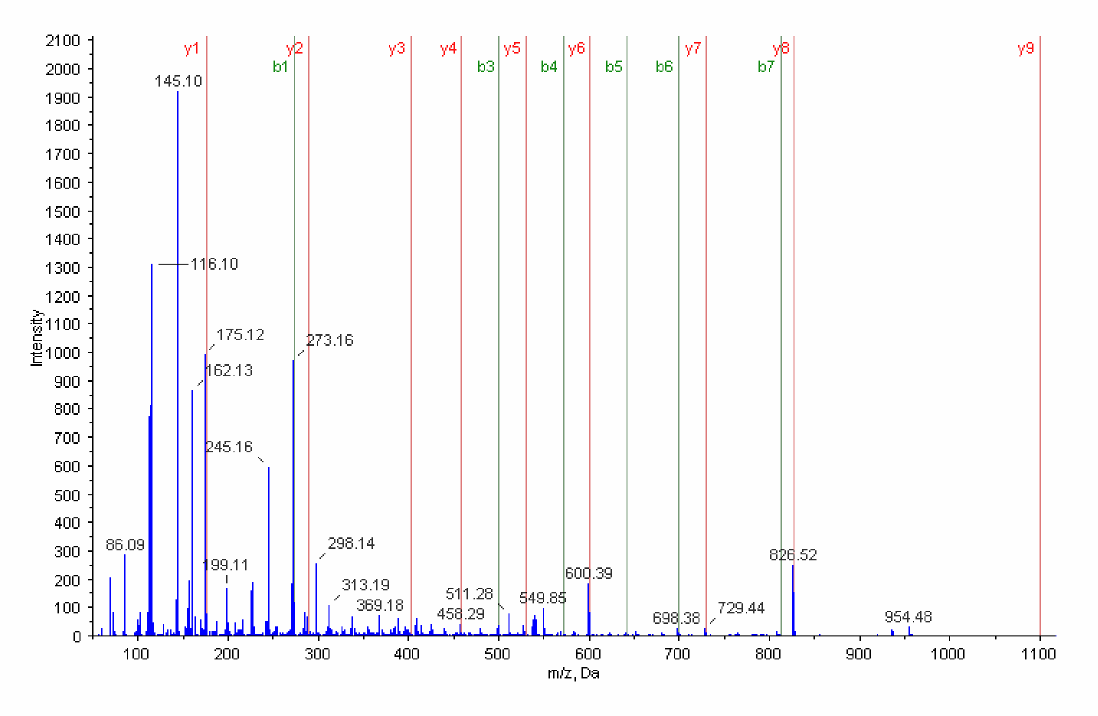

Protein: SCO5739, DapB [1 of 2]

Protein Quant | Protein ID | Spectra | Summary Statistics

Spectrum List

Spectrum	Time	Prec MW	Prec m/z	Prec z	Best Sequence	Modifications	Conf	Theor MW	z	115:114	%Err 115:114	116:
20.1.1.963.4	30.091	1755.8937	878.9541	2	AGSAPAPDATATALDGAR	iTRAQ@N-term	99	1755.8874	2	0.5772	13.96	

Peptide ID Hypotheses

Conf	Sc	Prot N	Sequence	Modifications	Theor MW	Theor m/z	z	ΔMass
99	14	853	AGSAPAPDATATALDGAR	iTRAQ@N-term	1755.8874	878.9509	2	0.0063
<1	6		AEEPTATTSRWPSAPR	No iTRAQ@N-term	1755.8540	878.9343	2	0.0396
<1	5		ALDDVTYTLCVITGAR	No iTRAQ@N-term, MMTS(...	1755.8536	878.9340	2	0.0401
<1	5		QATGERAAAVSATANAAGR	PGA of Q@N-term, Deamida...	1755.8500	878.9323	2	0.0437
<1	5		TLNDDRSWAHNGAR	iTRAQ@N-term	1755.8523	878.9334	2	0.0414

Precursor MS Region

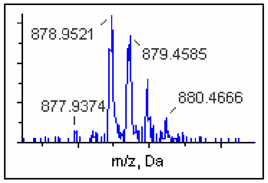

Fragmentation Evidence for Peptide

ITR AGSAPAPDATATALDGAR

Res	b	y
A	**216.1465**	**1756.8946**
G	**273.1679**	1541.7554
S	**360.1999**	1484.7340
A	**431.2371**	1397.7019
P	**528.2898**	**1326.6648**
A	**599.3269**	1229.6121
P	696.3797	**1158.5749**
D	811.4066	**1061.5222**
A	882.4438	**946.4952**
T	983.4914	875.4581
A	1054.5285	774.4104
T	1155.5762	**703.3733**
A	1226.6133	602.3257
L	1339.6974	**531.2885**
D	1454.7243	418.2045
G	1511.7458	**303.1775**
A	1582.7829	246.1561
R	1738.8840	**175.1190**

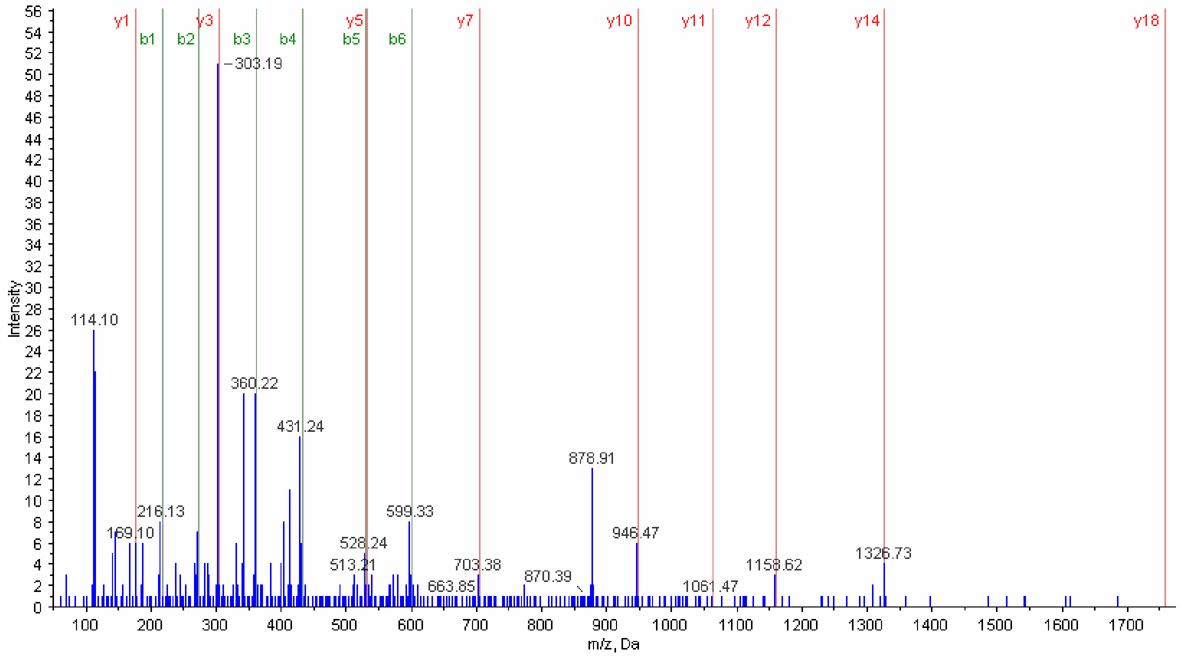

Protein: SCO5739, DapB [2 of 2]

Protein Quant | Protein ID | Spectra | Summary Statistics

Spectrum List

Spectrum	Time	Prec MW	Prec m/z	Prec z	Best Sequence	Modifications	Conf	Theor MW	z	115:114	%Err 115:114	116:
20.1.1.963.5	30.142	1755.9067	586.3095	3	AGSAPAPDATATALDGAR	iTRAQ@N-term	99	1755.8875	3			

Peptide ID Hypotheses

Conf	Sc	Prot N	Sequence	Modifications	Theor MW	Theor m/z	z	ΔMass
99	15	853	AGSAPAPDATATALDGAR	iTRAQ@N-term	1755.8875	586.3031	3	0.0193
< 1	5	80	ALAGAITSLMTGTSYR	iTRAQ@N-term	1755.9312	586.3177	3	-0.0245
< 1	7		ASDANVALGGGKNHR	iTRAQ@N-term, Deamidatio...	1755.9109	586.3109	3	-0.0041
< 1	7		ATADAVRVARAVEGAR	iTRAQ@N-term	1755.9827	586.3348	3	-0.0760
< 1	7		ESGSPDLALVVNTGPR	iTRAQ@N-term, Deamidatio...	1755.9126	586.3115	3	-0.0059

Precursor MS Region

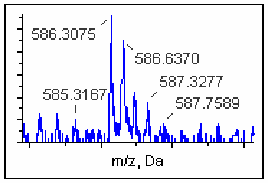

Fragmentation Evidence for Peptide

ITR AGSAPAPDATATALDGAR

Res	b	y
A	216.1465	1756.8946
G	273.1679	1541.7554
S	360.1999	1484.7340
A	431.2371	1397.7019
P	528.2898	1326.6648
A	599.3269	1229.6121
P	696.3797	1158.5749
D	811.4066	1061.5222
A	882.4438	946.4952
T	983.4914	875.4581
A	1054.5285	774.4104
T	1155.5762	703.3733
A	1226.6133	602.3257
L	1339.6974	531.2885
D	1454.7243	418.2045
G	1511.7458	303.1775
A	1582.7829	246.1561
R	1738.8840	175.1190

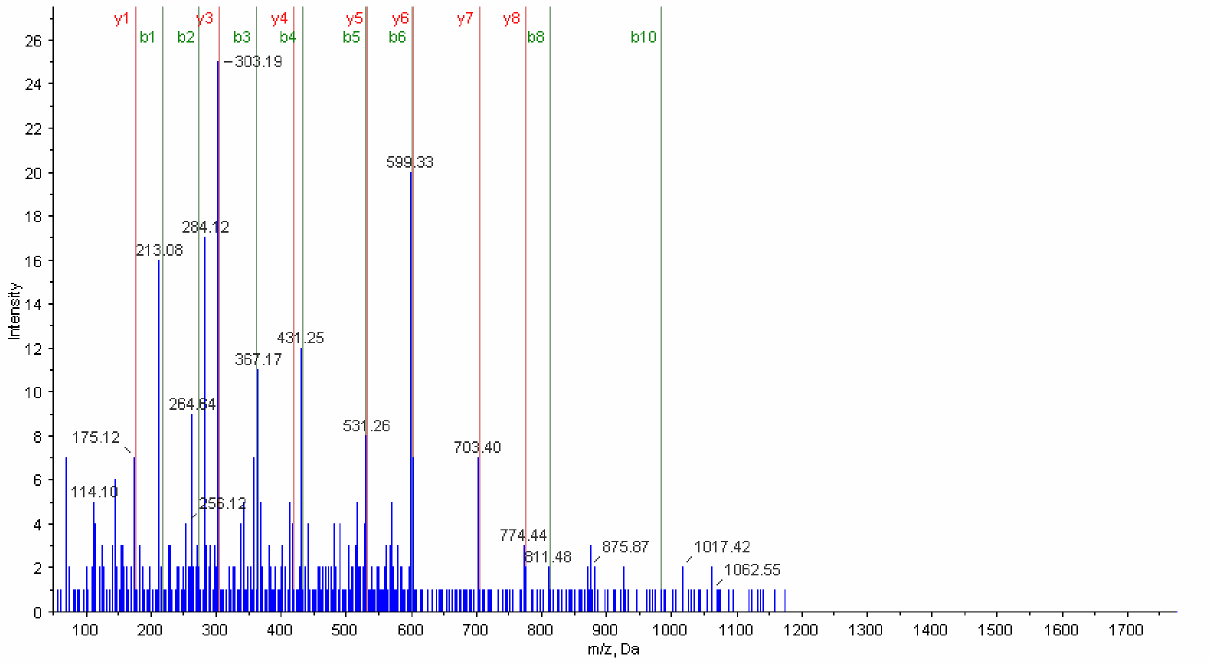

Protein: SCO5774, GluD [1 of 2]

Protein Quant | Protein ID | Spectra | Summary Statistics

Spectrum List

Spectrum	Time	Prec MW	Prec m/z	Prec z	Best Sequence	Modifications	Conf	Theor MW	z	115:114	%Err 115:114	116:
9.1.1.939.2	26.651	1628.7589	815.3867	2	ADDAGIPGGAATGAGGGGN	iTRAQ@N-term	99	1628.7512	2	4.5325	21.14	

Peptide ID Hypotheses

Conf	Sc	Prot N	Sequence	Modifications	Theor MW	Theor m/z	z	ΔMass
99	17	927	ADDAGIPGGAATGAGGGGN	iTRAQ@N-term	1628.7512	815.3829	2	0.0077
< 1	7		MPGADALERVQSAER	No iTRAQ@N-term	1628.7940	815.4043	2	-0.0351
< 1	6		LSAGEEVDVETEVPR	No iTRAQ@N-term	1628.7893	815.4019	2	-0.0304
< 1	6		MSGNTITHDVAAAGER	No iTRAQ@N-term	1628.7576	815.3861	2	0.0013
< 1	6		QAVGWEEEGGGDRTR	PGA of Q@N-term	1628.7179	815.3662	2	0.0410

Precursor MS Region

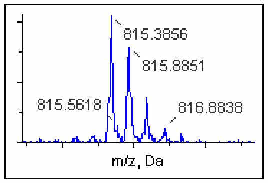

Fragmentation Evidence for Peptide

ITR ADDAGIPGGAATGAGGGGN

Res	b	y
A	216.1465	1629.7585
D	331.1734	1414.6193
D	446.2003	1299.5924
A	517.2375	1184.5654
G	574.2589	1113.5283
I	687.3430	1056.5069
P	784.3957	943.4228
G	841.4172	846.3700
G	898.4387	789.3486
A	969.4758	732.3271
A	1040.5129	661.2900
T	1141.5606	590.2529
G	1198.5820	489.2052
A	1269.6192	432.1837
G	1326.6406	361.1466
G	1383.6621	304.1252
G	1440.6835	247.1037
G	1497.7050	190.0822
N	1611.7479	133.0608

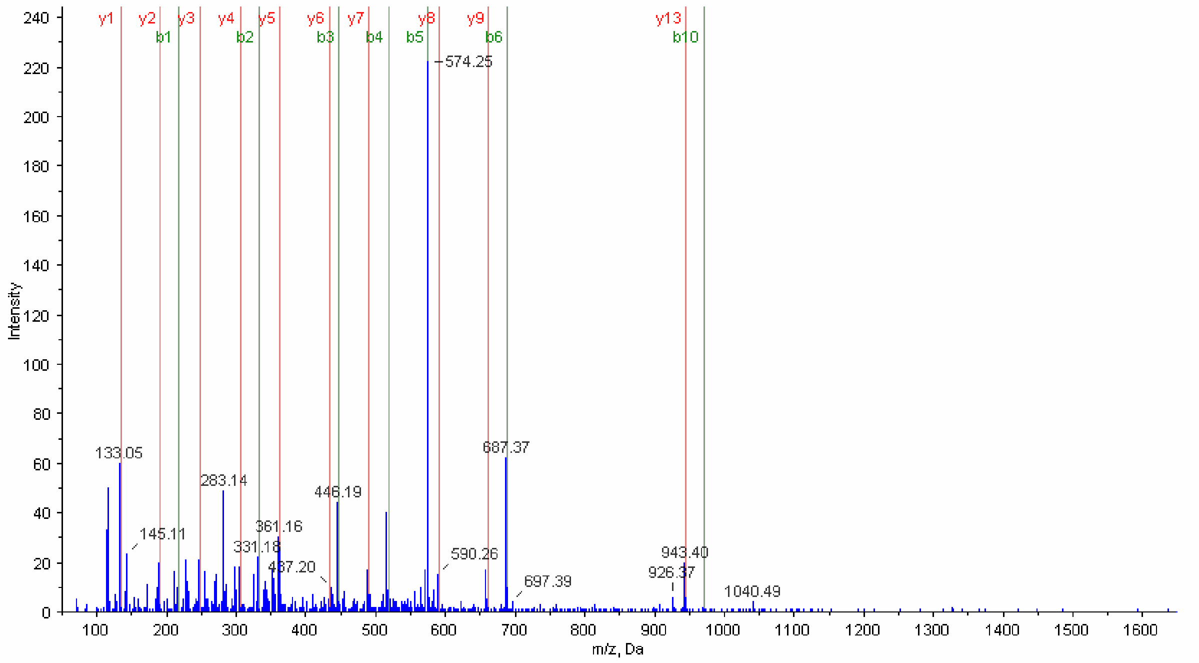

Protein: SCO5774, GluD [2 of 2]

Protein Quant | Protein ID | Spectra | Summary Statistics

Spectrum List

Spectrum	Time	Prec MW	Prec m/z	Prec z	Best Sequence	Modifications	Conf	Theor MW	z	115:114	%Err 115:114	116:
10.1.1.1039.2	27.010	1628.7336	815.3741	2	ADDAGIPGGAATGAGGGGN	iTRAQ@N-term	99	1628.7513	2	7.5631	40.41	

Peptide ID Hypotheses

Conf	Sc	Prot N	Sequence	Modifications	Theor MW	Theor m/z	z	ΔMass
99	16	927	ADDAGIPGGAATGAGGGGN	iTRAQ@N-term	1628.7513	815.3829	2	-0.0176
< 1	8		MPGADALERVQSAER	No iTRAQ@N-term	1628.7941	815.4043	2	-0.0604
< 1	7		RGEIFGLLGPNGAGK	No iTRAQ@N-term, iTRAQ(...	1628.9121	815.4633	2	-0.1784
< 1	6		GSRVVILGLNGAGK	iTRAQ@N-term, Deamidatio...	1628.9818	815.4981	2	-0.2481
< 1	5		AAFGGDLTVVTYK	iTRAQ@N-term, iTRAQ(K)@...	1628.9018	815.4581	2	-0.1681

Precursor MS Region

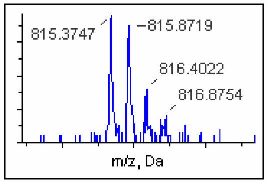

Fragmentation Evidence for Peptide

ITR ADDAGIPGGAATGAGGGGN

Res	b	y
A	216.1465	1629.7585
D	331.1734	1414.6193
D	446.2003	1299.5924
A	517.2375	1184.5654
G	574.2589	1113.5283
I	687.3430	1056.5069
P	784.3957	943.4228
G	841.4172	846.3700
G	898.4387	789.3486
A	969.4758	732.3271
A	1040.5129	661.2900
T	1141.5606	590.2529
G	1198.5820	489.2052
A	1269.6192	432.1837
G	1326.6406	361.1466
G	1383.6621	304.1252
G	1440.6835	247.1037
G	1497.7050	190.0822
N	1611.7479	133.0608

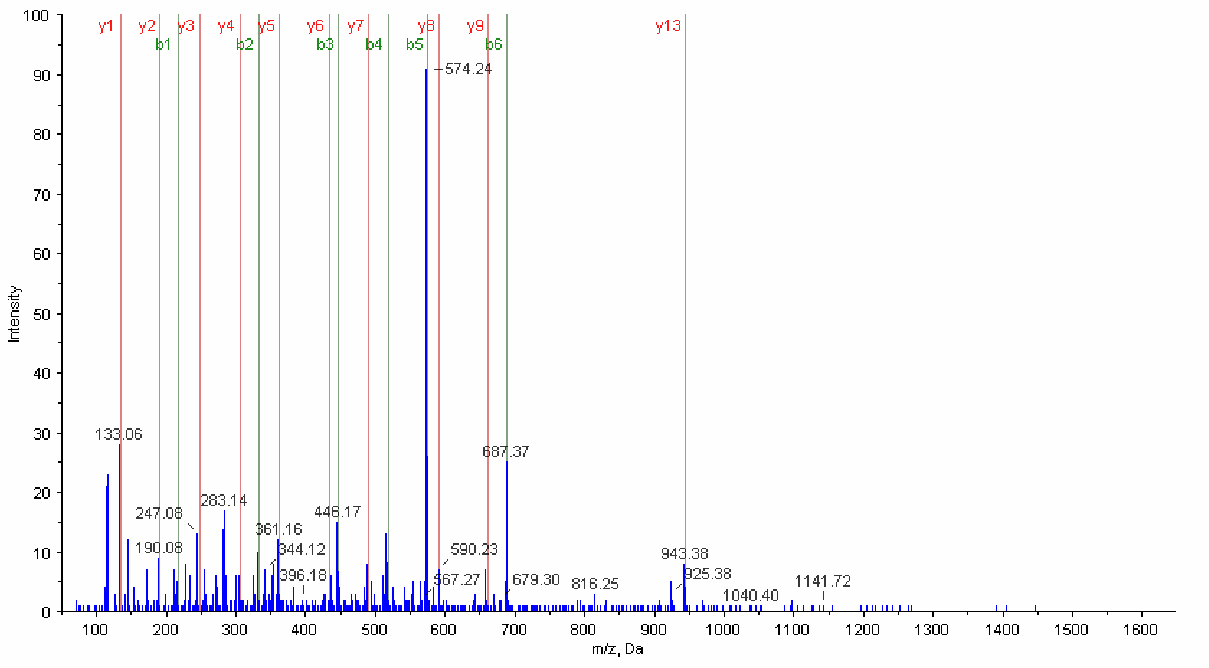

Protein: SCO5880, RedY [1 of 1]

Protein Quant | Protein ID | Spectra | Summary Statistics

Spectrum List

Spectrum	Time	Prec MW	Prec m/z	Prec z	Best Sequence	Modifications	Conf	Theor MW	z	115:114	%Err 115:114	116:
29.1.1.1098.2	48.096	2988.3972	997.1397	3	VSTEQDAPFHYFESISVTGPDEF...	iTRAQ@N-term	99	2988.3898	3			

Peptide ID Hypotheses

Conf	Sc	Prot N	Sequence	Modifications	Theor MW	Theor m/z	z	ΔMass
99	18	773	VSTEQDAPFHYFESISVTGPDEFSR	iTRAQ@N-term	2988.3898	997.1372	3	0.0074
< 1	6		QVYDDARHPYGRALSEAFPTIGDPASR	No iTRAQ@N-term	2988.4477	997.1565	3	-0.0505

Precursor MS Region

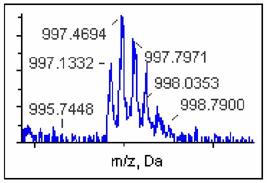

Fragmentation Evidence for Peptide

ITR VSTEQDAPFHYFESISVTGPDEFSR

Res	b	y
V	244.1778	2989.3972
S	331.2098	2746.2267
T	432.2575	2659.1947
E	561.3001	2558.1470
Q	689.3586	2429.1044
D	804.3856	2301.0459
A	875.4227	2186.0189
P	972.4755	2114.9818
F	1119.5439	2017.9290
H	1256.6028	1870.8606
Y	1419.6661	1733.8017
F	1566.7345	1570.7384
E	1695.7771	1423.6700
S	1782.8091	1294.6274
I	1895.8932	1207.5953
S	1982.9252	1094.5113
V	2081.9936	1007.4793
T	2183.0413	908.4108
G	2240.0628	807.3632
P	2337.1156	750.3417
D	2452.1425	653.2889
E	2581.1851	538.2620
F	2728.2535	409.2194
S	2815.2855	262.1510
R	2971.3866	175.1190

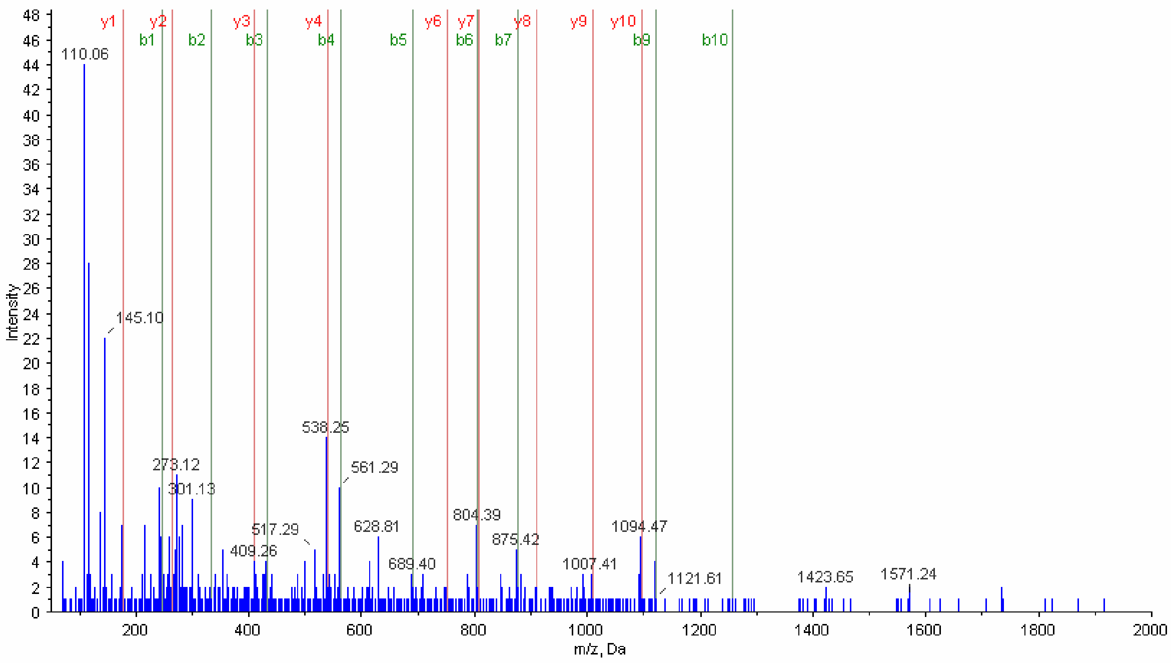

Protein: SCO5881, RedZ [1 of 1]

Protein Quant | Protein ID | Spectra | Summary Statistics

Spectrum List

Spectrum	Time	Prec MW	Prec m/z	Prec z	Best Sequence	Modifications	Conf	Theor MW	z	115:114	%Err 115:114	116:
44.1.1.2524.3	56.058	1995.1188	666.0469	3	AVLSAETSVEELVHVIR	iTRAQ@N-term	99	1995.1121	3	1.5388	6.53	

Peptide ID Hypotheses

Conf	Sc	Prot N	Sequence	Modifications	Theor MW	Theor m/z	z	ΔMass
99	14	539	AVLSAETSVEELVHVIR	iTRAQ@N-term	1995.1121	666.0447	3	0.0066
< 1	7		TVAVGGNAEAARLAGIDVRR	No iTRAQ@N-term	1995.0973	666.0397	3	0.0215
< 1	6		AALDAVFEPDPHFLGPR	iTRAQ@N-term	1995.0335	666.0184	3	0.0853
< 1	6		ALEWLRERGLADAIARR	No iTRAQ@N-term	1995.1125	666.0448	3	0.0063
< 1	5		GGNRWAVTGALSLHGVTR	iTRAQ@N-term	1995.0883	666.0367	3	0.0305

Precursor MS Region

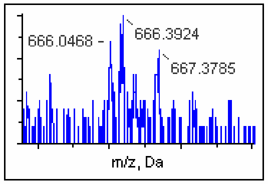

Fragmentation Evidence for Peptide

ITR AVLSAETSVEELVHVIR

Residue	b	y
A	216.1465	1996.1195
V	315.2149	1780.9803
L	428.2989	1681.9119
S	515.3310	1568.8279
A	586.3681	1481.7958
E	715.4107	1410.7587
T	816.4583	1281.7161
S	903.4904	1180.6684
V	1002.5588	1093.6364
E	1131.6014	994.5680
E	1260.6440	865.5254
L	1373.7280	736.4828
V	1472.7965	623.3988
H	1609.8554	524.3303
V	1708.9238	387.2714
I	1822.0078	288.2030
R	1978.1090	175.1190

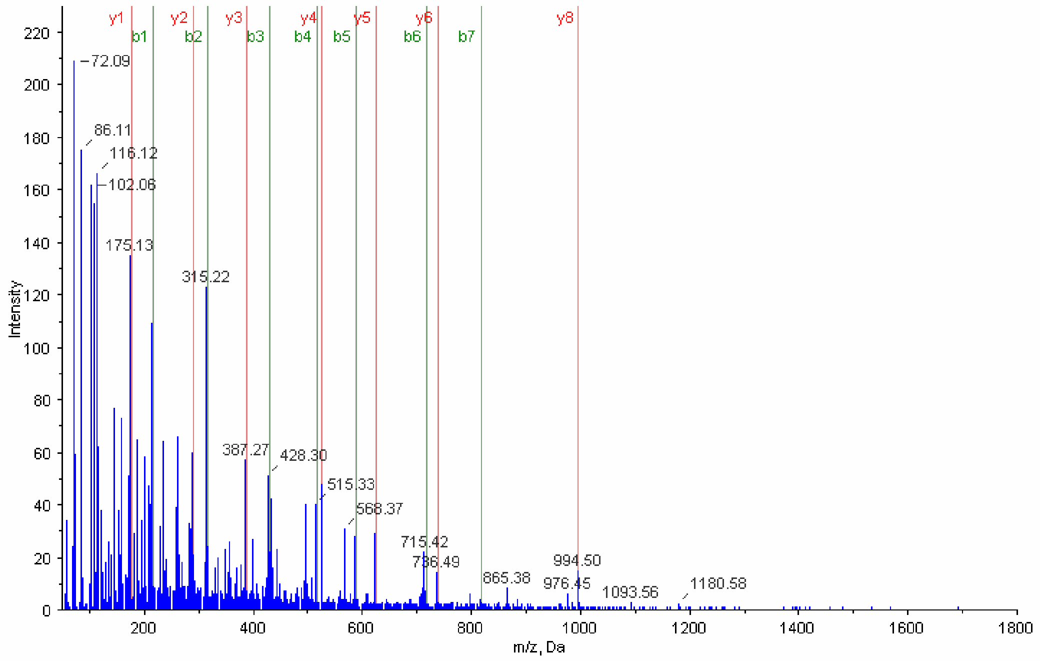

Protein: SCO5882, RedX, RedV [1 of 1]

Protein Quant | Protein ID | Spectra | Summary Statistics

Spectrum List

Spectrum	Time	Prec MW	Prec m/z	Prec z	Best Sequence	Modifications	Conf	Theor MW	z	115:114	%Err 115:114	116:
77.1.1.2229.2	42.282	2054.0269	685.6829	3	DLEEPATPGATAHPVHLR	iTRAQ@N-term	99	2054.0668	3	3.2294	5.62	

Peptide ID Hypotheses

Conf	Sc	Prot N	Sequence	Modifications	Theor MW	Theor m/z	z	ΔMass
99	14	508	DLEEPATPGATAHPVHLR	iTRAQ@N-term	2054.0668	685.6962	3	-0.0400
< 1	7		VGVAGRRLGVHQGHEVDLR	No iTRAQ@N-term	2054.1247	685.7155	3	-0.0979
< 1	6		ADEWFLYDQESPIATGGR	No iTRAQ@N-term	2053.9383	685.6534	3	0.0886
< 1	6		DGGPAVTSRAQARTAAAEAVR	No iTRAQ@N-term	2054.0618	685.6945	3	-0.0350
< 1	6		ECVEAALARVPVEPLGTVR	No iTRAQ@N-term, MMTS(...	2054.0654	685.6958	3	-0.0385

Precursor MS Region

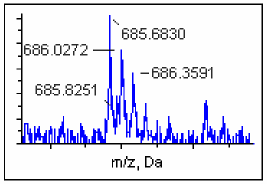

Fragmentation Evidence for Peptide

ITR DLEEPATPGATAHPVHLR

Residue	b	y
D	260.1363	2055.0740
L	373.2203	1795.9450
E	502.2629	1682.8609
E	631.3055	1553.8183
P	728.3583	1424.7757
A	799.3954	1327.7229
T	900.4431	1256.6858
P	997.4959	1155.6382
G	1054.5173	1058.5854
A	1125.5544	1001.5639
T	1226.6021	930.5268
A	1297.6392	829.4791
H	1434.6981	758.4420
P	1531.7509	621.3831
V	1630.8193	524.3303
H	1767.8782	425.2619
L	1880.9623	288.2030
R	2037.0634	175.1190

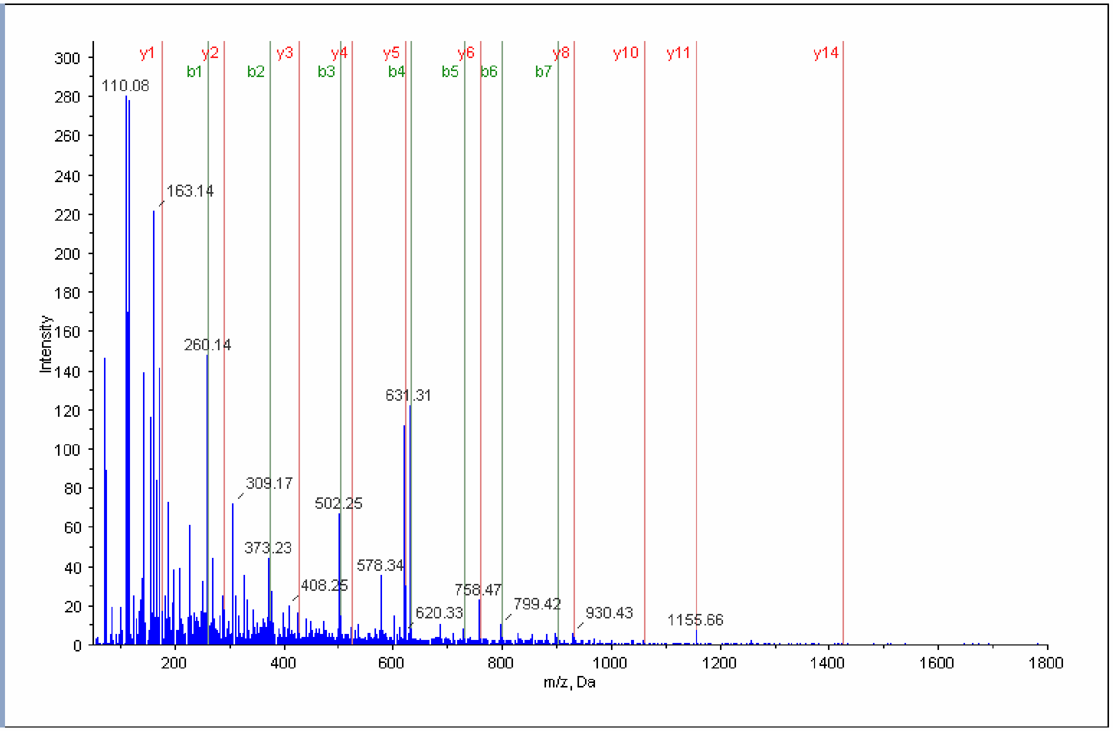

Protein: SCO6222 [1 of 1]

Protein Quant | Protein ID | Spectra | Summary Statistics

Spectrum List

Spectrum	Time	Prec MW	Prec m/z	Prec z	Best Sequence	Modifications	Conf	Theor MW	z	115:114	%Err 115:114	116:
50.1.1.1053.3	44.377	1865.0359	622.6859	3	ILTLPHAEDLEAAIGR	iTRAQ@N-term	99	1862.0382	3	0.6375	13.23	

Peptide ID Hypotheses

Conf	Sc	Prot N	Sequence	Modifications	Theor MW	Theor m/z	z	ΔMass
99	18	914	ILTLPHAEDLEAAIGR	iTRAQ@N-term	1862.0382	621.6867	3	2.9976
< 1	14	13	TLTLEVAQHLGDGLVR	iTRAQ@N-term	1865.0491	622.6903	3	-0.0133
< 1	6	88	IASGELTAVQVTEAHLAR	No iTRAQ@N-term	1865.0006	622.6741	3	0.0353
< 1	6	513	AAHGLADDLLTNTLLTAR	No iTRAQ@N-term	1865.0006	622.6741	3	0.0353
< 1	8		GSVITAKQNDYVQAAR	No iTRAQ@N-term, iTRAQ(...	1864.9764	622.6660	3	0.0595

Precursor MS Region

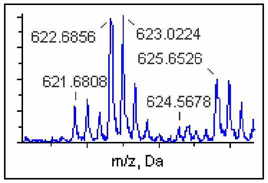

Fragmentation Evidence for Peptide

ITR ILTLPHAEDLEAAIGR

Res	b	y
I	258.1934	1863.0456
L	371.2775	1605.8595
T	472.3251	1492.7754
L	585.4092	1391.7278
P	682.4620	1278.6437
H	819.5209	1181.5909
A	890.5580	1044.5320
E	1019.6006	973.4949
D	1134.6275	844.4523
L	1247.7116	729.4254
E	1376.7542	616.3413
A	1447.7913	487.2987
A	1518.8284	416.2616
I	1631.9125	345.2245
G	1688.9339	232.1404
R	1845.0351	175.1190

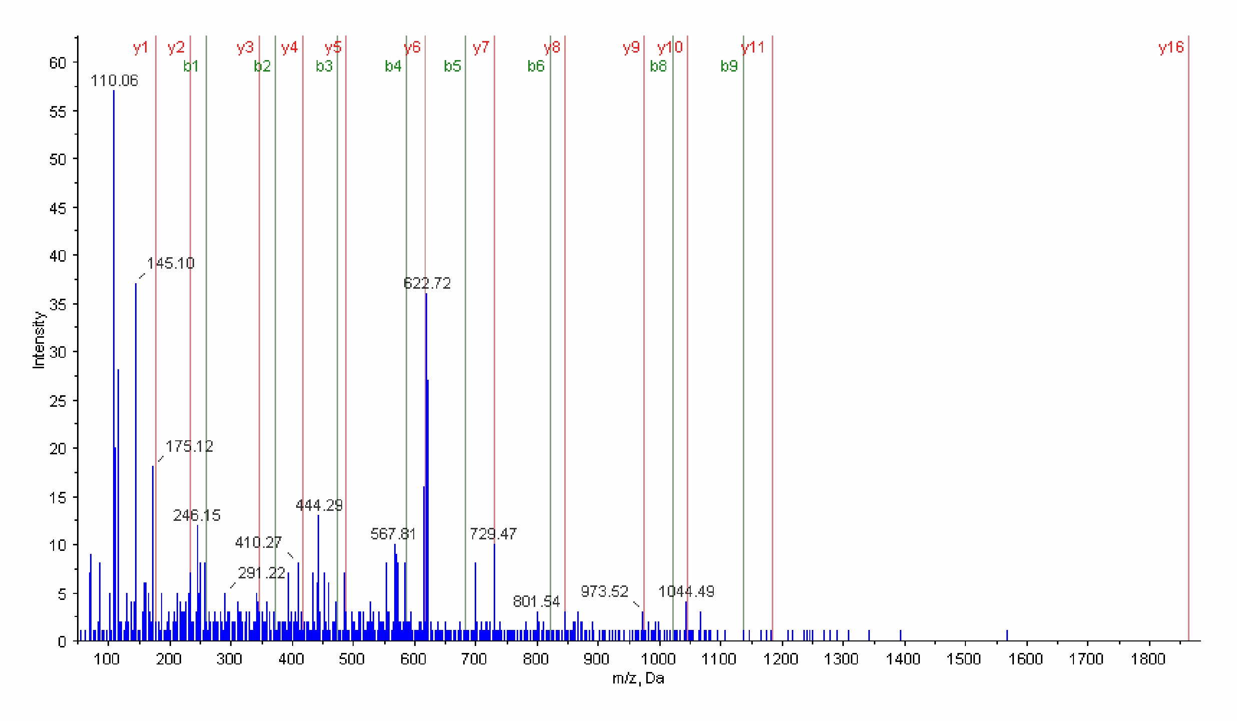

Protein: SCO6286 [1 of 1]

Protein Quant | Protein ID | **Spectra** | Summary Statistics

Spectrum List

Spectrum	Time	Prec MW	Prec m/z	Prec z	Best Sequence	Modifications	Conf	Theor MW	z	115:114	%Err 115:114	116:
16.1.1.1091.5	34.917	2504.2671	835.7630	3	ISADNGTGPVAPEGTSAPGGVVP...	iTRAQ@N-term, Deamidatio...	99	2504.2629	3			

Peptide ID Hypotheses

Conf	Sc	Prot N	Sequence	Modifications	Theor MW	Theor m/z	z	ΔMass
99	16	977	ISADNGTGPVAPEGTSAPGGVVPGPR	iTRAQ@N-term, Deamidatio...	2504.2629	835.7616	3	0.0042
< 1	7		DPATVDMMTVVTVGNTATREIAGR	No iTRAQ@N-term	2504.2363	835.7527	3	0.0308
< 1	5		ERLVSEAEALSNSTDWGPTAAR	iTRAQ@N-term, Deamidatio...	2504.2265	835.7495	3	0.0406
< 1	5		LVGGCCRVGPETITSIARALPR	iTRAQ@N-term, MMTS(C)@...	2504.2970	835.7729	3	-0.0299
< 1	5		TYPNVTIEGRSTPGQCLEPPR	iTRAQ@N-term, MMTS(C)@...	2504.2274	835.7497	3	0.0397

Precursor MS Region

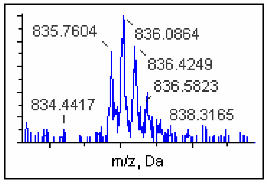

Fragmentation Evidence for Peptide

ITR ISADN[Dea]GTGPVAPEGTSAPGGVVPGPR

Res	b	y
I	258.1934	2505.27
S	345.2254	2248.08
A	416.2625	2161.05
D	531.2895	2090.01
N[	646.3164	1974.98
G	703.3379	1859.96
T	804.3856	1802.93
G	861.4070	1701.89
P	958.4598	1644.87
V	1057.5282	1547.81
A	1128.5653	1448.74
P	1225.6181	1377.71
E	1354.6607	1280.65
G	1411.6821	1151.61
T	1512.7298	1094.59
S	1599.7619	993.54
A	1670.7990	906.51
P	1767.8517	835.47
G	1824.8732	738.42
G	1881.8947	681.40
V	1980.9631	624.38
V	2080.0315	525.31
P	2177.0843	426.24
G	2234.1057	329.19
P	2331.1585	272.17

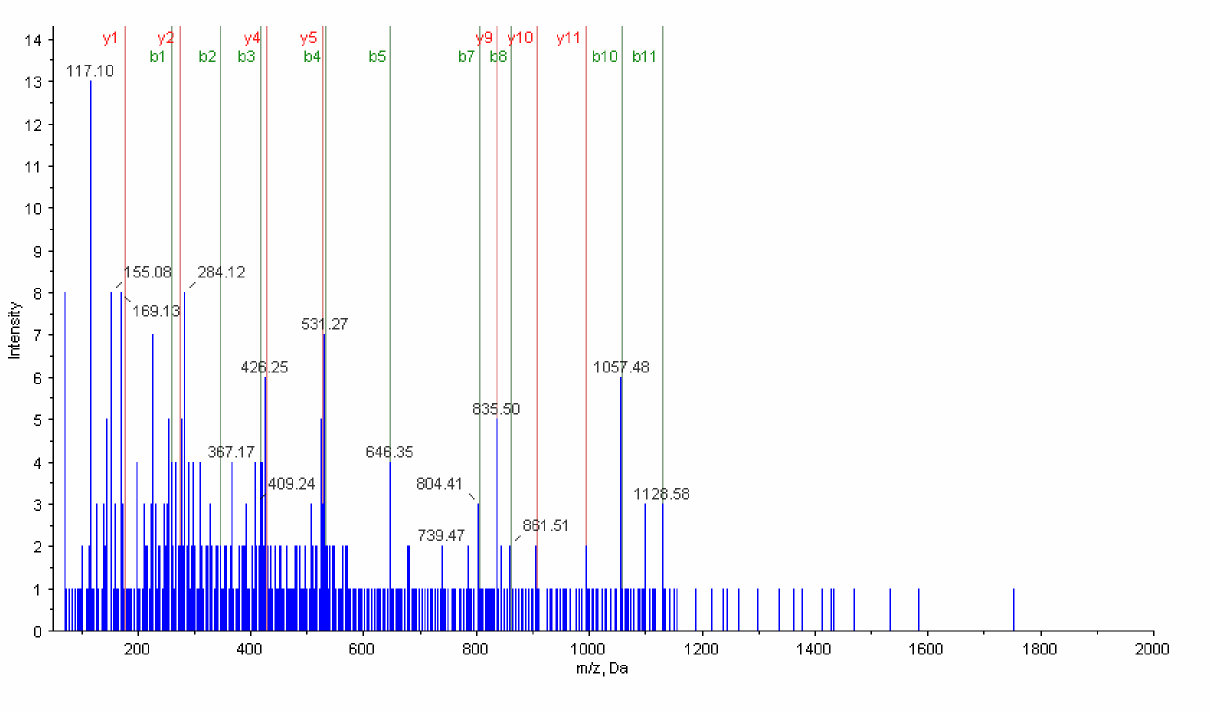

Protein: SCO7638, Eno2 [1 of 1]

Protein Quant | Protein ID | Spectra | Summary Statistics

Spectrum List

Spectrum	Time	Prec MW	Prec m/z	Prec z	Best Sequence	Modifications	Conf	Theor MW	z	115:114	%Err 115:114	116:
30.1.1.1036.3	39.126	2254.1643	752.3954	3	DLLAAGHSTGVGDEGGFAPALR	iTRAQ@N-term	99	2254.1463	3	0.9177	19.07	

Peptide ID Hypotheses

Conf	Sc	Prot N	Sequence	Modifications	Theor MW	Theor m/z	z	ΔMass
99	20	814	DLLAAGHSTGVGDEGGFAPALR	iTRAQ@N-term	2254.1463	752.3894	3	0.0180
< 1	8		AAGTLFACGALGMLAGDVTIGR	iTRAQ@N-term, MMTS(C)@8	2254.1393	752.3871	3	0.0250
< 1	8		HVPGDGIDPGPVPGMGPAERR	iTRAQ@N-term	2254.1398	752.3872	3	0.0245
< 1	7		LDSPALATVADAVVTAAADALR	iTRAQ@N-term	2254.2290	752.4169	3	-0.0647
< 1	7		LRDAGRSVLVVSHLAYDIDR	No iTRAQ@N-term	2254.2181	752.4134	3	-0.0538

Precursor MS Region

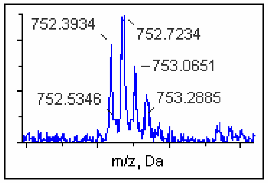

Fragmentation Evidence for Peptide

ITR DLLAAGHSTGVGDEGGFAPALR

Res	b	y
D	260.1363	2255.1537
L	373.2203	1996.0247
L	486.3044	1882.9406
A	557.3415	1769.8565
A	628.3786	1698.8194
G	685.4001	1627.7823
H	822.4590	1570.7608
S	909.4910	1433.7019
T	1010.5387	1346.6699
G	1067.5602	1245.6222
V	1166.6286	1188.6008
G	1223.6501	1089.5323
D	1338.6770	1032.5109
E	1467.7196	917.4839
G	1524.7411	788.4413
G	1581.7625	731.4199
F	1728.8309	674.3984
A	1799.8681	527.3300
P	1896.9208	456.2929
A	1967.9579	359.2401
L	2081.0420	288.2030
R	2237.1431	175.1190

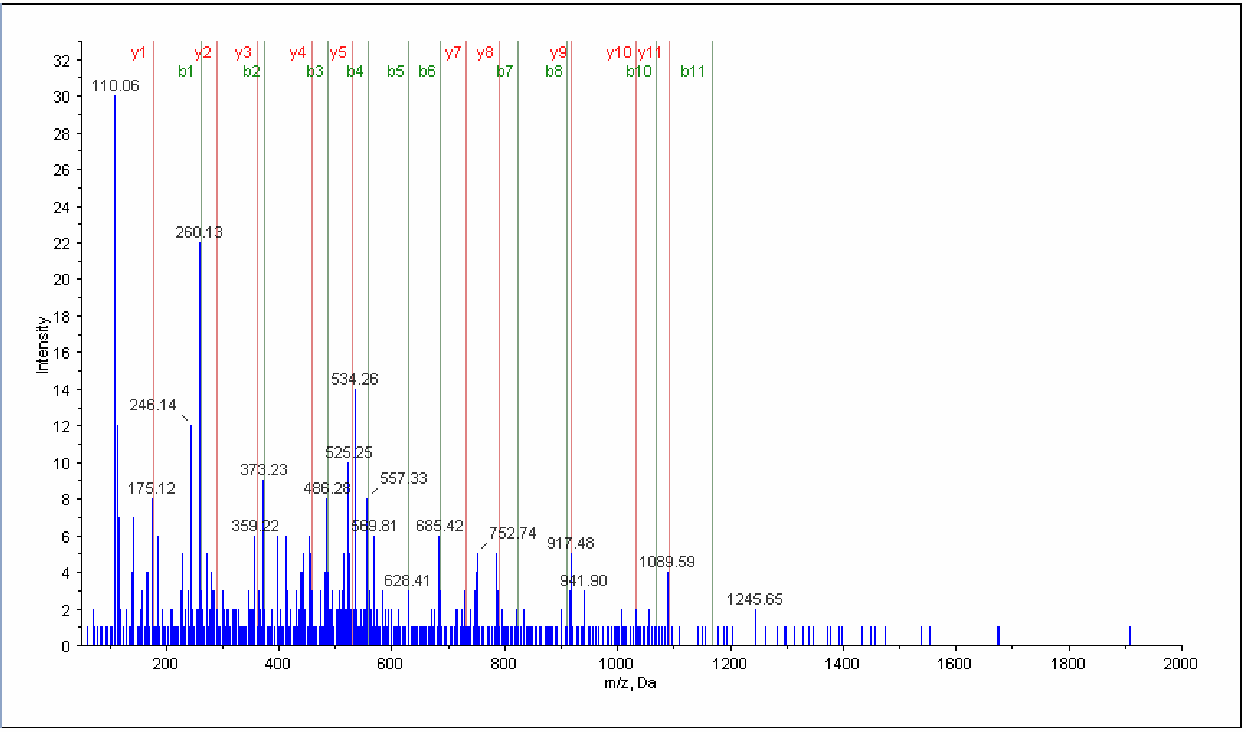

Supplement: Figure S4 — MS/MS fragmentation spectra for single peptide protein hits. This file contains a series of MS/MS fragmentation spectra for single peptide protein hits shown in Figure 5. The list also includes those single peptide hits that were sampled multiple times (i.e. multiple spectral evidence single peptide hits). In such cases, the protein number is repeated as many times as the number of spectra contributing for a given peptide. (4.28 MB PDF) [file pone.0002097.s004.pdf]
