# Supplementary material for: Uncovering Genes with Divergent mRNA-Protein Dynamics in Streptomyces coelicolor
Source: PLoS One. 2008 May 7;3(5):e2097. doi: 10.1371/journal.pone.0002097 (PMC2367054; doi:10.1371/journal.pone.0002097)
Supplement: Table S1 — List of genes with probable divergent mRNA-protein behavior discovered by PCA The list shows only data for which at least two unique peptide identifications in mass spectrometry data are available. Please refer to supplementary tables S2 and S3 for complete dataset including proteins with single peptide hits and concordant mRNA-protein behavior. (0.19 MB PDF) [file pone.0002097.s005.pdf]

# Table S1. List of genes with probable divergent mRNA-protein behavior discovered by PCA

The list shows only data for which at least two unique peptide identifications in mass spectrometry data are available. Please refer to supplementary tables S2 and S3 for complete dataset including proteins with single peptide hits and concordant mRNA-protein behavior.

<u>Column headings</u>

| | |
|---|---|
| Gene Name: | SCO number from Sanger annotation |
| Abb.: | Gene abbreviation from Sanger, KEGG, ScoCyc annotations |
| Category: | Gene category from Sanger annotation |
| Annotation: | Gene function from Sanger annotation |
| UPC: | Number of unique peptide count supporting protein identification |
| TP-m: | Number of time points with mRNA data available |
| TP-p: | Number of time points with protein data available |
| PC1 - m: | PCA 1 value from mRNA data |
| PC2 - m: | PCA 2 value from mRNA data |
| PC1 - p: | PCA 1 value from protein data |
| PC2 - p: | PCA 2 value from protein data |

Set categories shown in this Table and Tables S2, S3 are defined based on mRNA-protien divergence trends shown below with reference to Figure 4 (genes in set G – those with mRNA-protein concordance – are not shown in Table S1; refer to Tables S2, S3 for complete dataset)

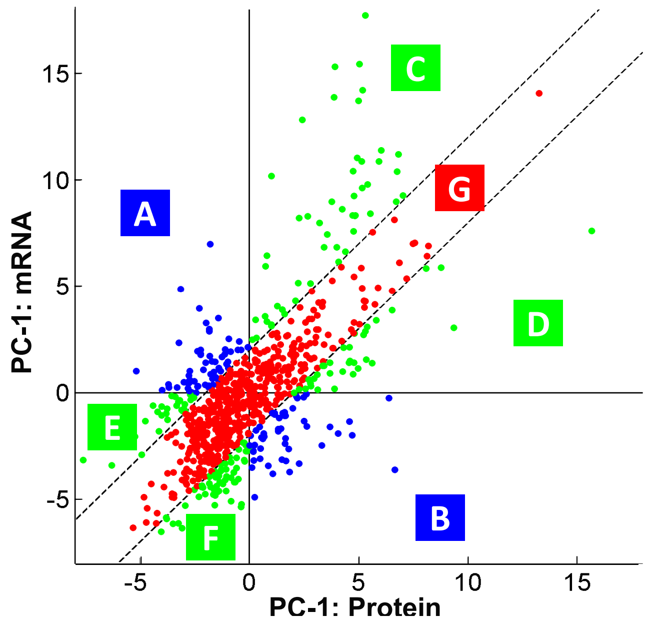


*Table begins next page…*

| Gene Name | Abb. | Category | Annotation | UPC | TP-m | TP-p | PC1-m | PC2-m | PC1-p | PC2-p |
|---|---|---|---|---|---|---|---|---|---|---|
| | | | ***Genes in Set A*** | | | | | | | |
| SCO1116 | | Conserved in organism other than Escherichia coli | hypothetical protein | 4 | 8 | 8 | 0.27 | -0.83 | -1.87 | 0.77 |
| SCO1441 | *ribAB* | Riboflavin | 3,4-dihydroxy-2-butanone 4-phosphate synthase | 2 | 8 | 4 | 1.45 | 0.22 | -0.99 | 1.17 |
| SCO1484 | *pyrAA* | Pyrimidine ribonucleotide biosynthesis | carbamoyl-phosphate synthase, pyrimidine-specific | 2 | 8 | 4 | 2.02 | 1.89 | -2.23 | -0.39 |
| SCO1793 | | Gram +ve exported/lipoprotein | hypothetical protein "hypothetical protein (putati | 6 | 8 | 8 | 1.17 | -0.27 | -0.97 | -0.41 |
| SCO1831 | | Not classified (included putative assignments) | putative oxidoreductase | 2 | 8 | 4 | 0.12 | 0.07 | -3.99 | 1.02 |
| SCO1922 | | Transport/binding proteins | putative ABC transporter ATP-binding subunit | 9 | 8 | 8 | 0.22 | -0.68 | -2.81 | -0.14 |
| SCO1958 | *uvrA* | DNA - replication, repair, restr./modific'n | ABC excision nuclease subunit A | 2 | 8 | 6 | 1.96 | -0.29 | -2.38 | -0.29 |
| SCO2013 | | Response regulator | putative two-component system response regulator | 4 | 8 | 6 | 0.03 | -0.80 | -2.64 | 0.41 |
| SCO2077 | | No homologues | hypothetical protein | 4 | 8 | 7 | 0.67 | -0.08 | -1.68 | 0.30 |
| SCO2161 | | Conserved in organism other than Escherichia coli | conserved hypothetical protein | 2 | 8 | 7 | 0.54 | 0.74 | -2.51 | 0.34 |
| SCO2180 | *pdhL* | Pyruvate dehydrogenase | putative dihydrolipoamide dehydrogenase | 21 | 8 | 8 | 2.03 | 1.16 | -0.53 | -0.05 |
| SCO2183 | *aceE1* | Pyruvate dehydrogenase | putative pyruvate dehydrogenase E1 component | 14 | 8 | 8 | 0.40 | -0.48 | -3.70 | -0.25 |
| SCO2589 | | Not classified (included putative assignments) | putative glycosyl transferase | 2 | 7 | 4 | 0.38 | -1.61 | -2.02 | 0.34 |
| SCO2620 | | Cell division | putative cell division trigger factor | 24 | 8 | 8 | 1.02 | -0.04 | -2.73 | 0.46 |
| SCO3622 | | Not classified (included putative assignments) | putative aminotransferase | 2 | 8 | 7 | 1.02 | 0.19 | -1.31 | 0.08 |
| SCO3801 | | Degradation of proteins, peptides, glycoproteins | putative aminopeptidase | 2 | 7 | 4 | 4.88 | 1.32 | -3.14 | -0.11 |
| SCO3966 | | Gram +ve exported/lipoprotein | putative secreted protein | 2 | 8 | 4 | 0.63 | -1.45 | -1.43 | -0.70 |
| SCO4068 | *purD* | Purine ribonucleotide biosynthesis | phosphoribosylamine-glycine ligase (EC 6.3.4.13) | 3 | 8 | 8 | 7.01 | 0.78 | -1.80 | -0.66 |
| SCO4071 | *purC* | Purine ribonucleotide biosynthesis | phosphoribosylaminoimidazole-succinocarboxamide s | 2 | 8 | 6 | 3.51 | -3.39 | -1.43 | 0.24 |
| SCO4185 | | Conserved in organism other than Escherichia coli | conserved hypothetical protein | 2 | 7 | 4 | 1.02 | -0.59 | -5.17 | 0.92 |

| | | | | | | | | | | |
|---|---|---|---|---|---|---|---|---|---|---|
| SCO4232 | | Other regulation | putative transcriptional factor regulator | 2 | 8 | 8 | 1.84 | -0.49 | -2.22 | -0.55 |
| SCO4266 | | Fatty acids | putative oxidoreductase | 2 | 8 | 4 | 2.14 | -1.40 | -0.07 | -3.58 |
| SCO4583 | | Menaquinone, ubiquinone | putative polyprenyl diphosphate synthase | 3 | 8 | 6 | 0.46 | -1.23 | -2.77 | 0.69 |
| SCO4683 | *gdhA* | Glutamate | NADP-specific glutamate dehydrogenase | 4 | 8 | 8 | 2.41 | -0.57 | -0.45 | 0.33 |
| SCO4932 | *hutH* | Amino acids | histidine ammonia-lyase | 3 | 8 | 4 | 0.38 | -0.21 | -1.67 | -0.52 |
| SCO4934 | | Gram +ve exported/lipoprotein | putative lipoprotein | 5 | 8 | 8 | 1.42 | -0.55 | -2.00 | -0.03 |
| SCO5044 | *fumB* | TCA cycle | fumarate hydratase class I | 5 | 8 | 7 | 0.97 | 0.91 | -1.62 | -0.23 |
| SCO5424 | *ackA* | Carbon compounds | acetate kinase | 2 | 8 | 4 | 0.49 | -0.08 | -2.75 | 0.33 |
| SCO5645 | | Conserved in organism other than Escherichia coli | conserved hypothetical protein SC6A9.22c | 2 | 8 | 4 | 0.26 | -0.14 | -3.31 | 0.21 |
| SCO5769 | *recA* | Chromosome replication | recombinase A | 3 | 8 | 7 | 0.88 | -0.82 | -1.78 | -0.95 |
| SCO5971 | | Conserved in organism other than Escherichia coli | conserved hypothetical protein | 5 | 8 | 7 | 2.88 | 0.09 | -1.84 | 0.34 |
| SCO6148 | | No homologues | hypothetical protein SC1A9.12 | 2 | 8 | 6 | 1.17 | 1.36 | -1.39 | 0.00 |
| SCO6471 | | Pool, multipurpose conversions of intermed. met'm | putative citratelyase | 2 | 7 | 6 | 1.93 | 1.30 | -0.85 | -1.10 |
| SCO6662 | *tal1* | Non-oxidative branch, pentose pwy | transaldolase | 6 | 8 | 6 | 0.09 | -0.10 | -2.74 | 0.50 |
| | | | ***Genes in Set B*** | | | | | | | |
| SCO1517 | | Gram +ve exported/lipoprotein | putative secreted protein | 4 | 8 | 8 | -3.13 | -0.10 | 1.49 | -0.65 |
| SCO1638 | *fkbP* | Protein modification | peptidyl-prolyl cis-trans isomerase | 4 | 8 | 8 | -2.05 | 1.23 | 0.67 | -0.06 |
| SCO2014 | *pyk1* | Glycolysis | pyruvate kinase | 5 | 8 | 8 | -1.67 | 1.50 | 1.45 | 1.63 |
| SCO2094 | | Other regulation | putative regulatory protein | 2 | 8 | 6 | -0.90 | -0.61 | 1.61 | 0.49 |
| SCO2389 | *acpP* | Fatty acid and phosphatidic acid biosynth | acyl carrier protein | 3 | 8 | 6 | -2.63 | 0.82 | 1.81 | 0.46 |
| SCO2780 | | Gram +ve exported/lipoprotein | putative secreted protein | 2 | 8 | 6 | -3.50 | -0.40 | 0.12 | 0.21 |
| SCO3013 | | Response regulator | putative two-component system response regulator | 3 | 8 | 8 | -2.44 | 0.59 | 0.25 | 0.35 |
| SCO3659 | | No homologues | hypothetical protein | 4 | 8 | 8 | -2.12 | 0.84 | 1.66 | 0.53 |
| SCO3815 | *bkdC1* | Pyruvate dehydrogenase | putative dihydrolipoamide acyltransferase compone | 3 | 8 | 8 | -1.80 | 0.80 | 1.33 | 0.66 |
| SCO3817 | *bkdA1* | Pyruvate dehydrogenase | putative branched-chain alpha keto acid dehydroge | 3 | 8 | 6 | -2.01 | 1.49 | 0.06 | 0.34 |
| SCO3977 | | Degradation of proteins, peptides, glycoproteins | putative protease (putative secreted protein) | 5 | 8 | 8 | -0.21 | 0.51 | 2.49 | -0.21 |
| SCO4856 | *dhsA* | TCA cycle | putative succinate dehydrogenase flavoprotein sub | 16 | 8 | 8 | -3.17 | 0.52 | 0.21 | -0.85 |
| SCO4885 | | Gram +ve exported/lipoprotein | putative lipoprotein | 18 | 8 | 8 | -4.90 | 0.84 | 0.25 | -0.08 |
| SCO4912 | | Not classified (included putative assignments) | putative aldehyde dehydrogenase | 3 | 8 | 8 | -0.94 | 1.30 | 1.15 | 0.00 |

| | | | | | | | | | | |
|---|---|---|---|---|---|---|---|---|---|---|
| SCO4919 | | Not classified (included putative assignments) | putative oxidoreductase | 4 | 8 | 7 | -1.08 | -0.10 | 1.66 | -1.20 |
| SCO4926 | *pccB* | Fatty acid and phosphatidic acid biosynth | propionyl-CoA carboxylase complex B subunit | 2 | 8 | 4 | -0.24 | 1.23 | 2.20 | 0.27 |
| SCO5113 | *bldKB* | Transport/binding proteins | BldKB, putative ABC transport system lipoprotein | 25 | 8 | 8 | -1.90 | 0.78 | 0.66 | 0.01 |
| SCO5169 | | Not classified (included putative assignments) | putative ATP-binding protein | 3 | 8 | 6 | -3.79 | 0.31 | 1.07 | 0.52 |
| SCO5204 | | Gram +ve membrane | hypothetical protein (fragment) "putative integral | 9 | 8 | 7 | -1.07 | 1.25 | 1.61 | -0.56 |
| SCO5464 | | Conserved in organism other than Escherichia coli | Not Specified | 4 | 8 | 8 | -2.47 | 1.04 | 3.33 | 0.07 |
| SCO5498 | *gatC* | Amino acyl tRNA syn; tRNA modific'n | probable Glu-tRNAGln amidotransferase subunit C | 2 | 8 | 6 | -2.75 | 0.93 | 0.19 | 0.77 |
| SCO5541 | *cvnD1* | Not classified (included putative assignments) | putative ATP-GTP binding protein | 2 | 8 | 4 | -3.10 | -0.40 | 0.49 | -0.19 |
| SCO5744 | *dapA* | Lysine | putative dihydrodipicolinate synthase | 3 | 7 | 7 | -1.70 | 0.26 | 1.32 | 0.71 |
| SCO5776 | *gluB* | Transport/binding proteins | glutamate binding protein | 22 | 8 | 8 | -1.60 | 0.13 | 3.69 | 0.36 |
| SCO5777 | *gluA* | Transport/binding proteins | glutamate uptake system ATP-binding protein | 7 | 7 | 8 | -3.31 | 0.45 | 2.28 | 1.07 |
| SCO5836 | | DNA - replication, repair, restr./modific'n | DNA gyrase-like protein | 3 | 8 | 7 | -0.96 | -0.87 | 1.54 | 0.69 |
| SCO5841 | *ptsH* | Transport/binding proteins | phosphocarrier protein hpr | 2 | 8 | 4 | -0.96 | 0.17 | 1.05 | 1.08 |
| SCO5869 | | Conserved in organism other than Escherichia coli | hypothetical protein SC2E9.10 | 6 | 8 | 4 | -2.47 | 0.46 | 0.02 | 0.25 |
| SCO6009 | | Transport/binding proteins | solute-binding protein | 14 | 8 | 8 | -1.91 | 1.40 | 4.06 | -0.11 |
| SCO6010 | | Transport/binding proteins | probable ABC-transport system ATP binding protein | 2 | 8 | 8 | -1.57 | 0.89 | 2.63 | 0.96 |
| SCO6011 | | Transport/binding proteins | probable ABC-type transmembrane transport protein | 3 | 8 | 7 | -1.99 | -0.19 | 4.70 | 0.40 |
| SCO6124 | | No homologues | hypothetical protein SC9B2.11 | 4 | 8 | 7 | -1.35 | 0.39 | 4.58 | 2.50 |
| SCO6451 | | Transport/binding proteins | putative substrate binding protein | 4 | 8 | 7 | -2.61 | 0.38 | 0.52 | -0.81 |
| SCO7463 | *cvnA13* | Sensor kinase | putative sensor histidine kinase | 2 | 8 | 4 | -2.08 | 0.47 | 0.56 | 0.76 |

***Genes in Set C***

| | | | | | | | | | | |
|---|---|---|---|---|---|---|---|---|---|---|
| SCO0200 | | Conserved in organism other than Escherichia coli | conserved hypothetical protein SCJ12.12c | 3 | 5 | 8 | 5.18 | 1.95 | 2.24 | -1.99 |
| SCO0379 | *katA* | Detoxification | catalase (EC 1.11.1.6) | 8 | 8 | 7 | 6.46 | -0.79 | 0.79 | -1.73 |
| SCO0560 | *cpeB* | Detoxification | catalase/peroxidase | 6 | 8 | 8 | 5.15 | 1.15 | 2.80 | 0.13 |
| SCO0608 | | Other regulation | putative regulatory protein | 7 | 8 | 8 | 8.01 | -0.08 | 3.21 | 0.31 |
| SCO1340 | | Conserved in organism other than Escherichia coli | conserved hypothetical protein | 2 | 8 | 4 | 3.21 | 1.08 | 1.00 | 0.31 |

| | | | | | | | | | | |
|---|---|---|---|---|---|---|---|---|---|---|
| SCO1644 | *prcB* | Degradation of proteins, peptides, glycoproteins | 20S proteasome beta-subunit precursor | 3 | 8 | 7 | 3.61 | 1.30 | 0.72 | -0.41 |
| SCO1648 | *arc* | Chaperones | AAA ATPase | 10 | 8 | 8 | 7.60 | 1.95 | 4.77 | -0.36 |
| SCO2008 | | Amino acids and amines | putative branched chain amino acid binding protei | 25 | 8 | 8 | 11.40 | -0.64 | 6.04 | -1.01 |
| SCO2286 | | Not classified (included putative assignments) | putative alkaline phosphatase | 2 | 8 | 8 | 8.32 | 4.04 | 2.68 | 0.24 |
| SCO2517 | | Response regulator | putative two-component system response regulator | 4 | 8 | 8 | 6.18 | -0.18 | 4.08 | -1.50 |
| SCO2519 | | Gram +ve membrane | putative integral membrane protein | 8 | 8 | 8 | 14.23 | -0.85 | 5.19 | -0.37 |
| SCO3767 | | Conserved in organism other than Escherichia coli | conserved hypothetical protein | 13 | 8 | 8 | 4.32 | -1.12 | 2.12 | -0.38 |
| SCO3790 | | Conserved in organism other than Escherichia coli | conserved hypothetical protein | 13 | 8 | 8 | 8.99 | 3.42 | 6.72 | 0.96 |
| SCO4142 | *pstS* | Not classified (included putative assignments) | phosphate-binding protein precursor | 30 | 8 | 8 | 18.22 | 2.27 | 7.18 | 0.44 |
| SCO4545 | | Conserved in organism other than Escherichia coli | conserved hypothetical protein | 9 | 8 | 8 | 8.44 | 0.10 | 5.54 | 0.38 |
| SCO4881 | | Polysaccharides - (cytoplasmic) | putative polysaccharide biosynthesis related prot | 14 | 8 | 8 | 9.81 | 2.94 | 5.42 | 1.90 |
| SCO5071 | *actVI-ORFA* | PKS | hydroxylacyl-CoA dehydrogenase | 2 | 8 | 6 | 8.23 | -5.56 | 2.28 | -1.71 |
| SCO5073 | *actVIORF2* | PKS | putative oxidoreductase | 5 | 8 | 8 | 10.44 | -6.39 | 4.75 | -3.41 |
| SCO5074 | *actVIORF3* | PKS | putative dehydratase | 3 | 8 | 7 | 12.84 | -6.29 | 2.43 | -2.96 |
| SCO5075 | *actVIORF4* | PKS | putative oxidoreductase | 2 | 8 | 7 | 8.34 | -5.65 | 4.83 | -2.09 |
| SCO5078 | *actVA3* | PKS | hypothetical protein | 4 | 8 | 8 | 8.64 | -5.49 | 4.26 | -1.83 |
| SCO5079 | *actVA4* | PKS | conserved hypothetical protein | 4 | 8 | 8 | 10.89 | -5.66 | 5.15 | -2.39 |
| SCO5080 | *actVA5* | PKS | putative hydrolase | 7 | 7 | 8 | 8.36 | -4.90 | 4.73 | -1.43 |
| SCO5087 | *actIORF1* | PKS | actinorhodin polyketide beta-ketoacyl synthase alpha | 3 | 8 | 6 | 9.26 | -5.94 | 4.79 | -2.39 |
| SCO5088 | *actIORF2* | PKS | actinorhodin polyketide beta-ketoacyl synthase beta | 4 | 8 | 4 | 9.65 | -6.14 | 5.16 | -3.34 |
| SCO5178 | | Thiamin | putative sulfurylase | 7 | 8 | 8 | 3.70 | 1.24 | 1.61 | -0.33 |
| SCO5396 | | Transport/binding proteins | putative cellulose-binding protein | 8 | 8 | 8 | 2.50 | 1.01 | 0.14 | 0.36 |
| SCO5657 | | Osmotic adaptation | aldehyde dehydrogenase | 5 | 8 | 8 | 2.59 | 0.58 | 0.32 | 0.44 |
| SCO5890 | *redN* | PKS | putative 8-amino-7-oxononanoate synthase | 7 | 8 | 8 | 10.41 | -0.44 | 6.77 | 0.05 |
| SCO5893 | *redK* | PKS | oxidoreductase | 5 | 8 | 8 | 9.00 | 0.83 | 3.78 | -1.83 |
| SCO5895 | *redI* | PKS | putative methyltransferase | 11 | 8 | 8 | 11.04 | 0.99 | 4.93 | -0.97 |
| SCO5896 | *redH* | PKS | phosphoenolpyruvate-utilizing enzyme | 12 | 8 | 8 | 10.89 | 1.62 | 5.93 | 0.37 |
| SCO5897 | *redG* | PKS | putative oxidase | 9 | 8 | 8 | 11.21 | -0.35 | 6.83 | -0.74 |
| SCO6273 | | PKS | putative type I polyketide synthase | 26 | 8 | 8 | 6.85 | 3.41 | 3.43 | -0.14 |
| SCO6275 | | PKS | putative type I polyketide synthase | 43 | 8 | 8 | 6.85 | 2.66 | 4.01 | 0.33 |

| | | | | | | | | | | |
|---|---|---|---|---|---|---|---|---|---|---|
| SCO6276 | | Secondary metabolism | putative secreted protein | 36 | 8 | 8 | 15.45 | 0.32 | 5.05 | -0.16 |
| SCO6277 | | Secondary metabolism | putative epoxide hydrolase | 10 | 8 | 8 | 15.33 | 1.01 | 3.92 | -0.84 |
| SCO6279 | | Secondary metabolism | putative diaminobutyrate-pyruvate aminotransferas | 25 | 8 | 8 | 13.74 | 1.72 | 5.01 | -0.82 |
| SCO6282 | | PKS | putative 3-oxoacyl-[acyl-carrier protein] reducta | 40 | 7 | 8 | 17.73 | -0.77 | 5.32 | -1.41 |
| SCO6283 | | Secondary metabolism | conserved hypothetical protein | 7 | 8 | 8 | 13.90 | 0.58 | 3.89 | -1.97 |
| SCO7536 | | Gram +ve membrane | putative integral membrane protein. | 2 | 8 | 7 | 8.36 | -2.10 | 4.84 | -0.30 |

## ***Genes in Set D***

| | | | | | | | | | | |
|---|---|---|---|---|---|---|---|---|---|---|
| SCO0546 | *pyc* | Gluconeogenesis | pyruvate carboxylase | 3 | 8 | 7 | 0.86 | 0.64 | 3.10 | 1.01 |
| SCO1222 | | Conserved in organism other than Escherichia coli | conserved hypothetical protein | 2 | 8 | 7 | 3.88 | 1.02 | 6.53 | 1.28 |
| SCO1223 | *rocD* | Arginine | ornithine aminotransferase | 3 | 8 | 8 | 2.15 | 0.47 | 4.57 | 1.18 |
| SCO1640 | | Conserved in organism other than Escherichia coli | conserved hypothetical protein SCI41.23c | 2 | 8 | 8 | 5.90 | 1.92 | 8.79 | 1.66 |
| SCO2582 | | Conserved in organism other than Escherichia coli | conserved hypothetical protein SCC123.20. | 2 | 8 | 4 | 0.87 | 0.39 | 4.74 | -0.88 |
| SCO2792 | *adpA* | AraC | araC-family transcriptional regulator | 3 | 8 | 7 | 2.79 | 1.36 | 4.81 | 0.20 |
| SCO2855 | | No homologues | hypothetical protein SCE20.29. | 2 | 8 | 7 | 1.41 | 0.03 | 3.68 | -0.98 |
| SCO2884 | | Electron transport | putative cytochrome P450. | 8 | 8 | 8 | 3.42 | 0.23 | 5.80 | 0.12 |
| SCO4252 | | No homologues | hypothetical protein SCD8A.25c | 4 | 8 | 8 | 2.73 | 2.96 | 5.23 | 0.83 |
| SCO4253 | | Conserved in organism other than Escherichia coli | conserved hypothetical protein SCD8A.26c | 21 | 8 | 8 | 1.50 | 2.61 | 4.60 | 0.88 |
| SCO4677 | | Other regulation | putative regulatory protein | 3 | 8 | 8 | 7.63 | -0.86 | 15.67 | 3.17 |
| SCO4875 | | Polysaccharides - (cytoplasmic) | putative sugar transferase (fragment) "putative tr | 2 | 7 | 4 | 1.40 | -1.16 | 5.63 | 1.06 |
| SCO4877 | | No homologues | hypothetical protein 2SCK8.03c | 3 | 8 | 6 | 3.10 | 1.35 | 5.40 | 1.72 |
| SCO4884 | | Gram +ve exported/lipoprotein | putative lipoprotein | 2 | 8 | 8 | 1.14 | 0.87 | 4.98 | -0.71 |
| SCO5479 | | Transport/binding proteins | oligopeptide ABC transporter ATP-binding protein | 5 | 8 | 8 | 0.45 | 1.42 | 2.79 | -0.18 |
| SCO5480 | | Transport/binding proteins | oligopeptide ABC transporter ATP-binding protein | 5 | 8 | 8 | 0.63 | 0.27 | 2.71 | 0.47 |
| SCO5874 | | Conserved in organism other than Escherichia coli | putative membrane protein | 2 | 8 | 4 | 0.01 | -0.15 | 2.06 | -0.73 |
| SCO5884 | | PKS | hypothetical protein SC3F7.04c | 2 | 8 | 4 | 3.07 | -0.29 | 9.36 | 0.20 |
| SCO6445 | | Phospholipids | putative inositol monophosphatase | 5 | 8 | 8 | 1.01 | 1.19 | 4.27 | 1.87 |
| SCO6638 | | Conserved in organism other than Escherichia coli | hypothetical protein SC4G2.12c | 3 | 8 | 8 | 1.27 | 0.10 | 3.37 | 0.22 |
| SCO7057 | | Not classified (included putative assignments) | putative esterase | 2 | 8 | 7 | 1.28 | 0.18 | 3.41 | 1.63 |
| SCO7417 | | Electron transport | putative cytochrome P450-family | 2 | 8 | 4 | 0.20 | -1.12 | 2.58 | 1.01 |

protein.

**Genes in Set E**

| | | | | | | | | | | |
|---|---|---|---|---|---|---|---|---|---|---|
| SCO1505 | *rspD* | Ribosomal proteins - synthesis, modification | 30S ribosomal protein S4 | 9 | 8 | 8 | -4.81 | 1.42 | -1.56 | -0.35 |
| SCO1598 | *rplT* | Ribosomal proteins - synthesis, modification | 50S ribosomal protein L20 | 6 | 8 | 8 | -4.09 | 0.24 | -1.10 | -0.40 |
| SCO1651 | | Conserved in organism other than Escherichia coli | conserved hypothetical protein SCI41.34c | 3 | 7 | 7 | -3.21 | -0.29 | -0.22 | -0.01 |
| SCO1655 | | Transport/binding proteins | putative lipoprotein oligopeptide binding protein | 6 | 8 | 8 | -3.58 | 0.01 | -1.06 | 0.15 |
| SCO1866 | | Osmotic adaptation | putative condensing enzyme | 2 | 8 | 6 | -4.56 | 0.70 | -1.58 | 1.43 |
| SCO1921 | | Incorporation metal ions | putative aminotransferase | 5 | 8 | 7 | -3.33 | 0.60 | -1.32 | -0.39 |
| SCO1998 | *rpsA* | Ribosomal proteins - synthesis, modification | 30S ribosomal protein S1 | 23 | 8 | 8 | -4.25 | 0.19 | -1.83 | -0.21 |
| SCO2088 | *murF* | Gram +ve peptidoglycan, teichoic acid | putative UDP-N-acetylmuramoylalanyl-D-glutamyl- 2 | 4 | 8 | 7 | -4.18 | -0.20 | -0.69 | 0.24 |
| SCO2155 | *cox1* | Aerobic respiration | putative cytochrome c oxidase subunit I | 3 | 8 | 8 | -4.04 | 1.02 | -0.51 | -0.55 |
| SCO2179 | | Degradation of proteins, peptides, glycoproteins | putative aminopeptidase | 16 | 8 | 8 | -3.15 | -0.21 | -0.23 | -0.09 |
| SCO2387 | *fabD* | Fatty acid and phosphatidic acid biosynth | malonyl CoA:acyl carrier protein malonyltransfera | 2 | 8 | 8 | -6.35 | -0.01 | -3.07 | -0.06 |
| SCO2390 | *fabF* | Fatty acid and phosphatidic acid biosynth | 3-oxoacyl-[acyl-carrier-protein] synthase II | 12 | 8 | 8 | -6.15 | 0.22 | -3.49 | 0.62 |
| SCO2504 | *glyS* | Amino acyl tRNA syn; tRNA modific'n | glycyl-tRNA synthetase | 11 | 8 | 8 | -4.41 | -0.35 | -1.78 | 0.16 |
| SCO2593 | | No homologues | hypothetical protein SCC88.04c | 3 | 8 | 6 | -2.65 | 0.08 | -0.49 | -0.55 |
| SCO2677 | | Transport/binding proteins | putative ABC transpoter ATP-binding protein | 8 | 8 | 8 | -3.26 | 0.76 | -0.84 | -0.15 |
| SCO2950 | *hup* | Other regulation | DNA-binding protein Hu (hs1) | 10 | 8 | 8 | -5.99 | 1.30 | -2.50 | 0.27 |
| SCO3123 | *prsA2* | Purine ribonucleotide biosynthesis | ribose-phosphate pyrophosphokinase | 7 | 8 | 8 | -4.41 | -0.89 | -2.18 | -0.36 |
| SCO3124 | | Ribosomal proteins - synthesis, modification | ribosomal L25p family protein | 11 | 8 | 8 | -5.24 | -0.17 | -0.36 | -0.37 |
| SCO3182 | *gtaB* | Carbon compounds | UTP-glucose-1-phosphate uridylyltransferase | 8 | 8 | 8 | -6.50 | -0.48 | -1.54 | 0.55 |
| SCO3409 | *ppa* | Phosphorus compounds | putative inorganic pyrophosphatase | 5 | 8 | 8 | -4.18 | 0.61 | -0.65 | 0.65 |
| SCO3615 | *ask* | Threonine | putative aspartokinase | 7 | 8 | 8 | -3.02 | 1.42 | -0.89 | 0.15 |
| SCO3877 | | Oxidative branch, pentose pathway | putative 6-phosphogluconate dehydrogenase | 10 | 8 | 8 | -4.53 | 0.08 | -2.52 | 1.00 |
| SCO3906 | *rpsF* | Ribosomal proteins - synthesis, modification | putative 30S ribosomal protein S6 | 6 | 8 | 8 | -3.94 | 0.27 | -1.83 | -0.23 |
| SCO3909 | *rplI* | Ribosomal proteins - synthesis, modification | 50S ribosomal protein L9 | 11 | 8 | 8 | -6.13 | 0.95 | -3.24 | 0.60 |

| | | | | | | | | | | |
|---|---|---|---|---|---|---|---|---|---|---|
| SCO3974 | | No homologues | hypothetical protein | 6 | 8 | 7 | -3.86 | -0.38 | -1.74 | 0.17 |
| SCO4328 | | Gram +ve exported/lipoprotein | putative lipoprotein | 2 | 8 | 8 | -4.65 | 0.29 | -1.46 | -1.26 |
| SCO4647 | *nusG* | RNA synthesis, modification, DNA transcript'n | transcription antitermination protein | 5 | 8 | 8 | -5.29 | -0.81 | -2.93 | 0.45 |
| SCO4652 | *rplJ* | Ribosomal proteins - synthesis, modification | 50S ribosomal protein L10 | 8 | 8 | 8 | -4.74 | -0.30 | -2.02 | 0.22 |
| SCO4653 | *rplL* | Ribosomal proteins - synthesis, modification | 50S ribosomal protein L7/L12 | 9 | 8 | 8 | -5.90 | -0.06 | -1.64 | 0.14 |
| SCO4654 | *rpoB* | RNA synthesis, modification, DNA transcript'n | DNA-directed RNA polymerase beta chain | 50 | 8 | 8 | -4.37 | -0.33 | -1.50 | -0.50 |
| SCO4655 | *rpoC* | RNA synthesis, modification, DNA transcript'n | DNA-directed RNA polymerase beta' chain (fragment | 52 | 8 | 8 | -3.96 | -0.49 | -1.54 | -0.16 |
| SCO4659 | *rspL* | Ribosomal proteins - synthesis, modification | 30S ribosomal protein S12 | 7 | 8 | 8 | -4.28 | -0.03 | -1.21 | -0.48 |
| SCO4660 | *rspG* | Ribosomal proteins - synthesis, modification | 30S ribosomal protein S7 | 14 | 8 | 8 | -4.61 | -1.13 | -1.39 | -0.29 |
| SCO4702 | *rplC* | Ribosomal proteins - synthesis, modification | 50S ribosomal protein L3 | 9 | 8 | 8 | -4.57 | -0.81 | -1.61 | 0.06 |
| SCO4708 | | Ribosomal proteins - synthesis, modification | 30S ribosomal protein S3 | 15 | 8 | 8 | -3.31 | 0.25 | -1.10 | -0.13 |
| SCO4709 | *rplP* | Ribosomal proteins - synthesis, modification | 50S ribosomal protein L16 | 6 | 8 | 8 | -3.58 | -0.81 | -1.27 | -0.61 |
| SCO4716 | *rpsH* | Ribosomal proteins - synthesis, modification | 30S ribosomal protein S8 | 10 | 8 | 8 | -3.65 | -0.44 | -1.29 | -0.48 |
| SCO4719 | *rpsE* | Ribosomal proteins - synthesis, modification | 30S ribosomal protein S5 | 17 | 8 | 8 | -4.18 | -0.56 | -1.36 | -0.02 |
| SCO4721 | *rplO* | Ribosomal proteins - synthesis, modification | 50S ribosomal protein L15 | 9 | 8 | 8 | -3.73 | 0.77 | -0.71 | -0.60 |
| SCO4723 | *adk* | Purine ribonucleotide biosynthesis | adenylate kinase (fragment) "adenylate kinase" | 4 | 8 | 4 | -2.45 | -0.18 | -0.17 | 0.31 |
| SCO4725 | *infA* | Proteins - translation and modification | translational initiation factor IF1 | 4 | 8 | 8 | -2.74 | -1.66 | -0.11 | 0.17 |
| SCO4734 | *rplM* | Ribosomal proteins - synthesis, modification | 50S ribosomal protein L13 | 9 | 8 | 8 | -5.08 | -0.62 | -0.84 | 0.01 |
| SCO4735 | *rpsI* | Ribosomal proteins - synthesis, modification | 30S ribosomal protein S9 | 8 | 8 | 8 | -3.92 | 0.66 | -1.03 | -0.43 |
| SCO4770 | *guaB* | Purine ribonucleotide biosynthesis | inosine 5' monophosphate dehydrogenase | 11 | 8 | 8 | -3.98 | -0.17 | -0.83 | -0.02 |
| SCO4785 | *guaA* | Purine ribonucleotide biosynthesis | GMP synthase | 5 | 8 | 6 | -4.88 | -0.36 | -2.32 | -0.03 |
| SCO4886 | | Transport/binding proteins | putative sugar ABC transporter ATP-binding protei | 3 | 8 | 4 | -3.53 | 0.03 | -0.59 | 0.13 |
| SCO4965 | | RNA synthesis, modification, DNA transcript'n | putative transcription elongation factor | 3 | 8 | 7 | -3.69 | -0.73 | -1.58 | 0.39 |

| | | | | | | | | | | |
|---|---|---|---|---|---|---|---|---|---|---|
| SCO5031 | *ahpD* | Detoxification | alkyl hydroperoxide reductase system hypothetical | 4 | 8 | 8 | -4.77 | 0.65 | -1.65 | -0.02 |
| SCO5032 | *ahpC* | Detoxification | alkyl hydroperoxide reductase | 9 | 8 | 8 | -5.91 | 0.70 | -3.79 | 0.24 |
| SCO5293 | | Not classified (included putative assignments) | putative oxygenase subunit | 4 | 8 | 6 | -2.35 | -0.04 | -0.23 | 0.45 |
| SCO5367 | *atpB* | ATP-proton motive force | ATP synthase A chain | 2 | 8 | 7 | -5.36 | -0.67 | -0.38 | -0.80 |
| SCO5371 | *atpA* | ATP-proton motive force | ATP synthase alpha chain | 34 | 8 | 8 | -3.05 | 0.04 | -0.59 | -0.46 |
| SCO5374 | *atpC* | ATP-proton motive force | ATP synthase epsilon chain | 4 | 8 | 8 | -2.33 | -0.11 | -0.28 | -0.41 |
| SCO5572 | | Degradation of RNA | ribonuclease III | 3 | 8 | 4 | -4.51 | -0.69 | -1.62 | 0.10 |
| SCO5591 | *rpsP* | Ribosomal proteins - synthesis, modification | 30S ribosomal protein S16 | 15 | 8 | 8 | -3.87 | -2.06 | -1.80 | -0.20 |
| SCO5624 | *rpsB* | Ribosomal proteins - synthesis, modification | 30S ribosomal protein S2 | 24 | 8 | 8 | -4.61 | -0.58 | -1.06 | -0.05 |
| SCO5676 | *gabT* | Pool, multipurpose conversions of intermed. met'm | putative 4-aminobutyrate aminotransferase | 10 | 8 | 8 | -5.84 | -0.53 | -3.80 | -0.05 |
| SCO5737 | *gpsI* | RNA synthesis, modification, DNA transcript'n | guanosine pentaphosphate synthetase/polyribonucle | 26 | 8 | 8 | -4.36 | 1.19 | -2.17 | 0.13 |
| SCO5745 | | Conserved in organism other than Escherichia coli | conserved hypothetical protein SC9A10.09 | 5 | 8 | 7 | -3.86 | 0.25 | -1.59 | -0.27 |
| SCO5805 | *nrdJ* | DNA - replication, repair, restr./modific'n | ribonucleotide reductase | 8 | 8 | 8 | -6.51 | -0.38 | -4.04 | -0.53 |
| SCO7000 | *idh* | Glyoxylate bypass | isocitrate dehydrogenase | 29 | 8 | 8 | -3.89 | 0.75 | -1.40 | 0.12 |
| SCO7510 | *cypH* | Proteins - translation and modification | peptidyl-prolyl cis-trans isomerase | 7 | 8 | 8 | -3.71 | 1.40 | -1.35 | -0.33 |
| | | | ***Genes in Set F*** | | | | | | | |
| SCO0888 | | Gram +ve exported/lipoprotein | putative secreted protein | 2 | 8 | 8 | -2.01 | -0.23 | -5.49 | 0.86 |
| SCO1089 | | No homologues | hypothetical protein 2SCG4.05c | 3 | 8 | 4 | -3.40 | -1.73 | -6.30 | -0.96 |
| SCO1483 | *pyrA* | Pyrimidine ribonucleotide biosynthesis | carbamoylphosphate synthetase large chain | 7 | 8 | 7 | -1.26 | 0.16 | -3.74 | -0.70 |
| SCO1486 | *pyrC* | Pyrimidine ribonucleotide biosynthesis | dihydroorotase | 2 | 8 | 7 | -2.04 | -1.37 | -5.24 | 0.25 |
| SCO1599 | *rpmI* | Ribosomal proteins - synthesis, modification | 50S ribosomal protein L35 | 2 | 8 | 7 | -0.60 | -1.74 | -4.41 | 0.47 |
| SCO1661 | | Aerobic respiration | putative glycerol-3-phosphate dehydrogenase | 2 | 8 | 4 | -0.64 | -0.75 | -4.01 | 0.04 |
| SCO2606 | *orfsyn* | Conserved in organism other than Escherichia coli | hypothetical protein | 2 | 8 | 4 | -0.84 | -1.80 | -3.05 | 0.25 |
| SCO2612 | *ndk* | Nucleotide interconversions | nucleoside diphosphate kinase | 2 | 8 | 7 | -1.80 | 0.83 | -4.14 | -0.13 |
| SCO3381 | *nadC* | Pyridine nucleotide | nicotinate-nucleotide pyrophophorylase | 2 | 8 | 4 | -0.33 | -0.41 | -2.69 | -0.16 |
| SCO3670 | *grpE* | Chaperones | heat chock protein | 6 | 8 | 8 | -0.35 | 2.18 | -3.18 | 0.80 |
| SCO4077 | | No homologues | hypothetical protein SCD25.13 | 3 | 8 | 7 | -0.19 | -0.84 | -3.39 | 0.01 |

| | | | | | | | | | | |
|---|---|---|---|---|---|---|---|---|---|---|
| SCO4469 | *hemL* | Heme, porphyrin | glutamate-1-semialdehyde 2,1-aminomutase | 2 | 8 | 4 | -0.86 | -0.61 | -2.93 | 0.25 |
| SCO5283 | | Response regulator | putative two-component system response regulator | 3 | 8 | 8 | -0.80 | -0.56 | -3.10 | -0.84 |
| SCO5472 | *gcvT* | Amino acids | aminomethyltransferase | 3 | 8 | 7 | -1.92 | -0.50 | -5.29 | -0.54 |
| SCO5556 | | Basic proteins - synthesis, modification | histone-like DNA binding protein | 4 | 8 | 6 | -3.14 | -0.96 | -7.61 | -0.35 |
| SCO6564 | *fabH2* | Fatty acid and phosphatidic acid biosynthesis | 3-oxoacyl-[acyl-carrier-protein] synthase II | 2 | 8 | 6 | -1.05 | 0.17 | -4.01 | 1.02 |
